# Fostamatinib for Hospitalized Adults With COVID-19 and Hypoxemia: A Randomized Clinical Trial

**DOI:** 10.1001/jamanetworkopen.2024.48215

**Published:** 2024-12-03

**Authors:** Sean P. Collins, Matthew S. Shotwell, Jeffrey R. Strich, Kevin W. Gibbs, Marjolein de Wit, D. Clark Files, Michelle Harkins, Kris Hudock, Lisa H. Merck, Ari Moskowitz, Krystle D. Apodaca, Aaron Barksdale, Basmah Safdar, Ali Javaheri, Jeffrey M. Sturek, Harry Schrager, Nicole M. Iovine, Brian Tiffany, Ivor Douglas, Joseph Levitt, Adit A. Ginde, David N. Hager, Nathan Shapiro, Abhijit Duggal, Akram Khan, Michael Lanspa, Peter Chen, Nina Gentile, Estelle Harris, Michelle Gong, Subhashini Sellers, Andrew J. Goodwin, Mark A. Tidswell, Michael Filbin, Neeraj Desai, Felix Gutiérrez, Vicente Estrada, Joaquin Burgos, Tom Boyles, Jose R. Paño-Pardo, Nazreen Hussen, Yves Rosenberg, James Troendle, Gordon R. Bernard, Amanda J. Bistran-Hall, Kelly Walsh, Jonathan D. Casey, Josh DeClercq, Meghan Morrison Joly, Jill Pulley, Todd W. Rice, Jonathan S. Schildcrout, Li Wang, Matthew W. Semler, Wesley H. Self

**Affiliations:** 1 Department of Emergency Medicine, Vanderbilt University Medical Center, Nashville, Tennessee; 2 Veterans Affairs Tennessee Valley Healthcare System, Geriatric Research, Education and Clinical Center, Nashville, Tennessee; 3 Department of Biostatistics, Vanderbilt University Medical Center, Nashville, Tennessee; 4 Critical Care Medicine Branch, National Heart, Lung, and Blood Institute, Bethesda, Maryland; 5 Department of Medicine, Wake Forest University, Winston-Salem, North Carolina; 6 Department of Medicine, Virginia Commonwealth University, Richmond; 7 Department of Internal Medicine, University of New Mexico, Albuquerque; 8 Department of Medicine, University of Cincinnati, Cincinnati, Ohio; 9 Department of Emergency Medicine, Virginia Commonwealth University Health System, Richmond; 10 Department of Medicine, Montefiore Medical Center, Bronx, New York; 11 Department of Medicine, University of Nebraska Medical Center, Omaha; 12 Department of Medicine, Yale University, New Haven, Connecticut; 13 Department of Medicine, Washington University and John Cochran VA Medical Center, St Louis, Missouri; 14 Department of Medicine, University of Virginia, Charlottesville; 15 Department of Medicine, Tufts School of Medicine, Newton-Wellesley Hospital, Newton, Massachusetts; 16 Department of Medicine, University of Florida, Gainesville; 17 Dignity Health, Phoenix, Arizona; 18 Department of Medicine, Denver Health Medical Center, Denver, Colorado; 19 Department of Medicine, Stanford University, Stanford, California; 20 Department of Emergency Medicine, University of Colorado, Aurora; 21 Department of Medicine, Johns Hopkins University, Baltimore, Maryland; 22 Department of Emergency Medicine, Beth Israel Deaconess Medical Center, Boston, Massachusetts; 23 Department of Medicine, Cleveland Clinic Foundation, Cleveland, Ohio; 24 Department of Medicine, Oregon Health and Science University, Portland; 25 Department of Medicine, Intermountain Medical Center, Murray, Utah; 26 Department of Medicine, Cedars-Sinai Medical Center, Los Angeles, California; 27 Department of Emergency Medicine, Temple University, Philadelphia, Pennsylvania; 28 Department of Medicine, University of Utah Health Sciences, Salt Lake City; 29 Department of Medicine, Jack D. Weiler Hospital, Albert Einstein College of Medicine, Bronx, New York; 30 Department of Medicine, University of North Carolina, Chapel Hill; 31 Department of Medicine, Medical University of South Carolina, Charleston; 32 Department of Medicine, Baystate Health, Springfield, Massachusetts; 33 Department of Emergency Medicine, Massachusetts General Hospital, Boston; 34 Department of Medicine, St Alexius Medical Center, Hoffman Estates, Illinois; 35 Department of Medicine, Division of Infectious Diseases, Centro de Investigación Biomédica en Red (CIBERINFEC), Hospital General de Elche & Universidad Miguel Hernández, Alicante, Spain; 36 Hospital Clinico San Carlos, Ciber de Enfermedades Infecciosas, CIBERINFEC, Universidad Complutense de Madrid, Madrid, Spain; 37 Department of Infectious Diseases, Hospital Universitario Vall d’Hebron, Barcelona, Spain; 38 Clinical HIV Research Unit, Helen Joseph Hospital, Johannesburg, South Africa; 39 Department of Infectious Diseases, Hospital Clinico Universitario Lozano Blesa, Jaragoza, Spain; 40 Department of Clinical Research, Worthwhile Clinical Trials (Lakeview Hospital), Benoni, South Africa; 41 National Heart, Lung, and Blood Institute, Bethesda, Maryland; 42 Department of Medicine, Vanderbilt University Medical Center, Nashville, Tennessee; 43 Vanderbilt Institute for Clinical and Translational Research, Vanderbilt University Medical Center, Nashville, Tennessee

## Abstract

**Importance:**

Fostamatinib, a spleen tyrosine kinase inhibitor, has been reported to improve outcomes of COVID-19.

**Objective:**

To evaluate the efficacy and safety of fostamatinib in adults hospitalized with COVID-19 and hypoxemia.

**Design, Setting, and Participants:**

This multicenter, phase 3, placebo-controlled, double-blinded randomized clinical trial was conducted at 41 US sites and 21 international sites between November 17, 2021, and September 27, 2023; the last follow-up visit was December 31, 2023. Participants were adults aged 18 years or older hospitalized with acute SARS-CoV-2 infection and hypoxemia. Data were analyzed between January 10 and March 8, 2024.

**Interventions:**

Fostamatinib, 150 mg orally twice daily for 14 days, or placebo.

**Main Outcomes and Measures:**

The primary outcome was oxygen-free days, an ordinal outcome classifying a participant’s status at day 28 based on mortality and duration of supplemental oxygen use. An adjusted odds ratio (AOR) greater than 1.0 was considered to indicate superiority of fostamatinib over placebo. A key secondary outcome was 28-day all-cause mortality. Safety outcomes included elevated transaminase values, neutropenia, and hypertension.

**Results:**

Of the 400 participants randomized (median age, 67 years [IQR, 58-76 years]; 210 [52.5%] men), 199 received fostamatinib and 201 received placebo. The mean (SD) number of oxygen-free days was 13.4 (12.4) in the fostamatinib group and 14.2 (12.1) in the placebo group (unadjusted mean difference, −1.26 days [95% CI, −3.52 to 1.00 days]; AOR, 0.82 [95% credible interval (CrI), 0.58-1.17]). Mortality at 28 days occurred in 22 of 195 patients (11.3%) in the fostamatinib group and 16 of 197 (8.1%) in the placebo group (AOR, 1.44; 95% CrI, 0.72-2.90). Aspartate aminotransferase elevation occurred more commonly in the fostamatinib group (23 [11.6%]) than in the placebo group (11 [5.5%]; AOR, 2.28; 95% CrI, 1.07-4.84). Other safety outcomes were similar between groups.

**Conclusions and Relevance:**

In this randomized clinical trial of adults hospitalized with COVID-19 and hypoxemia, fostamatinib did not increase the number of oxygen-free days compared with placebo. These results do not support the hypothesis that fostamatinib improves outcomes among adults hospitalized with hypoxemia during the Omicron era.

**Trial Registration:**

ClinicalTrials.gov Identifier: NCT04924660

## Key Points

**Question:**

Does fostamatinib, a spleen tyrosine kinase inhibitor, increase oxygen-free days in adults hospitalized with COVID-19 and hypoxemia during the Omicron era?

**Findings:**

In this randomized clinical trial that included 400 adults hospitalized with COVID-19 and hypoxemia, the mean number of oxygen–free days was not significantly different between patients receiving fostamatinib and those receiving placebo.

**Meaning:**

These findings do not support the hypothesis that fostamatinib increases oxygen-free days among adults hospitalized with COVID-19 and hypoxemia during the Omicron era.

## Teaser

This randomized clinical trial investigates whether fostamatinib increases oxygen-free days among adults hospitalized with COVID-19 and hypoxemia.

## Introduction

Despite advances in the treatment of COVID-19, mortality among patients admitted to the hospital with COVID-19 and hypoxemia remains high.^1^ Whether agents targeting circulating immune cells linked to thromboinflammation can improve outcomes of COVID-19 remains unknown.

Fostamatinib is an oral spleen tyrosine kinase inhibitor approved by the US Food and Drug Administration for the treatment of adult patients with chronic immune thrombocytopenia. Fostamatinib blocks the activation of neutrophils, macrophages, and platelets,^2^ each of which contribute to thromboinflammation in COVID-19. In vitro data have demonstrated the ability of fostamatinib to inhibit the release of neutrophil extracellular traps (NETs) in healthy neutrophils stimulated with COVID-19 plasma.^3^ NETs are weblike structures comprising DNA-histone complexes and immunomodulatory proteins that can cause epithelial and endothelial injury and promote thrombosis.^4,5,6,7,8^ In vitro data have also demonstrated that fostamatinib inhibits spike antigen-antibody complex–mediated activation of macrophages and platelets.^9,10,11^ In addition, analysis of cellular and soluble mediators in serum samples from patients with COVID-19 enrolled in a phase 2 randomized clinical trial demonstrated that fostamatinib was associated with decreased myeloid activation.^12^ Through these mechanisms, fostamatinib may dampen inflammatory pathways associated with thromboinflammation and mitigate acute lung injury, hastening recovery from COVID-19.

In a previous phase 2 study,^13^ fostamatinib was safe and resulted in a greater mean change in an 8-point COVID-19 ordinal scale of patient clinical status in adults hospitalized with COVID-19. A preliminary report from an unpublished phase 3 trial found that fostamatinib increased the number of days free of supplemental oxygen in adults hospitalized with COVID-19.^14,15^ To test the hypothesis that fostamatinib improves outcomes for patients with COVID-19, we performed a phase 3, placebo-controlled, randomized clinical trial examining the effects of fostamatinib on oxygen-free days in adults hospitalized with COVID-19 and hypoxemia.

## Methods

### Trial Oversight

The US National Institutes of Health (NIH) established the fourth Accelerating COVID-19 Therapeutic Interventions and Vaccines (ACTIV-4) program to rapidly evaluate therapies targeting the host response for the treatment of COVID-19.^16^ A description of the platform and the results of the first 2 trials have been published previously.^17,18^ In this randomized clinical trial, the ACTIV-4 Host Tissue platform was coordinated by Vanderbilt University Medical Center. The trial was overseen by 2 bodies: a data safety and monitoring board (DSMB) appointed by the National Heart, Lung, and Blood Institute of the NIH and the Vanderbilt University institutional review board. The platform was registered at ClinicalTrials.gov prior to enrollment (NCT04924660). Agents were investigated on the platform under a US Food and Drug Administration Investigational New Drug application. Written informed consent was obtained from all participants or surrogates prior to trial procedures. This manuscript was written in accordance with the Consolidated Standards of Reporting Trials (CONSORT).^19^

### Trial Design

This report describes the third trial of the ACTIV-4 Host Tissue platform (trial protocol and statistical analysis plan in Supplement 1). This trial ran from November 17, 2021, to September 27, 2023, at 62 hospitals, including 41 hospitals in the US and 21 hospitals outside the US (eTable 1 in Supplement 2). From November 17, 2021, to April 20, 2022, the fostamatinib trial was part of a platform including 3 total trials (eFigure 1 in Supplement 2). The other 2 trials enrolling at that time studied TXA-127 and TRV-027.^17^ Fostamatinib trial enrollment was from November 17, 2021, to September 27, 2023, with the last follow-up visit on December 31, 2023.

During the period when all 3 trials were concurrently enrolling on the platform, participants were randomized based on their eligibility to fostamatinib, TXA-127, TRV-027, or placebo. TXA-127 and TRV-027 had a matching intravenous placebo, while fostamatinib had a matching placebo pill. The trials shared placebo participants, such that each trial included participants randomized to the relevant active agent and all placebo participants eligible for that trial regardless of which type (intravenous vs pill) of placebo they received. On April 21, 2022, the TRV-027 and TXA-127 trials were stopped.^17^

### Patient Population

The trial included patients aged 18 years or older hospitalized for SARS-CoV-2 infection, defined as a positive SARS-CoV-2 molecular or antigen test result within 72 hours of randomization, and new-onset hypoxemia, defined as an oxygen saturation (Spo_2_) measured by pulse oximetry of less than 92% on room air, use of supplemental oxygen to maintain an Spo_2_ greater than 92%, or an increased oxygen requirement for patients who were receiving supplemental oxygen before their COVID-19 diagnosis to maintain their baseline Spo_2_. A complete list of eligibility criteria is available in eTable 2 in Supplement 2.

Race and ethnicity were collected by self-report using mutually exclusive categories provided by the trial’s case report form to report the demographic characteristics of the trial population. Race categories were American Indian or Alaska Native, Asian, Black, Middle Eastern or Northern African, Native Hawaiian or Other Pacific Islander, White, and other race or preferred not to answer. Ethnicity categories were Hispanic, not Hispanic, and other ethnicity or preferred not to answer. Other indicated that the listed categories did not apply.

### Randomization

Eligibility criteria were entered into a centralized randomization system (REDCap).^20^ Patients were randomized to ensure balance between the group receiving the active agent and the pooled placebo group for each trial. If the patient was eligible for more than 1 enrolling trial, the patient was randomized in equal ratios to a specific trial. Participants were then assigned to the active agent or placebo in an *m*:1 ratio, with *m* representing the number of eligible trials. For example, if a patient was eligible for all 3 trials, they were randomized in a 1:1:1 ratio to TXA-127, TRV-027, or fostamatinib and in a 3:1 ratio to active agent or placebo. If randomized to placebo, the patient received a placebo mimic of the medication corresponding to the assigned trial—TXA-127 or TRV-027 (both intravenous infusions) or fostamatinib (pill)—but would contribute to the pooled placebo group for each of the 3 trials. Randomization was implemented using permuted blocks stratified by site and trial eligibility. From April 20, 2022, through September 27, 2023, the fostamatinib trial was the only enrolling trial. During this period, participants were randomized 1:1 to fostamatinib or placebo.

### Blinding

While the 3 trials were enrolling concurrently, participants, treating clinicians, and trial personnel were blinded to active agent or placebo assignment but not blinded to the specific trial. Participants assigned to TXA-127 received 3-hour active agent or placebo infusion daily for 5 days, those assigned to TRV-027 received continuous active agent or placebo infusion for 5 days, and those assigned to fostamatinib received an active agent or placebo pill twice daily for 14 days. This design enabled blinded comparisons of active agent vs placebo for multiple trials on the same platform using agents administered by different routes and dosages and avoided a double dummy placebo requirement. Once the TRV-027 and TXA-127 trials were stopped, blinding was continued using an oral placebo identical to the fostamatinib active compound. Blinding was maintained for each trial until all follow-up was complete and the database was locked.

### Trial Interventions

Fostamatinib was administered at a dosage of 150 mg by mouth twice daily for 14 days. This study dose and duration was chosen based on the prior 2 fostamatinib trials suggesting efficacy.^13,14,15^ Study medication was continued on an outpatient basis if the patient was discharged prior to completing 28 doses. Treatment days were not added to the 14-day course if the study drug was held and then restarted, and the maximum treatment course was 28 pills. For participants unable to swallow, tablets were crushed and administered through an enteral tube.

### Study Drug Modification and Discontinuation for Safety

The trial protocol (Supplement 1) included criteria for both modifying the dose of the trial drug and stopping the study drug. When the study drug was restarted at 100 mg, the study team and patient remained blinded and the unblinded study pharmacist dispensed the corresponding 100-mg pill, either placebo or fostamatinib, depending on treatment allocation.

### Outcomes

The primary outcome was oxygen-free days, an ordinal outcome classifying a patient’s status at day 28 based on mortality and duration of supplemental oxygen use. The rationale and design of this outcome was previously published.^18^ This outcome was chosen to characterize recovery from acute lung injury using 2 patient-centered outcomes: mortality and hypoxia-related lung dysfunction. These 2 outcomes have been the focus of therapeutic interventions during inpatient management of patients with COVID-19 hypoxemia. Improvement in lung injury would be reflected by improvement in oxygen-free days. Oxygen-free days were calculated as 28 days minus the number of days between initiation of and final liberation from new supplemental oxygen use during the 28 days following randomization. Participants receiving long-term supplemental oxygen before a COVID-19 diagnosis were classified as liberated from new supplemental oxygen when their oxygen flow rate returned to their baseline level. Participants who died before day 28 were assigned a value of −1, signifying an outcome worse than the 0 value due to ongoing oxygen need at day 28. Participants who started the trial with new supplemental oxygen and continued to receive it through day 28 were coded as having 0 oxygen-free days. Hence, oxygen-free days was an ordinal outcome with 30 levels, ranging from −1 to 28. Patients were followed up daily in the hospital to document oxygen use and had telephone follow-up through day 28 if discharged. If the participant used supplemental oxygen for any part of a day, that day was counted as “on oxygen.” For patients who were intermittently receiving supplemental oxygen during the weaning process, we counted all days as being “on oxygen” until the last day of oxygen use.

The 3 key secondary efficacy outcomes included 28-day all-cause mortality, alive and free of respiratory failure at day 28 (defined as alive and not receiving high-flow nasal oxygen, noninvasive ventilation, or invasive mechanical ventilation at day 28), and status on the 8-level World Health Organization (WHO) COVID-19 clinical progression ordinal scale at day 28.^21^ The scale consisted of ambulatory (levels 1 and 2), hospitalized without use of supplemental oxygen (level 3), hospitalized with increasingly invasive lung and other organ support (simple supplemental oxygen by nasal prongs or mask [level 4], high-flow nasal oxygen or noninvasive ventilation [level 5], invasive mechanical ventilation [level 6], or invasive mechanical ventilation plus other organ support [level 7]), and death (level 8). The trial protocol (Supplement 1) specified 3 key safety outcomes: elevation in transaminase values through day 28, neutropenia through day 28, and hypertension through day 28. We defined elevated transaminase values to be 4 times a conservative upper limit of normal (ULN).^22^ At the request of the DSMB after a July 20, 2022, meeting, we proposed a definition and plan to collect adverse events of special interest (AESIs) for the DSMB on September 21, 2022, and this was accepted. We systematically collected AESIs from that point forward. These events were defined as the occurrence of an aspartate aminotransferase (AST) or alanine aminotransferase (ALT) value more than 5 times the local laboratory’s ULN or the baseline value (if above the ULN) at the time of randomization through day 28. A complete list of trial outcomes is available in eTable 3 in Supplement 2, and a complete list of protocol-specified exempt serious events (PSESEs) is available in eTable 4 in Supplement 2.

### Halting Enrollment

At the first interim analysis (200 participants enrolled), the efficacy probability for the primary outcome was 0.152, and enrollment was continued. At the request of the DSMB, we provided conditional power after enrollment of and outcome collection for 364 participants. The conditional power was the probability of concluding efficacy regarding the primary outcome if the trial completed enrollment (600 participants), conditional on the outcome data for the first 364 participants. The DSMB recommended halting enrollment on September 27, 2023, based on low conditional power.

### Statistical Analysis

Details of the statistical approach are available in the statistical analysis plan (Supplement 1). The modified intention-to-treat (mITT) primary analysis population included all eligible, randomized participants who received any amount of the assigned study drug.

The primary outcome, oxygen-free days, was analyzed using a bayesian multivariable proportional odds logistic regression (POLR) model with active vs placebo study drug as the primary independent variable, adjusted for age group (18-30, 31-64, and ≥65 years), sex at birth, and baseline level of oxygen support on the WHO COVID-19 ordinal scale (level 3 or 4 was no oxygen or oxygen receipt via standard nasal cannula or mask; level 5, high-flow nasal cannula or noninvasive ventilation; and level 6 or 7, invasive mechanical ventilation). A noninformative flat prior was used for all model parameters. The effect of treatment on oxygen-free days was quantified using an adjusted odds ratio (AOR) and 95% equal-tailed credible interval (CrI). An AOR greater than 1.0 indicated superiority (more oxygen-free days in the active agent group vs placebo group), and an AOR less than 1.0 indicated inferiority. The efficacy probability was the posterior probability that the adjusted AOR exceeded 1.0. Regression diagnostics included assessment of the proportional odds assumption and sensitivity analyses as described in the statistical analysis plan (Supplement 1). No evidence was found that the proportional odds assumption was violated (eFigure 2 in Supplement 2). Due to the irregular distribution of oxygen-free days, POLR was selected in favor of less flexible methods, such as Poisson or negative binomial regression.

All trials in the ACTIV-4 Host Tissue platform were powered based on the primary outcome of oxygen-free days and its distribution in a prior COVID-19 trial.^23^ Prior to enrollment, using statistical simulation, we calculated that 600 participants would provide 85% power to detect an AOR of 1.65, corresponding to an increase of 3.1 oxygen-free days and an absolute mortality reduction of 7.8% in the active agent group compared with the placebo group. This difference in oxygen-free days aligned with prior trials and was a clinically important difference to patients.^18,24,25,26^ Details of the simulations used for sample size calculations are available in the statistical analysis plan (Supplement 1).

Planned interim analyses were scheduled after one-third (200 participants) and two-thirds (400 participants) of the total planned sample size had reached primary outcome ascertainment. The DSMB was instructed to halt enrollment if the efficacy probability at an interim analysis was less than 5%. At the final analysis, if enrollment was not previously halted, a conclusion of superiority was indicated if the efficacy probability exceeded 0.976, a threshold selected to ensure a type I error rate of less than 2.5%. Inferiority was concluded if the efficacy probability was less than 0.05 at the final analysis.

In separate analyses, differential treatment effect was evaluated by adding an interaction term between prespecified baseline characteristics and trial group assignment to the primary analysis model for oxygen-free days.^27^ Baseline characteristics evaluated included age, COVID-19 vaccination status, respiratory support (classified with the WHO COVID-19 clinical progression ordinal scale), and use of an angiotensin-converting enzyme inhibitor or angiotensin receptor blocker at baseline. The rationale for evaluating differential treatment effect by patients’ severity of illness at enrollment was the findings of 2 previous trials^13,14,15^ suggesting fostamatinib might have greater efficacy in patients with more severe disease. An additional post hoc analysis was performed to examine the effect of trial group assignment on outcomes by comparing patients enrolled earlier in the pandemic (year 1) with patients enrolled later, when vaccination was more common, other concomitant COVID-19 therapies were being used, and the most common variant was Omicron (year 2).

Secondary efficacy and safety outcomes were analyzed with regression models using the same covariables as the primary model. A gatekeeping method was used to ensure a type I error rate of less than 2.5% across the family of primary and key secondary outcomes but not the safety outcomes; the key secondary outcomes were tested, in the specified order, only if the active drug was superior to placebo for the preceding outcome. For key secondary outcomes, the posterior efficacy probability was the probability of an AOR in the direction corresponding to a better outcome: AOR less than 1.0 for 28-day all-cause mortality, AOR greater than 1.0 for alive and free of respiratory failure at day 28, and AOR less than 1.0 for WHO COVID-19 clinical progression status at day 28. Superiority was indicated if the posterior efficacy probability exceeded 0.975. Systematically collected safety events, PSESEs, and adverse events were reported with frequency counts and proportions.

Notwithstanding the aforementioned formal superiority and inferiority assessments, estimates with a 95% CrI excluding the null were considered statistically significant. The widths of the 95% CrI were not adjusted for multiplicity. Missing and partially observed outcomes were analyzed using a likelihood-based method without imputation, as detailed in the statistical analysis plan (Supplement 1). Outcome summaries excluded participants who withdrew consent or were lost to follow-up prior to the outcome assessment. Data were analyzed between January 10 and March 8, 2024. Statistical analyses were conducted using R, version 4.2.0 (R Project for Statistical Computing) with packages rms, rmsb, and ordinal. Analyses of the primary and key secondary outcomes used a custom, validated implementation of the POLR model to account for partially observed outcomes.

## Results

### Participants

**Figure 1.**
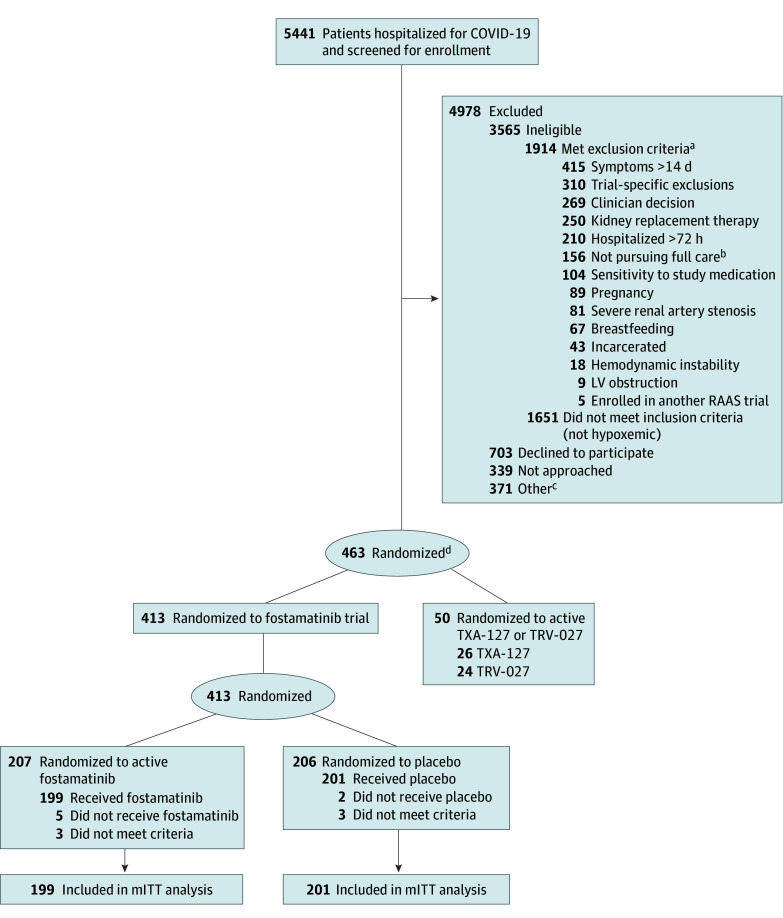
Patient Screening, Randomization, and Participation in the Fostamatinib Trial LV indicates left ventricular; mITT, modified intention-to-treat; RAAS, renin angiotensin aldosterone system. ^a^The criteria were not mutually exclusive; some potential participants met multiple criteria for ineligibility. ^b^The patient, clinical team, or both was not pursuing full medical management (eg, a do-not-intubate order). ^c^The patient was to be discharged from the hospital before the study procedures could be initiated, the patient was enrolled in another trial, or the patient’s situation presented logistical challenges for trial enrollment. ^d^Patients eligible for more than 1 trial were randomized with equal probability to a specific trial. The Randomization subsection of the Methods section gives more details on randomization.

Of the 5441 patients screened for the ACTIV-4 Host Tissue platform trials, 463 were eligible for the fostamatinib trial and randomized (Figure 1). Among those, 26 were assigned to active TXA-127 and 24 to active TRV-027 and excluded from the fostamatinib trial. The final number of patients enrolled in the fostamatinib trial, including those whose outcome data were not yet available at the time that enrollment was halted, was 413, with 7 patients identified as ineligible after randomization and 6 patients receiving no doses of study drug. Thus, 400 participants were analyzed in the mITT population (199 in the fostamatinib group and 201 in the placebo group) (Table 1). Median patient age was 67 years (IQR, 58-76 years); 210 (52.5%) were men, and 190 (47.5%) were women. A total of 6 patients (1.5%) were American Indian or Alaska Native; 12 (3.0%), Asian; 47 (11.8%), Black; 1 (0.2%), Middle Eastern or Northern African; 3 (0.8%), Native Hawaiian or Other Pacific Islander; 298 (74.5%), White; and 33 (8.3%), other race or preferred not to answer. Fifty-six patients (14.0%) were Hispanic; 329 (82.2%), not Hispanic; and 15 (3.8%), other ethnicity or preferred not to answer. Altogether, 289 patients (72.2%) had been vaccinated against COVID-19, 46 (11.5%) used supplemental oxygen at home prior to admission, and 103 (25.8%) were receiving high-flow oxygen therapy or noninvasive or invasive mechanical ventilation at enrollment (Table 1). Patients in both groups received similar concomitant therapies at the time of randomization and during the time of study drug administration: 344 (86.0%) were receiving concomitant corticosteroids, and 321 (80.3%) were receiving remdesivir. By study day 28, 6 participants (1.5%) were lost to follow-up (3 [1.5%] in each group) (eFigure 3 in Supplement 2).

**Table 1.** Patient Characteristics at Baseline

Characteristic	Patients^a^	
	Fostamatinib (n = 199)	Placebo (n = 201)
Age, y		
18-30	7 (3.5)	5 (2.5)
31-64	80 (40.2)	74 (36.8)
≥65	112 (56.3)	122 (60.7)
Sex at birth		
Female	99 (49.7)	91 (45.3)
Male	100 (50.3)	110 (54.7)
Race^b^		
American Indian or Alaska Native	2 (1.0)	4 (2.0)
Asian	6 (3.0)	6 (3.0)
Black	24 (12.1)	23 (11.4)
Middle Eastern or Northern African	1 (0.5)	0
Native Hawaiian or Other Pacific Islander	3 (1.5)	0
White	142 (71.4)	156 (77.6)
Other or preferred not to answer	21 (10.6)	12 (6.0)
Ethnicity^b^		
Hispanic	27 (13.6)	29 (14.4)
Not Hispanic	167 (83.9)	162 (80.6)
Other or preferred not to answer	5 (2.5)	10 (5.0)
Country		
US	186 (93.5)	188 (93.5)
Spain	9 (4.5)	9 (4.5)
South Africa	2 (1.0)	3 (1.5)
Brazil	1 (0.5)	0
Italy	1 (0.5)	0
Germany	0	1 (0.5)
Long-term supplemental oxygen use prior to COVID-19 diagnosis	23 (11.6)	23 (11.4)
Patient-reported chronic medical conditions		
Hypertension	122 (61.3)	135 (67.2)
Obesity^c^	84 (42.2)	94 (46.8)
Diabetes (with or without end-organ damage)	73 (36.7)	79 (39.3)
Chronic pulmonary disease	72 (36.2)	71 (35.3)
Chronic heart failure	45 (22.6)	50 (24.9)
Chronic kidney disease (not receiving renal replacement therapy)	35 (17.6)	41 (20.4)
Active cancer	20 (10.1)	23 (11.4)
Cirrhosis	10 (5.0)	8 (4.0)
Dementia	9 (4.5)	8 (4.0)
Long-term medication use		
ACE inhibitor	10 (5.0)	12 (6.0)
ARB	11 (5.5)	13 (6.5)
Predominant SARS-CoV-2 variant in US at time of enrollment		
Delta (before December 26, 2021)	12 (6.0)	11 (5.5)
Omicron (after December 26, 2021)	187 (94.0)	190 (94.5)
Receipt of ≥1 COVID-19 vaccine	144 (72.4)	145 (72.1)
WHO COVID-19 clinical progression scale at randomization^d^		
3	1 (0.5)	1 (0.5)
4	147 (73.9)	148 (73.6)
5	41 (20.6)	44 (21.9)
6	10 (5.0)	8 (4.0)
7	0	0
Time from hospitalization to randomization, median (IQR), d	1 (1-2)	1 (1-2)
Vasopressor use on day of randomization^e^	7 (3.5)	7 (3.5)
Acute in-hospital treatments for COVID-19 prior to randomization		
Corticosteroids	167 (83.9)	177 (88.1)
Anticoagulants	172 (86.4)	172 (85.6)
Remdesivir	160 (80.4)	161 (80.1)
Baricitinib	18 (9.1)	22 (10.9)
Tocilizumab	8 (4.0)	9 (4.5)

Abbreviations: ACE, angiotensin converting enzyme; ARB, angiotensin receptor blocker; WHO, World Health Organizations.

^a^ Data are presented as number (percentage) of patients unless otherwise indicated.

^b^ Race and ethnicity were self-reported using mutually exclusive categories. “Other” was selected when the remaining categories did not apply.

^c^ Defined as body mass index of 30 or greater, calculated as weight in kilograms divided by height in meters squared.

^d^ The WHO COVID-19 clinical progression scale is described in the Outcomes subsection of the Methods section, with additional details in eTable 3 in Supplement 2.

^e^ Includes any use of vasopressors or inotropes, including but not limited to dobutamine, dopamine, epinephrine, milrinone, norepinephrine, phenylephrine, and vasopressin.

### Study Drug Delivery

Among the 201 participants randomized to placebo, 12 (6.0%) received a placebo mimic of TXA-127 or TRV-027 by intravenous infusion and 189 (94.0%) received oral placebo. Participants received a median of 27 (IQR, 10.0-28.0) of 28 possible doses overall, 26 (IQR, 10.0-28.0) doses in the fostamatinib group, and 27 (IQR, 10.0-28.0) doses in the placebo group. In the fostamatinib group, participants received 7867 of 10 864 possible oral doses (72.4%). Among the 2997 doses not received (27.6%), the most common reasons were study drug discontinuation due to adverse events (714 doses [23.8%]) and death prior to the time the dose was scheduled (199 doses [6.6%]) (eTable 5 in Supplement 2). The proportion of patients who experienced any study drug discontinuation due to adverse events was 39 (19.6%) in the fostamatinib arm and 32 (15.9%) in the placebo arm, including temporary (24 [12.1%] in the fostamatinib arm and 15 [7.5%] in the placebo arm) or permanent (23 [11.6%] in the fostamatinib arm and 19 [9.5%] in the placebo arm) study drug discontinuation.

### Primary Outcome

**Figure 2.**
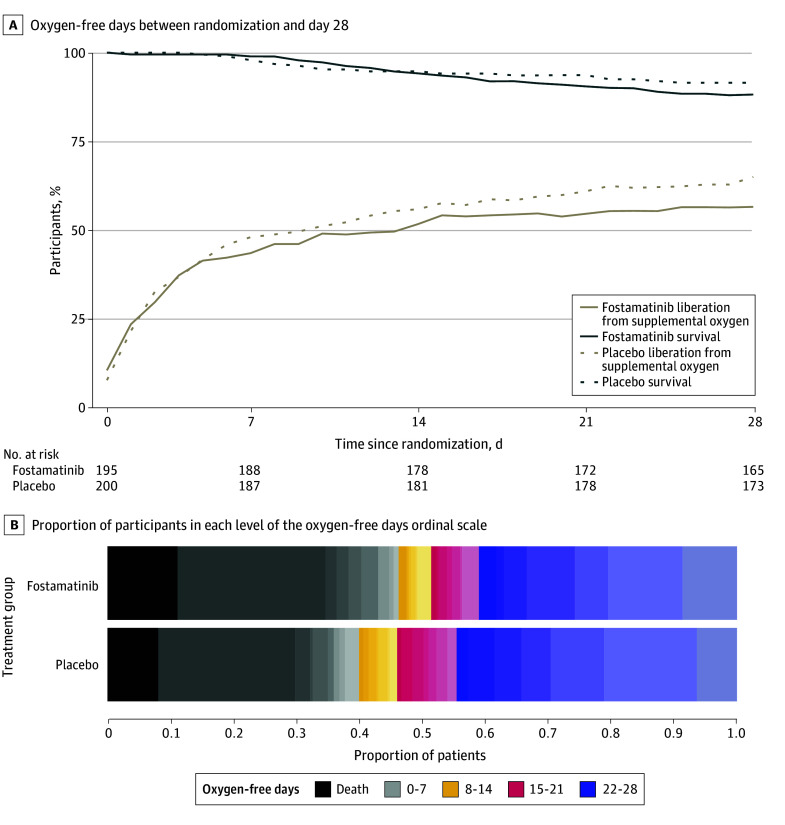
Primary Outcome of Oxygen-Free Days Between Randomization and Day 28 The day of randomization was study day 0. The total sample size was 400 participants in the fostamatinib trial. Participants were followed up until the earlier of death or day 28. A, The proportion of participants who were deceased or liberated from supplemental oxygen was calculated using the number of patients for whom information was available for that day. Incomplete follow-up, especially pertaining to oxygenation status, meant that some participants were not included in the calculation for that day, which along with allowing for patients to resume supplemental oxygen after a period of liberation, led to nonmonotonicity in the figure. B, The oxygen-free days outcome demonstrated null results for fostamatinib vs placebo, with point estimates in the direction of inferiority (adjusted odds ratio [AOR], 0.82; 95% credible interval, 0.58-1.17).

**Figure 3.**
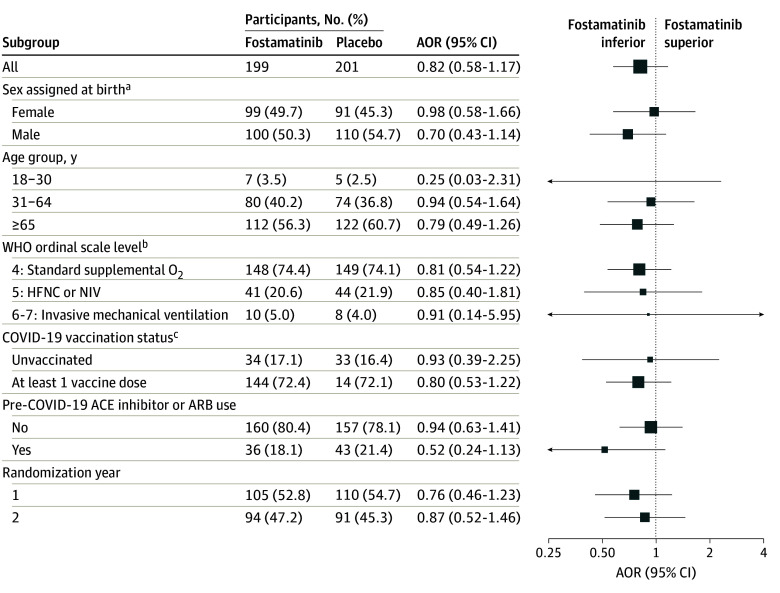
Differential Treatment Effect Odds ratios were adjusted for sex, age group, and baseline World Health Organization (WHO) ordinal scale level. No formal test of heterogeneity of treatment effect was implemented. An oxygen-free day was calculated as 28 minus the number of days between randomization (day 0) and liberation from new supplemental oxygen use during the 28 days. Participants who died before day 28 were coded as having −1 oxygen-free days (worst possible outcome). The subgroup analyses of oxygen-free days included participants with partially observed data (only known to be within a certain range). Additional information appears in eFigure 3 in Supplement 2, including the number of participants with partially observed oxygen-free days. ACE indicates angiotensin-converting enzyme; AOR, adjusted odds ratio; ARB, angiotensin receptor blocker; HFNC, high-flow nasal cannula; NIV, noninvasive. ^a^Not prespecified as a subgrouping variable; thus, differential treatment effect by sex was a post hoc analysis. ^b^The WHO COVID-19 clinical progression scale is described in the Outcomes subsection of the Methods section. There were 2 participants (1 in the placebo arm, 1 in the fostamatinib arm) with baseline WHO level of 3 who were hypoxemic and thus qualified for the trial and none with a WHO level of 7 at baseline. ^c^Participants with unknown vaccination status were excluded (23 receiving placebo and 21 receiving fostamatinib).

The mean (SD) number of oxygen-free days was 13.4 (12.4) in the fostamatinib group and 14.2 (12.1) in the placebo group (unadjusted mean difference, −1.26 days [95% CI, −3.52 to 1.00 days]; AOR, 0.82 [95% CrI, 0.58-1.17]; posterior probability of superiority, 0.14) (Table 2 and Figure 2). In the analyses to assess differential treatment effect, the point estimates were similar across all groups (Figure 3).

**Table 2.** Summary of Primary, Key Secondary, and Safety Outcomes

Outcome	Patients, No./total No. (%)		Unadjusted absolute difference (95% CI)^a^	AOR (95% CrI)^b^
	Fostamatinib (n = 199)	Placebo (n = 201)		
Primary outcome: oxygen-free days to day 28, mean (SD)^c^	13.4 (12.4)	14.2 (12.1)	−0.8 (−3.3 to 1.7)	0.82 (0.58 to 1.17)
Key secondary outcomes^d^				
Mortality at day 28	22/195 (11.3)	16/197 (8.1)	3.2 (−2.7 to 9.1)	1.44 (0.72 to 2.90)
Alive and respiratory failure free at day 28^e^	159/186 (85.5)	169/190 (88.9)	−3.5 (−10.3 to 3.2)	0.71 (0.37 to 1.36)
WHO COVID-19 clinical progression ordinal scale level at day 28^f^				
1	65/182 (35.7)	74/189 (39.2)	−3.4 (−13.2 to 6.4)	1.39 (0.95 to 2.04)
2	76/182 (41.8)	88/189 (46.6)	−4.8 (−14.8 to 5.3)	
3	6/182 (3.3)	3/189 (1.6)	1.7 (−1.3 to 5.1)	
4	8/182 (4.4)	3/189 (1.6)	2.8 (−0.5 to 6.5)	
5	1/182 (0.5)	2/189 (1.1)	−0.5 (−2.5 to 1.3)	
6	1/182 (0.5)	1/189 (0.5)	0.02 (−1.6 to 1.6)	
7	3/182 (1.7)	2/189 (1.1)	0.6 (−1.7 to 3.1)	
8	22/182 (12.1)	16/189 (8.5)	3.6 (−2.5 to 9.9)	
Prespecified safety outcomes through day 28^g^				
Hypertension	24/199 (12.1)	19/201 (9.5)	2.6 (−3.4 to 8.7)	0.75 (0.39 to 1.43)
ANC <500 cells/μL	17/199 (8.5)	9/201 (4.5)	4.1 (−0.7 to 9.0)	2.01 (0.86 to 4.67)
ALT >136 U/L for men or >96 U/L for women	14/199 (7.0)	14/201 (7.0)	0.1 (−4.9 to 5.1)	0.98 (0.45 to 2.14)
AST >128 U/L for men or >104 U/L for women	23/199 (11.6)	11/201 (5.5)	6.1 (0.7 to 11.6)	2.28 (1.07 to 4.84)

Abbreviations: ALT, alanine aminotransferase; ANC, absolute neutrophil count; AOR, adjusted odds ratio; AST, aspartate aminotransferase; CrI, credible interval; WHO, World Health Organization.

SI conversion: To convert ALT and AST to μkat/L, multiply by 0.0167.

^a^ Binomial distribution with improper β prior was used for each binary outcome.

^b^ Calculated using the regression techniques described in the Statistical Analysis subsection of the Methods section.

^c^ Oxygen-free days were calculated as 28 minus the number of days between initiation and final liberation from new supplemental oxygen use during the 28 days following randomization. Participants who died before day 28 were coded as having −1 oxygen-free days (worst possible outcome). The primary analysis of oxygen-free days included all participants, including those with partially observed data (participants for whom the number of oxygen-free days was not known precisely but was known to be within a certain range) (eFigure 3 in Supplement 2). The number of participants with partially observed data included 33 in the fostamatinib active group and 20 in the placebo group. The mean (SD) estimates exclude participants with partially observed oxygen-free days.

^d^ Additional secondary outcomes are presented in eFigure 4 and safety outcomes in eTable 7 in Supplement 2.

^e^ Defined as alive and not receiving high-flow nasal oxygen, noninvasive ventilation, or invasive ventilation.

^f^ The WHO COVID-19 clinical progression scale is described in the Outcomes subsection of the Methods section, with additional details in eTable 3 in Supplement 2.

^g^ All safety outcomes were based on clinician assessment during routine follow-up.

### Secondary Outcomes

Among the 392 participants with complete data at day 28 (98.0%), mortality occurred in 22 of 195 in the fostamatinib group (11.3%) and 16 of 197 in the placebo group (8.1%; AOR, 1.44; 95% CrI, 0.72-2.90) (Table 2 and eFigure 4 in Supplement 2). At day 28, 159 of 186 participants in the fostamatinib group (85.5%) and 169 of 190 in the placebo group (88.9%) were alive and free of respiratory failure (AOR, 0.71; 95% CrI, 0.37-1.36). Participants’ status on the 8-level WHO COVID-19 clinical progression ordinal scale at day 28 was a 1 or 2 in 141 of 182 participants in the fostamatinib group (77.5%) and 162 of 189 in the placebo group (85.7%; AOR, 1.39; 95% CrI, 0.95-2.04).

### Safety Outcomes and Adverse Events

The proportion of participants who experienced an AST value greater than 128 U/L (men) or 104 U/L (women) was greater in the fostamatinib group (23 [11.6%]) than in the placebo group (11 [5.5%]; AOR, 2.28; 95% CrI, 1.07-4.84) (to convert AST to μkat/L, multiply by 0.0167). The proportion of patients in each group who experienced an ALT value greater than 136 U/L for men or 96 U/L for women was similar (14 [7.0%] vs 14 [7.0%]; AOR, 0.98; 95% CrI, 0.45-2.14) (to convert ALT to μkat/L, multiply by 0.0167). The frequency of other safety outcomes, PSESEs (eTable 7 in Supplement 2), and serious adverse events (eTable 6 in Supplement 2) was similar between groups.

## Discussion

In this randomized clinical trial involving 400 adults hospitalized with COVID-19 and hypoxemia, fostamatinib did not increase the number of days alive and free from supplemental oxygen therapy or decrease mortality. These results suggest that fostamatinib did not improve recovery from COVID-19 in a population of largely vaccinated patients during the Omicron era.

The 2 previous fostamatinib trials^13,14,15^ suggested benefit in patients hospitalized with COVID-19, particularly patients with increased severity of lung injury, and this was the rationale for the design of the current trial. Our trial, however, did not find fostamatinib effective for increasing the number of oxygen-free days in hospitalized adults with COVID-19 despite enrolling a trial population in which 25.8% of participants were receiving high-flow nasal cannula, noninvasive ventilation, or invasive mechanical ventilation at the time of randomization. Our differential treatment effect analysis evaluating differences in oxygen-free days stratified by WHO COVID-19 severity status at randomization also did not suggest efficacy.

At least 5 potential explanations exist for the difference in findings between the current trial and the previous trials.^13,14,15^ First, the large sample size in this phase 3 trial may have provided more precise estimates of treatment effect and a lower risk of type I error than prior, earlier-phase studies lacking familywise type I error control.^13,14,15^ As often happens with confirmatory trials, a larger sample size in our trial allowed a better estimation of differences in efficacy not favoring treatment with fostamatinib. Second, the 2 previous trials were conducted earlier in the pandemic, when most participants were unvaccinated.^13,14,15^ In our trial, 72.3% of participants (n = 289) were vaccinated, and vaccination possibly diminished participants’ inflammatory response, decreasing the opportunity for benefit from a drug targeting immune dysregulation.^28,29^ However, in our differential treatment effect analysis, there was no evidence of a significant difference in efficacy between vaccinated and unvaccinated participants. Third, differences in the predominant SARS-CoV-2 virus variant between the previous trials and our trial may have led to differences in vaccine response, pathogenesis of disease, and clinical manifestations and response to treatment with fostamatinib. Fourth, compared with the trials conducted earlier in the pandemic,^30,31^ the participants in our trial were older and had a greater number of underlying chronic medical conditions, including chronic lung disease and long-term receipt of supplemental oxygen. This may have resulted in a lower proportion of hypoxemia cases being attributable to lung injury from COVID-19 and, therefore, more cases potentially less amenable to a drug targeting altered immune responses. Fifth, administration of antivirals was infrequent in the prior large fostamatinib trial.^14,15^ In contrast, these treatments were commonly administered in our trial, possibly diminishing any additive effect of fostamatinib.

Recent research has highlighted heterogeneity in the development and repair of lung injury.^32^ Whether the effect of fostamatinib on outcomes differs for participants with vs without biomarkers of proinflammatory cellular response, including NET formation, may help inform whether fostamatinib should be evaluated in other thromboinflammatory processes.^3,12^

### Limitations

Our trial has limitations. Fostamatinib was studied in addition to usual COVID-19 treatments, including corticosteroids and remdesivir for most participants. Thus, the effect of fostamatinib independent of other COVID-19 treatments could not be evaluated. Second, halting enrollment resulted in even lower power for our key secondary outcomes. Third, we do not yet have mechanistic data to inform whether receipt of fostamatinib affected soluble mediators of immune dysregulation associated with thromboinflammation, the mechanism by which fostamatinib is hypothesized to improve clinical outcomes. Mechanistic analyses are underway to investigate the differential effect of fostamatinib on soluble mediators associated with immune dysregulation relative to placebo (Jeff Stritch, MD, MHS; written communication; August 15, 2024). While differences in dose and type of anticoagulant could have a theoretical impact on microthrombosis, similar proportions of patients in each arm were receiving anticoagulants. However, we did not assess whether this dosage was prophylactic or therapeutic. There could have been imbalances in the amount of anticoagulation, potentially impacting the degree of lung injury via microthrombosis. In addition, while SARS-CoV-2 variants can be contextualized by the year of the pandemic in different countries, this may be a poor surrogate for the variant at a participant level when comparing data across countries.

## Conclusions

In this randomized clinical trial of adults hospitalized with COVID-19 and hypoxemia, fostamatinib did not increase the number of oxygen-free days compared with placebo. Our results do not suggest that using fostamatinib to alter dysregulated immune responses improved outcomes for a population of participants hospitalized with hypoxemia during the Omicron era.

## Notes

Supplement 1.
Trial Protocol

Supplement 2. **eTable 1.** Enrolling Sites **eTable 2.** Eligibility Criteria **eTable 3.** Trial Outcomes **eTable 4.** Protocol-Specified Exempt Serious Events **eTable 5.** Reasons for Not Receiving Full 28 Doses of Fostamatinib **eTable 6.** Adverse Events in Fostamatinib Trial **eTable 7.** Frequency of Other Safety Outcomes and PSESEs **eFigure 1.** Timeline of Trials Enrolling as Part of the ACTIV-4 Host Tissue Platform **eFigure 2.** Assessment of the Proportional Odds Assumption Using Repeated Dichotomizations **eFigure 3.** Distribution of Oxygen-Free Days in the Fostamatinib Trial **eFigure 4.** Treatment Effect on Primary, Secondary, and Exploratory Outcomes

Supplement 3.
ACTIV-4 Host Tissue Investigators

Supplement 4.
Data Sharing Statement

## References

[1] Adjei S, Hong K, Molinari NM, . Mortality risk among patients hospitalized primarily for COVID-19 during the Omicron and Delta variant pandemic periods—United States, April 2020-June 2022. MMWR Morb Mortal Wkly Rep. 2022;71(37):1182-1189. doi:10.15585/mmwr.mm7137a4 36107788 PMC9484808

[2] Cooper N, Ghanima W, Hill QA, Nicolson PL, Markovtsov V, Kessler C. Recent advances in understanding spleen tyrosine kinase (SYK) in human biology and disease, with a focus on fostamatinib. Platelets. 2023;34(1):2131751. doi:10.1080/09537104.2022.2131751 36331249

[3] Strich JR, Ramos-Benitez MJ, Randazzo D, . Fostamatinib inhibits neutrophils extracellular traps induced by COVID-19 patient plasma: a potential therapeutic. J Infect Dis. 2021;223(6):981-984. doi:10.1093/infdis/jiaa789 33367731 PMC7799006

[4] Brinkmann V, Reichard U, Goosmann C, . Neutrophil extracellular traps kill bacteria. Science. 2004;303(5663):1532-1535. doi:10.1126/science.1092385 15001782

[5] Saffarzadeh M, Juenemann C, Queisser MA, . Neutrophil extracellular traps directly induce epithelial and endothelial cell death: a predominant role of histones. PLoS One. 2012;7(2):e32366. doi:10.1371/journal.pone.0032366 22389696 PMC3289648

[6] Hudock KM, Collins MS, Imbrogno MA, . Alpha-1 antitrypsin limits neutrophil extracellular trap disruption of airway epithelial barrier function. Front Immunol. 2023;13:1023553. doi:10.3389/fimmu.2022.1023553 36703990 PMC9872031

[7] Fuchs TA, Brill A, Duerschmied D, . Extracellular DNA traps promote thrombosis. Proc Natl Acad Sci U S A. 2010;107(36):15880-15885. doi:10.1073/pnas.1005743107 20798043 PMC2936604

[8] Looney MR. Platelets induce neutrophil extracellular traps in transfusion-related acute lung injury. Blood. 2014;124(21):SCI-18. doi:10.1182/blood.V124.21.SCI-18.SCI-18 PMC3386815 22684106

[9] Hoepel W, Chen HJ, Geyer CE, . High titers and low fucosylation of early human anti-SARS-CoV-2 IgG promote inflammation by alveolar macrophages. Sci Transl Med. 2021;13(596):eabf8654. doi:10.1126/scitranslmed.abf8654 33979301 PMC8158960

[10] Apostolidis SA, Sarkar A, Giannini HM, ; UPenn COVID Processing Unit. Signaling through FcγRIIA and the C5a-C5aR pathway mediate platelet hyperactivation in COVID-19. Front Immunol. 2022;13:834988. doi:10.3389/fimmu.2022.834988 35309299 PMC8928747

[11] Bye AP, Hoepel W, Mitchell JL, . Aberrant glycosylation of anti-SARS-CoV-2 spike IgG is a prothrombotic stimulus for platelets. Blood. 2021;138(16):1481-1489. doi:10.1182/blood.2021011871 34315173 PMC8321687

[12] Wigerblad G, Warner SA, Ramos-Benitez MJ, . Spleen tyrosine kinase inhibition restores myeloid homeostasis in COVID-19. Sci Adv. 2023;9(1):eade8272. doi:10.1126/sciadv.ade8272 36598976 PMC9812373

[13] Strich JR, Tian X, Samour M, . Fostamatinib for the treatment of hospitalized adults with coronavirus disease 2019: a randomized trial. Clin Infect Dis. 2022;75(1):e491-e498. doi:10.1093/cid/ciab732 34467402 PMC9890443

[14] Gotur DB, Malik A, Markovtsov V, Yan L, Dummer W, Mallat Z. 88. Fostamatinib for the treatment of hospitalized patients with COVID-19 who required oxygen supplementation: results of a phase 3 trial. Open Forum Infect Dis. 2023;10(suppl 2):ofad500.004. doi:10.1093/ofid/ofad500.004

[15] Rigel announces top-line results from FOCUS phase 3 clinical trial of fostamatinib in high risk hospitalized COVID-19 patients. News release. Rigel Pharmaceuticals Inc; November 1, 2022. Accessed July 5, 2023. https://www.rigel.com/investors/news-events/press-releases/detail/345/rigel-announces-top-line-results-from-focus-phase-3

[16] National Institutes of Health. COVID-19 therapeutics prioritized for testing in clinical trials. July 21, 2020. Accessed March 13, 2024. https://www.nih.gov/research-training/medical-research-initiatives/activ/covid-19-therapeutics-prioritized-testing-clinical-trials

[17] Self WH, Shotwell MS, Gibbs KW, ; ACTIV-4 Host Tissue Investigators. Renin-angiotensin system modulation with synthetic angiotensin (1-7) and angiotensin II type 1 receptor-biased ligand in adults with COVID-19: two randomized clinical trials. JAMA. 2023;329(14):1170-1182. doi:10.1001/jama.2023.3546 37039791 PMC10091180

[18] Moskowitz A, Shotwell MS, Gibbs KW, ; Fourth Accelerating COVID-19 Therapeutic Interventions and Vaccines (ACTIV-4) Host Tissue Investigators. Oxygen-free days as an outcome measure in clinical trials of therapies for COVID-19 and other causes of new-onset hypoxemia. Chest. 2022;162(4):804-814. doi:10.1016/j.chest.2022.04.145 35504307 PMC9055785

[19] EQUATOR Network. CONSORT 2010 statement: updated guidelines for reporting parallel group randomised trials. Accessed September 23, 2024. https://www.equator-network.org/reporting-guidelines/consort/

[20] Harris PA, Taylor R, Thielke R, Payne J, Gonzalez N, Conde JG. Research electronic data capture (REDCap)—a metadata-driven methodology and workflow process for providing translational research informatics support. J Biomed Inform. 2009;42(2):377-381. doi:10.1016/j.jbi.2008.08.010 18929686 PMC2700030

[21] Marshall JC, Murthy S, Diaz J, ; WHO Working Group on the Clinical Characterisation and Management of COVID-19 Infection. A minimal common outcome measure set for COVID-19 clinical research. Lancet Infect Dis. 2020;20(8):e192-e197. doi:10.1016/S1473-3099(20)30483-7 32539990 PMC7292605

[22] Sohn W, Jun DW, Kwak MJ, . Upper limit of normal serum alanine and aspartate aminotransferase levels in Korea. J Gastroenterol Hepatol. 2013;28(3):522-529. doi:10.1111/j.1440-1746.2012.07143.x 22497339

[23] Self WH, Wheeler AP, Stewart TG, ; Passive Immunity Trial for Our Nation (PassITON) Investigators. Neutralizing COVID-19 convalescent plasma in adults hospitalized with COVID-19: a blinded, randomized, placebo-controlled trial. Chest. 2022;162(5):982-994. doi:10.1016/j.chest.2022.06.029 35780813 PMC9247217

[24] Wiedemann HP, Wheeler AP, Bernard GR, ; National Heart, Lung, and Blood Institute Acute Respiratory Distress Syndrome (ARDS) Clinical Trials Network. Comparison of two fluid-management strategies in acute lung injury. N Engl J Med. 2006;354(24):2564-2575. doi:10.1056/NEJMoa062200 16714767

[25] Laterre PF, Berry SM, Blemings A, ; SEPSIS-ACT Investigators. Effect of selepressin vs placebo on ventilator- and vasopressor-free days in patients with septic shock: the SEPSIS-ACT randomized clinical trial. JAMA. 2019;322(15):1476-1485. doi:10.1001/jama.2019.14607 31577035 PMC6802260

[26] Self WH, Semler MW, Leither LM, ; National Heart, Lung, and Blood Institute PETAL Clinical Trials Network. Effect of hydroxychloroquine on clinical status at 14 days in hospitalized patients with COVID-19: a randomized clinical trial. JAMA. 2020;324(21):2165-2176. doi:10.1001/jama.2020.22240 33165621 PMC7653542

[27] Wang R, Lagakos SW, Ware JH, Hunter DJ, Drazen JM. Statistics in medicine—reporting of subgroup analyses in clinical trials. N Engl J Med. 2007;357(21):2189-2194. doi:10.1056/NEJMsr077003 18032770

[28] DeCuir J, Surie D, Zhu Y, ; IVY Network. Effectiveness of monovalent mRNA COVID-19 vaccination in preventing COVID-19-associated invasive mechanical ventilation and death among immunocompetent adults during the Omicron variant period—IVY Network, 19 US States, February 1, 2022–January 31, 2023. MMWR Morb Mortal Wkly Rep. 2023;72(17):463-468. doi:10.15585/mmwr.mm7217a3 37104244

[29] Tenforde MW, Self WH, Gaglani M, ; IVY Network. Effectiveness of mRNA vaccination in preventing COVID-19–associated invasive mechanical ventilation and death—United States, March 2021–January 2022. MMWR Morb Mortal Wkly Rep. 2022;71(12):459-465. doi:10.15585/mmwr.mm7112e1 35324878 PMC8956334

[30] Griggs EP, Mitchell PK, Lazariu V, . Clinical epidemiology and risk factors for critical outcomes among vaccinated and unvaccinated adults hospitalized with COVID-19-VISION network, 10 states, June 2021–March 2023. Clin Infect Dis. 2024;78(2):338-348. doi:10.1093/cid/ciad505 37633258 PMC11293024

[31] Kojima N, Adams K, Self WH, ; Investigating Respiratory Viruses in the Acutely Ill (IVY) Network. Changing severity and epidemiology of adults hospitalized with coronavirus disease 2019 (COVID-19) in the United States after introduction of COVID-19 vaccines, March 2021–August 2022. Clin Infect Dis. 2023;77(4):547-557. doi:10.1093/cid/ciad276 37255285 PMC10526883

[32] Martin TR, Zemans RL, Ware LB, . New insights into clinical and mechanistic heterogeneity of the acute respiratory distress syndrome: summary of the Aspen Lung Conference 2021. Am J Respir Cell Mol Biol. 2022;67(3):284-308. doi:10.1165/rcmb.2022-0089WS 35679511 PMC9447141

