# Supplementary material for: Fostamatinib for Hospitalized Adults With COVID-19 and Hypoxemia: A Randomized Clinical Trial
Source: JAMA Netw Open. 2024 Dec 3;7(12):e2448215. doi: 10.1001/jamanetworkopen.2024.48215 (PMC11615712; doi:10.1001/jamanetworkopen.2024.48215)
Supplement: Supplement 1. — Trial Protocol [file jamanetwopen-e2448215-s001.pdf]

CONNECTS Master Protocol for Clinical Trials targeting macro-, micro-immuno-thrombosis, vascular hyperinflammation, and hypercoagulability and renin-angiotensin-aldosterone system (RAAS) in hospitalized patients with COVID-19 (ACTIV-4 Host Tissue)

Short Title: **N**ovelst **E**xperimental **C**OVID **T**herapies **A**ffecting Host **R**esponse (**NECTAR)**

COVID-19 Inpatient Host Tissue Master Protocol

ClinicalTrials.gov Number: NCT04924660

Supported by: NHLBI-CONNECTS

Version Number: 4.0

2022.10.18

**Study Chair**<br><br>**Principal Investigator, Clinical Coordinating Center**	Sean P. Collins, MD, MSc<br>Email: sean.collins@vumc.org<br><br>Wesley H. Self, MD, MPH<br>Email: wesley.self@vumc.org
**Principal Investigator, Data Coordinating Center**	Matthew S. Shotwell, PhD<br>Email: matt.shotwell@vumc.org
**Trial Biostatisticians**	Christopher J. Lindsell, PhD<br>Email: chris.lindsell@vumc.org<br><br>James Troendle, PhD<br>NHLBI<br>Email: James.troendle@nih.gov
**Medical Monitor**	Matthew Semler, MD, MSc<br>Email: matthew.w.semler@vumc.org
**NHLBI Representative**	Yves Rosenberg, MD<br>Email: rosenbey@nhlbi.nih.gov
**Ex-U.S. Sponsor**	NEAT-ID
**IND**	154000
**ClinicalTrials.gov Identifier**	NCT04924660
**NECTAR EudraCT**	2022-000715-31
**sIRB study number**	210982

Table of Contents

1 List of Abbreviations vi
2 Master Protocol Summary 8
3 Introduction, Background Information and Scientific Rationale 11
3.1 Background Information, Significance and Relevant Literature 11
3.2 Relevance of host tissue pathway(s) to COVID-19 12
3.3 Rationale for evaluating host tissue therapies in a single platform among patients who are hospitalized with COVID-19 12
3.4 Potential Risks & Benefits 13
4 Study Objectives and Purpose 13
4.1 Study Objectives 13
4.2 Study Hypothesis 13
5 Study Design and Outcomes 13
5.1 Overall Study Design 13
5.2 Randomization 13
5.3 Study Outcomes 14
5.3.1 Primary Study Outcome 14
5.3.2 Secondary Outcomes 15
5.3.3 Exploratory Outcomes 15
5.3.4 Safety Outcomes (systematically collected during index hospitalization) 16
6 Study population and enrollment 16
6.1 Inclusion criteria 16
6.2 Exclusion criteria 16
6.3 Justification of exclusion criteria 17
6.4 Special screening procedures 17
6.5 Assessment of eligibility and exclusion tracking 17
6.6 Process of obtaining informed consent 17
6.6.1 Paper-based approach 18
6.6.2 Electronic/e-consent approach for US sites only 18
6.6.3 Attestation of informed consent 19
6.7 Randomization and blinding 20
6.7.1 Emergency Unblinding 20
6.8 Vulnerable Subjects 21
6.9 Strategies for Recruitment and Retention 21
6.10 Duration of Study Participation 21
6.11 Participant Withdrawal or Termination 21
6.11.1 Reasons for Withdrawal or Termination 21
6.11.2 Premature Termination or Suspension 22
7 Study Procedures and Schedule 22
7.1 Study interventions 22
7.2 Expedited Critical and Major Event Reporting 23
7.3 Data and Safety Monitoring Plan 23

7.4 Biological specimens 23
7.5 Shared placebo group dose, duration, and route of administration....23
7.6 Co-Interventions and Co-enrollment 23
7.7 On study monitoring....24
7.7.1 Laboratory evaluations....24
7.7.2 Clinical evaluations 24
7.7.3 Criteria for stopping drug 25
7.7.4 Plan for drug shortages....25
7.7.5 Baseline variable collection....25
7.7.6 Assessments between hospital presentation and hospital discharge 26
7.7.7 Assessments following hospital discharge 27
8 Statistical Considerations....29
8.1 Statistical and Analytical Plans....29
8.2 Analysis Datasets 29
8.3 Statistical Modeling....29
8.4 Loss to Follow-up, Censoring, and Intercurrent Events 30
8.5 Model Prior and Bayesian Computation 30
8.6 Analysis of Primary Outcome....30
8.6.1 Primary Analysis 30
8.6.2 Planned Interim and Final Analyses, Early Stopping, and Type-I Error Control 31
8.6.3 Supplementary Efficacy Estimands....31
8.6.4 Sensitivity and Supplementary Analyses....31
8.6.5 Sample Size and Decision Thresholds....33
8.7 Analysis of Secondary Outcomes 35
8.8 Analysis of Safety Outcomes 36
8.9 Adherence and Retention Analyses 36
8.10 Baseline Descriptive Statistics 36
8.11 Exploratory Analyses 36
9 Measures to Minimize Bias 36
9.1 Enrollment/Randomization/Blinding 36
10 Source Documents and Access to Source Data/Documents....36
11 Quality Assurance and Quality Control....37
12 Ethics/Protection of Human Subjects 37
12.1 Ethical Standard 37
12.2 IRB/Ethics Committee/Competent Authority....37
12.3 Posting of Clinical Trial Consent Form 37
12.4 Participant and Data Confidentiality 37
12.5 Certificate of Confidentiality 38
13 Adverse events 38
13.1 Defining adverse events 40
13.2 Protocol-specified exempt serious events (PSESEs) 41
13.3 Monitoring and recording adverse events 43
13.4 Reporting adverse events 44
13.4.1 Adverse Events that Qualify for Expedited Reporting....44

13.4.2 Grade 3 and 4 Clinical Adverse Events 45
13.4.3 Pregnancy 45

13.5 Medical Monitor 47

14 Risk Assessment 47

14.1 Potential risks of other study procedures 47
14.2 Minimization of risk 47
14.3 Potential benefit 48
14.4 Risk in relation to anticipated benefit 48

15 Data and Safety Monitoring Board (DSMB) 48
16 Data Handling and Record Keeping 48

16.1 Data Collection and Management Responsibilities 48
16.2 Study Records Retention 49
16.3 Protocol Deviations 49
16.4 21 CFR Part 11 Compliance 49

16.4.1 Record locking 49
16.4.2 eConsent 49

17 End of Trial 50
18 Study Finances 50

18.1 Funding Source 50
18.2 Costs to the Participant 50

19 Appendix A: Primary study outcomes 51

19.1 Approach to ascertainment and verification of outcomes 51
19.2 Primary outcome: Oxygen free days 51
19.3 Definitions 53

19.3.1 ICU Level of care 53
19.3.2 Myocardial injury 53
19.3.3 Acute Kidney Injury 53
19.3.4 Disseminated Intravascular Coagulation (DIC) (Overt) – DIC score ≥ 5 53
19.3.5 ISTH Defined Major Bleeding 54

20 Appendix B: Data and Safety Monitoring Plan 55

20.1 Overview 55

21 Appendix C: Minimum Biological Specimen collection 56
22 Appendix D: Arm 3: Fostamatinib 57

22.1 Description of active therapy 57
22.2 Rationale for evaluating Fostamatinib 57
22.3 Fostamatinib dose, duration, and route of administration 58
22.4 Placebo 58
22.5 Fostamatinib-specific safety considerations and potential medication interactions 58
22.6 Fostamatinib Arm-Specific Exclusion Criteria 59
22.7 Dose Modification Considerations and Medication Interactions 60
22.8 Fostamatinib Arm Logistics 64

23 Appendix E. Non-NAT Tests Deemed with Equivalent Specificity to NAT by the Protocol Team 66

24 References 67

List of Tables

Table 1. **Schedule of Events**..28
Table 2. **Overview of Safety Data Collection** 39
Table 3. **WHO COVID-19 Clinical Status Scale and its use for enrollment eligibility and calculation of oxygen free days (OFD)**..52
Table 4. **Descriptions of OFD Data**..53

List of Figures

Figure 1. **Adverse Event and Clinical Outcome Assessment, Recording, and Reporting** 46
Figure 2. **Schematic overview of blood pressure considerations and procedures performed for patients still on study drug at the time of hospital discharge** 60

1 List of Abbreviations

AE	Adverse Event/Adverse Experience
AESI	Adverse Event of Special Interest
ARDS	Acute Respiratory Distress Syndrome
CA	Competent Authority
CFR	Code of Federal Regulations
CHF	Congestive Heart Failure
CrCl	Creatinine Clearance
COVID-19	Coronavirus Disease
CRA	Clinical Research Associate
CRF	Case Report Form
CSOC	Clinical Study Oversight Committee
DCC	Data Coordinating Center
DHHS	Department of Health and Human Services
DIC	Disseminated Intravascular Coagulation
DSMB	Data and Safety Monitoring Board
ECMO	Extracorporeal Membrane Oxygenation
eGFR	Estimated Glomerular Filtration Rate
ESRD	End-stage renal disease
FDA	Food and Drug Administration
FFR	Federal Financial Report
FWA	Federal Wide Assurance
GCP	Good Clinical Practice
GDPR	General Data Protection Regulations
GI	Gastrointestinal
HFNO	High-flow Nasal Oxygen (≥30L/min)
HIPAA	Health Insurance Portability and Accountability Act
ICF	Informed Consent Form
ICH	International Conference on Harmonization
ICMJE	International Committee of Medical Journal Editors
IEC	Independent Ethics Committee
IRB	Institutional Review Board
ISM	Independent Safety Monitor
ISTH	International Society on Thrombosis and Hemostasis
ITT	Intent to Treat
LAR	Legally Authorized Representative

LOS	Length of Stay
MOP	Manual of Procedures
N	Number (typically refers to participants)
NETs	Neutrophil extracellular traps
NIH	National Institutes of Health
NIV	Non-Invasive Ventilation
NYULH	New York University Langone Health
OFD	Oxygen Free Days
OHRP	Office for Human Research Protections
OHSR	Office of Human Participants Research
OSFD	Organ Support Free Days
PI	Principal Investigator
PRBC	Packed Red Blood Cells
PSESEs	Protocol-specified exempt serious events
PTT	Partial Thromboplastin Time
QA	Quality Assurance
QC	Quality Control
RRT	Renal Replacement Therapy
SARS-CoV-2	Severe Acute Respiratory Syndrome- Coronavirus- 2
SAE	Serious Adverse Event/Serious Adverse Experience
SUSAR	Suspected Unexpected Serious Adverse Reactions
SOP	Standard Operating Procedure
US	United States
VTE	Venous Thromboembolism
WHO	World Health Organization
WOCBP	Women of Childbearing Potential

2 Master Protocol Summary

Title	Master Protocol for Clinical Trials targeting host tissue and the renin-angiotensin-aldosterone system (RAAS) in hospitalized patients with COVID-19
Short Title	Novel Experimental COVID Therapies Affecting Host Response (NECTAR)
Brief Summary	This Master Protocol describes the general design features of a platform trial evaluating therapies targeting the host response to COVID-19 in hospitalized patients. The Master Protocol provides the background and overarching approach to all trials to be conducted on this platform. This includes a rationale for the choice of primary outcome, inclusion and exclusion criteria, randomization and blinding, interim and final analyses, sample size considerations, safety reporting, and data collection. In addition, the Master Protocol describes general principles for trial operations and oversight. Appendices to the Master Protocol provide agent-specific details, including treatment dose, route and frequency, safety information, and any agent-specific considerations related to drug starting/stopping, inclusion and exclusion criteria and blinding procedures.
Objectives	The overarching goal of the Master Protocol is to find effective strategies for inpatient management of patients with COVID-19. Therapeutic goals for patients hospitalized for COVID-19 include hastening recovery and preventing progression to critical illness, multiorgan failure, or death. Our objective is to determine whether modulating the host tissue response improves clinical outcomes among patients with COVID-19.<br><br>Potential agent(s) to investigate on this platform include, but are not limited to, TXA127, TRV027, and Fostamatinib. These agents all impact the host tissue response in COVID-19 via a number of unique mechanisms including potential beneficial effects on the RAAS system and formation of neutrophil extracellular traps (NETs). We will evaluate the efficacy of these agents' ability to impact the host tissue response and improve outcomes in patients hospitalized with COVID-19.
Methodology	This platform will be a randomized, placebo-controlled trial of agents targeting the host response in COVID-19 in hospitalized patients. The Master Protocol is designed to be flexible in the number of study arms, the use of a single placebo group, and the stopping and adding of new therapies.

<table>
<tr>
<td>Outcomes</td>
<td>
Primary Outcome:
Oxygen free days through day 28. This is defined as days alive and without supplemental oxygen use during the first 28 days following randomization. Patients who die prior to day 28 are assigned -1 oxygen free days.

Secondary outcomes:

In hospital mortality
Proportion of patients alive and oxygen free at days 14 and 28
Proportion of patients with new invasive mechanical ventilation at day 28
28-day mortality
60-day mortality
90-day mortality
WHO 8-point ordinal scale at 14, 28, and 60 days

1: Ambulatory – Not hospitalized and no limitation of activities
2: Ambulatory – Not hospitalized with limitation of activities or home oxygen use
3: Hospitalized Mild Disease – Hospitalized, no oxygen therapy
4: Hospitalized Mild Disease – Hospitalized, oxygen by mask or nasal prongs
5: Hospitalized Severe Disease – Non-invasive ventilation or high-flow nasal cannula
6: Hospitalized Severe Disease –Invasive mechanical ventilation
7: Hospitalized Severe Disease – Invasive mechanical ventilation plus additional organ support with- vasopressors, RRT, or ECMO
8: Dead

Support-free days through Day 28, including:

Hospital-free days
Ventilator-free days
Respiratory failure free days

Exploratory Outcomes

Renal outcomes: acute kidney Injury defined as ≥ KDIGO Stage 2 and changes in serum creatinine and estimated Glomerular Filtration Rate
Myocardial injury as measured by changes in troponin before, during and after therapy during hospitalization (when possible, at participating U.S. sites)
RAAS pathway mechanistic biomarkers (AngII, Ang(1-7), ACE and ACE2) (when possible and applicable, at participating U.S. sites)
Trajectories of biomarkers related to COVID-19 (when possible, at participating U.S. sites)
Changes in NT-proBNP before, during and after therapy during hospitalization (when possible, at participating U.S. sites)

</td>
</tr>
</table>

	**Safety outcomes (systematically collected during index hospitalization):**<br>• Hypotension as defined by low arterial blood pressure leading to either [1] initiation or increase in vasopressor therapy, [2] administration of a fluid bolus of 500 ml or more, or [3] modification of the dose or discontinuation of the study drug.<br>• Allergic reaction, including angioedema and rash<br>• Incident renal replacement therapy during hospitalization
Study Duration	Multiple arms can actively enroll concurrently for an anticipated 15 months.
Duration of Participant follow-up	Duration of hospitalization with post-discharge follow-up for up to 90 days after randomization. (Specific eligibility criteria are in the main protocol text)
Population	Patients hospitalized for COVID-19 with laboratory confirmed SARS-CoV-2 infection on oxygen therapy.
Study Sites	Sites affiliated with NHLBI-CONNECTS Network of Networks and other networks and sites with previous clinical trial experience. Selected sites will be sufficiently equipped and experienced to safely enroll and follow patients, and to produce accurate data. The number of enrolling sites will be informed by the number of hospitalized patients with COVID-19 at active sites, the sample size required, and projected patient accrual.
Planned Maximum Number of Subjects	We expect the maximum sample size to be about 300 per interventional treatment arm.<br><br>Prior to the first interim analysis, sample size adequacy was re-assessed based on the pooled (across all active and placebo arms) distribution of the primary outcome. No adjustments to the maximum sample size were made. Placebo enrollment beyond 300 participants may be required to ensure there are sufficient concurrently randomized and eligibility-matched placebo participants for comparison with each active drug arm.
Description of Study Agents	Specific agents will be described within the agent-specific appendices. The therapies relevant to this Master Protocol must have some mechanistic link to preventing progression to critical illness, multiorgan failure, or death in patients with COVID-19 and related to the host tissue response to COVID-19.
Key Procedures	Participants will be recruited in the inpatient setting. They will undergo baseline evaluations for eligibility. They will then be randomized, stratified by site, and study intervention will begin. Baseline laboratories will be required, and biobanking will occur both at randomization, and during the study period. The primary outcome will be assessed daily via chart review during hospitalization. Patients will undergo additional data collection by telephone, mail, in-person visits or electronic (e.g., email, text message) surveys after discharge.

Statistical Analysis	The effect of each study agent on the primary outcome, versus matching placebo, will be quantified using the odds ratio to evaluate the odds of greater oxygen free days at day 28 (i.e., the primary estimand). Estimation and inferences about the primary estimand will be made using proportional odds logistic regression methods. For each study agent, the comparison group will consist of all concurrently randomized placebo participants meeting the inclusion and exclusion criteria for that agent.<br><br>For each arm, we will use pre-planned interim analyses at fixed recruitment intervals to consider ending enrollment early due to strong evidence of inferiority. Early stopping and final analysis thresholds will be selected to ensure a type-I error probability of 2.5% (one-sided), separately for each study agent.<br><br>We will use a modified intention to treat (mITT) approach for primary analyses. All available data on participants who were eligible, randomized, and received at least some study drug will be used to compare each treatment versus control, regardless of post-randomization adherence to study protocols. The intercurrent event of death will be coded as a special value in the primary outcome (i.e., composite strategy). Censoring in the primary outcome will be modeled using likelihood methods. No other intercurrent events will affect the primary outcome (i.e., treatment policy strategy). We will monitor closely for patients who are randomized who do not receive study drug to ensure our preplanned sample size targets for the mITT group are met.

3 Introduction, Background Information and Scientific Rationale

3.1 Background Information, Significance and Relevant Literature

The severe acute respiratory syndrome coronavirus 2 (SARS-CoV-2), which causes coronavirus disease 2019 (COVID-19), has resulted in a global pandemic. The clinical spectrum of COVID-19 infection is broad, encompassing asymptomatic infection, mild upper respiratory tract illness, and severe viral pneumonia with respiratory failure and death. Between 13 and 40% of patients become hospitalized,[1,2] up to 30% of those hospitalized require admission for intensive care, and there is a 13% inpatient mortality rate.[3,4] The reasons for hospitalization include respiratory support, as well as support for failure of other organs, including the heart and kidneys. The risk of thrombotic complications is increased, even when compared to other viral respiratory illnesses, such as influenza.[5] While 82% of hospitalized patients with COVID-19 are ultimately discharged alive,[6] median length of stay is 10-13 days.[7] Clinical trials in COVID-19 inpatients are needed to find better strategies to prevent or treat progression to critical illness, multiorgan failure, or death.

Early work in treating COVID-19 has focused on preventing worsening of the initial clinical presentation to prevent hospitalization and disease progression to organ failure and death. Studies conducted under this Master Protocol are expected to extend our knowledge of how to manage patients who are hospitalized for COVID-19 illness.

This protocol intends to define effective therapeutic regimens in a randomized trial of patients hospitalized with COVID-19. The primary outcome is oxygen free days through day 28.

3.2 Relevance of host tissue pathway(s) to COVID-19

Most adults with SARS-CoV-2 infection recover after a brief illness with fever, cough, and fatigue or similar symptoms. Current therapies are limited in the subset of patients who progress to hypoxemic respiratory failure and acute respiratory distress syndrome (ARDS).[8–10] The SARS-CoV-2 virus enters pulmonary and myocardial cells by the binding of the spike viral protein to the Angiotensin-Converting Enzyme 2 (ACE2) receptor, a key actuator in the renin-angiotensin-aldosterone system (RAAS). Thus, in COVID-19, RAAS has been directly implicated in the pathogenesis of ARDS as part of the host tissue response. ACE2 catalyzes the conversion of Angiotensin II (AngII) to Ang(1-7). When ACE2 is not present AngII remains at increased levels stimulating vasoconstriction, the production of inflammatory cytokines, and pulmonary fibrosis.[11] Even before COVID-19, ACE2 was found to be protective in preclinical models of acute lung injury and ARDS. Mice deficient in ACE2 develop acute lung injury following a challenge with a variety of insults,[12,13] which improves on repletion with recombinant ACE2.[14]

3.3 Rationale for evaluating host tissue therapies in a single platform among patients who are hospitalized with COVID-19

The importance of the host tissue response is important to consider in patients hospitalized with COVID-19. ACE/Ang II signaling in human disease is suggested by increased levels of ACE and Ang II in ARDS and sepsis patients.[15–18] Patients with the D allele for the ACE gene have higher ACE and Ang II levels in tissue and serum[19] and these patients are at higher risk of death from ARDS in multiple large cohorts.[19–21] Restoration of ACE2 through the administration of recombinant ACE2 in a phase II trial of ARDS in humans (n=44) appeared to safely reduce AngII levels and increase Ang(1-7) levels without causing significant hemodynamic changes.[22] Further, up to 20% of patients with COVID-19 develop myocardial injury, which has been independently associated with increased arrhythmias, shock and mortality.[23–25] ACE2 receptors are present in cardiac myocytes and fibroblasts and the endothelium of coronary arteries, and the ACE2 receptor has been implicated as a potential mediator of cardiac injury in COVID-19.[26] Thrombotic events are a known complication in patients hospitalized with COVID-19. In vitro evidence suggests, R406, the active component of fostamatinib, can inhibit the Fc-mediated release of proinflammatory cytokines by macrophages and platelet-mediated thrombosis provoked by SARS-CoV-2 specific spike antigen/antibody complexes. Furthermore, R406 also has been shown to inhibit the release of neutrophil extracellular traps from neutrophils stimulated with plasma from patients with COVID-19. Taken together, it is hypothesized that fostamatinib will decrease the inflammatory milieu generated through Fc-activation, ultimately resulting in decreasing immunothrombosis. Preventing immunothrombosis in the pulmonary vasculature could mitigate lung injury and hasten recovery from COVID-19.

Recent large-scale cohort studies, however, have not found an association between current use of RAAS inhibitors and either increased risk of contracting COVID-19 infection or increased risk of severe disease from COVID-19.[27,28] Two randomized trials in patients hospitalized with COVID-19 who were already taking RAAS inhibitors found no benefit of stopping RAAS inhibitors when compared to continuing them (BRACE CORONA, REPLACE COVID).[29–31] Thus, mechanistic pathophysiology and preliminary data in ARDS provide a compelling rationale for studying the effect of agents targeting the RAAS system using a RAAS platform as we propose.

There is strong a rationale for considering multiple host tissue agents on this platform due to complementary but distinct mechanisms of action. TRV027 and Ang (1-7) both work to restore AngII balance by working downstream of the ACE2 receptor via different mechanisms of improving the Ang(1-7) to AngII ratio. Recent data from two Phase II trials suggested that fostamatinib may have substantial impact on outcomes in patients hospitalized with COVID-19, providing compelling

rationale for including this agent on our platform. A recent placebo-controlled randomized phase 2 study in hospitalized adults with Covid-19 (NCT04579393) suggested fostamatinib in addition to usual care was safe and did not result in more serious adverse events (10.5% in the fostamatinib group vs. 22% in the placebo group). Additionally, multiple secondary efficacy endpoints showed trends favoring the patients receiving fostamatinib, including 28-day mortality, days free of oxygen, and recovery as measured on the 8-point ordinal scale at day 15.

3.4Potential Risks & Benefits

Participating in this Master Protocol includes risks related to the treatment, as well as risks related to privacy and confidentiality. Benefits include the potential for benefit of the therapeutic strategy, increased attention to the participant's treatments and clinical course when compared with usual care, and the global societal benefit of contributing knowledge about COVID-19 treatments and pathophysiology. See sections 12 and 13 and agent-specific Appendices for details.

4 Study Objectives and Purpose

The overarching objective of this platform is to iteratively test treatment strategies targeting the host tissue response for improving clinical outcomes among adults hospitalized with COVID-19. Treatment strategies will be added to the current best practice and tested against best practice plus placebo. Best practice may itself be updated as therapies become available or are shown to be effective (or ineffective).

4.1 Study Objectives

Our objective is to determine the impact of modulating the host tissue response, including counterbalancing RAAS activity, on mortality and outcomes related to ARDS. A further objective is to determine which of the different RAAS agents' targets (AT1r biased agonist, Ang[1-7] infusion) and associated mechanisms of action, when added to current best practice and compared to current best practice plus placebo, result in an effective therapeutic approach to the RAAS system in patients infected with SARS-CoV-2.

4.2 Study Hypothesis

We hypothesize the administration of Ang (1-7), TRV027, or fostamatinib (all dosed individually, no combinations) will improve clinical outcomes and will result in improvement in oxygen-free days through day 28.

5 Study Design and Outcomes

5.1 Overall Study Design

This Master Protocol describes an overarching approach to studies of blinded, placebo-controlled therapeutic approaches of host-tissue targeted therapies in hospitalized COVID-19 patients. The Master Protocol is designed so the platform can be flexible in the number of study arms, the use of a single placebo group, and the stopping and adding of new therapies, while using a common approach to design and implementation.

5.2 Randomization

Randomization assignments are at the participant level and treatments are assigned at randomization. Randomization will be implemented using permuted blocks and stratified by site and eligibility group. Stratification ensures balance across the active and pooled placebo groups at

regular enrollment intervals within each stratum, thus mitigating the impact of stratum (i.e., site) heterogeneity on assessments of treatment effect. Allocation will be equally distributed across arms for which the participant is eligible.

5.3 Study Outcomes

5.3.1 Primary Study Outcome

The primary outcome for this platform is oxygen free days (OFD) at day 28. It is designed to assess lung function as determined by freedom from oxygen therapy for the first 28 days following randomization. This is an important patient-centric outcome reflective of recovery from SARS-CoV-2 infection. Additional rationale for the primary outcome is explained in detail in Appendix A: Primary study outcomes. OFD is a clinically relevant, longitudinal measure of lung function and mortality assessed at 28 days after randomization. Liberation from oxygen is an important patient-centric outcome and freedom from oxygen dependency is a primary goal for patients during both hospitalization and the early post-discharge period. OFD will be calculated using principles developed during the past 20 years for other free-day clinical trial outcomes, including ventilator free days,[32,33] organ support free days,[34] and hospital free days.[35] The concept of time to liberation from oxygen therapy, and the related outcome of time to recovery, has been extensively used in COVID-19 trials evaluating in-hospital therapies.[36,37] For example, the primary outcome for the first trial on the Adaptive COVID-19 Treatment Trial platform (ACTT-1)[38] was time to recovery during the first 28 days after randomization, defined as time between randomization and the earlier of hospital discharge or discontinuation of oxygen therapy and other in-hospital therapies for COVID-19. Oxygen free days was selected over time to recovery, as defined in ACTT-1, as the primary outcome for our proposal for two reasons: (1) to capture home oxygen use as part of the primary outcome; and (2) to incorporate the competing risk of death into the primary outcome using the same methodology commonly used for other outcomes evaluating duration of organ support, such as ventilator free days.

OFD will be calculated as the number of calendar days during the first 28 days after randomization during which the patient was alive and not receiving new supplemental oxygen therapy.[39] Patients will be considered to be receiving supplemental oxygen therapy when they are receiving any of the following: supplemental oxygen by nasal cannula, supplemental oxygen by face mask, high flow nasal cannula (HFNC), non-invasive ventilation (NIV), invasive mechanical ventilation (IMV), or extracorporeal membrane oxygenation (ECMO). The day of randomization is denoted as Day 0. Starting with calendar day 1 (the day after randomization) and continuing for 28 days, study personnel will document whether the patient received oxygen therapy on each day for any duration of time. While the patient is in the hospital, the highest level of respiratory support received during each calendar day will be documented according to the 8-category WHO COVID-19 clinical status scale. Categories 4, 5, 6, and 7 indicate in-hospital oxygen use.

Use of supplemental oxygen at home after discharge will be assessed via telephone follow-up calls and text/email responses to the participant or surrogates. Patients who chronically used supplemental oxygen prior to their COVID-19 illness will be considered oxygen free when they return to the same level of oxygen support, they had been using prior to COVID-19 illness. For example, a patient who chronically used supplemental oxygen at 4 liters per minute via nasal cannula before COVID-19 and who was intubated for acute management of COVID-19 would be considered oxygen free for calculation of the primary outcome when he/she returned to oxygen support via nasal cannula at 4 liters per minute or less.

The primary outcome, OFD, will be calculated as 28 minus the number of days with oxygen use during the first 28 days after randomization. OFD will be coded as -1 for patients who died before

study day 28. Hence, the range for OFD is from -1 to 28 days. The first day of follow-up is the day after randomization, so 28 OFDs are the maximum possible days (Appendix A: Primary study outcomes).

5.3.2 Secondary Outcomes

- Alive and oxygen free at days 14 and 28
- Alive and respiratory failure-free at days 14 and 28
- Alive and free of new invasive mechanical ventilation at 14 and 28 days
- In-hospital, 28-day, 60-day and 90-day mortality
- WHO 8-point ordinal scale at 14, 28 and 60 days
    - 1: Ambulatory – Not hospitalized, no limitation of activities
    - 2: Ambulatory – Not hospitalized with limitation of activities or home oxygen therapy
    - 3: Hospitalized Mild Disease – Hospitalized, no oxygen therapy
    - 4: Hospitalized Mild Disease – Oxygen by mask or nasal prongs
    - 5: Hospitalized Severe Disease – Non-invasive ventilation of high-flowa oxygen
    - 6: Hospitalized Severe Disease –Invasive mechanical ventilation
    - 7: Hospitalized Severe Disease – Invasive mechanical ventilation plus additional organ support with-vasopressors, RRT, or ECMO
    - 8: Dead
- Support-free days to Day 28, including:
    - Hospital-free days
    - Respiratory failure-free days
    - Ventilator-free days

Alive and respiratory failure-free at day 28, the WHO 8-point ordinal scale at day 28, and mortality at day 28 are key secondary outcomes that will be treated as a family for testing purposes, even though the studies will not be adequately powered to detect anything but a very strong treatment effect on these outcomes. A supplementary analysis to assess the evidence that treatment lowers the risk of death in a way that is consistent with its effect on nonfatal outcomes will be performed. A respiratory failure-free day is defined as a day alive without the use of HFNC, NIV, IMV, or ECMO. Participants that are alive but not hospitalized are considered free of respiratory failure.

5.3.3 Exploratory Outcomes

Exploratory outcomes will include the following (further defined in Appendix C: Minimum Biological Specimen collection):

- Myocardial injury described by changes in troponin before, during and after therapy during hospitalization (when possible, at participating U.S. sites).
- Myocardial function described by changes in NT-proBNP before, during and after therapy during hospitalization (when possible, at participating U.S. sites).
- RAAS mechanistic biomarkers (AngII, Ang(1-7), ACE and ACE2) before, during and after therapy during hospitalization (measured in samples from a subset of participants, when possible *and applicable*, at participating U.S. sites).
- Renal outcomes: acute kidney injury (following KDIGO) defined as $\geq$ KDIGO Stage 2 and changes in serum creatinine and estimated Filtration Rate during hospitalization
- Trajectories of biomarkers related to COVID-19 during hospitalization (when possible, at participating U.S. sites).

Exploratory outcomes may be collected at just a subset of sites.

5.3.4 Safety Outcomes (systematically collected during index hospitalization)

Safety outcomes will be measured to reflect the expected adverse consequences of therapeutic strategies.

- Hypotension as defined by low arterial blood pressure leading to either [1] initiation or increase in vasopressor therapy, [2] administration of a fluid bolus of 500 ml or more, or [3] modification of the dose or discontinuation of the study drug.
- Allergic reaction, including rash and angioedema
- Incident renal replacement therapy during hospitalization

6 Study population and enrollment

A broad population of adults hospitalized with COVID-19 will be enrolled on this platform without exclusions based on age, sex, race, ethnicity, severity of disease or preferred language. Exclusion criteria are related to safety. Eligibility criteria must be fulfilled at the time of randomization.

6.1 Inclusion criteria

1. Hospitalized for COVID-19
2. ≥18 years of age
3. SARS-CoV-2 infection, documented by:
   a) a nucleic acid test (NAT) or equivalent testing within 3 days prior to randomization OR
   b) documented by NAT or equivalent testing more than 3 days prior to randomization AND progressive disease suggestive of ongoing SARS-CoV-2 infection per the responsible investigator (For non-NAT tests, only those deemed with equivalent specificity to NAT by the protocol team will be allowed. A central list of allowed non-NAT tests is maintained in Appendix E. Non-NAT Tests Deemed with Equivalent Specificity to NAT by the Protocol Team).
4. Hypoxemia, defined as SpO2 <92% on room air, new receipt of supplemental oxygen to maintain SpO2 ≥92%, or increased supplemental oxygen to maintain SpO2 ≥92% for a patient on chronic oxygen therapy
5. Symptoms or signs of acute COVID-19, defined as one or more of the following:
   a) cough
   b) reported or documented body temperature of 100.4^{o} F or greater
   c) shortness of breath
   d) chest pain
   e) infiltrates on chest imaging (x-ray, CT scan, lung ultrasound)

6.2 Exclusion criteria

1. Onset of COVID-19 symptoms fulfilling inclusion criterion #5 >14 days prior to randomization
2. Hospitalized with hypoxemia (as defined in inclusion #4) for >72 hours prior to randomization (the 72-hour window for randomization begins when the patient first meets the hypoxemia inclusion criteria after hospital admission)
3. Pregnancy

4. Breastfeeding
5. Prisoners
6. End-stage renal disease (ESRD) on dialysis
7. Patient undergoing comfort care measures only such that treatment focuses on end-of-life symptom management over prolongation of life.
8. The treating clinician expects inability to participate in study procedures or participation would not be in the best interests of the patient
9. Known allergy/hypersensitivity to IMP or its excipients

6.3 Justification of exclusion criteria

The study medications' impact on breastfeeding and breastmilk is unknown, and we therefore exclude breastfeeding. We aim to study the impact of early interventions in the hospitalized setting and thus exclude those people who have prolonged symptoms or have been hospitalized greater than 72 hours prior to randomization. A patient is considered to have been hospitalized for > 72 hours prior to randomization if the presentation that initiated the current inpatient admission began more than 72 hours before randomization, regardless of the initial location of care (e.g., current hospital or transferring hospital) or level of care (e.g., emergency department, hospital ward, intensive care unit).

6.4 Special screening procedures

The site investigator or delegate will screen for hospitalized patients with laboratory confirmed COVID-19 (that is, a positive laboratory test for SARS-CoV-2) or a pending SARS-CoV-2 test. Treating clinicians will also be instructed to contact the site investigator or delegate for patients with a high clinical suspicion of COVID-19 prior to confirmatory testing.

6.5 Assessment of eligibility and exclusion tracking

For patients who appear to meet inclusion criteria during pre-screening, an electronic pre-screening form will be completed to determine eligibility and track exclusions. The electronic pre-screening form will be accessed and stored in the electronic pre-screening database. At the time of entry into the pre-screening database, the patient will be assigned a pre-screening number.

If a patient appears to meet all eligibility criteria, the site investigator or delegate will approach the treating clinician to ask permission to approach the patient or Legally Authorized Representative (LAR) to confirm eligibility, discuss potential study recruitment, and proceed with informed consent.

For excluded patients located in the United States, including refusal by the treating clinician or patient/surrogate, a small number of de-identified pre-screening variables will be collected including month and year the patient met pre-screening criteria, age, sex, ethnicity, and reason(s) the patient was excluded. For patients located outside of the United States, no personal information will be collected prior to consent, nor stored in the pre-screening database. For the safety of research personnel and conservation of personal protective equipment (PPE), these encounters may occur via telephone or videophone.

6.6 Process of obtaining informed consent

Informed consent is a process initiated prior to the individual's agreeing to participate in the study and continues throughout the individual's study participation. Informed consent should follow both local institutional policy and national regulatory guidance including following institutional COVID policy to protect study staff. Informed consent will be obtained from the patient or from a surrogate decision maker if the patient lacks decision-making capacity.

In some instances, bringing a paper consent form and pen to the bedside of a patient with known or suspected COVID-19 and then taking these out of the room would violate infection control principles and policies. Given the infectious risk from COVID-19 and potential shortages of PPE, there is a moral and practical imperative to minimize face-to-face contact between patients and non-clinical personnel. The current pandemic also presents unique challenges to obtaining consent from a participant's LAR. To minimize infectious risk, many institutions are not allowing visitors to enter the hospital. Furthermore, the LAR is likely to have been exposed to the patient and may therefore be under self-quarantine at the time of the informed consent discussion.

Therefore, in addition to the traditional approach of an in-person consent discussion and signed paper informed consent document, we will allow use of "no-touch" consent procedures for this trial. Below, we outline three examples of no-touch consent procedures that may be used: (a) a paper-based approach; (b) an electronic/e-consent approach; and (c) attestation of informed consent.

6.6.1 Paper-based approach

1. The informed consent document is delivered to the patient or LAR.
    a. If the patient or LAR is on-site, the informed consent document may be delivered to the patient or LAR either by research staff or by clinical staff.
    b. If the LAR is off-site, the informed consent document may be emailed, faxed, or otherwise electronically transferred to the LAR (method dictated by institutional policy).
2. Research staff discuss the informed consent document with the patient or LAR either in-person or by telephone or videophone. This step confirms subject/LAR identity.
3. If the patient or LAR decides to consent to participate, the patient or LAR signs the paper copy of the informed consent document.
4. A photograph is taken of the signature page by the patient or LAR (or research staff if onsite with patient/LAR) of the informed consent document and uploaded into the electronic database (e.g., REDCap). Non-US sites will upload consent images into a local database.
    a. If using the patient's device (such as a patient's personal cellular phone), a survey link can be sent to their device to allow direct upload of the image into the electronic database (e.g., REDCap).
    b. If using a staff device, it must be approved to store PHI by the local institution. In that case, research personnel can take a photograph of the signature page of the informed consent document either directly or through the window or glass door leading into the patient's room. The photograph can then be uploaded into the electronic database. If a staff device is taken into the patient's room to take a photograph it must be able to be disinfected according to local institutional practices.
5. Research staff and the witness provide signatures within the electronic database (e.g., REDCap) confirming their participation in the informed consent process.
6. The patient or LAR retains the paper consent document. The image of the signature page may be printed and bundled with a copy of the blank informed consent document for research records.

6.6.2 Electronic/e-consent approach for US sites only

1. The electronic informed consent document is opened on a research device or a link for the electronic informed consent document is sent to the patient's or LAR's device.
2. Research staff discuss the informed consent document with the patient or LAR either in person or by telephone or videophone. This step confirms subject/LAR identity.
3. If the patient or LAR decides to consent to participate, the patient or LAR signs the electronic informed consent document. This signature may be either:
    a. an actual signature (often tracing a finger on the screen) OR

b. a username and password specific to the individual signing

4. Research staff and the witness, provide signatures within the electronic database (e.g., REDCap) confirming their participation in the informed consent process.
5. The image of the signature page may be printed and bundled with a copy of the blank informed consent document for research records.

If a hospital device is provided to facilitate electronic or paper-based consent, that device will be disinfected according to institutional protocols and removed by research staff or clinical staff during the next entry into the patient's room.

This approach complies with relevant regulations and sub-regulator guidance at 45 CFR 46.117, 45 CFR 164.512, 21 CFR 11 Subpart C (11.100–11.300), https://www.hhs.gov/ohrp/regulations-and-policy/guidance/use-electronic-informed-consent-questions-and-answers/index.html , https://www.fda.gov/regulatory-information/search-fda-guidance-documents/informed-consent .

The information for the informed consent discussion will be provided in an informed consent document (or electronic equivalent), that has been approved by the sIRB and in a language comprehensible to the potential participant, using an interpreter if necessary. The information presented in the consent form and by the research staff will detail the nature of the trial and what is expected of participants, including any potential risks or benefits of taking part. It will be clearly stated that the participant is free to withdraw from the trial at any time for any reason without prejudice to future care, and with no obligation to give the reason for withdrawal. Where a patient does not speak English, a short-form consent and qualified interpreter will be employed, using similar "no-touch" principles. Use of an interpreter and the interpreter's identity will be documented on the electronic consent.

6.6.3 Attestation of informed consent

If none of the options outlined above (traditional signature and storage of a paper consent form, electronic photographs of a signed consent page, or e-consent) are available, study personnel may attest to completion of the informed consent process using the procedures outlined below. Importantly, the process of informed consent using this attestation option should not change compared with the traditional method of obtaining informed consent for trial participation except for the method of documenting the consent process in the research record. Rather than storing a paper document with the participant's signature, a member of the research team and an impartial witness will attest to completion of the informed consent process and that the participant signed the informed consent document. This option of attestation of informed consent is not available when obtaining consent through a LAR.

Procedures for attestation of informed consent:

1. An unsigned paper consent form is provided to the patient by a heath care worker or study member.
2. The study member obtaining consent arranges an in-person meeting or three-way call or video conference with himself/herself, the patient, and an impartial witness. If desired and feasible, additional people requested by the patient (e.g., next of kin) may also join this discussion.
3. A study member reviews consent and answers questions in the presence of the impartial witness.
4. Patient signs the paper informed consent document while the witness is listening on the phone or directly observing.
5. Patient provides verbal confirmation that he/she would like to participate in the trial, and he/she has signed and dated the informed consent document. This signed informed consent document stays with the patient due to the risk of spreading the virus.

6. Study member and witness attest that other techniques for documenting informed consent were not available for this participant and that the participant provided written informed consent for trial participation by signing a paper informed consent document. An attestation form with signatures from the study member and witness will be saved as evidence of the informed consent process. A signature from the participant will not be saved in the research record.

6.7 Randomization and blinding

Randomization assignments are performed for patients at enrollment. Randomization will be implemented using a permuted block method and stratified by site and eligibility group. The eligibility group for each participant is the set of study arms for which the participant is eligible. Participants are not eligible for arms that have not begun or have ceased enrollment. Stratification by site ensures balance across the active and pooled placebo groups at regular enrollment intervals at each site, thus mitigating the impact of site heterogeneity on assessments of treatment effect. Eligible participants will be randomized through a central electronic system. On entry to the study and confirmation of eligibility to at least one active drug arm, the participant will be randomized m:1 to either the active (will receive one of the study drugs) or placebo (will not receive one of the study drugs) condition. Here, m is the number of open study arms for which the patient is eligible. If the patient is eligible for only one agent, or only one study arm is open, then allocation is 1:1. For two agents, it is 2:1, for three it is 3:1 and so on. Once participants are assigned as active or placebo, the participant will then be randomized with equal probability to receive one of the active drugs for which they are eligible, or a corresponding placebo (matched by route and frequency of administration). For the purposes of interim and final analyses, the route and frequency of placebo will be ignored, and all placebo participants will be pooled together as a single group. In comparing an active drug versus placebo, only those placebo participants that were eligible for the active drug will be considered. Randomization will be implemented using permuted blocks, stratified by site and eligibility group. A block size multiplier, either 1 or 2, will be selected uniformly at random for each block. Blocking ensures balance within strata across each active and corresponding pooled placebo group and across study arms at the end of each block. Placebos that match the route (e.g., intravenous vs oral) and frequency of the corresponding active agent further ensure patient and assessor blinding. Which study arm the participant enters will be known to the research sites and the participants, but assignment to active versus placebo will be blinded. The randomized assignment, concealed from the research team, will be transmitted to the site pharmacy who will provide study medication. The participant, treating clinicians, study personnel (other than the investigational pharmacist, medical monitor, and the unblinded statistician who prepares closed session DSMB reports), and outcome assessors will all remain blinded to group assignment until after the database is locked and blinded analysis is completed. The medical monitor will remain blinded except as required for individual patient safety. In cases in which unblinding of the medical monitor is required for individual patient safety, assessment of adverse events by the medical monitor will be performed prior to unblinding. After assessing adverse events, the medical monitor will communicate directly with Coordinating Center to receive the unblinded group assignment needed to inform individual patient safety. The medical monitor will not discuss any unblinded information with blinded trial personnel. If an additional adverse event occurs for a patient for whom the medical monitor has been unblinded for individual patient safety, an alternate medical monitor will be appointed to review the adverse event and determine seriousness, severity, and relatedness.

6.7.1 Emergency Unblinding

The investigator has the sole responsibility to break the blind in the case of a patient medical emergency. If the investigator believes unblinding the treatment assignment for a participant is needed for the safe medical treatment of that participant, unblinding will be discussed and

information requested from the Vanderbilt Coordinating Center (VCC). For unblinding requests, including emergency unblinding, please contact the VCC as well as your local CRA.

Unblinding will only occur if determined to be required for patient safety or treatment after discussion between the site investigator and the Medical Monitor. Stopping of study drug without unblinding may be deemed to be sufficient in some cases. If a site investigator determines that unblinding may be warranted, the following steps should occur:

1. Site investigator determines unblinding may be warranted.
2. Site investigator contacts the VCC by phone to request unblinding. The unblinding request will be transmitted via the VCC to the Medical Monitor.
3. Medical Monitor will review the unblinding request and discuss with the site investigator regarding the need for unblinding.
4. If unblinding is determined to be warranted, Medical Monitor documents and certifies need for unblinding in REDCap eCRF, accesses the randomized group assignment, and transmits this information to the site investigator.
5. At sites outside the United States, the site investigator may unblind prior to contacting the medical monitor.

6.8 Vulnerable Subjects

Prisoners will not be enrolled due to difficulty obtaining follow-up for the primary outcome after hospital discharge. Children will not be enrolled because children typically do not display symptoms associated with COVID-19 and therefore are less likely to be hospitalized (the setting in which this study will be conducted). Pregnant women will not be enrolled due to potential teratogenicity of the investigational agents.

This trial may include participants who have no capacity to consent but for whom a LAR may provide consent. Patients without the capacity to consent for themselves will have a potential for direct benefit by participating in the trial. Capacity assessment will be conducted by the treating physician based on the standard clinical assessment of capacity and communicated to the study team. When a participant lacks capacity at enrollment, consent will be obtained from the LAR before any study-related procedures begin. Participants’ capacity will be monitored throughout the study by working with the treatment team. If the participant regains the capacity to consent, they will be approached for reconsent, including being informed of their participation in the study and having an opportunity to withdraw from further participation in the study. Consent from a LAR for persons lacking decision-making capacity will conform to local legal requirements.

6.9 Strategies for Recruitment and Retention

Listings of patients admitted to the participating sites with COVID-19 may be reviewed for eligibility by the study team, to identify and recruit potential participants, until study enrollment goals have been met. Participant recruitment will be by direct communication between the inpatient care team and the study team, allowing the treating team the option to advise of any conditions that would preclude any individual patient being approached.

6.10 Duration of Study Participation

Duration of study participation is for 90 days from randomization.

6.11 Participant Withdrawal or Termination

6.11.1 Reasons for Withdrawal or Termination

Participants are free to withdraw from participation in the study at any time upon request. If the nature of treatment makes immediate withdrawal unsafe, withdrawal may be tapered.

An investigator may terminate participation in the study if any situation occurs such that continued participation in the study would not be in the best interest of the participant or the integrity of the study.

Discontinuation of a study agent, regardless of the reason, e.g., patient or physician request, or adverse event, does not constitute study withdrawal. Patient data will still be collected as planned and analyzed as intention to treat unless the participant withdraws consent for continued follow-up.

6.11.2 Premature Termination or Suspension

The platform, or any arm of the study, may be temporarily suspended or prematurely terminated if there is sufficient reasonable cause. Circumstances outside of interim analyses that may warrant termination or suspension include, but are not limited to:

- Determination of unexpected, significant, or unacceptable risk to participants in a strategy, such as excess mortality or serious adverse treatment effects
- Insufficient compliance to the protocol requirements
- Insufficient accrual in a study arm

If the platform stops for safety, noncompliance, or data quality, it may resume once such concerns about safety, protocol compliance, or data quality are addressed and satisfy the requirements of appropriate oversight bodies including but not limited to the sIRB, the DSMB, and the FDA.

Decisions to stop a study arm or the platform based on the accruing data are not considered premature termination or suspension. They will be guided by the decision thresholds described in the statistical analysis plan and augmented by details in relevant Appendices. Such decisions will generally be weighed by the DSMB. Reasons for stopping based upon the data will include safety (DSMB review of AEs) or demonstration of inferiority.

7 Study Procedures and Schedule

7.1 Study interventions

Study agents are described in the agent-specific appendices.

A summary of the trial's schedule of events is listed in Section 7.7 and included in

Event	Baseline & Randomization Day 0	Day 1 – Day 28	Discharge	Day 60	Day 90
Visit Windows		Day 7 and 14 + 1 Day<br>Day 21 + 2 Days<br>Day 28 + 5 Days		+ 10 Days	+ 14 Days
Confirm eligibility	X				
Obtain informed consent	X				
Screen by reviewing medical history and EMR	X				
Pregnancy test,	X				
Randomization	X				
Concomitant medications	X	X[7]			
Record results of SOC laboratory assessments	X[4]	X			

Study-specific blood draws<br>• CBC with diff and LFTs in fostamatinib arm	X[4]	X			
Study-specific biological specimen collection<br>• EDTA plasma<br>• Serum	X[4]	X			
Respiratory failure free days, oxygen free days and hospital free days	X[4]	X	X		
WHO Ordinal Scale	X	X		X	
Mortality	X	X	X	X	X
Sequential Organ Failure Assessment score	X				
Initiate treatment	X				
Continue study medication treatment		X			
Adverse event monitoring	X	X[7]		X	
Record discharge disposition			X		

Table *1* (Following section 7.7).
Timing of study procedures is based on the day and time of randomization, which sets Day 0 and Time 0. The primary outcome will be assessed on Study Day 28 or at the time of death.

Study medications will be administered by clinical or research personnel while the patient is hospitalized. The first dose of study medication will be administered within 6 hours of randomization. In the hospital, medication delivery after the first dose will correspond to the timing of other scheduled medication delivery for the hospital/unit when possible. If the patient is discharged, the study medication will be stopped unless the oral study medication is planned to be continued in the outpatient setting.

, On each day the participant is hospitalized and scheduled to receive study drug, study personnel will review patient records to confirm administration of study drug and document the number and reason for any missed doses. Research personnel will also assess patients daily during hospitalization for up to 28 days post-randomization. If the participant is discharged before the full 28-day period, data will be collected according to a set schedule (Day 1, 3, 7, 14, 21, and 28 post-randomization) beginning at the next corresponding day post-discharge. This includes data regarding oral study medication administration in those subjects who are randomized to arms with oral study medication. These assessments may be completed by phone or electronically (email, text, or survey link) if the patient has been discharged from the hospital. Patients in the fostamatinib arm could require an in-person visit depending on their clinical profile at the time of discharge. This is further defined in Appendix D: Arm 3: Fostamatinib. At day 60 we will collect AEs, the WHO 8-point ordinal scale and assess vital status. A final contact will be made at day 90 to assess vital status.

7.2 Expedited Critical and Major Event Reporting

All efficacy and safety outcome events will be assessed and documented in the patients' study records as outlined in section 13. Events meeting the DSMB-specified *expedited reporting* criteria must be reported immediately to the coordinating center and no later than 24 hours from knowledge of occurrence. Standing SOPs applicable to all sub studies will guide the reporting of adverse

events to ensure they are assessed quickly and are submitted to the DSMB, IRB(s), sponsor and other groups as needed (e.g., FDA). All participating sites will also be expected to comply with any local requirements for reporting.

7.3 Data and Safety Monitoring Plan

[The data and safety monitoring plan (DSMP) is described briefly in Appendix B: Data and Safety Monitoring Plan *and in detail in a separate DSMP document.]*

7.4 Biological specimens

Participants in this Master Protocol are expected to contribute biological specimens for discovery. The biological specimens to be collected, including collection times, processing requirements, and storage and shipping, are described in Appendix C: Minimum Biological Specimen collection, and any additions to this minimum specimen collection will be described in relevant appendices.

7.5 Shared placebo group dose, duration, and route of administration

Each active agent will have a matching placebo. Placebo formulations are described in the appendices for each agent.

7.6 Co-Interventions and Co-enrollment

This trial will control the use of study medications (active and control) during the treatment window. Study arm specific medication contraindications are explained in detail in the study arm specific Appendices. All other treatment decisions will be made by treating clinicians without influence from the protocol. The decision to administer antiviral medications, including remdesivir or convalescent plasma, or immunomodulating medications, including corticosteroids, will be made by treating clinicians and will be recorded in the case report form. We expect usual care to evolve as the study progresses, and this will be defined by the most recent available evidence and local drug supply.

Sponsor and/or protocol leadership may, based upon convincing new evidence, act in the interest of participant protection, and in avoidance of confounding, to exclude/dis-allow use of any specific concomitant therapy found to be reasonably contraindicated for a well-defined portion of the study population. Such a determination may be made, communicated, and implemented by a Protocol Clarification Memo until it is reasonable to amend the protocol for other reasons.

Participants will be asked at screening to agree to refrain from participation in other clinical trials until at least the assessment at Day 90 except for trials comparing different approaches for implementing SOC interventions or those approved by trial leadership.

Co-enrollment in other trials will only be allowed in the US where a co-enrolling trial has been approved by trial leadership. We will consider several principles when considering co-enrollment in the Master Protocol.

1) This will only apply to clinical trials where there is open label enrollment to facilitate interim and final analyses of data for this trial, including treatment interactions, and the attribution of causality of serious adverse events and unanticipated problems.

2) Co-enrollment will not be permitted with trials involving medications with contraindications to co-administration with any study drug for which the participant is eligible. This review and consideration will be similar to consideration of concomitant medications. This assessment will occur prior to randomization in the ACTIV 4 Host Tissue platform. This ensures there are no specific drug-drug interactions in the event the patient is receiving active therapy in the assigned arm.

3) Trials involving medications impacting the RAAS pathway will not be considered.

4) Study procedures for the co-enrolling trial will be considered secondary to the procedures for the RAAS MP. We will aim to collect the primary and key secondary outcomes for the co-enrolling trial but consider the overall participant burden when fulfilling trial procedures for the co-enrolling trial such as additional blood draws and participant assessments.

5) Co-enrollment prior to randomization will be documented. The impact of co-enrollment on the effects of active agents versus placebo on the primary outcome (i.e., heterogeneity of treatment effect) will be examined as a supplementary analysis.

We aim to co-enroll with the ACTIV-4a trial only, an open-label randomized trial now studying the impact of P2Y12 inhibitors, SGLT2 inhibitors and crizanlizumab on top of usual care. Patients will be randomized in ACTIV-4a to one of these medications at a time in an open label fashion. There are no drug-drug interactions with agents in our platform. Patients co-enrolled in ACTIV-4 Host Tissue and the crizanlizumab domain of ACTIV-4a require 36 hours between administration of fostamatinib and the first crizanlizumab co-administration. Patients who receive both an SGLT2 inhibitor and fostamatinib need to be monitored for volume depletion. SGLT2 inhibitors can cause an osmotic diuresis and patients on fostamatinib may develop diarrhea. Based on the anticipated overlap in sites, similarity in inclusion and exclusion criteria, and willingness of patients to co-enroll in other ACTIV studies, we expect less than 5% of patients will be co-enrolled in ACTIV-4a.

7.7 On study monitoring

All patients will be hospitalized at the time they are enrolled in the study and will therefore receive monitoring as a part of routine clinical care, including monitoring by their physicians, nurses, respiratory therapists, and ancillary staff. Clinical and laboratory data obtained as part of routine clinical monitoring will be collected. Those labs required to evaluate secondary and safety outcomes that are not collected as part of usual care will be obtained for the purpose of the study protocol as outlined in section 7.7.6.

7.7.1 Laboratory evaluations

Routine clinical monitoring will follow laboratory results when measured as part of usual care which may include daily complete blood count (CBC), renal function (creatinine/eGFR), electrolytes, D-dimer, CRP, and measures of coagulation (PT/PTT/INR). In the fostamatinib trial a daily CBC and liver function tests will also be performed while in the hospital on study drug.

7.7.2 Clinical evaluations

Between randomization and hospital discharge or end of study drug, study personnel will review the electronic health record daily for potential medication interactions with the host tissue agents being studied (see Appendix D). If a medication considered to be contraindicated with the host tissue agent is discovered, treating clinicians will be contacted to discuss if stopping study drug is appropriate or if the medication in question can be stopped or substituted. Those medications in the fostamatinib arm with absolute contraindications (Appendix D) will be held or study drug stopped. When there are relative contraindications treating clinicians will determine whether an alternative medication would be appropriate or whether the risk-benefit ratio favors continuing the medication with the known potential interaction.

7.7.3 Criteria for stopping drug

Criteria for holding/stopping each trial drug are contained in the trial specific appendices (Appendix D).

To delineate reasons for study drug discontinuation, if study drug is discontinued the study team will be prompted to indicate why it was discontinued. There will be 3 options to choose from:

1. Logistical - i.e. loss of IV access, CT scan, transport, etc. – no AE reporting/recording required
2. Clinical events that did not represent an AE – i.e., Fostamatinib stopped due to starting contraindicated medication – no AE reporting/recording
3. Due to a possible AE or PSESE –study team directed to go to PSESE or AE reporting page in electronic data capture form.

7.7.4 Plan for drug shortages

In the event of a shortage of study drug at a participating trial site, the trial arm will be suspended at that site, but the platform trial will continue.

7.7.5 Baseline variable collection

Baseline is defined as the patient's status at randomization. Physiological measurements and laboratory results obtained in the 24 hours prior to randomization may contribute to baseline data. The baseline study-specific blood draws may be completed at any time between consent and the first dose of study medication. The following information will be obtained to reflect the patient's baseline status:

1. Confirmation of informed consent for trial participation
2. Confirmation of inclusion/exclusion eligibility criteria for trial participation
3. Confirmation of the participant not being pregnant including a study-dedicated pregnancy test for women of childbearing potential (WOCBP).
    a. WOCBP are defined as any female who has experienced menarche, has not undergone surgical sterilization (hysterectomy, bilateral salpingectomy, or bilateral oophorectomy), and has not experienced menopause
    b. Postmenopausal women, defined as someone who has gone through menopause, do not have to take a pregnancy test.
4. Admission data: date and time of presentation, origin (home, skilled nursing facility, rehabilitation/long-term-acute care hospital, nursing home, outside hospital, outside ICU), location at enrollment (ED, hospital ward, ICU)
5. Sociodemographics (such as age, sex, race, ethnicity, height, weight, poverty index)
6. Study-specific blood draws (AngII, Ang(1-7), ACE and ACE2, NTproBNP and Troponin) as indicated for each investigational agent in the Ancillary Biomarker Appendix (Appendix C: Minimum Biological Specimen collection).
7. Comorbidities such as: AIDS, Leukemia, Malignant Lymphoma, Hemiplegia, Cerebrovascular Disease, Prior Myocardial Infarction, Congestive Heart Failure, Peripheral Vascular Disease, Dementia, COPD, Connective Tissue Disease, Peptic Ulcer Disease, History of Hypertension, HIV positive (without AIDS), Alcoholism, Coronary Artery Disease, Solid Tumor, Liver Disease, Diabetes Mellitus, Chronic Kidney Disease
8. Acute signs and symptoms such as: altered mental status, acute hypoxemic respiratory failure, liver function tests, renal function, coagulation studies, chest imaging results
9. Sequential Organ Failure Assessment (SOFA)
10. Chronic use of medications, such as: corticosteroids, ACE inhibitors, angiotensin receptor blockers, non-steroids anti-inflammatory drugs, others
11. Receipt of antiviral medications between hospital presentation and randomization: chloroquine, hydroxychloroquine, remdesivir, lopinavir/ritonavir, others
12. Receipt of immunomodulators between hospital presentation and randomization: corticosteroids, tocilizumab, sarilumab, interferon β, others
13. Receipt of anticoagulation and anti-platelet agents between hospital presentation and randomization
14. COVID-19 vaccination status
15. Receipt of COVID-19 convalescent plasma between hospital presentation and randomization

16. Receipt of anti-SARS-CoV-2 monoclonal antibodies between hospitalization and randomization
17. Receipt of invasive mechanical ventilation, non-invasive ventilation, high-flow nasal cannula, vasopressors, and oxygen therapy at randomization
18. Vital signs

7.7.6 Assessments between hospital presentation and hospital discharge

1. On days of study medication administration (before study administration in those arms where study drug is not an infusion)
    a. Adverse events of any grade severity present prior to the infusion or medication administration
    b. Start and stop (or administration time if oral) times of the infusion of the investigational agent/placebo- and restart time if medication stopped for hypotension
    c. Starting dose of study medication administration
    d. New adverse events of grade 3-4 severity during and after study medication administration
2. Recording of specifics of study treatment according to assigned arm
3. Daily laboratory assessments as part of routine clinical care (CBC, BMP, LFTs, PT/PTT/INR, D-dimer and CRP).
4. Daily vital signs (including blood pressure), secondary outcomes and safety assessments. In the fostamatinib arm a daily CBC and liver function tests will also be performed.
5. Study-specific blood draws (AngII, Ang(1-7), NT-ProBNP and Troponin) as indicated for each investigational agent in the Ancillary Biomarker Appendix (Appendix C: Minimum Biological Specimen collection).
6. Targeted concomitant medications administered daily in the hospital including remdesivir, Corticosteroids, antiplatelet/anticoagulation, convalescent plasma, monoclonal Ab's, antibacterial agents, antiviral agents against SARS-CoV-2, ACEI's, ARBs, beta blockers.
7. Date and time of first receipt of supplemental oxygen, high-flow nasal cannula, non-invasive ventilation, invasive mechanical ventilation, vasopressors, and extracorporeal membrane oxygenation (if applicable)
8. Date and time of final receipt of supplemental oxygen, high-flow nasal cannula, non-invasive ventilation, invasive mechanical ventilation, vasopressors and extracorporeal membrane oxygenation (if applicable)
9. Pulmonary embolism, systemic arterial thromboembolism, myocardial infarction, or ischemic stroke at hospital discharge or 28 days, whichever occurs first
10. Date and time of first ICU admission
11. Date and time of final ICU discharge
12. Data and time of hospital discharge

7.7.7 Assessments following hospital discharge

Patients will be followed through 90 days following randomization. The following data will be collected:

1. Number and reason for missed doses of study drug (only for those discharged prior to completing study drug if applicable)
2. Date of death (if applicable) through day 90
3. ED visits, hospital readmissions, and use of supplemental oxygen, HFNC, NIV, IMV or ECMO after hospital discharge through day 60
4. Safety outcomes (section 5.3.4) after hospital discharge and adverse events as defined in the drug specific appendices and section 13 at day 28 and day 60 (or if discharged earlier as outlined in section 7.1)

5. New or worsening symptoms not previously present at day 28 and day 60 (or if discharged earlier as outlined in section 7.1) including fever, chills, cough, chest pain, dyspnea, headache, sore throat, congestion, runny nose, fatigue, body aches

Table 1. Schedule of Events

Event	Baseline & Randomization Day 0	Day 1 – Day 28[1]	Discharge	Day 60	Day 90
Visit Windows		Day 7 and 14 + 1 Day<br>Day 21 + 2 Days<br>Day 28 + 5 Days		+ 10 Days	+ 14 Days
Confirm eligibility	X				
Obtain informed consent	X				
Screen by reviewing medical history and EMR	X				
Pregnancy test[2,3]	X				
Randomization	X				
Concomitant medications	X[4]	X[7]			
Record results of SOC laboratory assessments	X[4]	X			
Study-specific blood draws[5]<br>• CBC with diff and LFTs in fostamatinib arm	X[4]	X			
Study-specific biological specimen collection[6]<br>• EDTA plasma<br>• Serum	X[4]	X			
Respiratory failure free days, oxygen free days and hospital free days	X[4]	X	X[7]		
WHO Ordinal Scale	X	X		X	
Mortality	X	X	X	X	X
Sequential Organ Failure Assessment score	X				
Initiate treatment[8]	X				
Continue study medication treatment		X			
Adverse event monitoring	X	X[7]		X	
Record discharge disposition			X		

[1] Perform events daily through day 28 or discharge, whichever occurs first. Only perform if patient is hospitalized.
[2] Perform for all women of childbearing potential.
[3] Perform only if not completed for current admission.
[4] Perform prior to treatment administration
[5] Only in the fostamatinib arm if not performed as part of usual care – performed daily while on study drug as an inpatient. A repeat CBC, LFTs and BP check may be necessary depending on values at discharge as indicated in Appendix D: Arm 3: Fostamatinib.
[6] Coordinate with clinical lab draws when possible- as delineated in Appendix C: Minimum Biological Specimen collection
[7] Collect at Day 1, 3, 7, 14, 21, and 28 post-randomization if patient is discharged before 28 days.
[8] Administration route and timing/frequency is treatment specific

8 Statistical Considerations

This section describes the statistical approach for each comparison of active treatment versus its concurrent and eligibility matched placebo comparator group.

8.1 Statistical and Analytical Plans

There will be a formal Statistical Analysis Plan (SAP). This SAP will provide detailed descriptions of all primary, secondary, and sensitivity analyses, all interim and final decision thresholds, and all required documentation to ensure the reproducibility of statistical analyses. The SAP will be finalized prior to the first interim analysis for the platform, and arm-specific SAP amendments (if required) will occur before the first interim analysis involving that arm. The finalized SAP will take precedence and override the statistical considerations described in this section of the master protocol (i.e., Statistical Considerations).

8.2 Analysis Datasets

All sub studies conducted under this protocol will use a modified intention-to-treat (mITT) approach for primary analyses. The mITT analysis dataset (i.e., the “full analysis set”) will include all randomized participants according to the treatment assigned at randomization regardless of subsequent compliance or protocol violations, with the following exceptions: Participants who do not receive study drug will be excluded from the mITT analysis dataset. Those patients who were randomized and found to be ineligible will be excluded from the mITT analysis dataset. The safety analysis dataset will be produced, which will consist of all participants who received at least one dose of study medication grouped by the drug received. No statistical hypothesis tests nor other statistical inferences will be made using the safety analysis dataset unless requested by the DSMB.

Per protocol analyses will not be routinely performed but may be conducted as sensitivity analyses to support the mITT analysis. A key monitoring data point is the count of participants who are randomized and included in the ITT dataset but have not received study drug. We expect this to be less than 5% among participants who have completed the study. If this becomes greater than 5% during the trial, study leadership will meet with study teams to explore and mitigate this issue and will ensure the number of patients who receive study drug meets the original enrollment goals. We will report results for both the mITT (primary) and ITT datasets.

8.3 Statistical Modeling

The effect of each study agent versus matching placebo will be quantified using an odds ratio. The odds ratio represents the treatment effect on the odds of greater values of the primary outcome (i.e., improved lung function through 28 days, as measured by oxygen-free days). Based on the behavior of similar outcomes in prior trials,[32–36] we anticipate the distribution of the primary outcome to be irregular, with peaks around -1 to 0 and between 22 and 28 days. Thus, we will use a flexible semi-parametric approach for the primary outcome analysis. Estimation and inferences about the odds ratio will be made using Bayesian proportional odds (PO) logistic regression methods.[40,41] For each study agent, the comparator group will consist of participants concurrently randomized to receive placebo who also meet the inclusion and exclusion criteria for that agent.

The general form of the PO model can be written in terms of the covariates X and an outcome variable Y, where probabilities of outcome value y or greater$\Pr(Y \geq y|X) = \mathrm{expit}(\alpha_y + X\beta)$ where α_y is the intercept for outcome value y and expit is the logistic (inverse logit) transformation and X contains baseline covariates and treatment. β represents the log odds ratio (OR) associated with

the effects of covariates and group assignment. Specifically, the odds ratio represents the relative effect of treatment versus placebo on the odds $\Pr(Y \geq y|X)/(1 - \Pr(Y \geq y|X))$, for any value y.

8.4 Loss to Follow-up, Censoring, and Intercurrent Events

Participants who withdraw consent prior to data collection, or for whom there is no partial information about the primary outcome, will not be excluded from analysis. We will strive to avoid loss to follow-up by making repeated attempts to contact participants or otherwise retrieve participant records. If loss-to-follow-up cannot be avoided, and the information needed to compute the primary endpoint is partially known (i.e., censored), we will use likelihood-based methods to account for this censoring. For example, if a study participant received supplemental oxygen every day during a 10-day period after randomization, but is then lost to follow-up, the primary outcome is only partially known (i.e., OFDs ≤ 18 in this example). The PO model provides a convenient mechanism to account for this and other types of censoring using a likelihood-based approach.[42] For observations that are fully observed, the log likelihood contribution is $l(\alpha, \beta;\ y, x) = \log \Pr(Y = y|X = x)$. For observations that are left censored at y (e.g., ≤ 18 OFDs) observations, the log likelihood contribution is $l(\alpha, \beta;\ y, x) = \log \Pr(Y \leq y|X = x)$. The latter is conveniently computed by substituting $1 - \text{expit}(\alpha_y + x\beta)$. Censored observations on the primary outcome due to loss of follow-up, including observations that are censored with respect to both oxygen requirement and mortality, will be handled using this mechanism.

All primary analyses will be implemented using the mITT analysis dataset as described above (see *Analysis Datasets*). The intercurrent event of death will be coded as a special value in the primary outcome (i.e., composite strategy). Censoring in the primary outcome will be modeled using the likelihood method described above. No other intercurrent events will affect the primary outcome assessment (i.e., treatment policy strategy).[43]

8.5 Model Prior and Bayesian Computation

A flat prior distribution will be used for all PO model parameters. This ensures that the estimate of the primary estimand will be free of influence from an informative prior, and the Bayesian estimate will be identical to the maximum likelihood estimate. The posterior distribution for the log odds ratio will be approximated using the Laplace method.[44] Use of a flat prior ensures the Laplace-approximated posterior distribution is identical to the asymptotic sampling distribution of the maximum likelihood estimate; in both cases a normal distribution centered at the estimate with variance-covariance equal to the inverse Hessian of the log likelihood function. All statistical inferences about the odds ratio will be made using this method. Statistical uncertainty about supplementary estimands (e.g., treatment difference in the median of the primary outcome) will be quantified using the delta method.[45] We feel there is insufficient information, specific to the study agents and primary outcome, upon which to justify a more informative prior. The flat prior approach ensures that Bayesian inferences regarding the efficacy of study agents are based exclusively on the data collected in the ACTIV-4 Host Tissue trial.

8.6 Analysis of Primary Outcome

8.6.1 Primary Analysis

The effect of each study agent versus matching placebo will be quantified using an odds ratio, which quantifies the treatment effect on the odds of greater values of the primary outcome. Estimation and inference about the primary estimand (and supplementary estimands) will be implemented using Bayesian PO logistic regression methods, adjusting for the active drug vs placebo indicator variable, age group (18-30, 31-65, >65 years), sex at birth, baseline WHO COVID

ordinal outcome score at baseline. Evidence for efficacy will be quantified using the posterior probability that the active agent versus placebo odds ratio is greater than one (i.e., treatment is associated with greater oxygen free days at day 28). This is denoted the "efficacy probability" or $P(\mathrm{OR} > 1|\mathrm{Data})$, where OR represents the odds ratio, and Data represents the available outcome data. The posterior probability for inferiority/harm is defined as $P(\mathrm{OR} \leq 1|\mathrm{Data})$. The primary analysis will be implemented separately for each study agent, where the matching placebo group will consist of concurrently randomized participants meeting the inclusion and exclusion criteria for that agent. The primary and supplementary estimands will be presented with 95% credible intervals. While we do not anticipate missing covariate data.

8.6.2 Planned Interim and Final Analyses, Early Stopping, and Type-I Error Control

At the final analysis (only) for each arm, efficacy will be indicated if the posterior probability for efficacy exceeds a common threshold. For studies under this master protocol, the efficacy threshold will be selected using statistical simulation to ensure a type-I error probability of 2.5% for each study agent.

Two planned interim analyses will occur separately for each study arm when the number of participants with complete 28-day follow-up (or were deceased, withdrawn, or lost-to-follow-up by day 28) reaches 33% and 67% of maximum enrollment for that arm. Interim analyses will be executed by unblinded personnel only. Participant records that inform the primary outcome must undergo monitoring prior to interim (and final) analysis. At each interim analysis, a study arm may be stopped early if there is evidence for inferiority/harm. The trial will be stopped early if the posterior probability for inferiority/harm exceeds 0.95.

8.6.3 Supplementary Efficacy Estimands

The PO model is attractive for the analysis of ordinal and quantitative response variables, such as the primary outcome, because they directly model the cumulative distribution function from which the mean, median, other percentiles, and cumulative probabilities of the primary outcome, stratified by treatment group, are easily derived.[46] In addition to the odds ratio, the effects of treatment versus placebo will be quantified using the difference in mean, difference in median, and differences in clinically relevant proportions associated with the primary outcome (e.g., mortality at day 28: $\Pr(Y = -1|X)$, and oxygen requirement every day until day 28: $\Pr(Y = 0|X)$). These important and clinically meaningful supplementary estimands will be used to describe and communicate the treatment effect. The posterior distribution for each of the supplementary estimands is readily computed using standard Bayesian methods.

8.6.4 Sensitivity and Supplementary Analyses

Sensitivity and supplemental analyses will be implemented at the final analysis.

The *proportional odds assumption* of the PO model specifies that the effect of treatment on the odds that $\mathrm{Y} \geq 3$ (measured as an odds ratio versus placebo) is the same relative effect as for $\mathrm{Y} \geq 4$. However, even when the PO assumption is strongly violated, the estimated OR remains a simple function of the Wilcoxon-Mann-Whitney U-statistic, namely the probability that a randomly chosen patient on treatment B has a higher response than a randomly chosen patient on treatment A,[47] the *probability index* or *concordance probability*. Thus, statistical testing based on the odds ratio, as estimated using the PO model, are robust to violations of the PO assumption and provide a reasonable global assessment of treatment effectiveness. However, derived quantities such as the difference in means may be more sensitive to violations of the PO assumption. Deviations from

proportional odds will be examined by separately estimating the odds ratio for each possible dichotomization (that preserves ordering) of the primary outcome (e.g., alive versus dead at day 28, alive and oxygen free for at least 10 days at day 28 versus alive and oxygen free for fewer than 10 days or dead at day 28, etc.).

Analysis of partially observed or missing outcome data requires assumptions regarding the mechanism by which censoring and missing values arise. The likelihood method described above, and other similar methods such as multiple imputation assume that missing values occur at random (i.e., missing at random or MAR). However, because censored and missing values cannot be observed, assumptions about the missingness mechanism are not verifiable. In order to assess the sensitivity of study findings to violations of this assumption, we will conduct additional sensitivity analyses by reproducing the primary analysis under alternative assumptions regarding the mechanism for missing values. Specifically, we will perform sensitivity analyses that vary assumptions about the missing outcomes on the two treatment arms separately. These analyses will consider the following two scenarios 1) each partially observed primary outcome in the placebo group will be assumed to have taken the highest/best possible value, whereas each partially observed primary outcome in the intervention group will be assumed to have taken the lowest/worst possible value, and 2) each partially observed primary outcome in the placebo group will be assumed to have taken the lowest/worst possible value, whereas each partially observed primary outcome in the intervention group will be assumed to have taken the highest/best possible value. These analyses will be implemented using the primary analysis methodology, including an assessment of hypothesis testing outcomes.

Co-enrollment in other studies testing COVID-19 therapeutics may occur. Co-enrollment may affect the treatment effect estimates if there is effect modification associated with co-enrollment. We expect co-enrollment to occur in fewer than 5% of patients enrolled in the trial. However, because the decision to co-enroll is not affected by the treatment assignment in ACTIV-4 Host Tissue, co-enrollment will not favor any particular treatment. In addition, due to its rarity, we expect co-enrollment to have little impact on the estimated treatment effects, even when there is effect modification.

Differential treatment effect, also referred to as heterogeneity of treatment effect, refers to differences in efficacy as a function of pre-existing patient characteristics such as baseline variables. This is often assessed by forming subgroups or using an interaction analysis. Supplemental interaction analyses will be implemented to examine the potential for differential treatment effect. Differential treatment effect will be examined in strata defined by (but not limited to) respiratory support category at enrollment, status of co-enrollment in an open label clinical trial of antiplatelet agents (ACTIV-4a), age category, SARS-CoV-2 vaccination status, and passive immunity status, co-enrollment in other studies, and concomitant use of study drug and other medications during the study drug administration period. These analyses will be implemented using a modified version of the primary analysis method, where the treatment effect will be estimated separately for each level of the stratification variable. Stratum-specific treatment effect estimates will be presented with 95% Bayesian confidence interval. No formal hypothesis testing will be implemented for these analyses. Studies under this master protocol will be sized only for assessing efficacy using the primary analysis. Thus, there may be inadequate power to examine differential treatment.

8.6.5 Sample Size and Decision Thresholds

The maximum number of participants to be enrolled in sub studies under the Master Protocol is 600 patients per trial, resulting in approximately 300 patients per active treatment arm, and 300 patients in the matching placebo arm. The placebo arm will be shared across all active treatment arms. We expect control arm participants to continue to accrue for as long as there are additional treatments to test and cases to enroll. New arms may be introduced according to scientific and public health needs.

Type-I error and power regarding the analysis of the primary outcome was assessed based on the pooled (across all active and placebo arms) distribution of the primary outcome among the first 100 participants to complete follow-up and monitoring. The efficacy threshold was identified using statistical simulation under the null hypothesis to ensure the study operating characteristics achieve design specifications. Pooled and blinded summaries of oxygen-free days at day 28 were used to approximate the distribution of the oxygen free days in the placebo group. Based on these data, the anticipated frequency distribution, mean, and median of oxygen-free days (OFDs) for the placebo group, and for the treatment group under hypothetical effect sizes computed using the PO model are displayed in the table below.

		**Inferiority**		**Superiority**						
**OFDs / Odds Ratio**	**Placebo**	**0.67**	**0.80**	**1.40**	**1.45**	**1.50**	**1.55**	**1.60**	**1.65**	**1.70**
Mean	8.8	6.6	7.5	10.8	11.1	11.3	11.5	11.7	11.9	12.0
Median	0	0	0	6.5	7.5	9.0	10.0	10.5	12.5	14.5
P(OFDs >= 22)	0.19	0.14	0.16	0.25	0.26	0.26	0.27	0.28	0.29	0.29
Proportion:										
-1 (death)	0.235	0.316	0.279	0.181	0.176	0.171	0.166	0.162	0.158	0.154
0	0.296	0.314	0.309	0.268	0.264	0.261	0.257	0.254	0.251	0.247
1	0.006	0.006	0.006	0.006	0.006	0.006	0.006	0.006	0.006	0.006
---	---	---	---	---	---	---	---	---	---	---
27	0.050	0.034	0.041	0.069	0.072	0.074	0.076	0.078	0.081	0.083
28	0.000	0.000	0.000	0.000	0.000	0.000	0.000	0.000	0.000	0.000

Based on these data and effect size scenarios, a series of statistical simulations were implemented to examine the operating characteristics of the statistical study design described above, including the plan for randomization, interim analysis, and final assessments of efficacy using the odds ratio. In each simulation, participant age group, sex, and baseline WHO COVID severity score were randomly sampled with replacement from the values observed, and their effects on the primary outcome were simulated to match the estimated effects of age, sex, and WHO score on the primary outcome among the first 100 participants and loss-to-follow-up, partially observed oxygen free days were simulated to match the observed frequency of partially observed outcomes, which occurred in 12% of the first 100 participants. To encode attrition, a subset of the simulated study participants was selected at random, each with probability 0.12. The primary outcome for each selected participant was encoded as partially observed by assuming that oxygen free days may have taken any value between -1 and a randomly sampled value ranging from the simulated oxygen free days to 28. For example, if the simulated oxygen free days is 10, then a value between 10 and 28 is sampled uniformly at random and this value is treated at the upper limit for the partially observed oxygen free days. This pattern of partially observed oxygen free days closely resembles the patterns observed among the first 100 participants. All simulation analyses, including those

associated with interim and final assessment of efficacy and inferiority were implemented using the methods described above for the analysis of the primary outcome.

Simulation under the null hypothesis was used to select the efficacy threshold for the final analysis of the primary outcome. The efficacy threshold was selected to ensure no more than 2.5% type-I error. In this simulation, 10000 replicates were used to ensure ~0.31% simulation margin of error in estimating the type-I error rate. The efficacy threshold was identified as 0.9760. The efficacy and inferiority/harm thresholds will be applied as follows:

Analysis	Rule	Result
Final analysis (only)	Efficacy probability > 0.976	Conclude efficacy
Each interim and final analysis	Inferiority/harm probability > 0.950	Stop and conclude inferiority/harm

Using the selected efficacy and inferiority/harm thresholds, the results of 10000 simulations under the null hypothesis, and 1000 simulations per inferiority/efficacy scenario are summarized in the table below. In these simulations, the type-I error probability was 2.47%. The frequency of stopping early for inferiority under the null was 8.6%. *A maximum sample size of 600 participants per arm (including matching placebo) provides greater than 85% power to detect an odds ratio of 1.65, corresponding to a 3.1-day difference in mean OFDs, and a 7.8 percentage point reduction in 28-day mortality.* Differences larger than 2 ventilator-free days on average have been considered clinically important in prior trials.[32–34] *Thus, the minimum detectable effect with 85% power (MDE85) is an odds ratio of 1.65.* The frequency of stopping early for inferiority when there was an effect larger than OR=1.40 was <1%. When the simulated treatment was inferior/harmful relative to placebo, at OR=0.67, a conclusion of inferiority/harm occurred in 83.3% of simulated trials (39.1%at the first interim, 27.9% at the second interim, and 16.3% at the final analysis), and the average sample size was 193.9 participants receiving the active drug.

	**Null**	**Inferiority**		**Superiority**						
**OFDs / Odds Ratio**	**1.00**	**0.67**	**0.80**	**1.40**	**1.45**	**1.50**	**1.55**	**1.60**	**1.65**	**1.70**
Pr(Efficacy)	**0.025**	0.000	0.001	**0.552**	**0.631**	**0.705**	**0.782**	**0.826**	**0.856**	**0.893**
Pr(Inferiority)	0.108	0.833	0.508	0.003	0.002	0.001	0.000	0.000	0.001	0.000
Pr(Inconclusive)	0.867	0.167	0.491	0.445	0.366	0.294	0.218	0.173	0.143	0.107
Average(N)	286.1	193.9	242.0	299.4	299.8	299.8	300.0	300.0	299.8	300.0

In order to characterize the effect of uncertainty in the distribution of the OFD outcome on the type-1 error probability, simulations under the null hypothesis were twice repeated assuming a “mild” and “severe” distribution for the OFD outcome. The mild and severe distributions were selected such that the unadjusted mortality rate ranged ± 3% relative to the initial simulation. The results of 1000 simulations in each of the mild placebo and severe placebo scenarios are summarized in the table below. In these simulations, the type-I error probability was 2.5% and 2.3%.

	**Severe**	**Mild**
	**OR = 1.00**	**OR = 1.00**
Mortality rate	0.266	0.206
Pr(Efficacy)	**0.023**	**0.025**

	Severe	Mild
	**OR = 1.00**	**OR = 1.00**
Pr(Inferiority)	0.0.119	0.117
Pr(Inconclusive)	0.858	0.858
Average(N)	284.0	286.4

Prior to the start of enrollment, initial sample size assessments were based on pooled and blinded summaries of OFDs from the PassItOn (convalescent plasma) trial of patients hospitalized for COVID-19. The inclusion and exclusion criteria for PassItOn are similar to that for ACTIV 4 Host Tissue. In these initial assessments, the estimated MDE85 was OR=1.55. Statistical power was subsequently reassessed using OFDs summaries in the first 100 participants enrolled in ACTIV 4 Host Tissue, which demonstrated a more severe distribution relative to PassItOn participants (23.6% vs 17.6% mortality). The estimated MDE85 was OR=1.65 at the time of sample size reassessment. However, additional information from blinded summaries of the first 200 enrolled participants are consistent with the distribution of OFDs observed in PassItOn (18.6% vs 17.6% mortality). After discussion of these findings among the blinded study investigators and study sponsor, it was determined that statistical power was sufficient, and no sample size adjustment was warranted.

8.7 Analysis of Secondary Outcomes

The effect of active agent versus placebo on the odds of binary and ordinal secondary outcomes will be quantified using logistic and PO regression methods, respectively, adjusting for patient demographic and clinical factors (see *Primary Analysis*). Time-to-event outcomes will be analyzed using Cox proportional hazards methods. To incorporate death as an appropriately unfavorable possible outcome, deaths will be treated as censored at the end of the evaluation period for the endpoint (e.g., Day 28). Where appropriate, the competing risk of death will be addressed using the cause-specific hazards method. The proportion of participants who died at fixed time points (e.g., day 28) will be estimated using Kaplan-Meier methods. In order to preserve consistency across the primary and secondary analyses, we will uniformly apply a Bayesian approach using flat priors. For each key secondary outcome, efficacy testing (one-sided) will be assessed using the odds ratio or hazard ratio, by comparing the corresponding posterior probability of efficacy to a threshold. Each threshold will be selected using either a simulation-based method, or an approach similar to most conventional statistical testing procedures, to ensure each test for efficacy has a type-I error probability no more than 2.5%. Odds ratio, hazard ratio, and differences in proportions (e.g., death at 28 days) estimates will be presented with a 95% credible interval.

A gatekeeping/fixed-sequence testing approach will be used to preserve the type-I error rate across tests of the primary and secondary outcomes. Specifically, a conclusion of efficacy regarding the primary outcome will be required prior to testing the key secondary outcomes. The fixed-sequence method will be used to test the following key secondary outcomes in the order given: alive and respiratory failure-free at day 28, the WHO 8-point ordinal scale at day 28, and mortality at day 28. A one-sided type-I error rate of 2.5% will be used for each test. This approach preserves the familywise type-I error rate for the family of primary and key secondary outcomes. Heterogeneity of treatment effect may be examined for secondary and safety outcomes, as a function of pre-existing patient characteristics and baseline variables.

8.8 Analysis of Safety Outcomes

Monitoring and reporting of safety events will be conducted continuously as described in the Data and Safety Monitoring Plan. This section describes the assessment of safety endpoints at the interim and final analyses. Agent-specific safety and toxicity endpoints (if any) are detailed in that therapy's appendix. The frequencies of adverse events, mortality, and other safety endpoints, and the treatment effect on the odds of these events (i.e., the odds ratio) will be reported with 95% credible intervals, using Bayesian ordinal and binary logistic regression methods in a manner similar to that described for the analysis of secondary outcomes.

8.9 Adherence and Retention Analyses

Receipt of planned therapy will be recorded on case report forms and monitored continuously. Should minimum adherence not be achieved routinely, the arm may require modification. Adherence, retention, and accrual will be reported to the DSMB and may be considered as reasons for premature termination or suspension of arms, or the entire platform.

8.10 Baseline Descriptive Statistics

All variables will be summarized using median and other quantiles, mean, and Gini's mean difference (a robust measure of variability defined as the mean absolute difference between any two patients' values). Variable summaries will be presented by treatment group. Because treatments are randomized, differences in baseline characteristics will not be formally tested with respect to treatment groups. Emphasis is placed on describing the patient sample. In the case that inclusion criteria differ across the various treatment arms, treatment specific summaries will be made by combining patients enrolled in each specific treatment arm and its matching placebo group.

8.11 Exploratory Analyses

Exploratory analyses may proceed as specified within arm-specific SAPs. Exploratory analyses that are not specified prior to data collection, such as exploration of the association between novel biomarkers and treatment response, are acceptable. In general, the SAP for such exploratory analyses should be specified prior to executing the exploratory analysis.

9 Measures to Minimize Bias

9.1 Enrollment/Randomization/Blinding

All participants meeting eligibility for inclusion will be screened for exclusion criteria. Reasons for exclusion will be documented. Monitoring for systematic exclusions will be continuous and failure to screen and enroll without bias may result in termination of a site from the trial.

To prevent bias in allocation of participants to individual sub studies or to arms within sub studies, participant eligibility should be confirmed prior to releasing the randomization allocation. Randomization will occur at baseline and will generally be equal across all arms for which a patient is eligible unless specified in an arm-specific appendix. Randomization will be stratified by study site.

Blinding of patients, providers, and study team members to study arm allocation will be employed to reduce bias in conducting study activities and evaluations. Special precautions may be needed to blind outcomes assessors if patients or investigators are unblinded to treatment assignment.

10 Source Documents and Access to Source Data/Documents

Source documents are original documents, data, or records that are created during a clinical study, relating to the medical treatment and the history of the participant, and from which study data are

obtained. The purpose of source documents is to document the existence of study participants and substantiate the integrity of the study data collected. Any document in which information, an observation, or data generated relevant to a study is recorded for the first time is a source document.

Each study participant will sign a consent form, which includes language on who may access their source data and documents used for the study. Locations where study data are generated must allow access to source documents as part of clinical study monitoring and oversight.

11 Quality Assurance and Quality Control

Quality assurance (QA) is implemented by the study team through a system of best-practice standards, reviews, and corrective actions ensuring products and services are of the highest achievable quality. The study team and staff members participate in a number of quality activities, ensuring the sponsor, OHRP, and FDA research standards are met. QA also encompasses independent QA oversight processes verifying the quality of the work through independent reviews, qualifications, inspections, and audits, assuring research staff members, contractors, and service providers are following the best research and professional practices.

Quality control (QC) activities include data entry checks in the electronic data capture (EDC) system, centralized monitoring, in-person or remote site monitoring, and other activities. To monitor studies, clinical monitoring staff review research records and regulatory documents. Reports generated from the EDC system may also guide discussions with site research staff.

12 Ethics/Protection of Human Subjects

12.1 Ethical Standard

All studies conducted under this Master Protocol will adhere to the highest ethical standards. The trial will be carried out in compliance with the protocol, the ethical principles laid down in the Declaration of Helsinki, in accordance with the ICH Harmonized Guideline for Good Clinical Practice (GCP), the EU directive 2001/20/EC/EU regulation 536/2014 and other relevant regulations. Further, studies will be conducted in full conformity with Regulations for the Protection of Human Subjects of Research codified in 45 CFR Part 46, 21 CFR Part 50, 21 CFR Part 56, and/or the ICH E6.

12.2 IRB/Ethics Committee/Competent Authority

This trial will be initiated only after all required legal documentation has been reviewed and approved by the Vanderbilt University Medical Center's IRB (serving as the single IRB [sIRB])/ Independent Ethics Committee (IEC) and competent authority (CA) according to national and international regulations. The same applies for the implementation of changes introduced by amendments to both the protocol and informed consent form. A determination will be made regarding whether previously consented participants need to be re-consented.

12.3 Posting of Clinical Trial Consent Form

The informed consent form approved by the sIRB for US sites will be posted on the clinical trials.gov website after the clinical trial protocol is finalized and the first patient is enrolled.

12.4 Participant and Data Confidentiality

Participant confidentiality and privacy is strictly held in trust by the participating investigators, their staff, and the sponsor(s). This confidentiality is extended to cover testing of biological samples and genetic tests in addition to the clinical information relating to participants.

All investigators and trial site staff will comply with the requirements of the current General Data Protection Regulations (GDPR) where applicable with regards to the collection, storage, processing and disclosure of personal information and will uphold the applicable local regulations core principles. Personal information collected will be kept secure and data access will be limited to the minimum number of individuals necessary for quality control, audit, and analysis.

The study monitor, other authorized representatives of the sponsor, representatives of the sIRB, and regulatory agencies may inspect all documents and records required to be maintained by the investigator, including but not limited to, medical records (office, clinic, or hospital) and pharmacy records for the participants in this study. The clinical study site will permit access to such records. In the case that a pharmaceutical company holds an IND for an agent contributing to this study and on which this study relies, the pharmaceutical company supplying study product may also inspect study records.

The study participant's research information will be securely stored at each clinical site and transmitted to and securely stored at the Data Coordinating Center. The study data entry and study management systems used by clinical sites and by the Data Coordinating Center research staff will be secure and password protected. Wherever feasible, data will be identified by a Participant ID number, and not by any direct identifiers. At the end of the study, all records at a clinical site will continue to be kept in a secure location for as long a period as dictated by the reviewing sIRB/IECs, Institutional policies, country-specific/local regulations, or sponsor requirements. On completion of the study, de-identified data may be made available to others outside the study team.

12.5 Certificate of Confidentiality

To further protect the privacy of study participants, a Certificate of Confidentiality will be issued by the NIH. This certificate protects identifiable research information from forced disclosure. It allows the investigator and others who have access to research records to refuse to disclose identifying information on research participation in any civil, criminal, administrative, legislative, or other proceeding, whether at the federal, state, or local level. By protecting researchers and institutions from being compelled to disclose information that would identify research participants, Certificates of Confidentiality help achieve the research objectives and promote participation in studies by helping assure confidentiality and privacy to participants.

13 Adverse events

Assuring patient safety is an essential component of this protocol. Use of these agents for COVID-19 raises unique safety considerations. This protocol addresses these considerations through:

1. Exclusion criteria designed to prevent enrollment of patients likely to experience adverse events with receipt of these agents
2. Proactive education of treating clinicians regarding medication interactions relevant to use of these agents in the inpatient setting
3. On-study monitoring of co-interventions and patient characteristics to intervene before adverse events occur
4. Systematic collection of outcomes relevant to the safety of these agents in this setting
5. Structured reporting of adverse events

The safety and monitoring approach in this platform is aligned with the expected impact of the investigational agents in the hospitalized COVID-19 population. All of the investigational agents have short half-lives, and it is expected their biologic effect would be seen during or shortly after treatment. Thus, the focus of safety monitoring through day 60 will be broad safety monitoring and reporting of serious adverse events felt to be at least possibly related to the investigational agent. Importantly, patients with COVID-19 often experience multisystem illness, including ARDS, cardiac

and renal injury. As a result, many anticipated serious adverse events will be collected as study outcomes (protocol-specified exempt serious events (PSESEs) as listed in section 13.2) and will be monitored by the DSMB rather than be subject to strict reporting criteria associated with adverse events. Adverse events and PSESEs will be monitored to ensure real-time participant protection. The safety evaluation of the study intervention includes several components to be reviewed regularly by the NHLBI-appointed independent DSMB.

All other AEs are collected for the study intervention (either the blinded investigational agent or placebo).

Events will be reported to regulators and IRBs/IECs as appropriate/required.

Adverse events and unanticipated problems will be regularly reviewed by the DSMB.

The following information will be collected on electronic case report forms, and will be regularly reviewed by the DSMB, to evaluate and help ensure safety:

- Deaths through Day 90
- Hospital readmissions through Day 60
- Protocol-specified exempt serious events (PSESEs) (see section 13.2) through Day 60
- Adverse Events that are Serious OR are Definitely or Possibly Related (or of Uncertain Relationship) OR are a Grade 3 or 4 Clinical AE (isolated laboratory abnormalities that are not associated with signs or symptoms are not collected)

We outline the safety data collected in Table 2.

Table 2. Overview of Safety Data Collection

	Day 0–5	Day 14	Day 28	Day 60	Day 90
All grade 3 and 4 clinical AEs (new or increased in severity to Grade 3/4)	X	X[a]	X[a]	X[a]	
Protocol-specified exempt serious events (PSESEs)[b]	X	X	X	X	
Recordable AEs that are not PSESEs	X	X	X	X	
Unanticipated Problems	X	X	X	X	
Mortality	X	X	X	X	X

[a]Participants will be asked about all new relevant adverse events which have occurred since the last data collection, up to that time point. On these visits, qualifying AEs will be collected.
[b]These are explained and defined in section 13.2.

13.1 Defining adverse events

Adverse Events will be defined as any untoward medical occurrence associated with use of the study drug or study procedures, whether or not the event is related to the study drug or study procedures. If a diagnosis is clinically evident (or subsequently determined), the diagnosis, rather than the individual signs and symptoms or lab abnormalities, will be recorded as the AE.

a. Seriousness:

Serious Adverse Event will be defined as an adverse event that, in the view of the investigator, resulted in any of the following outcomes:

1. **Death**
2. **A life-threatening event that places the patient at immediate risk of death**
   - *Does not include events that, had they been more severe, might have caused death*
3. **Inpatient hospitalization or prolongation of existing hospitalization**
   - *As per http://www.fda.gov/Safety/MedWatch/HowToReport/ucm053087.htm*
4. **Persistent or significant incapacity or substantial disruption of the ability to conduct normal life functions, or a congenital anomaly/birth defect**
   - *As per http://www.fda.gov/Safety/MedWatch/HowToReport/ucm053087.htm*

Important medical events that may not result in death, be life-threatening, or require hospitalization may be considered serious when, based on appropriate medical judgment, they jeopardize patient safety or require medical or surgical intervention to prevent one of the outcomes listed in this definition.

b. Causality:

A **Related or Possibly Related Adverse Event** will be defined as any adverse event for which there is a "reasonable possibility" of a causal relationship between the study drug or study procedure and the adverse event. For each recorded adverse event, investigators will grade the strength of the relationship of study drug or study procedure to the adverse event, as follows:

- Definitely Related: The adverse event meets all three of the following criteria: (a) a temporal sequence from receipt of study drug or study procedure to the adverse event suggests relatedness, (b) the event cannot be explained by the known characteristics of the patient's clinical state or other therapies, and (c) evaluation of the patient's clinical state indicates to the investigator the experience is definitely related to study drug or study procedures.
- Possibly Related: In the investigator's opinion, the adverse event is possibly related to study procedures but one or more of the above criteria for "Definitely Related" are not met.
- Probably Not Related: The adverse event occurred while the patient was on the study but, in the opinion of the investigator, can reasonably be explained by the known characteristics of the patient's clinical state or other therapies.
- Definitely Not Related: The adverse event was definitely produced by the patient's clinical state or by other therapies and not by the study drug or study procedures.
- Uncertain Relationship: The adverse event does not meet any of the criteria previously outlined.

c. Expectedness:
An **Unexpected Adverse Event** is defined as an adverse event that is not listed in the investigator brochure or study protocol or is not listed at the specificity or severity that has been observed.

d. Severity:
The investigator will evaluate all AEs with respect to both seriousness (defined in 13.1.a. above) and **severity** (intensity or grade). AEs will be graded for severity according to the *DAIDS Table for Grading the Severity of Adult and Pediatric Adverse Events* (also known at the DAIDS AE Grading Table). For specific events that are not included in the DAIDS AE Grading Table, the generic scale listed below is to be used:

Generic AE Grading Scale

Grade 1	Events causing no or minimal interference with usual social and functional activities, and NOT raising a concern, and NOT requiring a medical intervention/ therapy.
Grade 2	Events causing greater than minimal interference with usual social and functional activities; some assistance may be needed; no or minimal medical intervention/therapy required.
Grade 3	Events causing inability to perform usual social and functional activities; some assistance usually required; medical intervention/therapy required.
Grade 4	Events causing inability to perform basic self-care functions; medical or operative intervention indicated to prevent permanent impairment, persistent disability, or death.
Grade 5	Events resulting in death.

An Adverse Event of Special Interest (AESI) will be defined as a pre-specified event of scientific or medical concern that has the potential of being related to study drug and is important to understand regardless of investigator classifications. AESIs included below will be recorded in the adverse event eCRF and will require a written narrative.

13.2 Protocol-specified exempt serious events (PSESEs)

Outcomes of acute respiratory infection, COVID-19, and critical illness will be systematically collected as Protocol-specified exempt serious events (PSESEs) for all patients.

PSESEs are exempt from adverse event reporting unless:

1. the event is determined to be Serious and Definitely or Possibly Related to the study drug or study procedures;
2. the event is determined to be Unexpected and Definitely or Possibly Related to the study drug or study procedures; or

This approach is taken to avoid creating an overly cumbersome safety oversight environment by identifying expected clinical outcomes as safety events and obscuring real safety signals. Even as they are exempted from expedited reporting requirements, PSESEs will be reviewed regularly (unblinded, by treatment arm) by the DSMB to maintain the integrity of safety monitoring for the trial. PSESEs that meet none of the criteria above will not be recorded or reported as AEs. PSESEs may occur during the initial hospitalization, lead to a re-admission, or occur in a later hospitalization during follow-up. The following are study-specific exempt serious events:

- Death (not Definitely or Possibly Related to the study drug or study procedures)
- Neurological Events:
    - Seizure

    - Stroke
- Cardiovascular Events:
    - Hypotension as defined by low arterial blood pressure leading to either [1] initiation or increase in vasopressor therapy, [2] administration of a fluid bolus of 500 ml or more, or [3] modification of the dose or discontinuation of the study drug.
    - Atrial or ventricular arrhythmia
    - Cardiomyopathy
    - Cardiac arrest
    - Myocardial injury
    - Acute coronary syndrome
    - Hypertension as defined by elevated arterial blood pressure leading to either [1] initiation or increase in antihypertensive medications or [2] discontinuation of the study drug
- Respiratory events:
    - Hypoxemia requiring supplemental oxygen
    - Acute respiratory distress syndrome
    - Receipt of non-invasive or invasive mechanical ventilation
    - Receipt of extra-corporeal membrane oxygenation
- Gastrointestinal events:
    - Elevation in aspartate aminotransferase or alanine aminotransferase
    - Acute pancreatitis
- Renal events:
    - Acute kidney injury
    - Receipt of new renal replacement therapy
- Endocrine events:
    - Symptomatic hypoglycemia
- Hematologic or coagulation events:
    - Neutropenia, lymphopenia, anemia, or thrombocytopenia
    - Venous thromboembolism
- Dermatologic events:
    - Severe dermatologic reaction (e.g., Steven's Johnson Syndrome)

A PSESE for "initiation of vasopressor therapy" should be recorded for patients who newly receive any vasopressor at a dose of at least 0.1 mcg/kg/min norepinephrine equivalents (see table). A PSESE for an "increase in vasopressor therapy" should be recorded for patients receiving a vasopressor at a dose of at least 0.1 mcg/kg/min norepinephrine equivalents who experience a doubling of vasopressor dose compared to either the dose at the time of randomization or the lowest dose in the prior 24 hours.

Drug	Dose	Norepinephrine equivalent
Epinephrine[a]	0.1 μg/kg/min	0.1 μg/kg/min
Norepinephrine[a]	0.1 μg/kg/min	0.1 μg/kg/min
Dopamine[a]	15 μg/kg/min	0.1 μg/kg/min
Phenylephrine[b]	1.0 μg/kg/min	0.1 μg/kg/min
Vasopressin	0.04 U/min	0.1 μg/kg/min

Note: Consistent with this approach, sites will evaluate a potential adverse event to determine whether it is a PSESE. If it is not a PSESE, it will be recorded and reported as an adverse event as outlined below. If the event is a PSESE, it will be evaluated for relatedness and expectedness. If the event is Serious and Definitely or Possibly Related, Unexpected and Definitely or Possibly Related it will be recorded as both a PSESE and an Adverse Event. If the PSESE does not meet either of these three criteria, then the event will be recorded as a PSESE in the PSESE eCRF as a study outcome. A study-specific clinical outcome may also qualify as a reportable adverse event. For example, a ventricular arrhythmia the investigator considers Serious and Definitely or Possibly Related to the study drug would be both recorded as a study-specific clinical outcome and reported as a Serious and Definitely or Possibly Related Adverse Event.

13.3 Monitoring and recording adverse events

The principal investigator at each study site has the responsibility for the safety of the individual participants under his or her care. For inpatients through day 28, on a daily basis the investigator or designee will determine if any adverse event has occurred. For each adverse event, the investigator will determine whether the adverse event was serious, whether it was definitely or possibly related to study drug or study procedures, whether it was unexpected, and of what severity it was.

The following categories of adverse events will be recorded as AEs in the Adverse Event case report form:

- Adverse Events that Qualify for Expedited Reporting:
  - Serious Unexpected, and Definitely or Possibly Related Adverse Events [also known as “Suspected Unexpected Serious Adverse Reactions” (SUSARs)]- adverse events that are considered by the investigator to be serious, unexpected, and definitely or possibly related to the study drug or study procedures
- Adverse Events that Qualify for Recording and Routine Reporting through Day 60
  - Non-SUSAR adverse events that are ‘Serious and Definitely or Possibly Related or of Uncertain Relationship’ OR that are “Unexpected and Definitely or Possible Related or of Uncertain Relationship”, regardless of whether they are PSESEs
  - Non-SUSAR adverse events that are Serious and are not PSESEs

- Non-SUSAR adverse events that are Definitely Related, Possibly Related, or of Uncertain Relationship and are not PSESEs
- Non-SUSAR adverse events that are Grade 3 or 4 Clinical AEs and are not PSESEs
- AESIs will include a narrative and will be reported in a routine manner

13.4 Reporting adverse events

This section describes the schedule for recording and reporting different types of safety events on eCRFs. In the care of study participants more information may be collected and recorded in the participant's medical record. The information collected in the medical record serves as source documentation of events (e.g., signs, symptoms, diagnoses) considered for reporting on eCRFs as part of protocol data collection.

13.4.1 Adverse Events that Qualify for Expedited Reporting

Adverse Events that qualify for Expedited Reporting include events that: are Serious, Unexpected and Definitely or Possibly Related (also known as Suspected Unexpected Serious Adverse Reactions, SUSARs. Adverse Events that qualify for Expedited Reporting (SUSARs) must be reported to the coordinating center by site investigators within 24 hours of site investigators becoming aware of the adverse event. The investigator at the study site or designee should inform the clinical coordinating center both by telephone and by official notification via completion of the Adverse Event case report form. The Medical Monitor may discuss with the site PI to determine if this event meets criteria for requiring Expedited Reporting (SUSAR). Events requiring Expedited Reporting (SUSARs)will be reported by the clinical coordinating center to the DSMB, sIRB, FDA and NHLBI within 7 calendar days of receipt of the report from the study site (as well as CA, IECs and other bodies per applicable local regulations for ex-U.S. sites). A copy of the Adverse Event case report form will be sent to the FDA, DSMB, sIRB, and NHLBI and appropriate regulatory bodies for ex-U.S. sites per applicable local regulations within 14 calendar days of receipt of the report from the study site. Adverse Events requiring Expedited Reporting (SUSARs) are followed until the outcome of the Adverse Event is known. If the outcome of an Adverse Events is still unknown at the time of the final follow-up visit, the outcome will be entered in the database as "unknown."

In parallel, non-U.S. participant SAEs should be reported to the NEAT ID Sponsor using the study specific Safety Event Reporting Form (SERF), within 24 hours of a member of the study team becoming aware of the event via e-mail to **Safety@rokcservices.com** or via the fax number provided on the SERF.

For non-U.S. participants, processing of relevant safety events, including expedited SAE reporting pertinent to those ex-US territories, including electronic reporting via EudraVigilance of suspected unexpected serious adverse reactions (SUSARs) via ICH E2B compliant safety reports (ICSRs/acknowledgements) will be in line with Directive 2001/20/EC Detailed guidance on the collection, verification and presentation of adverse event/reaction reports arising from clinical trials on medicinal products for human use (CT-3).

Adverse Events, that qualify for recording, but do not qualify for Expedited Reporting (i.e., adverse events that are not SUSARs), will be recorded on the Adverse Event eCRF through day 60. The DSMB will review all recorded adverse events during the scheduled meetings. The clinical coordinating center will distribute the written summary of the DSMB's review including the review of adverse events to the sIRB. If the DSMB determines the overall rate of adverse events is higher in

the intervention group than the control group, the coordinating center will notify the sIRB and the Food and Drug Administration within 14 calendar days of this determination.

All deaths are reported on the eCRF for deaths. Deaths considered **definitely or possibly related to the study intervention** (blinded investigational agent/placebo) must **also** be reported as an AE and may qualify for Expedited Reporting.

All deaths that are felt to be possibly or definitely related to study drug would be unexpected (for reporting purposes).

Unanticipated problems are findings discovered during the conduct of the trial that suggest participation in the trial may have more risk than was anticipated prior to the initiation of the trial. Unanticipated problems will also be reported within 14 days of the coordinating center learning about them to the DSMB and NHLBI.

13.4.2 Grade 3 and 4 Clinical Adverse Events

From Day 0 through Day 60, Clinical Adverse Events that are not PSESEs reaching Grade 3 or 4 severity level will be recorded on an eCRF. For a Clinical Adverse Event that was present at baseline, only those newly reaching Grade 3 or 4 will be recorded.

Clinical Adverse Events that are not PSESEs reaching Grade 3 or 4 severity level that occur between days 0 through 60 will be recorded on an eCRF at the time of phone follow-up. The date the event reaches the indicated grade will be collected to permit time-to-event analyses. These Clinical Adverse Events should be assessed for seriousness, relatedness, expectedness, severity (Section 13.3) and unanticipated problems. These events should be reported on the Adverse Event eCRF.

13.4.3 Pregnancy

The investigator or designee will collect pregnancy information on any female participants who become pregnant up to 30 days after receiving study drug unless indicated differently in the drug-specific appendix. The participant will be followed to determine the outcome of the pregnancy.

Figure 1. Adverse Event and Clinical Outcome Assessment, Recording, and Reporting

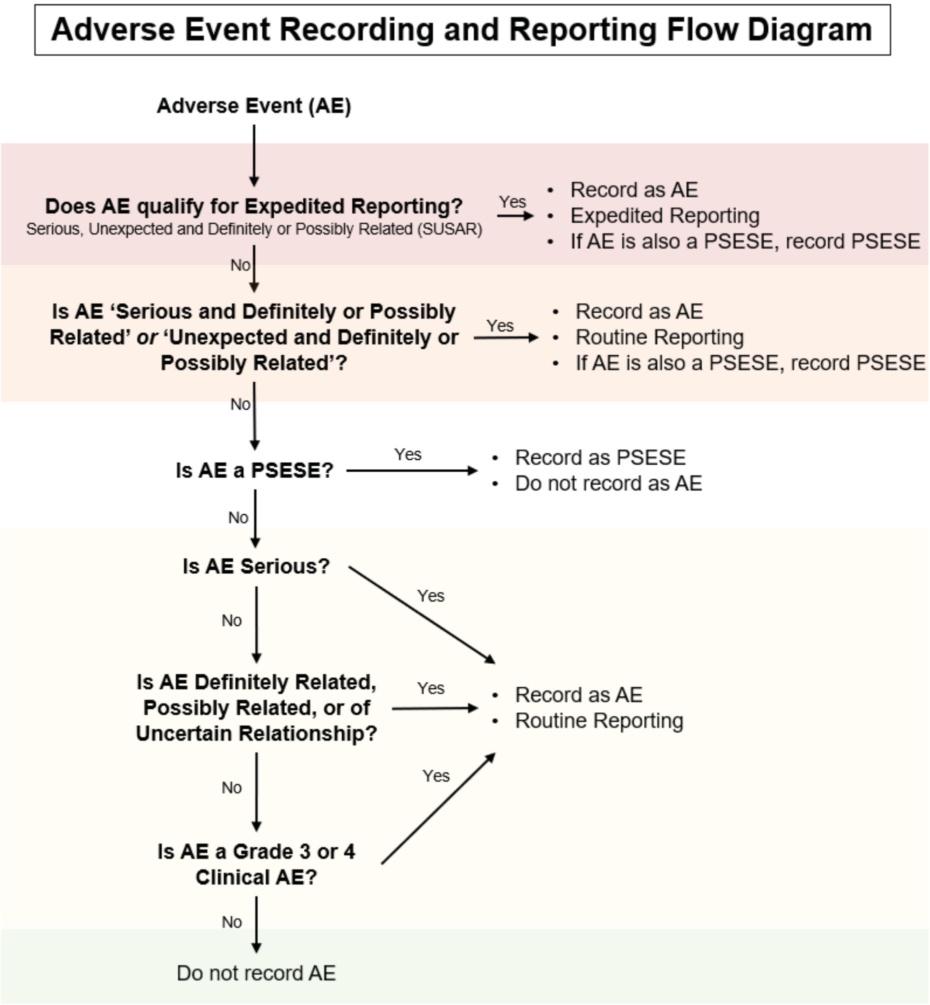

Recording of AEs – AEs that meet trial criteria for being recorded will be entered by site personnel into the AE eCRF in REDCap. Information will be provided on the attributes of each AE, including its seriousness, relatedness, expectedness, and severity.

Reporting of AEs – AEs that qualify for **Expedited Reporting** will be submitted to the Coordinating Center for review by the Medical Monitor and reporting to the DSMB, IRB, NHLBI, and FDA as outlined in the MOP. AEs that qualify for **Routine Reporting** will be reported using information in the eCRF to the DSMB at scheduled meetings, to the IRB at annual IRB review, and to the FDA as required.

13.5 Medical Monitor

An experienced medical monitor is assigned to the trial. The medical monitor will work with the site PI and study team to review Adverse Events that potentially require Expedited reporting and make an assessment of seriousness, relatedness, expectedness, and severity. The medical monitor will work with the Coordinating Center to prepare sponsor safety report and communicate as needed with the DSMB and the Investigational New Drug (IND) holder through the study safety office or other mechanism mutually agreed to and documented. An Urgent Safety Measure (USM) is a procedure which is not defined by the protocol that can be put in place to protect clinical trial participants from any immediate hazard to their health and safety. It is the responsibility of the investigator to apply the appropriate level of USM for the safety and protection of each participant in this study in order to prevent harm. USMs may be applied immediately without prior approval from the sponsor, competent authority (CA) or IRB/IEC. However, they must be reported to the sponsor and to the PI immediately (within 24 hours) who will then inform the CA and IRB/IEC according to local regulation. In addition to the expedited safety reporting mentioned above, the ex-US sponsor (NEAT-ID) will provide Development Safety Update Reports (DSURs) once a year throughout the clinical trial, or on request, to the CA and IRB/IEC. The report will be submitted within 60 days of the Developmental International Birth Date (DIBD) of the trial each year until the trial is declared ended or as per local regulation, if different.

The medical monitor will remain blinded in all discussions with the study team regarding expedited reporting and recording to perform an unbiased assessment of seriousness, relatedness, expectedness, and severity. In the event the monitor and clinical team feel that unblinding is needed for safety purposes the monitor will communicate this with the Coordinating Center for documentation purposes who will confer treating assignment to the medical monitor. When this occurs, an alternate medical monitor will be utilized to review the AE with the study team to determine seriousness, severity, and relatedness.

The medical monitor or the DSMB may request enrollment be halted for safety reasons (e.g., unacceptably high rate of Serious Adverse Events). If the treatment arm is temporarily halted or stopped for safety reasons, IRBs/ethics committees will be informed. The IND holder(s) and sponsor(s), in collaboration with the protocol chair and the DSMB, will determine if it is safe to resume the study. The sponsor will notify the Site Investigators of this decision. The conditions for resumption of the study will be defined in this notification. The Site Investigators will notify their local IRBs/ethics committees of the decision to resume the study.

14 Risk Assessment

14.1 Potential risks of other study procedures

See agent-specific appendices for agent-specific safety risks.

14.2 Minimization of risk

Federal regulations at 45 CFR 46.111(a)(1) require that risks to participants are minimized by using procedures which are consistent with sound research design. This trial protocol incorporates numerous design elements to minimize risk to patients that meet this human subject protection requirement.

14.3 Potential benefit

Study participants may or may not receive any direct benefits from their participation in this study. Administration of these agents may improve clinical outcomes among adults hospitalized for COVID-19 infection.

14.4 Risk in relation to anticipated benefit

Federal regulations at 45 CFR 46.111 (a)(2) require "the risks to subjects are reasonable in relation to anticipated benefits, if any, to subjects, and the importance of the knowledge that may reasonably be expected to result." Based on the preceding assessment of risks and potential benefits, the risks to subjects are reasonable in relation to anticipated benefits All agents have an acceptable safety profile and have been or are currently being studied in Phase 2 trials of patients with COVID-19. As new agents are added, any change in risks in relation to anticipated benefit will be described in the agent-specific appendix.

15 Data and Safety Monitoring Board (DSMB)

The role of the DSMB is to monitor patient safety and integrity of the trial. The full details of the DSMB will be provided separately by NHLBI in the DSMB charter. We outline the role of the DSMB here. The independent DSMB will be comprised of individuals with appropriate expertise such as clinician scientists in critical care, emergency medicine, pulmonology, nephrology, cardiology, trial design, biostatistics, and ethics. The DSMB will review reports. Any post-randomization or outcomes data presented by treatment group in reports will be prepared by unmasked statisticians not otherwise involved with trial conduct or design decisions, who will conceal such information from the investigator team. These unmasked statisticians will compute the efficacy and inferiority criteria at regular intervals as described previously. The DSMB chairperson will be alerted to any decision threshold for stopping being met. Beyond assessing fidelity to pre-specified adaptations, the principal role of the DSMB is to assure the safety of participants in this trial. They will regularly monitor data from this trial, review and assess the performance of its operations, and make recommendations with respect to aspects of trial conduct such as:

- Adverse events
- Evidence of efficacy or adverse events
- New external information, early attainment of study objectives, safety concerns
- Possible modifications in the clinical trial protocol
- Inadequate performance of the trial overall

16 Data Handling and Record Keeping

16.1 Data Collection and Management Responsibilities

Data collection is the responsibility of the delegated clinical trial staff at the site under the supervision of the site investigator. The investigator is responsible for ensuring the accuracy, completeness, legibility, and timeliness of the data reported.

All source documents should be completed in a neat, legible manner to ensure accurate interpretation of data.

Source document worksheets for recording data for each participant enrolled in the study will be provided as needed. Data recorded in the eCRF derived from source documents should be consistent with the data recorded on the source documents.

Clinical data (including AEs, concomitant medications, and expected adverse reactions data) and clinical laboratory data will be entered into secure, compliant data capture systems provided by the

Data Coordinating Center. The data system includes password protection and internal quality checks, such as automatic range checks, to identify data that appear inconsistent, incomplete, or inaccurate.

16.2 Study Records Retention

Per FDA regulation 312.62C, study documents should be retained for a minimum of 2 years after the last approval of a marketing application in an International Conference on Harmonization (ICH) region and until there are no pending or contemplated marketing applications in an ICH region or until at least 2 years have elapsed since the formal discontinuation of clinical development of the study intervention. Study documents may be retained for a longer period, however, if required by local regulations. No records will be destroyed without the written consent of the sponsor, if applicable. It is the responsibility of the sponsor to inform the investigator when these documents no longer need to be retained.

16.3 Protocol Deviations

A protocol deviation is any noncompliance with the clinical trial protocol and GCP requirements. The noncompliance may be either on the part of the participant, the investigator, or the study site staff. As a result of deviations, corrective actions are to be developed by the site and implemented promptly.

It is the responsibility of the site PI/study staff to use continuous vigilance to identify and report deviations.

Protocol deviations must be reported to the sIRB per their guidelines. The site PI/study staff is responsible for knowing and adhering to requirements for reporting protocol deviations to the study coordinating center and sIRB, and these details will be included in the platform MOP. Where applicable in ex-US territories, guidelines for reporting serious breach of protocol and GCP to the appropriate regulatory bodies will be followed.

16.4 21 CFR Part 11 Compliance

16.4.1 Record locking

This trial will utilize the REDCap record-locking feature, that will allow the users to lock a participant record. A complete audit trail of the locked records will be kept in the data instrument's history log. Users will receive a prompt when locking an entire record. They will be asked to review a PDF copy of the entire record to confirm it is the correct record and/or file. Once the PDF has been reviewed and confirmed, the record will be locked. All records that are locked using record-level locking will have a duplicate copy of the Portable Document Format (PDF) file automatically stored on a secure file server outside of REDCap. The record-locking feature complies with all the requirements described in 21 CFR Part 11 for FDA trials and has been validated at VUMC.

16.4.2 eConsent

Informed consent should follow both local institutional policy and national regulatory guidance. One option in this trial is to utilize the REDCap eConsent feature. VUMC has developed an electronic consent (eConsent) framework within the REDCap platform allowing research participants to rapidly review and sign consent documentation for delivery via web, tablet, or smartphone. Electronic consent forms can leverage standard REDCap survey features including multi-lingual language capacity for information rendering and capture. Upon completion, the system documents the

‘consent transaction’ and stores final, “frozen” consent PDFs in REDCap, allowing researchers to retrieve information on the consent type, status, and version at any time. Consents are also stored in a separate secure document system for redundancy and permanent archival. The REDCap eConsent framework has been 21 CFR Part 11 validated at VUMC.

17 End of Trial

The “end of the trial” occurs when patient enrolment, follow-up, data cleaning and analysis have been completed.

18 Study Finances

18.1 Funding Source

Support for studies conducted under the protocol includes funding from the National Institutes of Health.

18.2 Costs to the Participant

In sites and countries where health insurance is applicable, participant health insurance may be billed for the costs of medical care and activities occurring outside this protocol. If their insurance does not cover these costs or participants do not have insurance, these costs will be participant responsibility.

Activities of the sub studies may take advantage of standard of care activities for collecting information, such as at routine follow-up visits. Such visits will generally be charged to insurance unless the visit is required only for research. At mixed visits where both research and clinical care occur such as for inpatients enrolled in this trial, clinical care will generally be charged to insurance.

19 Appendix A: Primary study outcomes

19.1 Approach to ascertainment and verification of outcomes

Outcomes will be assessed locally and will not be centrally adjudicated unless specified in the arm-specific appendix. Outcomes should be assessed by a local investigator or other qualified study team member who is blinded to treatment assignment.

19.2 Primary outcome: Oxygen free days

The primary outcome for this platform is oxygen free days through day 28 (OFD). OFD is a clinically relevant, longitudinal measure of lung function and mortality for the first 28 days after randomization. OFD will be calculated using principles developed during the past 20 years for other free-day clinical trial outcomes, including ventilator free days,[32,33] organ support free days,[34] and hospital free days.[35] Free-day outcomes have also been successfully utilized in COVID-19 clinical trials.[36]

OFD will be calculated as the number of calendar days during the first 28 days after randomization during which the patient was alive and not receiving supplemental oxygen by nasal cannula, face mask, high flow nasal cannula (HFNC), non-invasive ventilation (NIV), invasive mechanical ventilation (IMV), or extracorporeal membrane oxygenation (ECMO). The day of randomization is denoted as Day 0. Starting with calendar day 1 (the day after randomization) and continuing for 28 days, study personnel will document whether supplemental oxygen therapy was received for any duration. While the patient is in the hospital, the highest level of respiratory support will be classified daily according to the 8-category WHO COVID-19 clinical status scale (Table 3).[48] Calendar days on which the patient received supplemental oxygen (category 4), HFNC or NIV (category 5), or IMV or ECMO (categories 6 and 7) will be classified as a day with oxygen use.

After hospital discharge, patients will be assessed for home oxygen use via serial telephone follow-up calls to the patient or surrogate. During these calls, study personnel will assess for new home oxygen use with the following questions:

(1) Were you discharged from the hospital on oxygen?
(2) Did you use oxygen at any time after hospital discharge?
(3) Are you still using oxygen?
(4) If you received oxygen at any time after hospital discharge and are no longer on oxygen, what was the last day you used oxygen at home?

Patients who chronically used supplemental oxygen prior to their COVID-19 illness will be considered oxygen free when they return to the same level of oxygen support, they had been using prior to COVID-19 illness. For example, a patient who chronically used supplemental oxygen at 4 liters per minute via nasal cannula before COVID-19 and who was intubated for acute management of COVID-19 would be considered oxygen free for calculation of the primary outcome when he/she returned to oxygen support via nasal cannula at 4 liters per minute or less.

Data collected reflecting the patient's status after day 28 will not be used for the calculation of OFD. OFD will be calculated as 28 minus the number of days with supplemental oxygen use during the first 28 days following randomization. OFD will be coded as -1 for patients who died before study day 28. Hence, the range for OFD is from -1 to 28 days. Examples of OFDs are shown in Table 4. Some patients will enter the trial with supplemental oxygen use (enrolled while in WHO category 4, 5, 6 or 7), while others will enter the trial without oxygen therapy (enrolled while in WHO category 3).

Table 3. WHO COVID-19 Clinical Status Scale and its use for enrollment eligibility and calculation of oxygen free days (OFD)

Category	Category Description	Notes for eligibility (baseline status)	Notes for OFD calculation (daily status on days 1 – 28)
1	Not hospitalized without limitation in daily activity	Patients in category 1 at baseline are not eligible for enrollment.	Classified as oxygen free day
2	Not hospitalized with limitation in daily activity or home oxygen use	Patients in category 2 at baseline are not eligible for enrollment.	Days with home oxygen use are classified as days with supplemental oxygen. Days at home with limitations in daily activity but with no home oxygen use are classified as oxygen free days.
3	Hospitalized not on supplemental oxygen	Patients in category 3 at baseline are not eligible for enrollment.	Classified as oxygen free day after enrollment
4	Hospitalized on standard supplemental oxygen via nasal cannula or mask	Eligible for enrollment	Classified as day with supplemental oxygen use
5	Hospitalized on high-flow nasal cannula or non-invasive ventilation	Eligible for enrollment	Classified as day with supplemental oxygen use
6	Hospitalized on invasive mechanical ventilation without other organ support	Eligible for enrollment	Classified as day with supplemental oxygen use
7	Hospitalized on invasive mechanical ventilation and other organ support (including vasopressors, RRT or ECMO)	Eligible for enrollment	Classified as day with supplemental oxygen use
8	Death	Patients who die before randomization are not eligible for enrollment	Death at any time prior to the earlier of hospital discharge or day 28 is coded as -1 OFD

The baseline (pre-randomization) clinical status will be used to determine eligibility for enrollment. Clinical status will be scored every day the patient is in the hospital through day 28; these daily scores will be used to calculate OFD.

Table 4. Descriptions of OFD Data

	OFD (days)	Description
More Severe ↑	-1	Patient died before the end of day 28.
	0	Patient survived through day 28 and had oxygen use on every calendar day between day 1 and day 28.
	1	Patient survived through day 28 and was free from oxygen use for 1 calendar day in the first 28 days following randomization. The patient was on nasal cannula, face mask, HFNC, NIV, IMV or ECMO on 27 of the first 28 calendar days following randomization.
	10	Patient survived through day 28 and was free from oxygen use for 10 days in the first 28 days following randomization. The patient was on nasal cannula, face mask, HFNC, NIV, IMV or ECMO on 18 of the first 28 calendar days following randomization.
	25	Patient survived through day 28 and was free from oxygen use for 25 days in the first 28 days following randomization. The patient was on nasal cannula, face mask, HFNC, NIV, IMV or ECMO on 3 of the first 28 calendar days following randomization.
↓ ***Less Severe***	28	Patient survived through day 28 and was free from oxygen use on every calendar day after the day of randomization (Day 0) for the first 28 days of follow-up. The patient did not receive oxygen by nasal cannula, face mask, HFNC, NIV, IMV, or ECMO at any time between day 1 and day 28.

19.3 Definitions

19.3.1 ICU Level of care
Defined as planned admission to ICU.

19.3.2 Myocardial injury
Myocardial injury will be defined as an increase in troponin above the 99th percentile with or without ECG changes consistent with ischemia. This diagnosis is made locally.

19.3.3 Acute Kidney Injury
Acute kidney injury after enrollment is defined by KDIGO criteria for Acute Kidney Injury in the setting of not meeting these criteria upon enrollment. We will define AKI as Stage 2 or higher for purposes of our AKI outcome:

THREE STAGES:

- Stage 1: Serum Cr 1.5–1.9 times baseline, OR ≥ 0.3 mg/dl increase in serum Cr
- Stage 2: Serum Cr 2.0–2.9 times baseline
- Stage 3: Serum Cr ≥ 3.0 times baseline OR Increase in serum creatinine to ≥ 4.0mg/dl, OR Initiation of renal replacement therapy

19.3.4 Disseminated Intravascular Coagulation (DIC) (Overt) – DIC score ≥ 5

1. Platelet count ≥ 100 K (0); 50–100K (1 point); < 50K (2 points)

2. Elevated D-dimer: no increase (0 points); moderate increase (1 point); severe increase (3 points) according to local criteria.
3. Prolonged PT < 3 seconds (0 points); 3–6 seconds (1 point); ≥ 6 seconds (2 points)
4. Fibrinogen level ≥ 100 (0 points); < 100 (1 point) mg/dL

19.3.5 ISTH Defined Major Bleeding

Bleeding that:

1. Resulted in death,
2. Occurred in a critical organ (intracranial, intraspinal, intraocular, retroperitoneal, intraarticular, intramuscular with compartment syndrome, or pericardial), or
3. Associated with either a decrease in the hemoglobin level of at least 2 g per deciliter or a transfusion of at least 2 units of packed red cells

20 Appendix B: Data and Safety Monitoring Plan

20.1 Overview

The purpose of a monitoring plan is to facilitate compliance with good clinical practice guidelines and federal regulations by: documenting a plan for verifying that the rights and well-being of participants are protected; the reported trial data are accurate, complete and verifiable from source documents; the confidentiality of participant data is maintained; serious adverse events and unanticipated problems are adequately addressed; and the trial is conducted in compliance with the protocol, prevailing SOPs, federal regulations, and other relevant requirements. The full Data and Safety Monitoring plan is described in detail in a separate document. The scope and content of this monitoring plan is based on the objective, purpose, design, and complexity of this platform, which is a multi-arm, blinded, randomized placebo-controlled trial. The safety profile of the selected agents being considered for this platform is based on prior clinical trials in patients with acute illness, both COVID-19 and non-COVID-19 related. Thus, a monitoring approach of surveillance and reporting of serious adverse events is sufficient. The trial is designed to enroll subjects at multiple sites, to include within-site randomization. A comprehensive project and data management system is in place to support real-time review of regulatory compliance, screening, enrollment, and data integrity with automated reporting to the study team. A risk management plan will also be deployed. Intensive patient monitoring in the clinical setting during and immediately following treatment is planned. These features mitigate risks from conduct of the trial and suggest verification of consent, eligibility, and primary outcomes with targeted verification of other data are sufficient to ensure study integrity and protection of the rights and welfare of participants. The data and safety monitoring plan will be approved by the DSMB prior to enrolling patients in this trial. The details of the scope of monitoring, monitoring personnel, site visits (remote and in-person) are delineated in the separate DSMP document.

21 Appendix C: Minimum Biological Specimen collection

Baseline specimens to be collected
Sample Processing
Biorepository

Study-specific biospecimen collection for exploratory outcomes will be collected, when possible, at participating U.S. Sites.

Blood collection times (4 total timepoints):

- Baseline—at time of randomization (Study Day 0).
- Two time points on study drug (Study Day 1 ± 1 day and Study Day 3 ± 1 day)
- Within 2-36 hours after inpatient study treatment ends (or any time on Study Day 5 + 36 hours for final blood collection in the Fostamatinib arm for those still hospitalized at Day 5)

Standard samples to be collected & volumes at each time point:

- Plasma – 30.4 mL
- Serum – 6 mL
- Total blood collection at each time point = 36.4 mL
    -

Peptides/enzymes to be measured at each time point:

- 1st priority:
    - Ang-(1-7), Ang II, NT-proBNP and hsTn, (plasma, collected in pretreated tubes)
    - ACE, ACEII activity and level, (serum)
- 2nd priority:
    - Renin, Ang I, Neprilysin, Prolyl oligopeptidase

Supplies:

- Source – The University of Vermont: packages sent to sites with EDTA plasma tubes (inhibitor cocktail) including labels and special tubes
- Sites – Batch shipping (overnight on dry ice)

Note 1: *We anticipate that some sites may not be able to collect & process all the samples and time points listed above. We plan to work with those sites to identify more limited time points and/or discarded samples that could be collected, processed, and sent to the biorepository.*

Note 2: *If a participant's blood specimens are unable to be obtained at the timepoints outlined in the protocol due to unforeseen circumstances such as a non-functioning IV, or the patients desire to not have blood drawn, this will not be considered a protocol violation.*

22 Appendix D: Arm 3: Fostamatinib

22.1 Description of active therapy

Fostamatinib is an oral spleen tyrosine kinase inhibitor FDA approved for the treatment of adult patients with chronic immune thrombocytopenia (ITP) who have had insufficient response to previous treatment. Fostamatinib is a prodrug with an active metabolite, R406. R406 is protein bound and has a half-life of 15 hours, with 80% of excretion occurring in feces and 20% excreted in urine.

22.2 Rationale for evaluating Fostamatinib

Spleen tyrosine kinase is a cytoplasmic tyrosine kinase that signals through Fc receptors, B-cell receptors, and c-type lectin receptors. Robust antibody responses are associated with severe disease in COVID-19 and may drive thromboinflammation. *In vitro* evidence suggests, R406, the active component of fostamatinib, can inhibit the release of cytokines by macrophages and platelet-mediated thrombosis provoked by SARS-CoV-2 specific spike antigen/antibody complexes. R406 also inhibits the release of neutrophil extracellular traps from neutrophils stimulated with plasma from patients with COVID-19, ultimately resulting in decreasing immunothrombosis. A recent placebo-controlled randomized phase 2 study in hospitalized adults with Covid-19 (NCT04579393) suggested fostamatinib in addition to usual care was safe and did not result in more serious adverse events (10.5% in the fostamatinib group vs. 22% in the placebo group). Additionally, multiple secondary efficacy endpoints showed trends favoring the patients receiving fostamatinib, including 28-day mortality, days free of oxygen, and recovery as measured on the 8-point ordinal scale at day 15.

22.3 Fostamatinib dose, duration, and route of administration

We will use a study dose of 150mg orally twice daily for 14 days (28 doses). This regimen was studied in the recently completed phase 2 trial in patients hospitalized with COVID-19. The dose may be modified to 100mg according to pre-defined criteria in section 22.7 (Dose Modification Considerations and Medication Interactions). Study medication will be continued as an outpatient if the patient is discharged prior to completing 28 doses. If necessary, in patients unable to swallow, tablets can be crushed until granular with an approximate particle size <2 mm, added to approximately 10 mL of water, and stirred to mix before administration through an enteral tube.

While many patients on BiPAP can safely receive oral medications, it may be potentially unsafe to administer oral medications to patients at certain times while they are receiving BiPAP therapy due to risk of hypoxemia if the mask is removed and/or risk of aspiration if an oral medication is administered and BiPAP is immediately re-initiated. Determining whether an oral medication should be given to a patient receiving BiPAP therapy is a bedside clinical decision.
The goal is to administer two doses of study medication each calendar day and approximately 12 hours apart from one another. If the time between study medication doses needs to be reduced, the recommendation is to administer two doses at least 4 hours apart from one another. If a dose of study medication is entirely skipped, a missed dose should be noted in the electronic data capture system (REDCap). Skipped doses are not “made up” by extending the dosing period; patients should only receive study drug for a maximum of 14 days, starting with randomization.

22.4 Placebo

The drug product is provided by Rigel Pharmaceuticals, Inc., and consists of 2 strengths of orange film-coated, plain, bioconvex tablets. The 150 mg tablet is oval, and the 100 mg tablet is round. The tablets are supplied in white opaque high-density polyethylene bottles capped with white polypropylene child resistant closures with foil induction seals. Placebo tablets to match fostamatinib 100 mg and 150 mg will be provided by Rigel Pharmaceuticals, Inc. An unblinded pharmacist at each study site will dispense and record randomization and treatment assignment.

22.5 Fostamatinib-specific safety considerations and potential medication interactions

Fostamatinib has a consistent safety profile, with the most common adverse reactions (≥5% and more than placebo) being diarrhea, hypertension, nausea, respiratory infection, dizziness, ALT/AST increase, rash, abdominal pain, fatigue, chest pain and neutropenia. Warnings and precautions included in the US product label include hypertension, elevated liver function tests, diarrhea, neutropenia, and embryo-fetal toxicity.

- Fostamatinib must be discontinued in case the patient becomes pregnant.

- Concomitant use with strong CYP3A4 inhibitors with fostamatinib will increase exposure to the active metabolite R406 and may result in increased risk of adverse reactions. **Therefore, *patients that cannot have strong CYP3A4 inhibitors held should be excluded from the Fostamatinib arm*.** Strong CYP3A4 inhibitors that should exclude patients from the fostamatinib arm include: Atazanavir, Certinib, Clarithromycin, Cobicistat and cobicistat-containing coformulations, Idelalisib, Indinavir, Itraconazole, Ketocanazole, Levoketoconazole, Lonafarnib, Lopinavir, Mifepristone, Mibefradil, Nefazodone, Nelfinavir, Ombitasvir-paritaprevir-ritonavir plus dasabuvir, Posaconazole, Ribociclib, Ritonavir, Saquinavir, Telithromycin, Troleandomycin, Tucatinib, Voriconazole. Novel strong CYP3A4

inhibitors may be released after the writing of this protocol appendix. If you anticipate the patient will require a strong CYP3A4 inhibitor that is not included in the list above, please reference this regularly updated list: https://drug-interactions.medicine.iu.edu/MainTable.aspx

Fostamatinib itself is a weak CYP3A4 inhibitor and will result in a modest (~1.25 fold) increase in CYP3A4 substrates. Medications processed (activated or deactivated) by the CYP3A4 enzyme are here referred to as CYP3A4 substrate medications. Concomitant use of CYP3A4 substrate medications with Fostamatinib will modestly decrease the processing of the CYP3A4 substrate medication. If possible, CYP3A4 substrate medications should be held or have another medication substituted for them. Due to its modest effects, concomitant use of CYP3A4 substrate medications is not an exclusion criterion for this trial. However, investigators should carefully consider inclusion of patients taking CYP3A4 substrate medications with narrow therapeutic windows and should monitor closely for toxicities.

- For a full list of CYP3A4 substrates, please reference this regularly updated list: https://drug-interactions.medicine.iu.edu/MainTable.aspx.
- All participants who are randomized to this arm should refrain from drinking alcohol while taking study drug.
- Once discharged, WOCBP must agree to use effective contraception to not get pregnant between the first dose of study drug and 30 days after the last day of study drug.

  Effective contraception is defined as:
  - A tubal ligation: for many teratogenic (and mutagenic) products, tubal ligation is considered a form of contraception, not a form of surgical sterilization (e.g., hysterectomy, bilateral oophorectomy).
  - An approved hormonal contraceptive such as oral contraceptives, emergency contraception used as directed, patches, implants, injections, rings, or hormonally impregnated intrauterine device (IUD).

  Less effective contraception is defined as:
  - A form of less effective contraception is defined as Barrier methods (such as a condom used with or without a spermicide or a diaphragm or cervical cap used with a spermicide).

22.6 Fostamatinib Arm-Specific Exclusion Criteria

The following **exclusion criteria** differ from the master protocol criteria:

1. Randomized in another trial evaluating fostamatinib in the prior 30 days

Study arm exclusion criteria measured within 24 hours prior to randomization:

1. AST or ALT ≥ 5 × upper limit of normal (ULN) or ALT or AST ≥ 3 × ULN and total bilirubin ≥ 2 × ULN
2. SBP > 160 mmHg or DBP > 100 mmHg at the time of screening and randomization
3. ANC < 1000/mL
4. Patient is anticipated to require a strong CYP3A inhibitor (Atazanavir, Certinib, Clarithromycin, Cobicistat and cobicistat-containing coformulations, Idelalisib,Indinavir, Itraconazole,

Ketoconazole, Levoketoconazole, Lonafarnib, Lopinavir, Mifeprostone, Mibefradil, Nefazodone, Nelfinavir, Ombitasvir-paritaprevir-ritonavir plus dasabuvir, Posaconazole, Ribociclib Ritonavir, Saquinavir, Telithromycin, Troleandomycin, Tucatinib, Voriconazole) from randomization to 21 days post-randomization. For a full list of CYP3A4 substrates, please reference this regularly updated list: https://drug-interactions.medicine.iu.edu/MainTable.aspx.

5. Patient unable to participate or declines participation in the fostamatinib arm.

22.7 Dose Modification Considerations and Medication Interactions

Dose Modifications: The following dose modifications will be utilized in patients randomized to the fostamatinib arm (active or placebo). Those patients who have doses held will only complete 14 days (up to 28 doses) of study treatment. Dosing will not extend beyond day 14. Blood pressure will be monitored daily in the hospital while on study drug up until the time of discharge.

Hypertension:
Blood pressure >140/90 should be treated with antihypertensives per usual care.

During the inpatient hospitalization: If systolic BP remains > 160 mmHg or diastolic BP > 100 mmHg or higher despite antihypertensive therapy, interrupt study drug. When restarting study drug once BP is below 160 mmHg systolic and 100 mmHg diastolic, drug dose should be reduced to 100 mg twice daily or matching placebo for the remainder of the study.

- **At the time of Hospital discharge:** Patients **still on study drug** at the time of hospital discharge will have the following study procedures performed (see Figure 2):

Figure 2. Schematic overview of blood pressure considerations and procedures performed for patients still on study drug at the time of hospital discharge

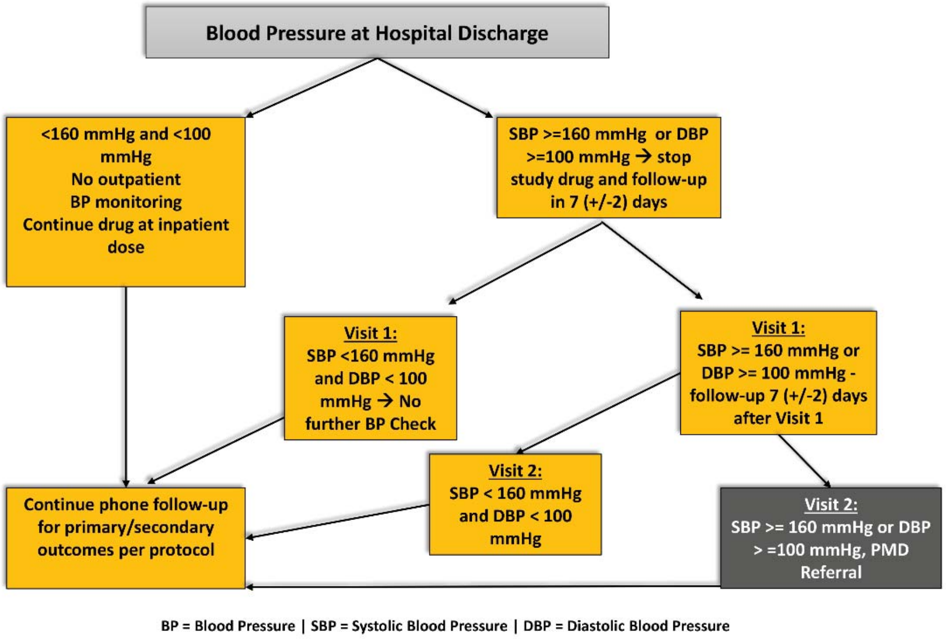

1) Those with SBP < 160 mmHg and DBP < 100 mmHg will continue on study drug at the inpatient dose and have no further BP measurements for study purposes
2) Those with SBP ≥ 160 mmHg or DBP ≥ 100 mmHg at the time of hospital discharge will have study medication stopped and undergo the following repeat testing:

1) 7 (+/-2) days after post-hospital discharge a repeat blood pressure will be obtained.
    a. If the SBP is < 160 mmHg and DBP is < 100 mmHg no further BP monitoring will be performed for study purposes
    b. If the SBP is ≥ 160 mmHg or the DBP is ≥ 100 mmHg a follow-up will be performed in 7 (+/-2) days after the prior visit
        i. At the repeat visit if the SBP is < 160 mmHg and DBP is < 100 mmHg no further BP monitoring will be performed for study purposes
        ii. At the repeat visit if the SBP is ≥ 160 mmHg or DBP is ≥ 100 mmHg study guidance will recommend BP recheck by a healthcare provider

Hepatotoxicity:
Inpatient monitoring: Liver function tests (LFT's) will be checked daily during hospitalization while on study drug.

1) Study drug should be stopped if there is an increase in either:
    a. AST or ALT greater than or equal to 5-times the upper limit of normal at the local lab or for those with elevated AST and ALT at the time of enrollment an increase to 5-times the level at enrollment
    b. AST or ALT to greater than or equal to 3-times the upper limit of normal at the local lab AND total bilirubin to greater than or equal to 2-times the upper limit of normal at the local lab.

Elevated unconjugated (indirect) bilirubin in absence of other LFT abnormalities – continue study drug with frequent monitoring since isolated increase in unconjugated (indirect) bilirubin may be due to UGT1A1 inhibition.

Outpatient monitoring: Patients discharged while on study drug with AST/ALT or bilirubin elevated > 2x the upper reference limits or up trending from most recent value (but less than the inpatient stopping criteria) AND patients discharged not on study drug with either AST or ALT at least 3-times the upper limit of normal or total bilirubin at least 2-times the upper limit of normal at the time of discharge will have the following study procedures performed:

1) 7 (+/-2) days post-hospital discharge a repeat measurement of AST, ALT and total bilirubin will be obtained.
    a. Patients NOT on study drug at the time of repeat LFT measurement:
    *If these repeat measurements are less than 3-times the upper limit of normal for AST or ALT and less than 2-times the upper limit of normal for total bilirubin, then no further study procedures will be performed related to LFT monitoring.
    *If these repeat measurements are greater than 3-times the upper limit of normal for AST or ALT or greater than 2-times the upper limit of normal for total bilirubin, then a repeat measurement of AST, ALT and total bilirubin should be obtained 7 (+/-2) days later. If the AST or ALT remain above 3-times the upper limit of normal or total bilirubin is above 2-times

the upper limit of normal after this repeat testing 7 days later, LFTs should be monitored per standard of care by a provider or if no provider followed-up by the study team.

b. Patients ON study drug at the time of repeat LFT measurement:

*If these repeat LFT measurements are below the upper reference limit of normal continue study drug to completion and no recheck is needed.

*If these measurements remain above the upper reference limit of normal for either AST, ALT or total bilirubin but do not meet inpatient stopping criteria (defined above), continue study drug and a repeat measurement of AST, ALT and total bilirubin should be obtained 7 (+/-2) days later. If this repeat testing remains above the upper limit of normal for AST, ALT or bilirubin it should be monitored per standard care by a provider or if no provider followed-up by the study team.

*If study drug is stopped (per inpatient stopping rules above) repeat measurement of AST, ALT and total bilirubin should be obtained 7 (+/-2) days later. If this repeat testing remains above the upper limit of normal for AST, ALT or bilirubin it should be monitored per standard care by a provider or if no provider followed-up by the study team.

Once study drug is stopped for abnormal LFT values, the study drug will not be restarted.

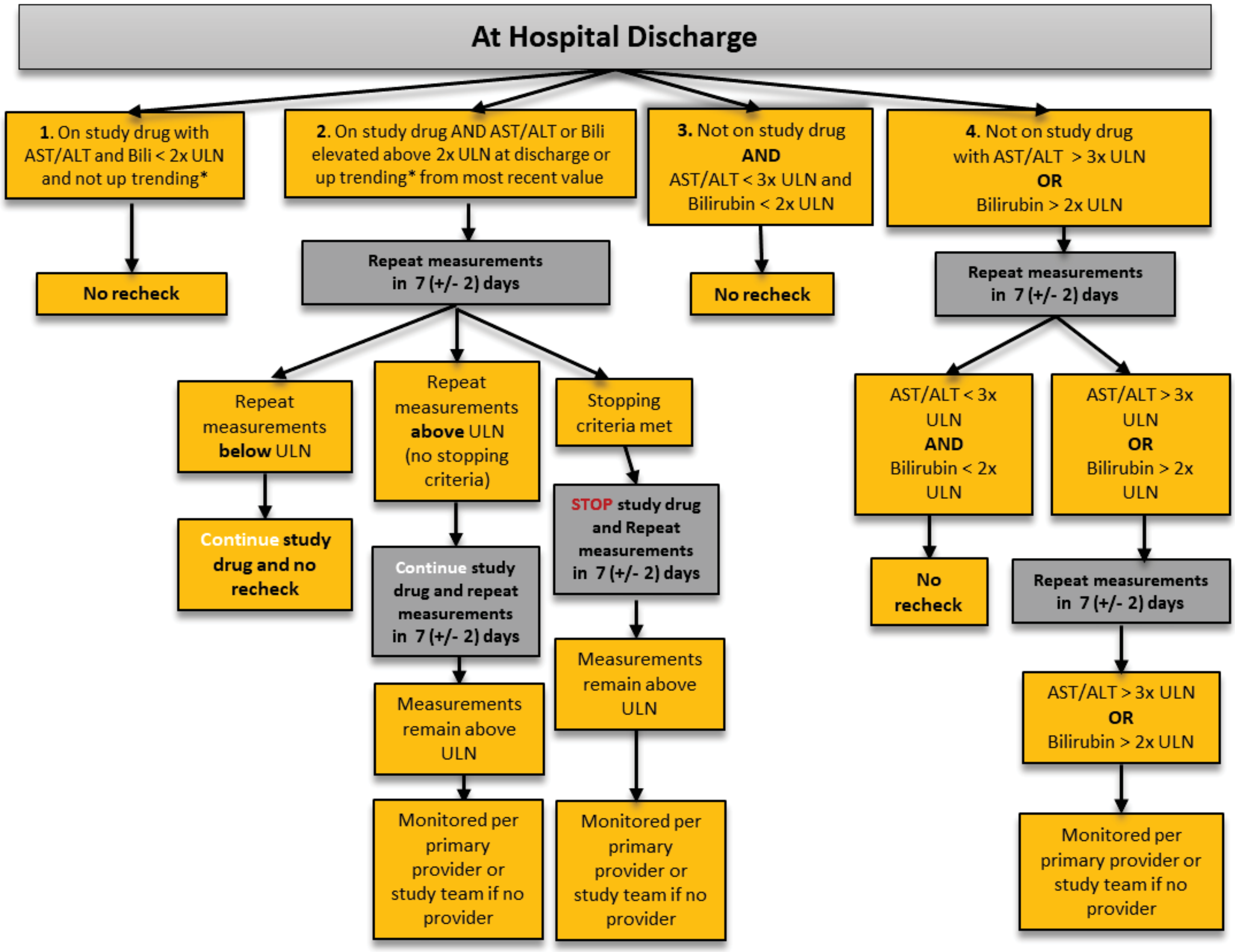

*up trending is defined as a >25% increase in LFTs from the day prior

Diarrhea: If symptoms become severe (grade 3 or above) while the patient is hospitalized, temporarily stop study drug until symptoms resolve to mild (grade 1). When restarting, study drug dose should be reduced to 100 mg twice daily or matching placebo for the remainder of the study. Study drug should be stopped if diarrhea becomes severe in the outpatient setting.

Neutropenia:
If the absolute neutrophil count (ANC) decreases to less than 1.0x10^9/L, the study drug should be discontinued.

- If the ANC returns to above 1.0x10^9/L, the study drug may be restarted. When restarting the study drug, the dose should be reduced to 100 mg twice daily or matching placebo for the remainder of the study.
- In patients who restart fostamatinib at 100 mg BID after neutropenia resolves within the 14-day window, and labs are not rechecked during hospitalization, the team must either: 1) repeat CBC with differential within 7 days of discharge OR 2) not restart the medication if they are unable to repeat CBC with differential in this 7-day time frame.

- A CBC with differential will be checked daily while the patient is in the hospital and on study drug. If the patient is discharged with an ANC < 1.0x10^9/L then a repeat CBC with differential will be performed within 7 days of hospital discharge and repeated weekly for 4 weeks or until the ANC is >1.0x10^9/L.
- If a patient is found to have an ANC <1.0x10^9/L as part of routine care after hospital discharge while still in the follow-up period, then a CBC with differential should be checked weekly for 4 weeks or until the ANC is >1.0x10^9/L.
- Study drug is not restarted when the ANC remains <1.0x10^9/L Those who are discharged with a normal ANC require no further measurement after discharge.

Medication Interactions:
Patients will be monitored after randomization to ensure the following medications are not started during study treatment: Atazanavir, Certinib, Clarithromycin, Cobicistat and cobicistat-containing coformulations, Idelalisib, Indinavir, Itraconazole, Ketoconazole, Levoketoconazole, Lonafarnib, Lopinavir, Mifeprostone, Mibefradil,Nefazodone, Nelfinavir, Ombitasvir-paritaprevir-ritonavir plus dasabuvir, Posaconazole, Ribociclib, Ritonavir, Saquinavir, Telithromycin, Troleandomycin, Tucatinib, Voriconazole) from randomization to 21 days post-randomization. For a full list of CYP3A4 substrates, please reference this regularly updated list: https://drug-interactions.medicine.iu.edu/MainTable.aspx.

22.8 Fostamatinib Arm Logistics

The use of fostamatinib for the proposed indication is investigational and the study will be conducted under FDA IND #154000 and will cross reference IND #152131. Fostamatinib can be stored at room temperature, 20°C to 25°C (68°F to 77°F); excursions permitted between 15°C to 30°C (59°F to 86°F). Rigel has adequate investigational product and placebo to enroll 300 active and 300 placebo patients.

For regulatory reporting purposes, including identification and potential expedited reporting of 'SUSAR' events, the following serious and/or non-serious Adverse Events/Reactions are among the events that are considered expected for this study:

- Neutropenia
- Diarrhea
- Elevated AST, ALT, or bilirubin. However, AST or ALT greater than 10 times the local laboratory ULN or the baseline value at the time of randomization, whichever is greater, is considered unexpected for fostamatinib study drug for the purposes of adverse event reporting.
- Hypertension

The following is considered an AESI for the fostamatinib arm:

- AST or ALT greater than 5 times the local laboratory ULN or the baseline value at the time of randomization, whichever is greater

23 Appendix E. Non-NAT Tests Deemed with Equivalent Specificity to NAT by the Protocol Team

Date EUA* Updated (first issued)	Manufacturer	Diagnostic (Letter of Authorization)	PPA- Sensitivity	NPA- Specificity	Antigen	Days since symptom onset
13-Jan-2021 (02-Jul-2020)	Becton, Dickinson and Company (BD)	BD Veritor System for Rapid Detection of SARS-CoV-2	84%	100%	nucleocapsid	5
(11-Jan-2021) 11-Jan-2021	Ortho Clinical Diagnostics Inc.	VITROS Immunodiagnostic Products SARS-CoV-2 Antigen Reagent Pack	90.0% (76.3–97.2%)	100% (95% CI: 99.1–100.0%)	nucleocapsid	7
01-May-2021 (01-May-2021)	Quanterix Corporation	Simoa SARS-CoV-2 N Protein Antigen Test	97.7 % (95% CI: 92.03-99.72)	100% (95% CI: 90.75-100.0)	nucleocapsid	14
23-Dec-2020 (15-Dec-2020)	Ellume Limited	Ellume COVID-19 Home Test	95% [95% CI 82% - 99%]	97% [95% CI 93% - 99%]	nucleocapsid	w/ or wo/ symptoms
22-Dec-2020 (18-Dec-2020)	Quidel Corporation	QuickVue SARS Antigen Test	96.6%	99.3%	nucleocapsid	5
16-Dec-2020 (26-Aug-2020)	Abbott Diagnostics Scarborough, Inc.	BinaxNOW COVID-19 Ag Card	84.6% (95% CI: 76.8% - 90.6%)	98.5% (95% CI: 96.6% - 99.5%)	nucleocapsid	7
16-Dec-2020 (16-Dec-2020)	Abbott Diagnostics Scarborough, Inc.	BinaxNOW COVID-19 Ag Card Home Test	91.7% (95% CI: 73.0% - 98.9%)	100.0% (95% CI: 87.7% - 100.0%)	nucleocapsid	7
07-Dec-2020 07-Dec-2020	Luminostics, Inc.	Clip COVID Rapid Antigen Test	96.9% (95% CI: 83.8% - 99.9%)	100% (95% CI: 97.3% - 100%)	nucleocapsid	7
23-Oct-2020 (23-Oct-2020)	Celltrion USA, Inc.	Sampinute COVID-19 Antigen MIA	99.4 %	100%	receptor binding domains (RBDs) spike proteins	5
13-Oct-2020 (08-Oct-2020)	Access Bio, Inc.	CareStart COVID-19 Antigen test	88.4 %	100%	nucleocapsid	5
02-Oct-2020 (02-Oct-2020)	Quidel Corporation	Sofia 2 Flu + SARS Antigen FIA	95.2 %	100%	nucleocapsid	5
18-Aug-2020 (18-Aug-2020)	LumiraDx UK Ltd.	LumiraDx SARS-CoV-2 Ag Test	97.6 % (91.6 % - 99.3 %)	96.6 % (92.7 % - 98.4 %)	nucleocapsid	12
17Jul 2020 (08-May-2020)	Quidel Corporation	Sofia SARS Antigen FIA	96.7 % (96.7% - 99.4 %)	100 % (97.9 %- 100.0 %)	nucleocapsid	-

24 References

1. Kashyap S, Gombar S, Yadlowsky S, Callahan A, Fries J, Pinsky BA, Shah NH. Measure what matters: Counts of hospitalized patients are a better metric for health system capacity planning for a reopening. Journal of the American Medical Informatics Association. 2020 Jul 1;27(7):1026–1131.
2. Price-Haywood EG, Burton J, Fort D, Seoane L. Hospitalization and Mortality among Black Patients and White Patients with Covid-19. N Engl J Med. Massachusetts Medical Society; 2020 May 27;382(26):2534–2543.
3. Xu P, Sun GD, Li ZZ. Clinical characteristics of two human-to-human transmitted coronaviruses: Corona Virus Disease 2019 vs. Middle East Respiratory Syndrome Coronavirus. Eur Rev Med Pharmacol Sci. 2020;24(10):5797–5809. PMID: 32495918
4. Nguyen NT, Chinn J, Nahmias J, Yuen S, Kirby KA, Hohmann S, Amin A. Outcomes and Mortality Among Adults Hospitalized With COVID-19 at US Medical Centers. JAMA Netw Open. 2021 Mar 5;4(3):e210417.
5. Bilaloglu S, Aphinyanaphongs Y, Jones S, Iturrate E, Hochman J, Berger JS. Thrombosis in Hospitalized Patients With COVID-19 in a New York City Health System. JAMA. 2020 Aug 25;324(8):799.
6. Lewnard JA, Liu VX, Jackson ML, Schmidt MA, Jewell BL, Flores JP, Jentz C, Northrup GR, Mahmud A, Reingold AL, Petersen M, Jewell NP, Young S, Bellows J. Incidence, clinical outcomes, and transmission dynamics of severe coronavirus disease 2019 in California and Washington: prospective cohort study. BMJ [Internet]. British Medical Journal Publishing Group; 2020 May 22 [cited 2020 Sep 17];369. Available from: https://www.bmj.com/content/369/bmj.m1923 PMID: 32444358
7. CDC. Coronavirus Disease 2019 (COVID-19) [Internet]. Centers for Disease Control and Prevention. 2020 [cited 2020 Sep 17]. Available from: https://www.cdc.gov/coronavirus/2019-ncov/hcp/clinical-guidance-management-patients.html
8. Goldman JD, Lye DCB, Hui DS, Marks KM, Bruno R, Montejano R, Spinner CD, Galli M, Ahn MY, Nahass RG, Chen YS, SenGupta D, Hyland RH, Osinusi AO, Cao H, Blair C, Wei X, Gaggar A, Brainard DM, Towner WJ, Muñoz J, Mullane KM, Marty FM, Tashima KT, Diaz G, Subramanian A. Remdesivir for 5 or 10 Days in Patients with Severe Covid-19. New England Journal of Medicine. Massachusetts Medical Society; 2020 May 27;0(0):null.
9. RECOVERY Collaborative Group, Horby P, Lim WS, Emberson JR, Mafham M, Bell JL, Linsell L, Staplin N, Brightling C, Ustianowski A, Elmahi E, Prudon B, Green C, Felton T, Chadwick D, Rege K, Fegan C, Chappell LC, Faust SN, Jaki T, Jeffery K, Montgomery A, Rowan K, Juszczak E, Baillie JK, Haynes R, Landray MJ. Dexamethasone in Hospitalized Patients with Covid-19 - Preliminary Report. N Engl J Med. 2020 Jul 17; PMCID: PMC7383595
10. Pan A, Liu L, Wang C, Guo H, Hao X, Wang Q, Huang J, He N, Yu H, Lin X, Wei S, Wu T. Association of Public Health Interventions With the Epidemiology of the COVID-19 Outbreak in Wuhan, China. JAMA. 2020 May 19;323(19):1915–1923. PMCID: PMC7149375
11. Uhal BD, Li X, Piasecki CC, Molina-Molina M. Angiotensin signalling in pulmonary fibrosis. Int J Biochem Cell Biol. 2012 Mar;44(3):465–468. PMCID: PMC3288339
12. Gu H, Xie Z, Li T, Zhang S, Lai C, Zhu P, Wang K, Han L, Duan Y, Zhao Z, Yang X, Xing L, Zhang P, Wang Z, Li R, Yu JJ, Wang X, Yang P. Angiotensin-converting enzyme 2 inhibits lung injury induced by respiratory syncytial virus. Sci Rep. 2016 Jan 27;6:19840. PMCID: PMC4728398
13. Zou Z, Yan Y, Shu Y, Gao R, Sun Y, Li X, Ju X, Liang Z, Liu Q, Zhao Y, Guo F, Bai T, Han Z, Zhu J, Zhou H, Huang F, Li C, Lu H, Li N, Li D, Jin N, Penninger JM, Jiang C. Angiotensin-converting enzyme 2 protects from lethal avian influenza A H5N1 infections. Nat Commun. 2014 May 6;5:3594. PMCID: PMC7091848

14. Liu M, Wang T, Zhou Y, Zhao Y, Zhang Y, Li J. Potential Role of ACE2 in Coronavirus Disease 2019 (COVID-19) Prevention and Management. J Transl Int Med. 2020 Mar;8(1):9–19. PMCID: PMC7227161
15. Wenz M, Hoffmann B, Bohlender J, Kaczmarczyk G. Angiotensin II formation and endothelin clearance in ARDS patients in supine and prone positions. Intensive Care Med. 2000 Mar;26(3):292–298. PMID: 10823385
16. Doerschug KC, Delsing AS, Schmidt GA, Ashare A. Renin-angiotensin system activation correlates with microvascular dysfunction in a prospective cohort study of clinical sepsis. Crit Care. 2010;14(1):R24. PMCID: PMC2875539
17. Wenz M, Steinau R, Gerlach H, Lange M, Kaczmarczyk G. Inhaled Nitric Oxide Does Not Change Transpulmonary Angiotensin II Formation in Patients With Acute Respiratory Distress Syndrome. Chest. 1997 Aug;112(2):478–483.
18. Shen L, Mo H, Cai L, Kong T, Zheng W, Ye J, Qi J, Xiao Z. Losartan prevents sepsis-induced acute lung injury and decreases activation of nuclear factor kappaB and mitogen-activated protein kinases. Shock. 2009 May;31(5):500–506. PMID: 18827741
19. Marshall RP, Webb S, Bellingan GJ, Montgomery HE, Chaudhari B, McAnulty RJ, Humphries SE, Hill MR, Laurent GJ. Angiotensin converting enzyme insertion/deletion polymorphism is associated with susceptibility and outcome in acute respiratory distress syndrome. Am J Respir Crit Care Med. 2002 Sep 1;166(5):646–650. PMID: 12204859
20. Adamzik M, Frey U, Sixt S, Knemeyer L, Beiderlinden M, Peters J, Siffert W. ACE I/D but not AGT (-6)A/G polymorphism is a risk factor for mortality in ARDS. European Respiratory Journal. 2007 Mar 1;29(3):482–488.
21. Jerng JS, Yu CJ, Wang HC, Chen KY, Cheng SL, Yang PC. Polymorphism of the angiotensin-converting enzyme gene affects the outcome of acute respiratory distress syndrome. Crit Care Med. 2006 Apr;34(4):1001–1006. PMID: 16484896
22. Khan A, Benthin C, Zeno B, Albertson TE, Boyd J, Christie JD, Hall R, Poirier G, Ronco JJ, Tidswell M, Hardes K, Powley WM, Wright TJ, Siederer SK, Fairman DA, Lipson DA, Bayliffe AI, Lazaar AL. A pilot clinical trial of recombinant human angiotensin-converting enzyme 2 in acute respiratory distress syndrome. Crit Care. 2017 07;21(1):234. PMCID: PMC5588692
23. Shi S, Qin M, Shen B, Cai Y, Liu T, Yang F, Gong W, Liu X, Liang J, Zhao Q, Huang H, Yang B, Huang C. Association of Cardiac Injury With Mortality in Hospitalized Patients With COVID-19 in Wuhan, China. JAMA Cardiology. 2020 Jul 1;5(7):802.
24. Guo T, Fan Y, Chen M, Wu X, Zhang L, He T, Wang H, Wan J, Wang X, Lu Z. Cardiovascular Implications of Fatal Outcomes of Patients With Coronavirus Disease 2019 (COVID-19). JAMA Cardiology. 2020 Jul 1;5(7):811.
25. Kochi AN, Tagliari AP, Forleo GB, Fassini GM, Tondo C. Cardiac and arrhythmic complications in patients with COVID-19. J Cardiovasc Electrophysiol. 2020;31(5):1003–1008. PMCID: PMC7262150
26. Verdecchia P, Cavallini C, Spanevello A, Angeli F. The pivotal link between ACE2 deficiency and SARS-CoV-2 infection. European Journal of Internal Medicine. 2020 Jun;76:14–20.
27. Mancia G, Rea F, Ludergnani M, Apolone G, Corrao G. Renin–Angiotensin–Aldosterone System Blockers and the Risk of Covid-19. N Engl J Med. 2020 Jun 18;382(25):2431–2440.
28. Reynolds HR, Adhikari S, Pulgarin C, Troxel AB, Iturrate E, Johnson SB, Hausvater A, Newman JD, Berger JS, Bangalore S, Katz SD, Fishman GI, Kunichoff D, Chen Y, Ogedegbe G, Hochman JS. Renin–Angiotensin–Aldosterone System Inhibitors and Risk of Covid-19. N Engl J Med. 2020 Jun 18;382(25):2441–2448.
29. Lopes RD, Macedo AVS, de Barros E Silva PGM, Moll-Bernardes RJ, Feldman A, D'Andréa Saba Arruda G, de Souza AS, de Albuquerque DC, Mazza L, Santos MF, Salvador NZ, Gibson CM, Granger CB, Alexander JH, de Souza OF, BRACE CORONA investigators.

Continuing versus suspending angiotensin-converting enzyme inhibitors and angiotensin receptor blockers: Impact on adverse outcomes in hospitalized patients with severe acute respiratory syndrome coronavirus 2 (SARS-CoV-2)--The BRACE CORONA Trial. Am Heart J. 2020;226:49–59. PMCID: PMC7219415

30. Lopes RD, Macedo AVS, de Barros E Silva PGM, Moll-Bernardes RJ, Dos Santos TM, Mazza L, Feldman A, D'Andréa Saba Arruda G, de Albuquerque DC, Camiletti AS, de Sousa AS, de Paula TC, Giusti KGD, Domiciano RAM, Noya-Rabelo MM, Hamilton AM, Loures VA, Dionísio RM, Furquim TAB, De Luca FA, Dos Santos Sousa ÍB, Bandeira BS, Zukowski CN, de Oliveira RGG, Ribeiro NB, de Moraes JL, Petriz JLF, Pimentel AM, Miranda JS, de Jesus Abufaiad BE, Gibson CM, Granger CB, Alexander JH, de Souza OF, BRACE CORONA Investigators. Effect of Discontinuing vs Continuing Angiotensin-Converting Enzyme Inhibitors and Angiotensin II Receptor Blockers on Days Alive and Out of the Hospital in Patients Admitted With COVID-19: A Randomized Clinical Trial. JAMA. 2021 Jan 19;325(3):254–264. PMCID: PMC7816106
31. Continuation versus discontinuation of renin–angiotensin system inhibitors in patients admitted to hospital with COVID-19: a prospective, randomised, open-label trial - The Lancet Respiratory Medicine [Internet]. [cited 2021 Jul 12]. Available from: https://www.thelancet.com/journals/lanres/article/PIIS2213-2600(20)30558-0/fulltext
32. Schoenfeld DA, Bernard GR, ARDS Network. Statistical evaluation of ventilator-free days as an efficacy measure in clinical trials of treatments for acute respiratory distress syndrome. Crit Care Med. 2002 Aug;30(8):1772–1777. PMID: 12163791
33. National Heart, Lung, and Blood Institute Acute Respiratory Distress Syndrome (ARDS) Clinical Trials Network, Wiedemann HP, Wheeler AP, Bernard GR, Thompson BT, Hayden D, deBoisblanc B, Connors AF, Hite RD, Harabin AL. Comparison of two fluid-management strategies in acute lung injury. N Engl J Med. 2006 Jun 15;354(24):2564–2575. PMID: 16714767
34. Laterre PF, Berry SM, Blemings A, Carlsen JE, François B, Graves T, Jacobsen K, Lewis RJ, Opal SM, Perner A, Pickkers P, Russell JA, Windeløv NA, Yealy DM, Asfar P, Bestle MH, Muller G, Bruel C, Brulé N, Decruyenaere J, Dive AM, Dugernier T, Krell K, Lefrant JY, Megarbane B, Mercier E, Mira JP, Quenot JP, Rasmussen BS, Thorsen-Meyer HC, Vander Laenen M, Vang ML, Vignon P, Vinatier I, Wichmann S, Wittebole X, Kjølbye AL, Angus DC, SEPSIS-ACT Investigators. Effect of Selepressin vs Placebo on Ventilator- and Vasopressor-Free Days in Patients With Septic Shock: The SEPSIS-ACT Randomized Clinical Trial. JAMA. 2019 Oct 2; PMCID: PMC6802260
35. Self WH, Semler MW, Wanderer JP, Wang L, Byrne DW, Collins SP, Slovis CM, Lindsell CJ, Ehrenfeld JM, Siew ED, Shaw AD, Bernard GR, Rice TW, SALT-ED Investigators. Balanced Crystalloids versus Saline in Noncritically Ill Adults. N Engl J Med. 2018 01;378(9):819–828. PMCID: PMC5846618
36. Self WH, Semler MW, Leither LM, Casey JD, Angus DC, Brower RG, Chang SY, Collins SP, Eppensteiner JC, Filbin MR, Files DC, Gibbs KW, Ginde AA, Gong MN, Harrell FE, Hayden DL, Hough CL, Johnson NJ, Khan A, Lindsell CJ, Matthay MA, Moss M, Park PK, Rice TW, Robinson BRH, Schoenfeld DA, Shapiro NI, Steingrub JS, Ulysse CA, Weissman A, Yealy DM, Thompson BT, Brown SM, National Heart, Lung, and Blood Institute PETAL Clinical Trials Network, Steingrub J, Smithline H, Tiru B, Tidswell M, Kozikowski L, Thornton-Thompson S, De Souza L, Hou P, Baron R, Massaro A, Aisiku I, Fredenburgh L, Seethala R, Johnsky L, Riker R, Seder D, May T, Baumann M, Eldridge A, Lord C, Shapiro N, Talmor D, O'Mara T, Kirk C, Harrison K, Kurt L, Schermerhorn M, Banner-Goodspeed V, Boyle K, Dubosh N, Filbin M, Hibbert K, Parry B, Lavin-Parsons K, Pulido N, Lilley B, Lodenstein C, Margolin J, Brait K, Jones A, Galbraith J, Peacock R, Nandi U, Wachs T, Matthay M, Liu K, Kangelaris K, Wang R, Calfee C, Yee K, Hendey G, Chang S, Lim G, Qadir N, Tam A, Beutler R, Levitt J, Wilson J,

Rogers A, Vojnik R, Roque J, Albertson T, Chenoweth J, Adams J, Pearson S, Juarez M, Almasri E, Fayed M, Hughes A, Hillard S, Huebinger R, Wang H, Vidales E, Patel B, Ginde A, Moss M, Baduashvili A, McKeehan J, Finck L, Higgins C, Howell M, Douglas I, Haukoos J, Hiller T, Lyle C, Cupelo A, Caruso E, Camacho C, Gravitz S, Finigan J, Griesmer C, Park P, Hyzy R, Nelson K, McDonough K, Olbrich N, Williams M, Kapoor R, Nash J, Willig M, Ford H, Gardner-Gray J, Ramesh M, Moses M, Ng Gong M, Aboodi M, Asghar A, Amosu O, Torres M, Kaur S, Chen JT, Hope A, Lopez B, Rosales K, Young You J, Mosier J, Hypes C, Natt B, Borg B, Salvagio Campbell E, Hite RD, Hudock K, Cresie A, Alhasan F, Gomez-Arroyo J, Duggal A, Mehkri O, Hastings A, Sahoo D, Abi Fadel F, Gole S, Shaner V, Wimer A, Meli Y, King A, Terndrup T, Exline M, Pannu S, Robart E, Karow S, Hough C, Robinson B, Johnson N, Henning D, Campo M, Gundel S, Seghal S, Katsandres S, Dean S, Khan A, Krol O, Jouzestani M, Huynh P, Weissman A, Yealy D, Scholl D, Adams P, McVerry B, Huang D, Angus D, Schooler J, Moore S, Files C, Miller C, Gibbs K, LaRose M, Flores L, Koehler L, Morse C, Sanders J, Langford C, Nanney K, MdalaGausi M, Yeboah P, Morris P, Sturgill J, Seif S, Cassity E, Dhar S, de Wit M, Mason J, Goodwin A, Hall G, Grady A, Chamberlain A, Brown S, Bledsoe J, Leither L, Peltan I, Starr N, Fergus M, Aston V, Montgomery Q, Smith R, Merrill M, Brown K, Armbruster B, Harris E, Middleton E, Paine R, Johnson S, Barrios M, Eppensteiner J, Limkakeng A, McGowan L, Porter T, Bouffler A, Leahy JC, deBoisblanc B, Lammi M, Happel K, Lauto P, Self W, Casey J, Semler M, Collins S, Harrell F, Lindsell C, Rice T, Stubblefield W, Gray C, Johnson J, Roth M, Hays M, Torr D, Zakaria A, Schoenfeld D, Thompson T, Hayden D, Ringwood N, Oldmixon C, Ulysse C, Morse R, Muzikansky A, Fitzgerald L, Whitaker S, Lagakos A, Brower R, Reineck L, Aggarwal N, Bienstock K, Freemer M, Maclawiw M, Weinmann G, Morrison L, Gillespie M, Kryscio R, Brodie D, Zareba W, Rompalo A, Boeckh M, Parsons P, Christie J, Hall J, Horton N, Zoloth L, Dickert N, Diercks D. Effect of Hydroxychloroquine on Clinical Status at 14 Days in Hospitalized Patients With COVID-19: A Randomized Clinical Trial. JAMA. 2020 Dec 1;324(21):2165–2176. PMCID: PMC7653542

37. Self WH, Stewart TG, Wheeler AP, Atrouni WE, Bistran-Hall AJ, Casey JD, Cataldo VD, Chappell JD, Cohn CS, Collins JB, Denison MR, de Wit M, Dixon SL, Duggal A, Edwards TL, Fontaine MJ, Ginde AA, Harkins MS, Harrington T, Harris ES, Hoda D, Ipe TS, Jaiswal SJ, Johnson NJ, Jones AE, Laguio-Vila M, Lindsell CJ, Mallada J, Mammen MJ, Metcalf RA, Middleton EA, Mucha S, O'Neal HR, Pannu SR, Pulley JM, Qiao X, Raval JS, Rhoads JP, Schrager H, Shanholtz C, Shapiro NI, Schrantz SJ, Thomsen I, Vermillion KK, Bernard GR, Rice TW. Passive Immunity Trial for Our Nation (PassITON): study protocol for a randomized placebo-control clinical trial evaluating COVID-19 convalescent plasma in hospitalized adults. Res Sq. 2021 Mar 2; PMCID: PMC7941637
38. Beigel JH, Tomashek KM, Dodd LE, Mehta AK, Zingman BS, Kalil AC, Hohmann E, Chu HY, Luetkemeyer A, Kline S, Lopez de Castilla D, Finberg RW, Dierberg K, Tapson V, Hsieh L, Patterson TF, Paredes R, Sweeney DA, Short WR, Touloumi G, Lye DC, Ohmagari N, Oh MD, Ruiz-Palacios GM, Benfield T, Fätkenheuer G, Kortepeter MG, Atmar RL, Creech CB, Lundgren J, Babiker AG, Pett S, Neaton JD, Burgess TH, Bonnett T, Green M, Makowski M, Osinusi A, Nayak S, Lane HC, ACTT-1 Study Group Members. Remdesivir for the Treatment of Covid-19 - Final Report. N Engl J Med. 2020 Nov 5;383(19):1813–1826. PMCID: PMC7262788
39. Moskowitz A, Shotwell MS, Gibbs KW, Harkins M, Rosenberg Y, Troendle J, Merck LH, Files DC, Wit M de, Hudock K, Thompson BT, Gong MN, Ginde AA, Douin DJ, Brown SM, Rubin E, Joly MM, Wang L, Lindsell CJ, Bernard GR, Semler MW, Collins SP, Self WH. Oxygen-Free Days as an Outcome Measure in Clinical Trials of Therapies for COVID-19 and Other Causes

of New Onset Hypoxemia. CHEST [Internet]. Elsevier; 2022 Apr 30 [cited 2022 Jun 7];0(0). Available from: https://journal.chestnet.org/article/S0012-3692(22)00889-3/abstract

40. Jr FEH. rmsb Package Examples Using rstan [Internet]. 2024 [cited 2024 Sep 25]. Available from: https://hbiostat.org/r/examples/blrm/blrm
41. Harrell, Frank. General COVID-19 Therapeutics Trial Design [Internet]. [cited 2020 Nov 6]. Available from: https://hbiostat.org/proj/covid19/statdesign.html
42. Simpson DG, Carroll RJ, Zhou H, Guth DJ. Interval Censoring and Marginal Analysis in Ordinal Regression. Journal of Agricultural, Biological, and Environmental Statistics. 1996 Sep;1(3):354.
43. Food and Drug Administration. E9(R1) Statistical Principles for Clinical Trials: Addendum: Estimands and Sensitivity Analysis in Clinical Trials [Internet]. 2017. Available from: https://www.fda.gov/media/108698/download
44. Zhenming S, McCullagh P. Laplace approximation of high dimensional integrals. Journal of the Royal Statistical Society. 1995;57(4):749–760.
45. Doob J. The limiting distributions of certain statistics. The Annals of Mathematical Statistics. 1935 Sep 1;6(3):160–169.
46. Liu Q, Shepherd BE, Li C, Harrell FE. Modeling continuous response variables using ordinal regression. Stat Med. 2017 Nov 30;36(27):4316–4335. PMCID: PMC5675816
47. Harrell, Frank. Violation of Proportional Odds is Not Fatal | Statistical Thinking [Internet]. [cited 2020 Nov 6]. Available from: https://www.fharrell.com/post/po/
48. World Health Organization. WHO R&D Blueprint - Novel Coronavirus, COVID-19 Therapeutic Trial Synopsis [Internet]. 2020. Available from: https://www.who.int/blueprint/priority-diseases/key-action/COVID-19_Treatment_Trial_Design_Master_Protocol_synopsis_Final_18022020.pdf

NECTAR

Novel Experimental COVID Therapies Affecting Host Response

Statistical Analysis Plan

CONNECTS Master Protocol for Clinical Trials targeting macro-, micro-immuno-thrombosis, vascular hyperinflammation, and hypercoagulability and renin-angiotensin-aldosterone system (RAAS) in hospitalized patients with COVID-19 (ACTIV 4 Host Tissue)

Version 1.4
Nov 14, 2023

Study Chair:

Sean P. Collins, MD, MSc.
Vanderbilt University Medical Center
1313 21st Ave S #801
Nashville, TN 37232
Phone: 615-936-0087
Email: sean.collins@vumc.org

Principal Investigator, Clinical Coordinating Center (CCC):

Wesley H. Self, MD, MPH
Vanderbilt University Medical Center
1313 21st Ave S
Nashville, TN 37232
Phone: 615-936-0253
Email: wesley.self@vumc.org

Principal Investigator, Data Coordinating Center (DCC)

Matthew S. Shotwell, PhD
Vanderbilt University School of Medicine
2525 West End Avenue
Suite 11000
Nashville, TN 37203
Phone: 615-875-3397
Email: matt.shotwell@vumc.org

Revision History:

Ver.	Date	Authors	Summary of Revisions:
1.0	2/14/22	M. Shotwell	Initial version
1.1	3/8/22	M. Shotwell	• Simplified interim stopping rule<br>• Updated simulation results and decision thresholds at interim and final analyses<br>• Clarified purpose of AngioNECTAR<br>• Additional details about sensitivity analyses<br>• Summarized sample size reassessment process<br>• Additional details of final analysis procedure
1.2	7/28/22	M. Shotwell	• Clarifications to address FDA comments<br>• Additional tipping point analysis to evaluate effect of partially observed outcomes on efficacy conclusion<br>• Additional supplemental appendices
1.3	3/2/22	M. Shotwell	• Clarifications to address FDA comments
1.4	11/14/23	M. Shotwell	• Clarifications to address FDA comments<br>• Additional sensitivity analysis to evaluate treatment efficacy if the proportional odds assumption is violated.

Signatures:

Name **Date**

Name **Date**

Name **Date**

Name **Date**

Name **Date**

ABBREVIATIONS AND ACRONYMS

ACE	Angiotensin-Converting Enzyme
AE	Adverse Event
ANG	Angiotensin
API	Application Programming Interface
ARDS	Acute Respiratory Distress Syndrome
BD	Becton Dickinson
BID	Biospecimen Identity
BCL	Biorepository and Central Laboratory
BMP	Basic Metabolic Panel
CBC	Complete Blood Count
CCC	Clinical Coordinating Center
CDE	Common Data Element
CDISC	Clinical Data Interchange Standards Consortium
CDASH	Clinical Data Acquisition Standards Harmonization
CFR	Code of Federal Regulations
COVID-19	Coronavirus Disease 2019
CRF	Case Report Form
CRP	C-Reactive Protein
CTOM	Clinical Trial Operation Manager
CTCAE	Common Terminology Criteria for Adverse Events
DAG	Data Access Group
DAIDS	Division of Acquired Immunodeficiency Syndrome
DCC	Data Coordinating Center
DOB	Date of Birth
DOR	Delegation of Responsibilities
DR	Disaster Recovery
DSMB	Data Safety Monitoring Board
DSMP	Data Safety Monitoring Plan
eConsent	Electronic Consent
eCRF	Electronic Case Report Forms
EDC	Electronic Data Capture
EMR	Electronic Medical Record
FAQ	Frequently Asked Question
FDA	Food and Drug Administration
GDPR	General Data Protection Regulation
GUID	Globally Unique Identifier
HT	Host Tissue
hsTn	High-sensitivity Troponins
ICF	Informed Consent Form
ICH GCP	International Conference of Harmonization Good Clinical Practice
ID	Identity
IMV	Invasive Mechanical Ventilation
INR	International Normalized Ratio
IP	Investigational Product
IRB	Institutional Review Board

IT	Information Technology
ITT	Intent-To-Treat
KSP	Key Study Personnel
LAR	Legally Authorized Representative
LFT	Liver Function Test
MOP	Manual of Procedures
NAT	Nucleic Acid Test
NHLBI	National Heart, Lung, and Blood Institute
NINDS	National Institute of Neurological Disorders and Stroke
NTproBNP	N-terminal prohormone B-type natriuretic peptide
N3C	National COVID Cohort Collaborative
PDF	Portable Data Format
PI	Principal Investigator
PSESE	Protocol-Specified Exempt Serious Events
PT	Prothrombin Time
PTT	Partial Thromboplastin Time
REDCap	Research Electronic Data Capture
RTI	Research Triangle Institute
SAP	Statistical Analysis Plan
SAE	Serious Adverse Event
sIRB	Single Institutional Review Board
SOC	Standard of Care
SOFA	Sequential Organ Failure Assessment
UAT	User Acceptance Testing
VCC	Vanderbilt Coordinating Center
VMP	Validation Master Plan
VUMC	Vanderbilt University Medical Center
VUMC-IT	Vanderbilt University Medical Center – Information Technology
VICTR-ORI	Vanderbilt Institute for Clinical and Translational Research – Office of Research Informatics
WHO	World Health Organization

1 INTRODUCTION

The Statistical Analysis Plan (SAP) was developed by DCC statisticians in collaboration with study team leadership and NHLBI representatives. The SAP describes treatment arms, analysis datasets, all outcomes and planned analyses, randomization procedure and algorithm, decision thresholds and interim stopping rules, design and results of simulations to determine power and sample size and demonstrate study operating characteristics, procedures for handling missing data, and any other information that is essential to carry out all statistical analyses.

1.1 AngioNECTAR SAP

AngioNECTAR is a mechanistic sub-study that will utilize biospecimens collected as part of ACTIV 4 Host Tissue and complement the clinical information obtained in our primary analysis. This sub-study will examine the effects of study therapies on biomarkers of the Renin-Angiotensin-Aldosterone-System. Statistical analyses associated with the AngioNECTAR sub-study will be designed and implemented by AngioNECTAR PI D. Clark Files, MD, and Co-Investigators Mark Chappell, PhD and Chris Schaich, PhD. A separate SAP for the AngioNECTAR sub-study will be finalized by the AngioNECTAR investigators prior to unblinding of the active/placebo status for sub-study participants.

1.2 SAP Approval and Revision

The SAP will be reviewed and approved by the ACTIV 4 Host Tissue stakeholders listed below prior to the first interim analysis for any arm:

- ACTIV 4 Host Tissue Study Chair: Sean Collins, MD
- ACTIV 4 Host Tissue DCC PI: Matthew S. Shotwell, PhD
- NHLBI Statistician: James Troendle, PhD

Amendments to the SAP must also be approved by the stakeholders listed above. Amendments must be version controlled and numbered. All revisions will be summarized briefly, including the changes made, new version number, and the author of the changes.

2 STUDY DESIGN

2.1 Summary

The ACTIV 4 Host Tissue master protocol describes a common approach to studies of blinded, placebo-controlled therapeutic approaches of host-tissue targeted therapies in hospitalized COVID-19 patients. The Master Protocol is designed to be flexible in the number of study arms, to have a common placebo group, and to allow for stopping and adding of new therapies, while using a common approach to design, analysis, and implementation.

2.2 Study Arms and Pooled Placebo

The ACTIV 4 Host Tissue platform consists of multiple study arms that represent distinct drug therapies. During the randomization process, each participant is assigned a study arm and either the active drug or a matching placebo. The statistical analyses described herein will be implemented separately for each study arm. However, placebo participants will be pooled across arms. For each study arm, the placebo comparator group will consist of all placebo participants that were *eligible* for that study arm at the time of randomization. A participant is considered eligible for a study arm if assignment to that arm was a possible outcome of randomization. Participants that decline to participate in any one or more study arms prior to

randomization will be treated as ineligible for those arms. The randomization process is designed to ensure balance in each active drug group versus the corresponding placebo comparator group.

2.3 Randomization

Participants are randomized individually at enrollment using a central electronic system. The permuted block method, with stratification by study site and study arm eligibility is used to generate treatment assignments. An eligibility stratum is the collection of study arms for which a participant is eligible. Stratification by site ensures balance across the active and pooled placebo comparator groups at regular enrollment intervals at each site, thus mitigating the impact of site heterogeneity on assessments of treatment effect. Each block contains a multiple of $m(m+1)$ assignments, where m is the number of study arms in the corresponding eligibility stratum. Within each block there are an equal number of allocations across study arms and, for each study arm, there are m active and 1 placebo assignments. For example, in the TXA127 and TRV027 eligibility stratum, each block consists of the following allocations, or multiples thereof:

Study Arm	Placebo/Active
TXA127	Active
TXA127	Active
TXA127	Placebo
TRV027	Active
TRV027	Active
TRV027	Placebo

Thus, within each block, assignments are balanced across study arms, and the active assignments are balanced with the pooled placebo assignments. The block size multiple is either 1 or 2, selected uniformly at random for each block.

2.4 Blinding

For organizational purposes, the randomized assignment comprises two distinct pieces of information: 1) study arm, and 2) active vs. placebo assignment. The study arm is not blinded, whereas the active/placebo assignment is blinded from participants and investigators (other than unblinded personnel as required for study operations, data quality/analysis, and safety). Blinding will remain in place until all participants have completed the study, all data quality monitoring is complete, and the database is locked.

3 OUTCOMES

3.1 Primary Outcome

The primary outcome for the ACTIV 4 Host Tissue platform is oxygen free days (OFD) at day 28. OFD will be calculated as the number of calendar days during the first 28 days after randomization during which the patient was alive and not receiving supplemental oxygen therapy. Participants who chronically used supplemental oxygen prior to their COVID-19 illness will be considered oxygen free when their use of supplemental oxygen does not exceed the level of oxygen support (measured in daily L/min·h by nasal canula) used prior to COVID-19 illness. Supplemental oxygen therapy includes the following: supplemental oxygen by nasal cannula, supplemental oxygen by face mask, high flow nasal cannula (HFNC), non-invasive ventilation (NIV), invasive mechanical ventilation (IMV), or extracorporeal membrane

oxygenation (ECMO). The day of randomization is defined as day 0. Starting with study day 1 (the day after randomization) and continuing for 28 days, study personnel will document whether the participant received supplemental oxygen therapy on each day for any duration of time. Use of supplemental oxygen at home after discharge will be assessed via telephone follow-up calls to the participant or surrogates. OFD will be calculated as 28 minus the number of days between and including the first and last days of supplemental oxygen use during the first 28 days after randomization. OFD will be coded as -1 for patients who died on or before study day 28. Hence, OFD may take any integer value between -1 and 28. OFD is an ordered categorical (i.e., ordinal) outcome that may be interpreted as a count of days. Additional details about calculating OFDs may be found in the SAP appendix (see Appendix: Algorithm to Compute Primary Outcome).

3.2 Secondary Outcomes

Listed below are the ACTIV 4 Host Tissue platform secondary outcomes. The “Test Order” field indicates the order in which key secondary outcomes will be tested, using the fixed-sequence method, to control the familywise type-I error probability across the primary and key secondary outcomes.

Description	Type	Test Order	Analysis Method
Alive and oxygen free at day 14	Binary		LogR
Alive and oxygen free at day 28	Binary		LogR
Alive and respiratory failure-free at day 14	Binary		LogR
Alive and respiratory failure-free at day 28	Binary	1	LogR
Alive and free of new IMV at day 14	Binary		LogR
Alive and free of new IMV at day 28	Binary		LogR
Mortality in-hospital	Binary		LogR
Mortality at day 28	Binary	3	LogR
Mortality at day 60	Binary		LogR
Mortality at day 90	Binary		LogR
WHO 8-point ordinal scale at day 14	Ordinal		POLR
WHO 8-point ordinal scale at day 28	Ordinal	2	POLR
WHO 8-point ordinal scale at day 60	Ordinal		POLR
Hospital-free days at day 28	Ordinal		POLR
Respiratory failure-free days at day 28	Ordinal		POLR
Ventilator-free days at day 28	Ordinal		POLR

LogR – Logistic Regression; POLR – Proportional Odds Logistic Regression

The WHO 8-point ordinal scale is defined as most severe clinical status among the following on the day of assessment:

1. Ambulatory – Not hospitalized, no limitation of activities
2. Ambulatory – Not hospitalized with limitation of activities or home oxygen therapy
3. Hospitalized Mild Disease – Hospitalized, no oxygen therapy
4. Hospitalized Mild Disease – Oxygen by mask or nasal prongs
5. Hospitalized Severe Disease – Non-invasive ventilation of high-flow oxygen
6. Hospitalized Severe Disease – IMV
7. Hospitalized Severe Disease – IMV + organ support with-vasopressors, RRT, or ECMO
8. Dead

Alive and respiratory failure-free at day 28, the WHO 8-point ordinal scale at day 28, and Mortality at day 28 are key secondary outcomes that will be treated as a family for testing

purposes, even though the studies will not be adequately powered to detect anything but a very strong treatment effect on these outcomes. A supplementary analysis to assess the evidence that treatment lowers the risk of death in a way that is consistent with its effect on nonfatal outcomes will be performed. A respiratory failure-free day is defined as a day alive without the use of HFNC, NIV, IMV, or ECMO.

3.3 Safety Outcomes

Safety outcomes include the following events, assessed daily during hospitalization or intermittently following hospital discharge. For each event, we will analyze two composite binary outcomes: 1) the occurrence of one or more such events by the end of study day 7 and 2) the occurrence of one or more such events by the end of study day 28.

Description	Type	Analysis Method
Hypotension	Binary	LogR
Allergic reaction, rash, or angioedema	Binary	LogR
Incident renal replacement therapy	Binary	LogR
Other PSESE	Binary	LogR

LogR – Logistic Regression

Hypotension is defined by low arterial blood pressure leading to either [1] initiation or increase in vasopressor therapy, [2] administration of a fluid bolus of 500 ml or more, or [3] modification of the dose or discontinuation of the study drug.

3.4 Exploratory Outcomes

Exploratory outcomes will include (at least) the following:

Description	Type	Analysis Method
Change in troponin during hospitalization	Quantitative	LinR
Change in NT-proBNP	Quantitative	LinR
Change in RAAS mechanistic biomarkers:<br>1. AngII<br>2. Ang(1-7)<br>3. Plasma renin activity<br>4. Aldosterone<br>5. ACE<br>6. ACE2	Quantitative	LinR
Change in serum creatinine	Quantitative	LinR
Change in eGFR	Quantitative	LinR
Acute kidney injury (KDIGO criteria)	Ordinal	POLR

LinR – Linear Regression; POLR – Proportional Odds Logistic Regression

Exploratory outcomes may be collected at just a subset of sites.

4 ANALYSIS DATASETS

For each study arm, the following analysis datasets will be produced using records for participants that were assigned to the active drug group and placebo participants that were *eligible* for the active drug group at the time of randomization:

Modified intention-to-treat dataset: The mITT analysis dataset will include all randomized participants grouped by study arm and active/placebo assignment at randomization, regardless of subsequent compliance or protocol violations, with the following exceptions: 1. Participants who have not received the study drug assigned at randomization will be excluded. 2. Participants who were randomized and later found to be ineligible based on assessments initiated prior to randomization will be excluded. All statistical analyses will be implemented using mITT dataset unless otherwise explicitly specified in this statistical analysis plan.

Intention-to-treat dataset: The intention-to-treat (ITT) analysis dataset will consist of all randomized participants grouped by study arm and active/placebo assignment at randomization regardless of subsequent compliance or protocol violations.

Safety dataset: The safety analysis dataset will consist of all participants grouped by the drug(s) received.

5 EFFICACY TESTING & FAMILYWISE TYPE-I ERROR CONTROL

Efficacy regarding the primary outcome and each key secondary outcome will be tested using a one-sided method that ensures no more than a 2.5% chance of a type-I error. The fixed-sequence method will be used to control the familywise type-I error probability at 2.5% for the family of primary and key secondary outcomes.[1] Specifically, a conclusion of efficacy regarding the primary outcome will be required prior to testing the first designated key secondary outcome. Each subsequent key secondary outcome, in the designated order, will take place only if the preceding key secondary outcome demonstrates efficacy. This approach provides strong control of the familywise type-I error probability at 2.5% for the family of primary and key secondary outcomes. No other statistical hypothesis tests will be made regarding other secondary, safety, or exploratory outcomes. P-values associated with certain null hypothesis tests may be provided for descriptive purposes, or to fulfill special requests, e.g., for DSMB safety assessments.

6 ANALYSIS OF THE PRIMARY OUTCOME

The effect of the active drug versus placebo will be quantified using an odds ratio – the primary estimand – which quantifies the treatment effect on the odds of greater oxygen-free days at day 28. Based on the behavior of similar outcomes in prior trials,[2-6] we anticipate the distribution of the primary outcome to be irregular, with peaks around -1 to 0 and between 22 and 28 days. Thus, we will use a flexible semi-parametric approach for the primary outcome analysis. Estimation and inferences about the odds ratio will be made using Bayesian proportional odds (PO) logistic regression methods, adjusting for the active drug vs placebo indicator variable, age group (18-30, 31-65, >65 years), sex at birth, and WHO COVID ordinal outcome score at baseline (4, 5, and 6-7).[7] Evidence for efficacy will be quantified using the posterior probability that the active drug versus placebo odds ratio is greater than one (i.e., treatment is associated with greater oxygen free days at day 28). This is denoted the "efficacy probability" or $P(\mathrm{OR} > 1|\mathrm{Data})$, where OR represents the odds ratio, and Data represents the mITT analysis dataset. The "inferiority/harm probability" is defined as $P(\mathrm{OR} \leq 1|\mathrm{Data})$. The primary analysis will be implemented separately for each study arm, where the placebo comparator group will consist of placebo participants that were eligible for the corresponding study arm at randomization, regardless of the study arm assigned. The primary and supplementary estimates will be presented with 95% credible intervals.

6.1 Statistical Model

The PO model can be written in terms of the covariates X and an outcome variable Y, where probabilities of outcome value y or greater $\Pr(Y \geq y|X) = \mathrm{expit}(\alpha_y + X\beta)$ where α_y is the intercept for outcome value y and expit is the logistic (inverse logit) transformation and the columns of matrix X contain coded baseline covariates and the active/placebo treatment indicator. β represents the log odds ratio (OR) associated with the effects of covariates and group assignment. Specifically, the group assignment odds ratio represents the relative effect of treatment versus placebo on the odds $\Pr(Y \geq y|X)/(1 - \Pr(Y \geq y|X))$, for any value y.

A flat prior distribution will be used for all PO model parameters. This ensures that the estimate of the primary estimand will be free of influence from an informative prior, and the Bayesian maximum *a posteriori* estimate will be identical to the maximum likelihood estimate (see Appendix: Cumulative Logit Model). The posterior distribution for the log odds ratio will be approximated using the Laplace method.[8] Use of a flat prior ensures the Laplace-approximated posterior distribution is identical to the asymptotic sampling distribution of the maximum likelihood estimate; in both cases a normal distribution centered at the estimate with variance-covariance equal to the negative inverse Hessian of the log likelihood function (inverse observed Fisher information; see Appendix: Laplace Approximation). All statistical inferences about the odds ratio will be made using this method. Statistical uncertainty about supplementary estimands (e.g., treatment difference in the median of the primary outcome) will be quantified using the delta method.[9] Given the investigational nature of the agents tested by this platform, there is insufficient information upon which to justify a more informative prior. The flat prior approach ensures that Bayesian inferences regarding the efficacy of study agents are based exclusively on the data collected in the ACTIV 4 Host Tissue platform.

6.2 Loss to Follow-up, Censoring, and Intercurrent Events

Participants who withdraw consent prior to data collection, or for whom there is no partial information about the primary outcome, will not be excluded from analysis. We will strive to avoid loss to follow-up by making repeated attempts to contact participants or otherwise retrieve participant records. If loss-to-follow-up cannot be avoided, and the information needed to compute the primary endpoint is partially known (i.e., censored), we will use a likelihood-based method to account for this censoring. For example, if a study participant received supplemental oxygen every day during the 10-day period after randomization, but is then lost to follow-up, the primary outcome is only partially known (i.e., OFDs $\leq$ 18 in this example). The PO model provides a convenient mechanism to account for this and other types of censoring using a likelihood-based approach.[10] For observations that are fully observed, the log likelihood contribution is $l(\alpha, \beta;\ y, x) = \log \Pr(Y = y|X = x)$. For observations that are left censored at y (e.g., $\leq$ 18 OFDs), the log likelihood contribution is $l(\alpha, \beta;\ y, x) = \log \Pr(Y \leq y|X = x)$. The latter is conveniently computed by substituting $1 - \mathrm{expit}(\alpha_y + x\beta)$. More complex partially observed outcomes (e.g., right or interval censored) are modeled in a similar manner.

All primary analyses will be implemented using the mITT analysis dataset. The intercurrent event of death will be coded as a special value in the primary outcome (i.e., composite strategy). No other intercurrent events will affect the primary outcome assessment (i.e., treatment policy strategy).[11]

Participant age, sex, and WHO COVID scale at baseline are subject to source verification monitoring. Thus, we do not anticipate missing covariate data.

6.3 Planned Interim and Final Analyses, Early Stopping, and Type-I Error Control

Two planned interim analyses will occur separately for each study arm when the number of participants with complete 28-day follow-up (or were deceased, withdrawn, or lost-to-follow-up by day 28) reaches 33% and 67% of maximum enrollment for that arm. Interim analyses will be executed by unblinded personnel only. Participant records that inform the primary outcome must undergo monitoring prior to interim (and final) analysis. At each interim analysis, a study arm may be stopped early if there is evidence for inferiority/harm. Enrollment in the trial will be stopped early if the posterior probability for inferiority/harm exceeds 0.95.

Final analysis will occur once enrollment, follow-up, and the required monitoring are completed. Should additional data be collected after enrollment is halted at an interim analysis, the final analysis will incorporate this additional data. If the trial was stopped early at an interim analysis due to evidence of inferiority/harm, a conclusion of inferiority/harm will be indicated if the posterior probability for inferiority/harm remains greater than 0.95 at the final analysis. If the trial was not stopped early at an interim analysis due to evidence of inferiority/harm, efficacy will be indicated if the posterior probability for efficacy regarding the primary outcome exceeds a threshold as follows: For studies under this master protocol, the efficacy threshold was selected using statistical simulation to ensure a type-I error probability of 2.5% for each study arm. In all other scenarios, the trial is inconclusive.

6.4 Supplementary Efficacy Estimands

The PO model is attractive for the analysis of ordinal and quantitative response variables, such as the primary outcome, because they directly model the cumulative distribution function from which the mean, median, other percentiles, and cumulative probabilities of the primary outcome, stratified by treatment group, are easily derived.[12] In addition to the odds ratio, the effects of treatment versus placebo will be quantified using the difference in mean, difference in median, and differences in clinically relevant proportions associated with the primary outcome: mortality at day 28: $\Pr(Y = -1|X)$, and oxygen requirement every day until day 28: $\Pr(Y = 0|X)$, adjusted to the modal value for each covariate. These important and clinically meaningful supplementary estimands will be used to describe and communicate the treatment effect. The posterior distribution for each of the supplementary estimands is readily computed using standard Bayesian methods.

6.5 Sensitivity and Supplementary Analyses

Sensitivity and supplemental analyses will be implemented at the final analysis.

Most regression methods, including proportional odds logistic regression, Cox proportional hazards regression, and linear (mean) regression, assume that the effects of the independent variables are consistent across the outcome distribution. Violation of this assumption is a complex situation that implies a heterogeneous treatment effect. For example, the active drug group might experience more frequent extreme outcomes (death and 28 oxygen-free days) versus the placebo group. In this type of situation, much like differential treatment effects observed across participant strata such as sex, no single summary fully describes the treatment effect, and the differential treatment effects must be carefully examined and interpreted in their totality. The *proportional odds assumption* of the PO model specifies that the effect of treatment on the odds that $Y \geq 3$ (measured as an odds ratio versus placebo) is the same relative effect as for $Y \geq 4$. However, even when the PO assumption is strongly violated, the estimated OR remains a simple function of the Wilcoxon-Mann-Whitney U-statistic, namely the probability that a randomly chosen patient on treatment B has a higher response than a randomly chosen

patient on treatment A,[13] the *probability index* or *concordance probability*. In addition, under the null hypothesis, the PO assumption is always satisfied. Thus, statistical testing based on the odds ratio, as estimated using the PO model, has the specified type-I error rate and provides a reasonable global assessment of treatment effectiveness, regardless of violations of the proportional odds assumption. However, derived quantities such as the difference in means may be more sensitive to violations of the PO assumption. Deviations from proportional odds will be examined by separately estimating the odds ratio for each possible dichotomization (that preserves ordering) of the primary outcome (e.g., alive versus dead at day 28, alive and oxygen free for at least 10 days at day 28 versus alive and oxygen free for fewer than 10 days or dead at day 28, etc.), in a planned sensitivity analysis. These analyses will be implemented using the logistic regression method described below (see *Logistic Regression (LogR)*). No hypothesis testing will be implemented regarding the PO assumption. This sensitivity analysis serves the following two purposes: 1) to assess for evidence of violation of the proportional odds assumption and 2) to estimate the differential treatment effects (with 95% credible interval) when the proportional odds assumption is relaxed. The latter provides the information needed to interpret the treatment effects should there be evidence of violation of the proportional odds assumption. In addition, as a key secondary outcome, we will quantify the effect of treatment on 28-day mortality, which directly addresses the possibility that treatment affects 28-day mortality differently than cumulative oxygen use within the first 28 days. This analysis enables us to detect non-proportional effects of the treatment on the two major components of the primary outcome, mortality, and oxygen-free days.

As a supplementary analysis to inform decision-making in the event there is evidence of violation of the PO assumption, we will implement an alternative primary outcome using a partial proportional odds (PPO) model[14], where the effect of the study intervention and each covariate will be allowed to vary across all possible order-preserving dichotomizations of the primary outcome. These covariate effects will be “unconstrained.” However, to ensure estimability of this model, and to shrink toward the PO model, a weakly informative prior will be assigned to shrink each dichotomization effect toward the common effect. The log odds ratio for each dichotomization will have a normal prior distribution centered at the common log odds ratio with standard deviation 0.354, which assigns 95% prior probability that each dichotomization odds ratio falls within a factor of 0.5 and 2.0 of the common odds ratio. The estimated common odds ratio from the PPO model will be evaluated in a manner similar to the common odds ratio in the primary analysis using the PO model. Specifically, we will report the estimated common odds ratio with 95% credible interval and the posterior probability for efficacy. Due to limitations of existing software to implement the PPO model, partially observed values of the primary outcome will be treated as missing.

Analysis of partially observed or missing outcome data requires assumptions regarding the mechanism by which censoring and missing values arise. The likelihood method described above, and other similar methods such as multiple imputation assume that missing values occur at random (i.e., missing at random or MAR). However, because censored and missing values cannot be observed, assumptions about the missingness mechanism are not verifiable. In order to assess the sensitivity of study findings to violations of this assumption, we will conduct additional sensitivity analyses by reproducing the primary analysis under alternative assumptions regarding the mechanism for missing values. Specifically, we will perform sensitivity analyses that vary assumptions about the missing outcomes on the two treatment arms separately. These analyses will consider the following two scenarios: 1 “missing favors inefficacy”) each partially observed primary outcome in the placebo group will be assumed to have taken the highest/best possible value, whereas each partially observed primary outcome in the intervention group will be assumed to have taken the lowest/worst possible value, and 2

“missing favors efficacy”) each partially observed primary outcome in the placebo group will be assumed to have taken the lowest/worst possible value, whereas each partially observed primary outcome in the intervention group will be assumed to have taken the highest/best possible value. These analyses will be implemented using the primary analysis methodology, including an assessment of hypothesis testing outcomes. For any trial under this platform, if there is a conclusion of efficacy at the final analysis, and the conclusion would have been different under the “missing favors inefficacy” scenario, then an additional tipping-point analysis will be implemented to estimate the association between the degree to which missing values must favor inefficacy versus the probability the trial would have failed to conclude efficacy. In these analyses, the partially observed outcomes will be randomly imputed under the assumption that partially observed outcomes favor the inefficacy conclusion by a specified amount. The degree to which the partially observed outcomes favor inefficacy will be encoded using an odds ratio that adjusts the outcome probabilities conditional on the participant covariates, using the maximum a posteriori (MAP) estimate at the final analysis. These probabilities will then be used to randomly sample the outcome for imputation purposes. For partially observed outcomes that exclude some levels of the outcome, the sampling probabilities for the excluded levels will be set to zero and the remaining probabilities normalized to sum to one. After sampling the outcome for all partially observed outcomes, the primary analysis will then be implemented using the imputed outcome data and the study conclusion recorded. This process will be repeated 1000 times and the probability of a trial conclusion other than efficacy will be calculated using a Monte-Carlo estimate. This process will again be repeated for a range of odds ratios encoding the degree to which the partially observed outcomes favor inefficacy. Specifically, this will be guided by two parameters: $\alpha 0$ and $\alpha 1$, where $\alpha 0$ is the log odds ratio of more oxygen free days comparing partially missing versus non-missing placebo arm participants and $\alpha 1$ is the log odds ratio of a higher score comparing partially missing versus non-missing active arm participants. We will vary both parameters across the tipping point analysis within in the following range (-0.5, -0.25, 0.25, 0.5) (i.e., 16 total scenarios). We chose this range of parameters because together they induce a treatment effect in the partially observed participants that ranges from reasonably pessimistic to reasonably optimistic. If β is the observed log odds ratio (active versus control) in the analysis, then the treatment effect in the partially observed participants would range from β-1 to β+1. This range (2 on the log odds ratio scale) is four times the anticipated treatment effect for which the study is powered (i.e., to detect a log odds ratio equal to 0.5, or an odds ratio equal to 1.65). The results of this sensitivity analysis will be summarized graphically.

Co-enrollment in other studies testing COVID-19 therapeutics may occur. Co-enrollment may affect the treatment effect estimates if there is effect modification associated with co-enrollment. We expect co-enrollment to occur in fewer than 5% of patients enrolled in the trial. However, because the decision to co-enroll is not affected by the treatment assignment in ACTIV 4 Host Tissue, co-enrollment will not favor any particular treatment. In addition, due to its rarity, we expect co-enrollment to have little impact on the estimated treatment effects, even when there is effect modification.

Differential treatment effect, also referred to as heterogeneity of treatment effect, refers to differences in efficacy as a function of pre-existing patient characteristics such as baseline variables. This is often assessed by forming subgroups or using an interaction analysis. Supplemental interaction analyses will be implemented to examine the potential for differential treatment effect. Differential treatment effect will be examined in strata defined by (but not limited to) respiratory support category at enrollment, status of co-enrollment in an open label clinical trial of antiplatelet agents (ACTIV 4a), age category, SARS-CoV-2 vaccination status, passive immunity status, co-enrollment in other studies, and concomitant use of study drug and

other medications during the study drug administration period. These analyses will be implemented using a modified version of the primary analysis method, where the treatment effect will be estimated separately for each level of the stratification variable. Stratum-specific treatment effect estimates will be presented with 95% Bayesian confidence interval. No formal hypothesis testing will be implemented for these analyses. Studies under this master protocol will be sized only for assessing efficacy using the primary analysis. Thus, there may be inadequate power to examine differential treatment.

6.6 Sample Size and Decision Thresholds

The maximum number of participants to be enrolled in sub studies under the Master Protocol is 600 participants per trial, resulting in approximately 300 patients per active treatment arm, and 300 patients in the matching placebo arm. The placebo arm will be shared across all active treatment arms. We expect placebo participants to continue to accrue for as long as there are additional treatments to test and cases to enroll.

Type-I error and power regarding the analysis of the primary outcome was assessed based on the pooled (across all active and placebo arms) distribution of the primary outcome among the first 100 participants to complete follow-up and monitoring. The efficacy threshold was identified using statistical simulation under the null hypothesis to ensure the study operating characteristics achieve design specifications. Pooled and blinded summaries of oxygen-free days at day 28 were used to approximate the distribution of the oxygen free days in the placebo group. Based on these data, the anticipated frequency distribution, mean, and median of oxygen-free days (OFDs) for the placebo group, and for the treatment group under hypothetical effect sizes computed using the PO model are displayed in the table below.

		**Inferiority**		**Superiority**						
**OFDs / Odds Ratio**	**Placebo**	**0.67**	**0.80**	**1.40**	**1.45**	**1.50**	**1.55**	**1.60**	**1.65**	**1.70**
Mean	8.8	6.6	7.5	10.8	11.1	11.3	11.5	11.7	11.9	12.0
Median	0	0	0	6.5	7.5	9.0	10.0	10.5	12.5	14.5
P(OFDs >= 22)	0.19	0.14	0.16	0.25	0.26	0.26	0.27	0.28	0.29	0.29
Proportion:										
-1 (death)	0.235	0.316	0.279	0.181	0.176	0.171	0.166	0.162	0.158	0.154
0	0.296	0.314	0.309	0.268	0.264	0.261	0.257	0.254	0.251	0.247
1	0.006	0.006	0.006	0.006	0.006	0.006	0.006	0.006	0.006	0.006
---	---	---	---	---	---	---	---	---	---	---
27	0.050	0.034	0.041	0.069	0.072	0.074	0.076	0.078	0.081	0.083
28	0.000	0.000	0.000	0.000	0.000	0.000	0.000	0.000	0.000	0.000

Based on these data and effect size scenarios, a series of statistical simulations were implemented to examine the operating characteristics of the statistical study design described above, including the plan for randomization, statistical analysis method, interim analysis, and final assessments of efficacy using the odds ratio. In each simulation, participant age group, sex, and baseline WHO COVID severity score were randomly sampled with replacement from the values observed, and their effects on the primary outcome were simulated to match the estimated effects of age group, sex, and WHO score on the primary outcome among the first 100 participants. In order to assess the potential impact of attrition and loss-to-follow-up, partially observed oxygen free days were simulated to match the observed frequency of partially observed outcomes, which occurred in 12% of the first 100 participants. To encode attrition, a subset of the simulated study participants was selected at random, each with probability 0.12.

The primary outcome for each selected participant was encoded as partially observed by assuming that oxygen free days may have taken any value between -1 and a randomly sampled value ranging from the simulated oxygen free days to 28. For example, if the simulated oxygen free days is 10, then a value between 10 and 28 is sampled uniformly at random and this value is treated at the upper limit for the partially observed oxygen free days. This pattern of partially observed oxygen free days closely resembles the patterns observed among the first 100 participants. All simulation analyses, including those associated with interim and final assessment of efficacy and inferiority were implemented using the methods described above for the analysis of the primary outcome.

Simulation under the null hypothesis was used to select the efficacy threshold for the final analysis. The efficacy threshold was selected to ensure no more than 2.5% type-I error. In this simulation, 10000 replicates were used to ensure ~0.31% simulation margin of error in estimating the type-I error rate. The efficacy threshold was identified as 0.976. A final analysis will occur once enrollment, follow-up, and the required monitoring are completed for all participants. Should additional data be collected after enrollment is halted at an interim analysis, the final analysis will incorporate this additional data. If enrollment was halted at an interim analysis due to evidence of inferiority/harm, a conclusion of inferiority/harm will be indicated if the posterior probability for inferiority/harm remains greater than 0.95 at the final analysis. If the trial was not stopped early at an interim analysis due to evidence of inferiority/harm, efficacy will be indicated if the posterior probability for efficacy regarding the primary outcome exceeds 0.976 at the final analysis. If neither condition is met for a conclusion of efficacy or inferiority/harm at the final analysis, the trial is inconclusive. The efficacy and inferiority/harm thresholds will be applied as described in the table below.

**Analysis**	**Condition**	**Action**
Interim analysis	Inferiority/harm probability > 0.950	Halt enrollment
Final analysis	Inferiority/harm probability ≤ 0.950 at all interim analyses and efficacy probability > 0.976	Conclude efficacy
Final analysis	Inferiority/harm probability > 0.950	Conclude inferiority/harm

Using the selected efficacy and inferiority/harm thresholds, the results of 10000 simulations under the null hypothesis, and 1000 simulations per inferiority/efficacy scenario are summarized in the table below. In these simulations, the type-I error probability was 2.47%. The frequency of stopping early for inferiority under the null was 8.6% (5.3% at the first interim analysis, and 3.2% at the second interim analysis). *A maximum sample size of 600 participants per trial provides greater than 85% power to detect an odds ratio of 1.65, corresponding to a 3.1-day difference in mean OFDs, and a 7.8 percentage point reduction in 28-day mortality.* Differences larger than 2 ventilator-free days on average have been considered clinically important in prior trials.[2-4] *Thus, the minimum detectable effect with 85% power (MDE85) is an odds ratio of 1.65.* The frequency of stopping early for inferiority when there was an effect larger than OR=1.40 was <1%. When the simulated treatment was inferior/harmful relative to placebo, at OR=0.67, a conclusion of inferiority/harm occurred in 83.3% of simulated trials (39.1%at the first interim, 27.9% at the second interim, and 16.3% at the final analysis), and the average half-sample size was 193.9 per arm.

	**Null**	**Inferiority**		**Superiority**						
**OFDs / Odds Ratio**	**1.00**	**0.67**	**0.80**	**1.40**	**1.45**	**1.50**	**1.55**	**1.60**	**1.65**	**1.70**
Pr(Efficacy)	**0.025**	0.000	0.001	**0.552**	**0.631**	**0.705**	**0.782**	**0.826**	**0.856**	**0.893**

	Null	Inferiority		Superiority						
**OFDs / Odds Ratio**	**1.00**	**0.67**	**0.80**	**1.40**	**1.45**	**1.50**	**1.55**	**1.60**	**1.65**	**1.70**
Pr(Inferiority)	0.108	0.833	0.508	0.003	0.002	0.001	0.000	0.000	0.001	0.000
Pr(Inconclusive)	0.867	0.167	0.491	0.445	0.366	0.294	0.218	0.173	0.143	0.107
Average(N/2)	286.1	193.9	242.0	299.4	299.8	299.8	300.0	300.0	299.8	300.0

In order to characterize the effect of uncertainty in the distribution of the OFD outcome on the type-I error probability, simulations under the null hypothesis were twice repeated assuming a “mild” and “severe” distribution for the OFD outcome. The mild and severe distributions were selected such that the unadjusted mortality rate ranged ± 3% relative to the initial simulation. The results of 1000 simulations in each of the mild placebo and severe placebo scenarios are summarized in the table below. In these simulations, the type-I error probability was 2.5% and 2.3%.

	Severe	Mild
	**OR = 1.00**	**OR = 1.00**
Mortality rate	0.266	0.206
Pr(Efficacy)	**0.023**	**0.025**
Pr(Inferiority)	0.0.119	0.117
Pr(Inconclusive)	0.858	0.858
Average(N)	284.0	286.4

Prior to the start of enrollment, initial sample size assessments were based on pooled and blinded summaries of OFDs from the PassItOn (convalescent plasma) trial of patients hospitalized for COVID-19. The inclusion and exclusion criteria for PassItOn are similar to that for ACTIV 4 Host Tissue. In these initial assessments, the estimated MDE85 was OR=1.55. Statistical power was subsequently reassessed using OFDs summaries in the first 100 participants enrolled in ACTIV 4 Host Tissue, which demonstrated a more severe distribution relative to PassItOn participants (23.6% vs 17.6% mortality). The estimated MDE85 was OR=1.65 at the time of sample size reassessment. However, additional information from blinded summaries of the first 200 enrolled participants are consistent with the distribution of OFDs observed in PassItOn (18.6% vs 17.6% mortality). After discussion of these findings among the blinded study investigators and study sponsor, it was determined that statistical power was sufficient and no sample size adjustment was warranted.

7 ANALYSIS OF SECONDARY, EXPLORATORY, AND SAFETY OUTCOMES

Final analysis of the secondary, exploratory, and safety outcomes will be implemented separately for each study arm by comparing each active drug group with the corresponding pooled placebo comparator group. The effect of active agent versus placebo on the odds of binary and ordinal outcomes will be quantified using logistic and proportional odds logistic regression. Quantitative outcomes will be analyzed using a linear regression method. In order to preserve consistency across statistical analyses, we will uniformly apply a Bayesian approach using flat priors. Odds ratio, hazard ratio, and differences in mean estimates will be presented with a 95% credible interval.

7.1 Statistical Methods for Secondary, Exploratory, and Safety Analyses

The methods described below will be applied uniformly to the examine the effect of each active drug versus the placebo comparator on the secondary, exploratory, and safety outcomes, as appropriate.

7.1.1 Proportional Odds Logistic Regression (POLR)

Ordinal secondary, exploratory, and safety outcomes will be analyzed using a method similar to that described above for the analysis of the primary outcome, using proportional odds logistic regression (POLR), and adjusting for participant age group, sex, and WHO COVID ordinal severity at baseline. The effect of the active drug versus placebo will be presented using an odds ratio which quantifies the treatment effect on the odds of greater values of the ordinal outcome. The odds ratio will be presented with 95% credible interval. A flat prior distribution will be used for all model parameters. The posterior distribution for the log odds ratio will be approximated using the Laplace method. All statistical inferences about the odds ratio will be made using this method. The proportional odds assumption means that the odds-ratio has the same interpretation for all dichotomizations (that preserve ordering) of the ordinal outcome. The repeated dichotomization method, as described for the analysis of the primary outcome, will be used to assess for severe violations of the proportional odds assumptions. Missing or partially observed outcomes will be handled using the likelihood method as described for the primary analysis (see *Loss to Follow-up, Censoring, and Intercurrent Events*).

7.1.2 Logistic Regression (LogR)

Binary secondary, exploratory, and safety outcomes will be analyzed using logistic regression (LogR), and adjusting for participant age group, sex, and WHO COVID ordinal severity at baseline. The effect of the active drug versus placebo will be presented using an odds ratio which quantifies the treatment effect on the odds of outcome occurrence. The odds ratio will be presented with 95% credible interval. In addition, to facilitate clinical interpretability and meaningfulness, the difference in proportions corresponding to the most common (modal) values of the adjustment variables will be presented with 95% credible interval. A flat prior distribution will be used for all model parameters. The posterior distribution for the log odds ratio will be approximated using the Laplace method. All statistical inferences about the odds ratio and other posterior quantities will be made using this method. Missing outcomes will be handled using the likelihood method as described for the primary analysis (see *Loss to Follow-up, Censoring, and Intercurrent Events*).

7.1.3 Linear Regression (LinR)

Quantitative exploratory will be analyzed using linear regression (LinR), and adjusting for participant age group, sex, and WHO COVID ordinal severity at baseline. The effect of the active drug versus placebo will be presented using a difference in means. The difference in means will be presented with 95% credible interval. A flat prior distribution will be used for all model parameters. The posterior distribution for the difference in means will be approximated using the Laplace method. All statistical inferences about the difference in means will be made using this method. Graphical regression diagnostics, including normal Q-Q plots, will be used to assess for severe violations of the linear regression assumptions. Missing exploratory outcomes will be omitted from linear regression analyses.

7.1.4 Key Secondary Outcome Testing Procedure

A fixed-sequence testing approach will be used to preserve the type-I error rate across tests of the primary and key secondary outcomes. The key secondary outcomes will be tested in the specified order (see *Secondary Outcomes*). This approach provides strong control of the

familywise type-I error rate for the family of primary and key secondary outcomes. No other formal hypothesis tests will be made regarding the secondary, exploratory, or safety outcomes.

All key secondary outcomes use Bayesian logistic regression with a flat prior. Thus, the log odds ratio estimate is also a maximum likelihood estimate (MLE). At the final analysis (only) for each arm and key secondary outcome, efficacy will be indicated using a one-sided likelihood-based Wald test, to ensure a type-I error probability of 2.5% for each test. Specifically, a one-sided test of the null hypothesis (log OR = 0) will be computed by approximating the asymptotic distribution of the MLE under the null hypothesis: a Gaussian distribution with mean zero and variance equal to the inverse observed Fisher information. For descriptive purposes, evidence for efficacy will also be quantified using the posterior probability that the efficacy odds ratio is greater than one (i.e., treatment is associated with greater odds of a favorable outcome). This is denoted the “posterior probability for efficacy” or $P(\mathrm{OR} > 1|\mathrm{Data})$, where OR represents the odds ratio, and Data represents the mITT analysis dataset.

7.2 Analysis of Safety, Adherence, and Retention Outcomes for DSMB Review

Monitoring and reporting of safety events will be conducted continuously as described in the Data and Safety Monitoring Plan. Records will undergo monitoring for a two-week period (at minimum) prior to interim analysis for inferiority or futility. However, all records, regardless of monitoring status, will be used in enrollment, demographic, and safety summaries for DSMB safety reporting. Agent-specific safety and toxicity endpoints (if any) are detailed in that therapy’s appendix. The frequencies of PSESEs, adverse events, mortality, and other safety endpoints will be reported. Screening, enrollment, withdrawal, loss-to-follow-up, mortality, study completion, hospitalization status and discharge location will be summarized in a similar manner. All safety-related protocol violations will be listed in the DSMB report. Receipt of planned therapy and adverse events will be recorded on case report forms and monitored continuously. Study drug stoppages and adverse events will be summarized and reported to the DSMB.

8 DATA FLOW, SHARING, AND ARCHIVING

8.1 Requests for secondary use of the data

Requests for secondary use of study data must adhere to review, approval, and provision processes developed by ACTIV 4 Host Tissue leadership and must comply with all applicable rules and regulations. All study data will be de-identified prior to sharing for secondary use.

8.2 Data flow for final and interim analyses

All data necessary for interim analyses, final analyses, and DSMB reporting will be exported from the EDC using the REDCap API. A custom R script will be used to both export the data and perform the interim analyses.

8.3 Archival data model

Data will remain in the production database. At the time of data locking, all users will be moved to read only access or removed, or as specified in the Data Management Plan.

8.4 Final analysis procedure

Once a study arm has completed enrollment, follow-up, and monitoring for all participants, all records that contribute to final analyses will be locked. Final analysis will be executed promptly after data lock, regardless of the status of other study arms. Blinded personnel will remain

blinded to the active/placebo status for individual participants until all arms that share blinded information with the completed arm have also been completed and their records locked. Final analyses will be executed by unblinded personnel only. Reporting of final analyses should avoid revealing the blinded treatment assignment for individual participants.

9 REFERENCES

1. Weins BL. A fixed sequence Bonferroni procedure for testing multiple endpoints. Pharmaceutical Statistics, 2003; 2:211-215.
2. Schoenfeld DA, Bernard GR, ARDS Network. Statistical evaluation of ventilator-free days as an efficacy measure in clinical trials of treatments for acute respiratory distress syndrome. Crit Care Med. 2002 Aug;30(8):1772–1777. PMID: 12163791
3. National Heart, Lung, and Blood Institute Acute Respiratory Distress Syndrome (ARDS) Clinical Trials Network, Wiedemann HP, Wheeler AP, Bernard GR, Thompson BT, Hayden D, deBoisblanc B, Connors AF, Hite RD, Harabin AL. Comparison of two fluid-management strategies in acute lung injury. N Engl J Med. 2006 Jun 15;354(24):2564–2575. PMID: 16714767
4. Laterre P-F, Berry SM, Blemings A, Carlsen JE, François B, Graves T, Jacobsen K, Lewis RJ, Opal SM, Perner A, Pickkers P, Russell JA, Windeløv NA, Yealy DM, Asfar P, Bestle MH, Muller G, Bruel C, Brulé N, Decruyenaere J, Dive A-M, Dugernier T, Krell K, Lefrant J-Y, Megarbane B, Mercier E, Mira J-P, Quenot J-P, Rasmussen BS, Thorsen-Meyer H-C, Vander Laenen M, Vang ML, Vignon P, Vinatier I, Wichmann S, Wittebole X, Kjølbye AL, Angus DC, SEPSIS-ACT Investigators. Effect of Selepressin vs Placebo on Ventilator- and Vasopressor-Free Days in Patients With Septic Shock: The SEPSIS-ACT Randomized Clinical Trial. JAMA. 2019 Oct 2; PMCID: PMC6802260
5. Self WH, Semler MW, Wanderer JP, Wang L, Byrne DW, Collins SP, Slovis CM, Lindsell CJ, Ehrenfeld JM, Siew ED, Shaw AD, Bernard GR, Rice TW, SALT-ED Investigators. Balanced Crystalloids versus Saline in Noncritically Ill Adults. N Engl J Med. 2018 01;378(9):819–828. PMCID: PMC5846618
6. Self WH, Semler MW, Leither LM, Casey JD, Angus DC, Brower RG, Chang SY, Collins SP, Eppensteiner JC, Filbin MR, Files DC, Gibbs KW, Ginde AA, Gong MN, Harrell FE, Hayden DL, Hough CL, Johnson NJ, Khan A, Lindsell CJ, Matthay MA, Moss M, Park PK, Rice TW, Robinson BRH, Schoenfeld DA, Shapiro NI, Steingrub JS, Ulysse CA, Weissman A, Yealy DM, Thompson BT, Brown SM, National Heart, Lung, and Blood Institute PETAL Clinical Trials Network, Steingrub J, Smithline H, Tiru B, Tidswell M, Kozikowski L, Thornton-Thompson S, De Souza L, Hou P, Baron R, Massaro A, Aisiku I, Fredenburgh L, Seethala R, Johnsky L, Riker R, Seder D, May T, Baumann M, Eldridge A, Lord C, Shapiro N, Talmor D, O'Mara T, Kirk C, Harrison K, Kurt L, Schermerhorn M, Banner-Goodspeed V, Boyle K, Dubosh N, Filbin M, Hibbert K, Parry B, Lavin-Parsons K, Pulido N, Lilley B, Lodenstein C, Margolin J, Brait K, Jones A, Galbraith J, Peacock R, Nandi U, Wachs T, Matthay M, Liu K, Kangelaris K, Wang R, Calfee C, Yee K, Hendey G, Chang S, Lim G, Qadir N, Tam A, Beutler R, Levitt J, Wilson J, Rogers A, Vojnik R, Roque J, Albertson T, Chenoweth J, Adams J, Pearson S, Juarez M, Almasri E, Fayed M, Hughes A, Hillard S, Huebinger R, Wang H, Vidales E, Patel B, Ginde A, Moss M, Baduashvili A, McKeehan J, Finck L, Higgins C, Howell M, Douglas I, Haukoos J, Hiller T, Lyle C, Cupelo A, Caruso E, Camacho C, Gravitz S, Finigan J, Griesmer C, Park P, Hyzy R, Nelson K, McDonough K, Olbrich N, Williams M, Kapoor R, Nash J, Willig M, Ford H, Gardner-Gray J, Ramesh M, Moses M, Ng Gong M, Aboodi M, Asghar A, Amosu O, Torres M, Kaur S, Chen J-T, Hope A, Lopez B, Rosales K, Young You J, Mosier J, Hypes C, Natt B, Borg B, Salvagio Campbell E, Hite RD,

Hudock K, Cresie A, Alhasan F, Gomez-Arroyo J, Duggal A, Mehkri O, Hastings A, Sahoo D, Abi Fadel F, Gole S, Shaner V, Wimer A, Meli Y, King A, Terndrup T, Exline M, Pannu S, Robart E, Karow S, Hough C, Robinson B, Johnson N, Henning D, Campo M, Gundel S, Seghal S, Katsandres S, Dean S, Khan A, Krol O, Jouzestani M, Huynh P, Weissman A, Yealy D, Scholl D, Adams P, McVerry B, Huang D, Angus D, Schooler J, Moore S, Files C, Miller C, Gibbs K, LaRose M, Flores L, Koehler L, Morse C, Sanders J, Langford C, Nanney K, MdalaGausi M, Yeboah P, Morris P, Sturgill J, Seif S, Cassity E, Dhar S, de Wit M, Mason J, Goodwin A, Hall G, Grady A, Chamberlain A, Brown S, Bledsoe J, Leither L, Peltan I, Starr N, Fergus M, Aston V, Montgomery Q, Smith R, Merrill M, Brown K, Armbruster B, Harris E, Middleton E, Paine R, Johnson S, Barrios M, Eppensteiner J, Limkakeng A, McGowan L, Porter T, Bouffler A, Leahy JC, deBoisblanc B, Lammi M, Happel K, Lauto P, Self W, Casey J, Semler M, Collins S, Harrell F, Lindsell C, Rice T, Stubblefield W, Gray C, Johnson J, Roth M, Hays M, Torr D, Zakaria A, Schoenfeld D, Thompson T, Hayden D, Ringwood N, Oldmixon C, Ulysse C, Morse R, Muzikansky A, Fitzgerald L, Whitaker S, Lagakos A, Brower R, Reineck L, Aggarwal N, Bienstock K, Freemer M, Maclawiw M, Weinmann G, Morrison L, Gillespie M, Kryscio R, Brodie D, Zareba W, Rompalo A, Boeckh M, Parsons P, Christie J, Hall J, Horton N, Zoloth L, Dickert N, Diercks D. Effect of Hydroxychloroquine on Clinical Status at 14 Days in Hospitalized Patients With COVID-19: A Randomized Clinical Trial. JAMA. 2020 Dec 1;324(21):2165–2176. PMCID: PMC7653542

7. Harrell, Frank. General COVID-19 Therapeutics Trial Design [Internet]. 2020. Available from: https://hbiostat.org/proj/covid19/statdesign.html
8. Zhenming S, McCullagh P. Laplace approximation of high dimensional integrals. Journal of the Royal Statistical Society. 1995;57(4):749–760.
9. Doob J. The limiting distributions of certain statistics. The Annals of Mathematical Statistics. 1935 Sep 1;6(3):160–169.
10. Simpson DG, Carroll RJ, Zhou H, Guth DJ. Interval Censoring and Marginal Analysis in Ordinal Regression. Journal of Agricultural, Biological, and Environmental Statistics. 1996 Sep;1(3):354.
11. Food and Drug Administration. E9(R1) Statistical Principles for Clinical Trials: Addendum: Estimands and Sensitivity Analysis in Clinical Trials [Internet]. 2017. Available from: https://www.fda.gov/media/108698/download
12. Liu Q, Shepherd BE, Li C, Harrell FE. Modeling continuous response variables using ordinal regression. Stat Med. 2017 Nov 30;36(27):4316–4335. PMCID: PMC5675816
13. Harrell, Frank. Violation of Proportional Odds is Not Fatal | Statistical Thinking [Internet]. 2020. Available from: https://www.fharrell.com/post/po/
14. P Peterson, B. and Harrell Jr, F.E., 1990. Partial proportional odds models for ordinal response variables. Journal of the Royal Statistical Society: Series C (Applied Statistics), 39(2), pp.205-217.

10 APPENDIX: ALGORITHM TO COMPUTE PRIMARY OUTCOME

The primary outcome is oxygen-free days at study day 28. It can take values -1, 0, 2, …, 27, 28. When computing oxygen free days, the “outcome” for each participant should be a length 30 vector of zeros and ones that indicate which of the 30 possible values (-1, 0, 2, …, 27, 28) that OFDs could take for that participant. This representation allows for arbitrary censoring of the outcome. For example [0,1,1,0,0,...,0] indicates that OFDs could be either 0 or 1. If there is loss-to-follow-up, withdrawal, or missing follow-up information, there can be interval censoring. The algorithm below is designed to compute OFDs in this representation.

- If participant was deceased by study day 28, OFDs is [1,0,0,0,0,...,0]
- For study day 1 through 28, compute whether or not supplemental oxygen was used (code with “yes” or “no”), or if supplemental oxygen use was uncertain (code with “?”).
    - For our purposes supplemental oxygen means oxygen use that exceeds any pre-enrollment home oxygen use. Home oxygen use is recorded in the “Medical History” form in variables mhco2, mhio2, and the amount (L/m) in field home_ox. If a participant had not used pre-enrollment home oxygen, then it should be assumed that all hospital and post-discharge use of oxygen counts against oxygen-free days. If a participant had used pre-enrollment home oxygen, then only the supplemental oxygen use that exceeds the amount used at home should count against oxygen-free days. If the participant is in the inpatient phase of the study and using standard supplemental oxygen (o2type = “O2 by mask or nasal prongs”), then the L/m recorded on the vitals signs form (o2_lpm_cannula_sofa) must exceed the amount used at home (home_ox). If hospital oxygen use takes any other value except “No O2 therapy” and “O2 by mask or nasal prongs”, then that study day should count against oxygen free-days.
    - If the participant is in the outpatient phase of the study (i.e., after discharge from the enrollment admission or after 28 days, whichever comes first), but is not hospitalized, then only the post-discharge home oxygen use that exceeds the amount used at home prior to enrollment (if any) will count against oxygen-free days. The phone script and outpatient form are designed to record only the home oxygen use that exceeds any pre-hospitalization oxygen use.
    - If the participant is in the outpatient phase of the study, but is hospitalized, the branching logic on the outpatient form determines whether the participant had used oxygen. Any hospital oxygen use during the outpatient phase counts against oxygen-free days.
    - If the preceding calculations cannot be made for any particular study day, then the supplemental oxygen status is “?” for that study day.
- The preceding step results in “yes”, “no”, or “?” for each study day 1 through 28.
    - If there are no “?” values, then OFDs is 28 minus the number of days between and including the days of the first “yes” and the last “yes”.
    - If there are “?” before the first “yes” or after the last yes, then OFDs is partially observed and multiple values are possible. To compute the possible values, consider each possible pair of first ‘yes’ and last ‘yes’ days, and compute the associated OFDs.

OFDs should be represented as a vector of length 30, one element for each value that OFDs can take: -1, 0, 1, …, 27, 28. There should be a 1 for each element that OFD that is possible for this participant, and a zero otherwise. The -1 (first) element should take a value 0 if the participant was known to be alive at day 28 and 1 otherwise.

11 APPENDIX: CUMULATIVE LOGIT MODEL

11.1 Model Formulation

The cumulative logit model can be written in terms of the covariates X and an ordinal outcome variable G, where probabilities of outcome value g or smaller are modeled as follows

$$\Pr(G \leq g|X) = \mathrm{expit}\left(\alpha_g - X\beta\right). \#(1)$$

Without loss of generality, an outcome with p levels may be coded using the first p integers, such that g may take on the values $1, \dots, p$. In the expression above, α_g is a scalar intercept, expit is the logistic (inverse logit) transformation, and the vector X contains coded baseline covariates and the active/placebo treatment indicator. The model has intercepts for each of the first $p-1$ outcome levels, and the intercepts must be ordered: $\alpha_1 \leq \alpha_2 \leq \cdots \leq \alpha_{p-1}$. The ordering of intercepts ensures that the probabilities $\Pr(G \leq g|X)$ are monotonically increasing in g. The parameter vector β represents the log odds ratios (OR) associated with the effects of covariates and group assignment. Specifically, the group assignment odds ratio represents the relative effect of treatment versus placebo on the odds $\Pr(G > g|X)/(1 - \Pr(G > g|X))$, for each of the first $p-1$ values that G may take.

The $p-1$ linear predictors $\alpha_g - X\beta$ represent the logit transformed cumulative probabilities associated with the first $p-1$ levels of the ordinal outcome, adjusted for the effects of covariates X. The probabilities that the outcome takes a specific value g, adjusted for covariates X, is derived as follows:

$$\Pr(G = g|X) = \text{expit}\left(\alpha_g - X\beta\right) - \text{expit}\left(\alpha_{g-1} - X\beta\right), \#(2)$$

where $\text{expit}(\alpha_0 - X\beta)$ and $\text{expit}\left(\alpha_p - X\beta\right)$ are defined to be 0 and 1, respectively. When there are partially observed ordinal outcomes, it is convenient to recode the outcome as a vector $Y = [Y_1, \dots, Y_p]$, such that $Y_g = 1$ if $G = g$ or when g is one of the values that G might have taken if the outcome were fully observed, and $Y_g = 0$ otherwise. Thus, the cumulative logit model may be written as follows

$$\Pr\left(Y_1 = 1 \cup Y_2 = 1 \cup \cdots \cup Y_g = 1 \middle| X\right) = \Pr(G \leq g|X) = \text{expit}\left(\alpha_g - X\beta\right). \#(3)$$

Denote a sample of covariate vectors $x_1, \dots, x_N$ and outcomes $g_1, \dots, g_N$, and corresponding outcome vectors $y_1, \dots, y_N$, where $y_i = [y_{i1}, \dots, y_{ip}]$. Using this representation, partially observed outcomes are encoded by assigning a value 1 to each element of y_i that the outcome g_i might have taken if fully observed. For example, if g_i might have taken values 1 or 2, but other values were not possible, then y_i would be coded $y_i = [1, 1, 0, \dots, 0]$. Further denote the collection of model parameters $\theta = \left[\alpha_1, \dots, \alpha_{p-1}, \beta\right]$. Using this notation, the observed data likelihood is as follows:

$$L(\theta|y_1 \dots y_N, x_1 \dots x_N) = \prod_{i=1}^{N} L_i(\theta|y_i, x_i) = \prod_{i=1}^{N} \sum_{j=1}^{p} I\left(y_{ij} = 1\right) \Pr(Y_j = 1\,|X = x_i), \#(4)$$

where $I(\cdot)$ is the indicator function that takes a value 1 when its argument is true, and 0 otherwise.

In a Bayesian analysis, the posterior density function is proportional to the likelihood function multiplied by the prior density function as follows:

$$P(\theta|y_1 \dots y_N, x_1 \dots x_N) \propto L(\theta|y_1 \dots y_N, x_1 \dots x_N) P(\theta) \#(5)$$

A flat prior distribution, where $P(\theta) \propto 1$, is used for all model parameters. Thus, the posterior density is proportional to the likelihood function.

Each supplemental estimand is a treatment difference in one of the following summaries of the adjusted outcome distribution: mean, median, proportion experiencing mortality by day 28, and proportion receiving oxygen every day until day 28. Supplemental estimands are computed using the probabilities expressed in equations (1) and (2) above, adjusted to the modal value for each covariate: age group 31-64, WHO COVID-19 Score value 4 (hospitalized receiving oxygen via nasal cannula), and male sex. The adjusted means are computed as the linear combination of all possible outcome values and their associated adjusted probabilities. The adjusted medians are computed as the smallest value of g such that the $\Pr(G \geq g|X) \geq 0.5$. The adjusted proportions of participants experiencing mortality by day 28 or oxygen requirement every day until day 28 are computed as $\Pr(G = -1|X)$ and $\Pr(G = 0|X)$, respectively.

11.2 Model-based Statistical Inferences

The posterior distribution for the log odds ratio and any other required parameter is approximated using the Laplace method. A flat prior ensures the Laplace-approximated posterior distribution is identical to the approximate sampling distribution of the maximum likelihood estimate for θ; in both cases a normal distribution centered at the estimate (i.e., the maximum likelihood estimate or equivalently the maximum *a posteriori* estimate) with variance-covariance equal to the negative inverse Hessian of the log likelihood function (inverse observed Fisher information) evaluated at the estimate (see "Appendix: Laplace Approximation"). All statistical inferences about the odds ratio and derivative quantities (including all supplementary estimands) will be made using this method.

11.3 Model Fitting and Computation

The cumulative logit model is implemented in the R code file "clm_model.R". Readers should examine the `clm_fit` function first, which is the entry point for model fitting, and then examine other functions as they are called by `clm_fit`. The function `clm_fit` takes as arguments the matrix of coded covariates `x`, and a matrix of coded outcomes `y`. Each matrix has one row per record (i.e., study participant). The covariate matrix has one column per coded covariate (e.g., age group has three levels and thus requires two columns to distinguish the levels), and the outcome matrix has one column per value that the outcome might take. The cells of the outcome matrix `y` contain the values y_{ij} as defined above (see "Model Formulation").

In practice, when one or more levels of an ordinal outcome are not observed in the analysis data set, some of the model intercepts are not estimable (i.e., there is no unique set of model intercepts that maximizes the likelihood/posterior density function). To overcome this, each outcome level is characterized as "estimable" if there is at least one record in the analysis data set where that level is observed and no other level was possible (i.e., ignoring partially observed outcomes), and "not estimable" otherwise. Levels of the outcome that are not estimable are collapsed with the nearest adjacent estimable level to form a new level, e.g., levels 3, 4, and 5 may be collapsed to form level "3|5". When levels are collapsed, if any collapsed level was possible as part of a partially observed outcome, then the collapsed level is considered possible as well. This functionality is implemented by the function `clm_collapse`, which is called by `clm_fit` prior to any model fitting.

The estimate of θ is found by maximizing the log of the posterior density function (i.e., a maximum *a posteriori* estimate, or MAP for short) defined in expression (5). Note that the normalizing constant in expression (5) is not needed to identify the MAP estimate, nor is it necessary to form a Laplace approximation to the posterior density. The estimate of θ is found using an iterative optimization algorithm, and the associated observed Fisher information is estimated using a finite difference method. These calculations are implemented using the R function `optim`, which uses the quasi-Newton "BFGS" method (Byrd, Lu, Nocedal, and Zhu,

1995, A limited memory algorithm for bound constrained optimization. SIAM Journal on Scientific Computing, 16, 1190–1208. doi: 10.1137/0916069), and is built-in as part of the “stats” package for R (R Core Team, 2022, R: A language and environment for statistical computing. R Foundation for Statistical Computing, Vienna, Austria. URL https://www.R-project.org/). The initial values for β are set to zero. Initial values for the model intercepts are generated by first calculating the fraction of each observed outcome level (i.e., an initial estimate of $\Pr(G = g|X)$ where $\beta = 0$), and then applying the inverse of expression (2) as follows:

$$\alpha_g^{\text{init}} = \text{logit}\left(\sum_{k=1}^{g} \frac{\sum_i y_{ik}}{\sum_i \sum_j y_{ij}}\right). \#(6)$$

The initial values calculations for the model intercepts are implemented by the function `clm_alpha_init`. Starting at the initial values, the `optim` function iteratively maximizes the `clm_optim` function, which computes the log of the posterior density function given by expression (5). The `clm_optim` function calls the `clm_loglik` and `clm_logpri` functions, which evaluate the log of the likelihood function given by expression (4) and log of the prior density function (defined to be zero for a flat prior), respectively. The `clm_loglik` function calls `clm_predict` which computes, for each record, the linear predictors, $\alpha_g - X\beta = \text{logit}\Pr(G \leq g|X)$, and the associated covariate adjusted probabilities for each ordinal outcome level $\Pr(G = g|X)$. The `clm_predict` function calls `alphs_to_probs` to convert the logit cumulative probabilities to level specific probabilities according to expression (2). The `probs_to_alphs` function computes the inverse of `alphs_to_probs`.

The `clm_fit` function returns a model fit object that contains a model convergence assessment, the MAP estimate for θ, and the estimated Hessian of the log posterior density function evaluated at the estimate. The MAP estimate and Hessian are sufficient to define the Laplace (Normal) approximation to the posterior density, and are used to compute posterior cumulative probabilities as follows

$$\Pr(\theta_k \leq q|y_1 \ldots y_N, x_1 \ldots x_N) = \Phi\left(\frac{q-\hat{\theta}_k}{\sqrt{[-H^{-1}]_{kk}}}\right),$$

where H is the estimated Hessian, $\hat{\theta}$ is the MAP estimate, and Φ is the standard normal cumulative density function. This is implemented by the `clm_ppost` function for specified scalar elements θ_k. Notably, this function is used to compute the posterior probabilities used for decision-making at the interim and final analyses.

For supplementary estimands, $g(\theta)$, that are smooth scalar functions of θ (i.e., treatment difference in the mean of the primary outcome, and treatment difference in the probabilities associated with outcome categories -1 and 0), the posterior distribution will be approximated using the delta method, for example, to compute posterior cumulative probabilities as follows:

$$\Pr(g(\theta) \leq q|y_1 \ldots y_N, x_1 \ldots x_N) = \Phi\left(\frac{q-g(\hat{\theta})}{\sqrt{g'(\hat{\theta})^T[-H^{-1}]g'(\hat{\theta})}}\right),$$

Where $g'(\hat{\theta})$ is the gradient of $g(\cdot)$ evaluated at $\hat{\theta}$, which is approximated numerically using a finite difference method. For non-smooth scalar functions of θ (i.e., treatment difference in the

median of the primary outcome), the posterior distribution will be identified using a Monte Carlo method; by generating 10000 realizations from the posterior distribution for θ, and evaluating the supplementary estimand using those realizations. For either approach, an equal-tailed, level $(1-\alpha)$ credible interval will then be identified by selecting the $\alpha/2$ and $1-\alpha/2$ quantiles of the approximate posterior distribution. The functions `clm_crint_delta` and `clm_crint_montecarlo` compute credible intervals for supplementary estimands using the two methods described above, respectively. The adjusted outcome mean and median calculations are implemented by functions `mean_xp` and `quantile_xp`, respectively. The four supplementary estimands are implemented by functions defined in the R code file "supplemental_estimands.R"

The four supplementary estimands include the treatment difference in mean and median of the primary outcome, and the treatment difference in probabilities associated with outcome levels -1 and 0. Each of these estimands will be adjusted to the most common (modal) value for each covariate. The mean and median estimates are defined as the mean and median of the distribution defined by the cumulative probabilities associated with each outcome level, adjusted for covariates.

12 APPENDIX: KEY SECONDARY OUTCOME TESTING PROCEDURE

Each trial in the ACTIV 4 Host Tissue platform will separately use a fixed sequence method to control the familywise type-I error probability, i.e., the probability of erroneously concluding efficacy of the trial intervention with respect to any one or more of the primary and key secondary outcomes. Specifically, a conclusion of efficacy regarding the primary outcome will be required prior to testing the first designated key secondary outcome. Each subsequent key secondary outcome, in the designated order, will take place only if the preceding key secondary outcome demonstrates efficacy. This approach provides strong control of the familywise type-I error probability for the family of primary and key secondary outcomes. For weak familywise type-I error control (i.e., under the assumption that the intervention effect is null for all tests in the family), the fixed sequence method requires only that the test of the primary outcome (i.e., the outcome tested first) have the specified type-I error rate. For strong type-I error control, the fixed sequence procedure requires that each individual test in the sequence have the desired type-I error probability, 2.5% for trials under the ACTIV 4 Host Tissue platform. Because the test of efficacy associated with the primary outcome has adaptive elements, including interim analyses, a statistical simulation (as described in the "Statistical Analysis Plan") was implemented to identify the test characteristics that ensure a 2.5% type-I error probability for that test. Each key secondary outcome is tested for efficacy only at the final analysis. Thus, type-I error control for the key secondary outcomes relies on established theoretical arguments and methods.

All key secondary outcomes use Bayesian logistic regression or proportional odds logistic regression. If key secondary outcome testing is required under the fixed sequence procedure, efficacy will be concluded if the posterior probability for efficacy ($P(OR > 1|Data)$ for Alive and respiratory failure-free at day 28, and $P(OR < 1 \mid Data)$ for WHO 8-point ordinal scale at day 28 and Mortality at day 28) exceeds 0.975.

Because a flat prior is used, and the posterior is computed using a Laplace approximation, the maximum *a posteriori* estimate of the log odds ratio is identical to the maximum likelihood estimate (MLE), and the Laplace approximated posterior distribution is identical to the approximate sampling distribution of the MLE: a normal distribution with mean equal to the estimate and variance-covariance equal to the inverse observed Fisher information (see

Appendix: Laplace Approximation). In conventional frequentist testing, efficacy is indicated when the estimate exceeds a critical value selected such that the frequency of this occurring under the null hypothesis is 0.025. Because of the equivalence between the approximate posterior and MLE sampling distributions, setting the posterior probability for efficacy threshold to 0.975 ensures that any estimate meeting this threshold must also exceed the critical value that ensures less than 2.5% type-I error frequency. The figure below illustrates this concept:

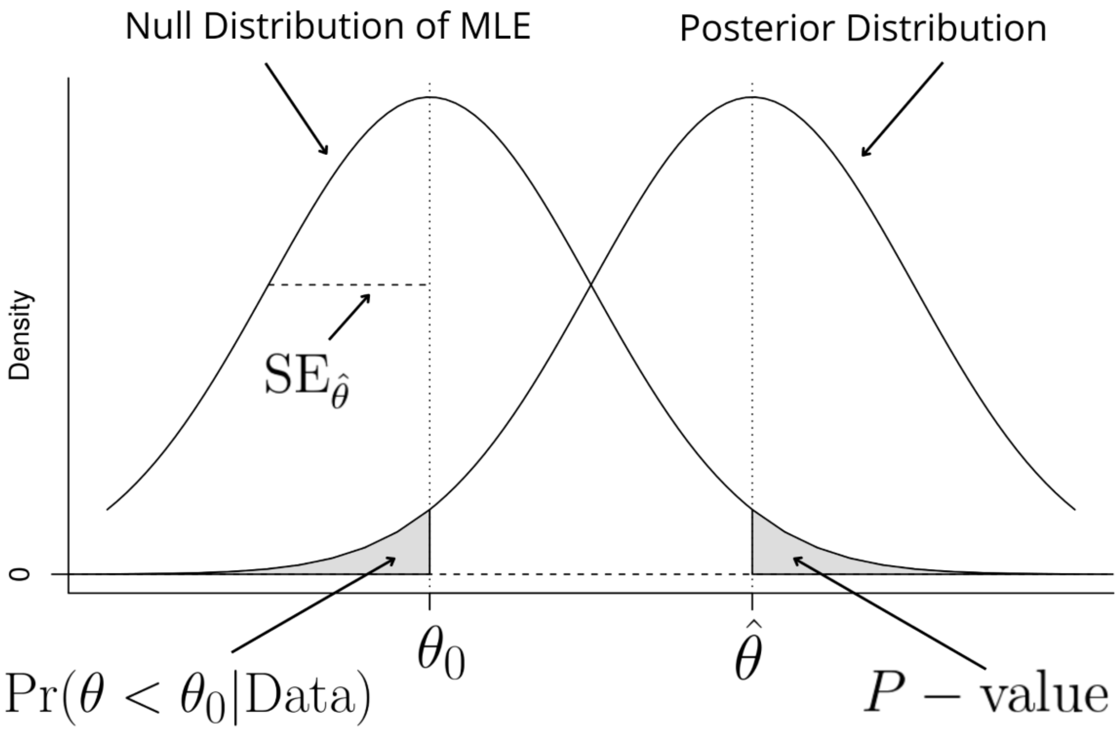

13 APPENDIX: LAPLACE APPROXIMATION

Let random variables $Y_1 \dots Y_N$ represent an independent and identically distributed sample from a probability distribution with density function $f(Y|\theta)$, and define $y_1 \dots y_N$ as realizations of this sample. If $f(Y|\theta)$ is derived from a regression model, then the density function may also condition on covariates (elsewhere denoted X and x). However, covariate information is not pertinent to the derivations below, and are omitted for clarity. The likelihood function is defined as follows:

$$L(\theta|y_1 \dots y_N) = \prod_{i=1}^{N} f(y_i|\theta) \ \#(1)$$

The natural log of the likelihood function is defined as follows:

$$\ell(\theta|y_1 \dots y_N) = \sum_{i=1}^{N} \log f(y_i|\theta) \ \#(2)$$

In Bayesian analysis, the posterior density function is proportional to the likelihood function multiplied by the prior density function as follows:

$$P(\theta|y_1 \dots y_N) \propto L(\theta|y_1 \dots y_N)P(\theta)\#(3)$$

13.1 Equivalence of MAP and MLE with Flat Prior

A "flat prior" density function is defined to be proportional to 1 for all values of θ. Thus, when a flat prior is specified, the posterior density function is proportional to the likelihood function. In addition, the maximum *a posteriori* (MAP) estimator of θ is also a maximum likelihood estimator (MLE):

$$\hat{\theta} = \arg\max_{\theta} P(\theta|y_1 \dots y_N) = \arg\max_{\theta} L(\theta|y_1 \dots y_N) = \arg\max_{\theta} \ell(\theta|y_1 \dots y_N)\ \#(4)$$

13.2 Asymptotic Normality of MLE

Under regularity conditions, the MLE converges in distribution to a normal distribution:

$$\hat{\theta} \xrightarrow{d} N(\theta_0, I^{-1})\#(5)$$

where θ_0 is the true but unknown value of θ, and I is the Fisher information:

$$I = E_{\theta_0}\left[-\frac{\partial^2}{\partial\theta^2}\ell(\theta_0|Y_1 \dots Y_N)\right]\ \#(6)$$

In practice, because θ_0 is unknown, inferences about θ_0 are made by substituting $\hat{\theta}$ in place of θ_0 and the observed information is substituted in place of the Fisher information:

$$\hat{\theta} \sim N\left(\hat{\theta}, \hat{I}^{-1}\right)\ \#(7)$$

The observed information is the negative Hessian of the log likelihood function evaluated at $\hat{\theta}$:

$$\hat{I} = \left[-\frac{\partial^2}{\partial\theta^2}\ell(\theta|y_1 \dots y_N)\right]_{\theta=\hat{\theta}}\ \#(8)$$

13.3 Laplace Approximation to Posterior

The Laplace approximation to a posterior density function (or any density function) is based on a two-term Taylor expansion of the natural log of the density function about $\hat{\theta}$:

$$q(\theta) \approx q\left(\hat{\theta}\right) + \left(\theta - \hat{\theta}\right)q'\left(\hat{\theta}\right) + \frac{1}{2}\left(\theta - \hat{\theta}\right)^T q''\left(\hat{\theta}\right)\left(\theta - \hat{\theta}\right)\#(9)$$

where $q(\theta)$ is the log posterior density function and $q'\left(\hat{\theta}\right)$ and $q''\left(\hat{\theta}\right)$ are the gradient and Hessian of $q(\theta)$, respectively, evaluated at $\hat{\theta}$. When a flat prior is used, $q(\theta)$ is equal to the log likelihood function plus a constant c:

$$q(\theta) = \log P(\theta|y_1 \dots y_N) = \ell(\theta|y_1 \dots y_N) + c\#(10)$$

Because $\hat{\theta}$ is defined to be the MAP estimate, $q'(\hat{\theta}) = 0$. Thus, expression (9) simplifies:

$$q(\theta) \approx -\frac{1}{2}(\theta - \hat{\theta})^T[-q''(\hat{\theta})](\theta - \hat{\theta}) + c\#(11)$$

where the negative Hessian is identical to the observed information when a flat prior is used:

$$-q''(\hat{\theta}) = \left[-\frac{\partial^2}{\partial\theta^2}\log P(\theta|y_1 \ldots y_N)\right]_{\theta=\hat{\theta}} = \left[-\frac{\partial^2}{\partial\theta^2}\ell(\theta|y_1 \ldots y_N)\right]_{\theta=\hat{\theta}} = \hat{I}\#(12)$$

Exponentiating expression (11) demonstrates that the Laplace approximation to the posterior density must be a normal density with mean $\hat{\theta}$ and variance-covariance $\hat{I}^{-1}$. This is identical to the asymptotic sampling distribution of the MLE given in expression (7):

$$(\theta|y_1 \ldots y_N) \sim N(\hat{\theta}, \hat{I}^{-1})\ \#(13)$$

Under regularity conditions, the Bernstein-von Mises theorem provides asymptotic guarantees regarding the quality of the Laplace approximation.
