# Supplementary material for: Fostamatinib for Hospitalized Adults With COVID-19 and Hypoxemia: A Randomized Clinical Trial
Source: JAMA Netw Open. 2024 Dec 3;7(12):e2448215. doi: 10.1001/jamanetworkopen.2024.48215 (PMC11615712; doi:10.1001/jamanetworkopen.2024.48215)
Supplement: Supplement 2. — eTable 1. Enrolling Sites eTable 2. Eligibility Criteria eTable 3. Trial Outcomes eTable 4. Protocol-Specified Exempt Serious Events eTable 5. Reasons for Not Receiving Full 28 Doses of Fostamatinib eTable 6. Adverse Events in Fostamatinib Trial eTable 7. Frequency of Other Safety Outcomes and PSESEs eFigure 1. Timeline of Trials Enrolling as Part of the ACTIV-4 Host Tissue Platform eFigure 2. Assessment of the Proportional Odds Assumption Using Repeated Dichotomizations eFigure 3. Distribution of Oxygen-Free Days in the Fostamatinib Trial eFigure 4. Treatment Effect on Primary, Secondary, and Exploratory Outcomes [file jamanetwopen-e2448215-s002.pdf]

Supplementary Online Content

Collins SP, Shotwell MS, Strich JR, et al; ACTIV-4 Host Tissue Investigators. Fostamatinib for hospitalized adults with COVID-19 and hypoxemia: a randomized clinical trial. *JAMA Netw Open*. 2024;7(12):e2448215. doi:10.1001/jamanetworkopen.2024.48215

eAppendix 1. ACTIV-4 Host Tissue Investigators and Collaborators
eTable 1. Enrolling Sites
eTable 2. Eligibility Criteria
eTable 3. Trial Outcomes
eTable 4. Protocol-Specified Exempt Serious Events
eTable 5. Reasons for Not Receiving Full 28 Doses of Fostamatinib
eTable 6. Adverse Events in Fostamatinib Trial
eTable 7. Frequency of Other Safety Outcomes and PSESEs
eFigure 1. Timeline of Trials Enrolling as Part of the ACTIV-4 Host Tissue Platform
eFigure 2. Assessment of the Proportional Odds Assumption Using Repeated Dichotomizations
eFigure 3. Distribution of Oxygen-Free Days in the Fostamatinib Trial
eFigure 4. Treatment Effect on Primary, Secondary, and Exploratory Outcomes
eAppendix 2. Study Drug Dose Modification Considerations and Stopping Rules

This supplementary material has been provided by the authors to give readers additional information about their work.

© 2024 Collins SP et al. *JAMA Network Open*.

eAppendix 1. ACTIV-4 Host Tissue Investigators and Collaborators

Investigators and collaborators for the fostamatinib trial in the ACTIV-4 Host Tissue platform (by institution) not included as masthead authors.

Data Safety and Monitoring Board (DSMB)
Richard C. Becker, MD; Gregory del Zoppo, MD; Peter Henke, MD; Richard Holubkov, PhD; Maryl Johnson, MD; Kim Kerr, MD; Hannah I. Lipman, MD; Fedor Lurie, MD, PhD; Bertram Pitt, MD; Sara K. Vesely, PhD; Jerome L. Fleg, MD.

Coordinating Center (Vanderbilt University Medical Center)
Dave Aamodt, Debra Clark; Jessica Collins; Maya Cook; Sheri Dixon; Carly Gatewood; John Graves; Debbie Hunter; Courtney Jordan; Sahar Ko; Ugo (Ben) Lekwauwa; Itzel Lopez; David McKeel; Dirk Orozco; Nelson Prato; Ally Qi; Kalley Stagner; Krista Vermillion; Stephanie Winchell; Taylor Young

Rigel Pharmaceuticals and Affiliates
Esteban Masuda, Asif Siddiqui

Research Organization (KC) LTD (ROKC)
Caroline Brooks, Carl Fletcher, Annie Duffy, Paul Walsh, Jack Moody, Toni Sobande, Amina Ali, Yomi Henry, Frances Ogueri, Alba Sierto, Maria Sanchez Grande, Chiara Spinello, Olufunke Anthony, Michal Sieracki, Michaella Tomlin

NEAT ID
Prof Anton Pozniak, Tim Leaver, Polly Parks

ENROLLING SITES

1. **Vanderbilt University Medical Center**
   Jakea Johnson; Ryan Walsh, MD; Brian Bales, MD; Karen Miller, RN; Donna Torr, PharmD

2. **Wake Forest University**
   D. Clark Files MD; Kevin W. Gibbs MD; Darija Ward MBA; Leigha Landreth, RN; Lisa Parks RN; Lori Flores NP

3. **University of New Mexico Health Sciences Center**
   J. Pedro Teixeira; Sandra Cardenas; Juan A Ceniceros; Amy G Cunningham; Susan Kunkel; Debbie M Lovato

4. **University of Nebraska Medical Center**
   Brooklin Zimmerman MSN; Thanh Nguyen PhD; Wesley Zeger MD; Noah Wiedel MD

5. **Washington University**
   Stephanie Stilinovic; Caroline Foster; Jeanne Flannigan

6. **Yale University**
   Carolyn Brokowski; Jing Lu; Muriel Solberg; Dana Lee

7. **Dignity Health Research Institute**
   Brian Tiffany; Charlotte Tanner; Annette Taylor; Jennine Zumbahl

8. **Virginia Commonwealth University**
   Aamer Syed, MD; Jessica Mason, MPH

© 2024 Collins SP et al. *JAMA Network Open*.

9. **University of Virginia**
Patrick E. H. Jackson; Rachael W. Coleman; Heather M. Haughey

10. **University of Florida, Gainesville**
Kartik Cherabuddi; Rebecca Wakeman Murray

11. **Newton-Wellesley Hospital**
Kathryn Fairbank-Haynes CRC

12. **Stanford University**
Angela J. Rogers; Jennifer G. Wilson; Rosemary Vojnik; Cynthia Perez

13. **Denver Health Medical Center**
David Wyles; Terra D. Hiller; Judy L. Oakes; Ana Z. Garcia

14. **University of Colorado**
Jennifer Fickes-Siler, David Douin; Amiran Baduashvili; Henry Kramer; Lakshmi Chauhan; Amanda Martinez; Jennifer Peers; Kristine Schauer; Flora Martinez; Lani Finck; Jill Bastman; Ashley Licursi

15. **Beth Israel Deaconess Medical Center**
Sharon Hayes; Nicholas Kurtzman; Elinita Rosseto; Douglas Scaffidi; Nathan Shapiro

16. **Oregon Health and Science University**
Jonathan Pak; Gopal Allada; Genesis Briceno; Jose Peña; Minn Oh

17. **Moses Hospital of Montefiore Medical Center**
Michelle Gong; Amira Mohamed MD; Luke Andrea MD; Rahul Nair MD; William Nkemdirim MD; Sabah Boujid; Martha Torres; Ofelia Garcia

18. **Johns Hopkins University**
Harith Ali, Sasha Beselman, Yolanda J Eby, Arber Shehu, Vitaliy Klimov

19. **University of Cincinnati**
R. Duncan Hite, Hammad Tanzeem, Chris Droege, Jessica Winter

20. **Cleveland Clinic Foundation**
Simon Mucha, MD; Nirosshan Thiruchelvam, MD; Matthew Siuba, MD; Omar Mehkri, MD

21. **Cedars-Sinai Medical Center**
Susan Jackman; Antonina Caudill; Emad Bayoumi; Ethan Pascual; Po-En Chen, Sam S Torbati, MD, Tanyalak Parimon, MD, Bradley Rosen, MD, and Yuri Matusov, MD

22. **Intermountain Healthcare**
Michael J. Lanspa; Ithan D. Peltan; Samuel M. Brown; Jason R. Carr; Daniel B. Knox; Lindsay M. Leither

23. **Jack D. Weiler Hospital of Montefiore Medical Center**
Brenda Lopez, Benjamin Galen MD

24. **Temple University**
Nina Gentile, MD; Derek Isenberg, MD; Hannah Reimer; Paul Cincola

25. **University of Utah Health Sciences Center**
Estelle S. Harris; Sean J Callahan; Misty B Yamane; Macy AG Barrios

26. **Alexian Brothers Medical Center**
Neeraj Desai; Amit Bharara; Michael Keller; Prat Majumder; Carri Dohe

© 2024 Collins SP et al. *JAMA Network Open*.

27. **Massachusetts General Hospital**
Kathryn Hibbert, MD; Justin Margolin, BS; Blair Parry, CCRC, BA

28. **Baystate Health**
Mark A. Tidswell, Jay S. Steingrub, Lesley De Souza, Cynthia Kardos, Denise Gallant, Rae Lynn DeFoe, Sarah Romain, Scott Ouellette, Serena Estrada, Ryan Kindle, Bogdan Tiru, Howard Smithline, Carolyn Garcia

29. **Medical University of South Carolina**
Charles Terry, MD, MSCR; Melissa Blender; Abbey Grady

30. **University of North Carolina**
Andrew Powell, BS

31. **Brigham and Women's Hospital**
Peter C. Hou, Mohammad A. Hasdianda, Steven E. Miyawaki, Nikita Umale, Brandon Lee

32. **Grady Memorial Hospital/Ponce de Leon CRS**
Valeria D.Cantos, Colleen F. Kelley, Paulina A. Rebolledo, Sheetal Kandiah, Renee Cook, Betsy Hall, Christin Root, Juliet Brown, Christopher Foster, Philip Powers, Pamela Lankford-Turner

33. **University of Texas, Houston**
Bela Patel, MD; Rodeo Abrencillo, MD; Robier Aguillon-Prada, MD; Ryan Huebinger, MD; Gabriel Patarroyo Aponte, MD; Syed Ghazi Rizvi, MD; Elizabeth Vidales; Idorenyin Udoh-Bradford

34. **Hospital General Universitario de Elche**
Mar Masiá, Sergio Padilla, Guillermo Telenti, Paula Mascarell, Javier García-Abellán, Ángela Botella, Catalina Robledano

35. **Hospital Clinico San Carlos**
Vicente Estrada, Reynaldo Homen, Javier Rodriguez-Añover

36. **Hospital Universitario Vall dHebron (VHIR)**
Joaquin Burgos, MD

37. **Clinical HIV Research Unit - Helen Joseph Hospital (WITS CHRU)**
Tom Boyles, MD; Zanele Mkhabela

38. **Hospital Clinico Universitario Lozano Blesa**
Galadriel Pellejero; Elena Morte-Romea; Santiago Letona-Carbajo; María José Esquillor; Carla Toyas; Silvia Loscos

39. **Worthwhile Clinical Trials (WWCT Lakeview Hospital)**
Nazreen Hussen, MD

40. **Hospital Universitario Fundacion Alcorcon**
Dr Carlos Guijarro, Dr Maria Velasco-Arribas

41. **Hospital Federal dos Servidores do Estado**
Esaú Custódio João Filho, Elaine C. de Oliveira Souza

42. **University Hospital Bonn**
Dr Christoph Boesecke; Prof. Jurgen Rockstroh.

© 2024 Collins SP et al. *JAMA Network Open*.

43. **San Raffaele Turro Hospital**
Dr Marco Ripa, Dr Silvia Nozza

© 2024 Collins SP et al. *JAMA Network Open*.

eTable 1. Enrolling sites

Enrolling Site Number	Hospital	Number of In-patient Hospital Beds	Type of Hospital	Fostamatinib eligible randomizations
1	Vanderbilt University Medical Center	1046	Teaching Hospital Affiliated with a University	19
2	Wake Forest University	886	Teaching Hospital Affiliated with a University	28
3	University of New Mexico Health Sciences Center	618	Teaching Hospital Affiliated with a University	19
4	University of Nebraska Medical Center	616	Teaching Hospital Affiliated with a University	7
5	Washington University	375	Teaching Hospital Affiliated with a University	24
6	Yale University	1541	Teaching Hospital Affiliated with a University	34
7	Dignity Health Research Institute	429	Community Hospital System	12
8	Virginia Commonwealth University	805	Teaching Hospital Affiliated with a University	12
9	University of Virginia	671	Teaching Hospital Affiliated with a University	5
10	University of Florida, Gainesville	1037	Teaching Hospital Affiliated with a University	10
11	Newton-Wellesley Hospital	273	Community Hospital Affiliated with a University	2
12	Stanford University	584	Teaching Hospital Affiliated with a University	11

© 2024 Collins SP et al. *JAMA Network Open*.

Enrolling Site Number	Hospital	Number of In-patient Hospital Beds	Type of Hospital	Fostamatinib eligible randomizations
13	Denver Health Medical Center	472	Public Hospital, academically affiliated	11
14	University of Colorado Denver	703	Teaching Hospital Affiliated with a University	48
15	Beth Israel Deaconess Medical Center	673	Teaching Hospital Affiliated with a University	2
16	Oregon Health and Science University	549	Teaching Hospital Affiliated with a University	33
17	Moses Hospital of Montefiore Medical Center	1530	Teaching Hospital Affiliated with a University	5
18	Johns Hopkins University	1162	Teaching Hospital Affiliated with a University	2
19	University of Cincinnati	726	Teaching Hospital Affiliated with a University	17
20	Cleveland Clinic Foundation	1300	Teaching Hospital Affiliated with a University	6
21	Cedars-Sinai Medical Center	886	Community Hospital, academically affiliated	5
22	Intermountain Healthcare	430	Community Hospital, academically affiliated	17
23	Jack D. Weiler Hospital of Montefiore Medical Center	369	Teaching Hospital Affiliated with a University	7
24	Temple University	879	Teaching Hospital Affiliated with a University	8
25	University of Utah Health Sciences Center	806	Teaching Hospital	10

© 2024 Collins SP et al. *JAMA Network Open*.

Enrolling Site Number	Hospital	Number of In-patient Hospital Beds	Type of Hospital	Fostamatinib eligible randomizations
			Affiliated with a University	
26	St. Alexius Medical Center	318	Community Hospital	2
27	Massachusetts General Hospital	999	Teaching Hospital Affiliated with a University	2
28	Baystate Health	734	Teaching Hospital Affiliated with a University	3
29	Medical University of South Carolina	709	Teaching Hospital Affiliated with a University	4
30	University of North Carolina	932	Teaching Hospital Affiliated with a University	5
31	Brigham and Women's Hospital	888	Teaching Hospital Affiliated with a University	1
32	Grady Memorial Hospital/Emory Ponce de Leon CRS	953	Community Hospital Affiliated with a University	1
33	University of Texas, Houston	723	Teaching Hospital Affiliated with a University	1
34	Hospital General Universitario de Elche	408	Teaching Hospital Affiliated with a University	8
35	Hospital Clinico San Carlos	861	Teaching Hospital Affiliated with a University	4
36	Hospital Universitario Vall dHebron (VHIR)	1400	Teaching Hospital Affiliated with a University	4
37	Clinical HIV Research Unit - Helen Joseph Hospital (WITS CHRU)	700	Teaching Hospital Affiliated with a University	3
38	Hospital Clinico Universitario Lozano Blesa	800	Teaching Hospital	2

© 2024 Collins SP et al. *JAMA Network Open*.

Enrolling Site Number	Hospital	Number of In-patient Hospital Beds	Type of Hospital	Fostamatinib eligible randomizations
			Affiliated with a University	
39	Worthwhile Clinical Trials (WWCT Lakeview Hospital)	94	Private Hospital	2
40	Hospital Universitario Fundacion Alcorcon	400	Teaching Hospital Affiliated with a University	1
41	Hospital Federal dos Servidores do Estado	398	Teaching Hospital Affiliated with a University	1
42	University Hospital Bonn	1306	Teaching Hospital Affiliated with a University	1
43	San Raffaele Turro Hospital	1350	Teaching Hospital Affiliated with a University	1

© 2024 Collins SP et al. *JAMA Network Open*.

eTable 2. Eligibility Criteria

To enter a trial, patients must have met all eligibility criteria for the ACTIV-4 Host Tissue Platform and the specific criteria for the individual trial.

	Inclusion Criteria	Exclusion Criteria
**ACTIV-4 Host Tissue Platform**	1. Hospitalized for COVID-19<br>2. ≥18 years of age<br>3. SARS-CoV-2 infection, documented by:<br>a) a nucleic acid test (NAT) or equivalent testing within 3 days prior to randomization OR<br>b) documented by NAT or equivalent testing more than 3 days prior to randomization AND progressive disease suggestive of ongoing SARS-CoV-2 infection per the responsible investigator (For non-NAT tests, only those deemed with equivalent specificity to NAT by the protocol team will be allowed. A central list of allowed non-NAT tests is maintained in Appendix F.)<br>4. Hypoxemia, defined as SpO2 <92% on room air, new receipt of supplemental oxygen to maintain SpO2 ≥92%, or increased supplemental oxygen to maintain SpO2 ≥92% for a patient on chronic oxygen therapy<br>5. Symptoms or signs of acute COVID-19, defined as one or more of the following:<br>a) cough<br>b) reported or documented body temperature of 100.4º F or greater<br>c) shortness of breath<br>d) chest pain<br>e) infiltrates on chest imaging (x-ray, CT scan, lung ultrasound)	1. COVID-19 symptom onset >14 days prior to randomization<br>2. Hospitalized for >72 hours prior to randomization<br>3. Pregnancy<br>4. Breastfeeding<br>5. Prisoners<br>6. End-stage renal disease (ESRD) on dialysis<br>7. Patient and/or clinical team is not pursuing full medical management (if a patient has a Do Not Resuscitate order that precludes chest compressions in the event of a cardiac arrest but is otherwise pursuing full medical management, he/she is eligible for this trial).<br>8. The treating clinician expects inability to participate in study procedures or participation would not be in the best interests of the patient

**Fostamatinib trial specific criteria**		**1.** Randomized in another trial evaluating fostamatinib in the prior 30 days<br>**2.** Study arm exclusion criteria measured within 24 hours prior to randomization:<br>• AST or ALT ≥ 5 × upper limit of normal (ULN) or ALT or AST ≥ 3 × ULN and total bilirubin ≥ 2 × ULN<br>• SBP > 160 mmHg or DBP > 100 mmHg at the time of screening and randomization<br>• ANC < 1000/mL<br>• Patient is anticipated to require a strong CYP3A inhibitor (Atazanavir, Certinib, Clarithromycin, Cobicistat and cobicistat-containing coformulations, Idelalisib, Indinavir, Itraconazole, Ketoconazole, Levoketoconazole, Lonafarnib, Lopinavir, Mifeprostone, Mibefradil, Nefazodone, Nelfinavir, Ombitasvir-paritaprevir-ritonavir plus dasabuvir, Posaconazole, Ribociclib Ritonavir, Saquinavir, Telithromycin, Troleandomycin, Tucatinib, Voriconazole) from randomization to 21 days post-randomization. For a full list of CYP3A4 substrates, please reference this regularly updated list: https://drug-interactions.medicine.iu.edu/MainTable.aspx.<br>**3.** Patient unable to participate or declines participation in the fostamatinib arm.

© 2024 Collins SP et al. *JAMA Network Open*.

eTable 3. Trial Outcomes

Pre-specified clinical outcomes are shown in this table. Exploratory biomarker-based outcomes will be reported separately in a different manuscript.

Outcome	Definition	Variable type	Analysis approach	Interpretation of OR <1.0
Primary efficacy outcome				
Oxygen-free days to day 28	28 minus the number of days between initiation and final liberation from new supplemental oxygen use during the 28 days following randomization. Patients who died before day 28 were coded as -1.	Ordinal scale with 30 levels	Multivariable proportional odds regression model adjusted for age group (18-30, 31-65, or >65 years), sex at birth, and WHO COVID ordinal scale at baseline (level 4, 5, or 6-7)	Inferiority of active agent
Key secondary efficacy outcomes				
All-cause all-location mortality at day 28	Alive vs dead at study day 28	Binary	Multivariable logistic regression model adjusted for age group (18-30, 31-65, or >65 years), sex at birth, and WHO COVID ordinal scale at baseline (level 4, 5, or 6-7)	Superiority of active agent
Alive and respiratory failure free at day 28	Composite of alive and off respiratory support (no high flow nasal oxygen, non-invasive ventilation, or invasive mechanical ventilation) vs either dead or on respiratory support at day 28	Binary	Multivariable logistic regression model adjusted for age group (18-30, 31-65, or >65 years), sex at birth, and WHO COVID ordinal scale at baseline (level 4, 5, or 6-7)	Inferiority of active agent
WHO COVID-19 clinical progression ordinal scale at day 28	Patient's clinical status at day 28 classified based on the following 8 mutually exclusive categories:<br>1. Ambulatory – Not hospitalized, no limitation of activities<br>2. Ambulatory – Not hospitalized with limitation of activities or home oxygen therapy<br>3. Hospitalized Mild Disease – Hospitalized, no oxygen therapy<br>4. Hospitalized Mild Disease – Oxygen by mask or nasal prongs<br>5. Hospitalized Severe Disease – Non-invasive	Ordinal scale with 8 levels	Multivariable proportional odds regression model adjusted for age group (18-30, 31-65, or >65 years), sex at birth, and WHO COVID ordinal scale at baseline (level 4, 5, or 6-7)	Superiority of active agent

© 2024 Collins SP et al. *JAMA Network Open*.

Outcome	Definition	Variable type	Analysis approach	Interpretation of OR <1.0
	ventilation or high-flow oxygen<br>6. Hospitalized Severe Disease – Invasive mechanical ventilation<br>7. Hospitalized Severe Disease – Invasive mechanical ventilation plus additional organ support with vasopressors, renal replacement therapy or extracorporeal membrane oxygenation (ECMO)<br>8. Dead			
Other secondary efficacy outcomes				
Alive and oxygen free at 14 days	Composite of alive and off new supplemental oxygen therapy vs either dead or on new supplemental oxygen therapy at day 14	Binary	Multivariable logistic regression model adjusted for age group (18-30, 31-65, or >65 years), sex at birth, and WHO COVID ordinal scale at baseline (level 4, 5, or 6-7)	Inferiority of active agent
Alive and oxygen free at 28 days	Composite of alive and off new supplemental oxygen therapy vs either dead or on new supplemental oxygen therapy at day 28	Binary	Multivariable logistic regression model adjusted for age group (18-30, 31-65, or >65 years), sex at birth, and WHO COVID ordinal scale at baseline (level 4, 5, or 6-7)	Inferiority of active agent
Alive and respiratory failure free at 14 days	Composite of alive and off respiratory support (no high flow nasal oxygen, non-invasive ventilation, or invasive mechanical ventilation) vs either dead or on respiratory support at day 14	Binary	Multivariable logistic regression model adjusted for age group (18-30, 31-65, or >65 years), sex at birth, and WHO COVID ordinal scale at baseline (level 4, 5, or 6-7)	Inferiority of active agent
Alive and free of new invasive mechanical ventilation at day 14	Composite of alive and off new invasive mechanical ventilation vs either dead or on invasive mechanical ventilation at day 14	Binary	Multivariable logistic regression model adjusted for age group (18-30, 31-65, or >65 years), sex at birth, and WHO COVID ordinal scale at baseline (level 4, 5, or 6-7)	Inferiority of active agent

© 2024 Collins SP et al. *JAMA Network Open*.

Outcome	Definition	Variable type	Analysis approach	Interpretation of OR <1.0
Alive and free of new invasive mechanical ventilation at day 28	Composite of alive and off new invasive mechanical ventilation vs either dead or on invasive mechanical ventilation at day 28	Binary	Multivariable logistic regression model adjusted for age group (18-30, 31-65, or >65 years), sex at birth, and WHO COVID ordinal scale at baseline (level 4, 5, or 6-7)	Inferiority of active agent
In-hospital mortality	Died prior to hospital discharge vs survived to hospital discharge based on the index hospitalization in which the patient was enrolled in the trial	Binary	Multivariable logistic regression model adjusted for age group (18-30, 31-65, or >65 years), sex at birth, and WHO COVID ordinal scale at baseline (level 4, 5, or 6-7)	Superiority of active agent
60-day mortality	Dead vs alive at day 60	Binary	Multivariable logistic regression model adjusted for age group (18-30, 31-65, or >65 years), sex at birth, and WHO COVID ordinal scale at baseline (level 4, 5, or 6-7)	Superiority of active agent
90-day mortality	Dead vs alive at day 90	Binary	Multivariable logistic regression model adjusted for age group (18-30, 31-65, or >65 years), sex at birth, and WHO COVID ordinal scale at baseline (level 4, 5, or 6-7)	Superiority of active agent
WHO COVID-19 clinical progression ordinal scale at day 14	Patient's clinical status at day 14 classified based on the 8 mutually exclusive categories defined above.	Ordinal scale with 8 levels	Multivariable proportional odds regression model adjusted for age group (18-30, 31-65, or >65 years), sex at birth, and WHO COVID ordinal scale at baseline (level 4, 5, or 6-7)	Superiority of active agent
WHO COVID-19 clinical progression ordinal scale at day 60	Patient's clinical status at day 60 classified based on the 8 mutually exclusive categories defined above.	Ordinal scale with 8 levels	Multivariable proportional odds regression model adjusted for age group (18-30, 31-65, or >65 years), sex at	Superiority of active agent

© 2024 Collins SP et al. *JAMA Network Open*.

Outcome	Definition	Variable type	Analysis approach	Interpretation of OR <1.0
			birth, and WHO COVID ordinal scale at baseline (level 4, 5, or 6-7)	
Hospital-free days through day 28	28 minus the number of days between randomization and hospital discharge for the index hospital admission in which the patient was enrolled in the trial. Patients who died before day 28 were coded as -1. Patients who remained hospitalized after day 28 were coded as 0.	Ordinal scale with 30 levels	Multivariable proportional odds regression model adjusted for age group (18-30, 31-65, or >65 years), sex at birth, and WHO COVID ordinal scale at baseline (level 4, 5, or 6-7)	Inferiority of active agent
Respiratory-failure-free days through day 28	28 minus the number of days between initiation and final liberation from respiratory support (high flow nasal oxygen, non-invasive ventilation, or invasive mechanical ventilation) during the 28 days following randomization. Patients who died before day 28 were coded as -1.	Ordinal scale with 30 levels	Multivariable proportional odds regression model adjusted for age group (18-30, 31-65, or >65 years), sex at birth, and WHO COVID ordinal scale at baseline (level 4, 5, or 6-7)	Inferiority of active agent
Ventilator-free days through day 28	28 minus the number of days between initiation and final liberation from invasive mechanical ventilation during the 28 days following randomization. Patients who died before day 28 were coded as -1.	Ordinal scale with 30 levels	Multivariable proportional odds regression model adjusted for age group (18-30, 31-65, or >65 years), sex at birth, and WHO COVID ordinal scale at baseline (level 4, 5, or 6-7)	Inferiority of active agent
Safety outcomes				
Allergic reaction through day 7	Angioedema or another acute reaction proximate to study drug administration that the site investigator judged to be possibly related to a study drug allergy.<br>Patients were coded as no allergic reaction vs ≥1 allergic reaction.	Binary	Multivariable logistic regression model adjusted for age group (18-30, 31-65, or >65 years), sex at birth, and WHO COVID ordinal scale at baseline (level 4, 5, or 6-7)	Fewer safety events with active agent

© 2024 Collins SP et al. *JAMA Network Open*.

Outcome	Definition	Variable type	Analysis approach	Interpretation of OR <1.0
Allergic reaction through day 28	Same definition as above for allergic reaction through day 7	Binary	Multivariable logistic regression model adjusted for age group (18-30, 31-65, or >65 years), sex at birth, and WHO COVID ordinal scale at baseline (level 4, 5, or 6-7)	Fewer safety events with active agent
New renal replacement therapy through day 7	Initiation of renal replacement therapy prior to day 28 in a patient not on renal replacement therapy at randomization.	Binary	Multivariable logistic regression model adjusted for age group (18-30, 31-65, or >65 years), sex at birth, and WHO COVID ordinal scale at baseline (level 4, 5, or 6-7)	Fewer safety events with active agent
New renal replacement therapy through day 28	Same definition as above for new renal replacement therapy through day 7	Binary	Multivariable logistic regression model adjusted for age group (18-30, 31-65, or >65 years), sex at birth, and WHO COVID ordinal scale at baseline (level 4, 5, or 6-7)	Fewer safety events with active agent
Hypotension through day 7	A low blood pressure leading to initiation or increase in vasopressor therapy, administration of a fluid bolus of ≥500 ml, or discontinuation of the study drug. Patients were coded as no hypotensive events vs ≥1 hypotensive events.	Binary	Multivariable logistic regression model adjusted for age group (18-30, 31-65, or >65 years), sex at birth, and WHO COVID ordinal scale at baseline (level 4, 5, or 6-7)	Fewer safety events with active agent
Hypotension through day 28	Same definition as above for hypotension through day 7	Binary	Multivariable logistic regression model adjusted for age group (18-30, 31-65, or >65 years), sex at birth, and WHO COVID ordinal scale at baseline (level 4, 5, or 6-7)	Fewer safety events with active agent
Hypertension through Day 28	Elevated arterial blood pressure leading to either [1] initiation or increase in antihypertensive medications or [2] discontinuation of the study drug	Binary	Multivariable logistic regression model adjusted for age group (18-30, 31-65, or >65 years), sex at birth, and WHO COVID ordinal scale at baseline (level 4, 5, or 6-7)	Fewer safety events with active agent

© 2024 Collins SP et al. *JAMA Network Open*.

Outcome	Definition	Variable type	Analysis approach	Interpretation of OR <1.0
Neutropenia through Day 28	ANC < 500 cell/mcl	Binary	Multivariable logistic regression model adjusted for age group (18-30, 31-65, or >65 years), sex at birth, and WHO COVID ordinal scale at baseline (level 4, 5, or 6-7)	Fewer safety events with active agent
Hepatotoxicity through Day 28	ALT > 136 U/L for men, ALT > 96 U/L for women	Binary	Multivariable logistic regression model adjusted for age group (18-30, 31-65, or >65 years), sex at birth, and WHO COVID ordinal scale at baseline (level 4, 5, or 6-7)	Fewer safety events with active agent
Hepatotoxicity through Day 28	AST > 128 U/L for men, AST > 104 U/L for women	Binary	Multivariable logistic regression model adjusted for age group (18-30, 31-65, or >65 years), sex at birth, and WHO COVID ordinal scale at baseline (level 4, 5, or 6-7)	Fewer safety events with active agent

© 2024 Collins SP et al. *JAMA Network Open*.

eTable 4. Protocol-specified exempt serious events (PSESEs)

Protocol specified exempt serious events (PSESEs) were medical events that were systemically collected for all enrolled patients so that data on these events did not rely on *ad hoc* adverse event reporting. PSESEs were collected through day 60.

PSESE (collected through day 60)	**Definition**	**Variable type**	**Analysis approach**
Seizure	Clinically identified seizure	Binary	Frequency counts and proportions
Stroke	Clinically identified ischemic or hemorrhagic stroke	Binary	Frequency counts and proportions
Arrythmia (atrial or ventricular)	Clinically identified atrial or ventricular arrythmia	Binary	Frequency counts and proportions
Cardiomyopathy	Clinically identified cardiomyopathy	Binary	Frequency counts and proportions
Cardiac arrest	Clinically identified cardiac arrest, regardless of cardiac rhythm	Binary	Frequency counts and proportions
Myocardial injury	Clinical identified myocardial injury that was judged not to be due to acute coronary syndrome	Binary	Frequency counts and proportions
Acute coronary syndrome	Clinically identified acute coronary syndrome	Binary	Frequency counts and proportions
Hypotension	Low arterial blood pressure leading to either [1] initiation or increase in vasopressor therapy, [2] administration of a fluid bolus of 500 ml or more, or [3] modification of the dose or discontinuation of the study drug	Binary	Frequency counts and proportions
Hypertension	Elevated arterial blood pressure leading to either [1] initiation or increase in antihypertensive medications or [2] discontinuation of the study drug	Binary	Frequency counts and proportions
Hypoxemia requiring supplemental oxygen	New supplement oxygen treatment	Binary	Frequency counts and proportions
Acute respiratory distress syndrome	Clinically identified acute respiratory distress syndrome	Binary	Frequency counts and proportions
Receipt of non-invasive ventilation	New non-invasive ventilation	Binary	Frequency counts and proportions
Receipt of invasive ventilation	New invasive ventilation	Binary	Frequency counts and proportions
Receipt of extracorporeal membrane oxygenation (ECMO)	New receipt of ECMO	Binary	Frequency counts and proportions
Elevation of aspartate aminotransferase (AST)	Clinically identified AST elevations, or AST > 128U/L for Men, AST > 104U/L for Women	Binary	Frequency counts and proportions

© 2024 Collins SP et al. *JAMA Network Open*.

PSESE (collected through day 60)	**Definition**	**Variable type**	**Analysis approach**
Elevation of alanine aminotransferase (ALT)	Clinically identified ALT, or ALT > 136U/L for Men, ALT > 96U/L for Women	Binary	Frequency counts and proportions
Acute pancreatitis	Clinically identified acute pancreatitis	Binary	Frequency counts and proportions
Acute kidney injury	Clinically identified acute kidney injury or criteria for KDIGO AKI stage I or greater (serum creatinine criteria only)	Binary	Frequency counts and proportions
Receipt of renal replacement therapy	New receipt of renal replacement therapy	Binary	Frequency counts and proportions
Symptomatic hypoglycemia	Blood glucose level <60 mg/dl and clinically identified symptoms related to low blood glucose	Binary	Frequency counts and proportions
Neutropenia	Clinically identified neutropenia or ANC < 500 cells/mcl	Binary	Frequency counts and proportions
Lymphopenia	Clinically identified lymphopenia or ALC < 1000 cells/mcl	Binary	Frequency counts and proportions
Anemia	Clinically identified anemia or Hgb < 7g/dL for Men, Hgb < 6.5g/dL for Women	Binary	Frequency counts and proportions
Thrombocytopenia	Clinically identified thrombocytopenia or platelets < 100 thousand/L	Binary	Frequency counts and proportions
Venous thromboembolism	Clinically identified new deep vein thrombosis or pulmonary embolism	Binary	Frequency counts and proportions
Severe dermatologic reaction	New severe skin reaction (e.g., Stevens-Johnson Syndrome)	Binary	Frequency counts and proportions

© 2024 Collins SP et al. *JAMA Network Open*.

eTable 5. Reasons for not receiving full 28-doses of fostamatinib

Dose status	Dose Detail	Reason for discontinuation	Combined Doses (N = 10864)	Placebo Doses (N = 5292)	Active Doses (N = 5572)
**Taken**			**7867**	**3850**	**4017**
	In-hospital		5059	2334	2725
	Outpatient		2808	1516	1292
**Not taken**			**2275**	**1116**	**1159**
	Death		199	107	92
	No reason given		399	162	237
	Permanent discontinuation [a]		*1360*	*706*	*654*
		AE/PSESE/ADR	*594*	*293*	*301*
		Logistical	*51*	*36*	*15*
		Patient refusal	*498*	*192*	*306*
		Other	*246*	*189*	*57*
		Clinical events (other than AE/PSESE)	*98*	*98*	*0*
	Temporary discontinuation [a]		317	141	176
		AE/PSESE	*120*	*35*	*85*
		Logistical	*76*	*47*	*29*
		Patient refusal	*39*	*11*	*28*
		Other	*109*	*65*	*44*
		Clinical events (other than AE/PSESE)	*72*	*55*	*17*
**Uncertain due to loss to follow-up**			**722**	**326**	**396**

[a] Discontinuation reasons are not mutually exclusive

eTable 6. Adverse events (AEs) in Fostamatinib trial

The trial protocol instructed investigators to record adverse events (AEs) that met any of the following criteria: (1) serious and definitely or possibly related (regardless of PSESE status); (2) unexpected and definitely or possibly related (regardless of PSESE status);(3) serious and not a PSESE; (4) definitely or possibly related or of uncertain relationship and not a PSESE; (4) severity grade 3 or 4 clinical adverse event and not a PSESE. Some lower grade adverse events were reported. A serious adverse event (SAE) was defined as an adverse event leading to death, a life-threatening experience, prolongation of inpatient hospitalization or re-hospitalization, or persistent or significant disability or incapacity. Site investigators reported the suspected relatedness of each adverse event to study procedures using the following 5 options: definitely related; probably related; possibly related; probably not related; definitely not related; uncertain relationship. In the Fostamatinib active agent group, 132 total adverse events were reported. In the placebo group of the Fostamatinib trial, 123 total adverse events were reported.

Patients with Adverse Events (AEs) in the Fostamatinib trial		
	**Fostamatinib (n = 199)**	**Placebo (n = 201)**
Grade 1: patients with ≥1 grade 1 AE, no. (%)	13 (6.5)	5 (2.5)
Grade 2: patients with ≥1 grade 2 AE, no. (%)	11 (5.5)	13 (6.5)
Grade 3: patients with ≥1 grade 3 AE, no. (%)	30 (15.1)	18 (9.0)
Grade 4: patients with ≥1 grade 4 AE, no. (%)	13 (6.5)	12 (6.0)
Grade 5: patients with ≥1 grade 5 AE, no. (%)	3 (1.5)	8 (4.0)
Serious AE: patients with ≥1 SAE, no. (%)	33 (16.6)	33 (16.4)
Any AESI; AST or ALT elevation >5x the larger of the local laboratory ULN or baseline (AST/ALT at day 0)	6 (3.0)	4 (2.0)
AST or ALT elevation >10x the larger of the local laboratory ULN or baseline (AST/ALT at day 0)	1 (0.5)	3 (1.5)

© 2024 Collins SP et al. *JAMA Network Open*.

eTable 7. Frequency of other safety outcomes and PSESEs

PSESE = protocol-specified exempt serious events, which were systematically collected events captured through day 60

Fostamatinib Safey Outcomes and PSESEs	**Fostamatinib**, N = 199[1]	**Placebo**, N = 201[1]
**Safety outcomes to Day 28**		
ALT > 136U/L for Men, ALT > 96U/L for Women	14 (7.0)	14 (7.0)
AST > 128U/L for Men, AST > 104U/L for Women	23 (11.6)	11 (5.5)
ANC < 500 cells/mcl	17 (8.5)	9 (4.5)
Hypertension	24 (12.1)	19 (9.5)
**PSESEs to Day 60**		
Seizure	1 (0.5)	1 (0.5)
Stroke	0 (0.0)	1 (0.5)
Atrial or ventricular arrhythmia event	16 (8.0)	9 (4.5)
Cardiomyopathy event	2 (1.0)	0 (0.0)
Cardiac arrest event	2 (1.0)	3 (1.5)
Myocardial injury event	0 (0.0)	1 (0.5)
Acute coronary syndrome	0 (0.0)	1 (0.5)
Hypotension	26 (13.1)	18 (9.0)
Hypertension	24 (12.1)	19 (9.5)
Hypoxemia requiring supplemental oxygen	16 (8.0)	10 (5.0)
Acute respiratory distress syndrome	9 (4.5)	6 (3.0)
Non-invasive ventilation	21 (10.6)	20 (10.0)
Invasive mechanical ventilation	26 (13.1)	23 (11.4)
ECMO	0 (0.0)	2 (1.0)
ALT > 136U/L for Men, ALT > 96U/L for Women	14 (7.0)	14 (7.0)
AST > 128U/L for Men, AST > 104U/L for Women	23 (11.6)	11 (5.5)
Acute pancreatitis	0 (0.0)	1 (0.5)
KDIGO AKI Stage 1	40 (20.1)	30 (14.9)
Incident renal replacement therapy through 28 days	5 (2.5)	9 (4.5)
Symptomatic hypoglycemia	1 (0.5)	0 (0.0)
ANC < 500 cells/mcl	17 (8.5)	9 (4.5)
ALC < 1000 cells/mcl	110 (55.3)	119 (59.2)
Hgb < 7g/dL for Men, Hgb < 6.5g/dL for Women	12 (6.0)	6 (3.0)
Platelets < 100 G/L	32 (16.1)	24 (11.9)
Venous thromboembolism	4 (2.0)	9 (4.5)
Severe dermatologic reaction	0 (0.0)	0 (0.0)

[1]n (%)

© 2024 Collins SP et al. *JAMA Network Open*.

eFigure 1. Timeline of trials enrolling as part of the ACTIV-4 Host Tissue Platform

The figure displays the timeline of the ACTIV-4 Host Tissue platform including enrollment periods for each trial and the shared populations.

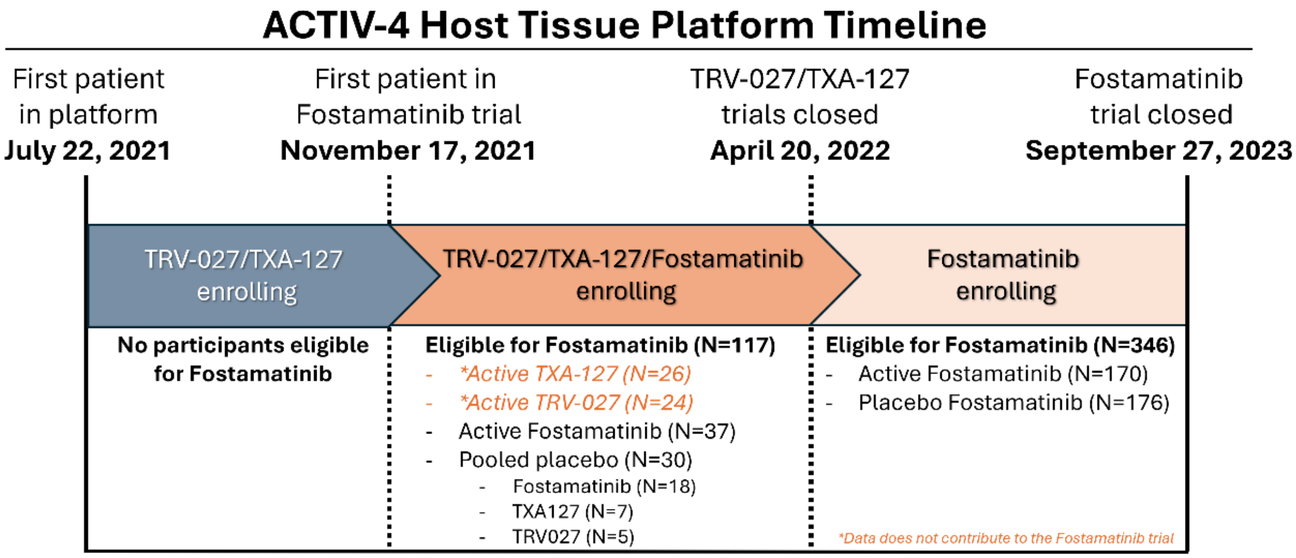

© 2024 Collins SP et al. *JAMA Network Open*.

eFigure 2. Assessment of the proportional odds assumption using repeated dichotomizations

The dots represent the Adjusted Odds Ratio comparing Fostamatinib versus placebo in 29 successive logistic regression models with the oxygen-free days outcome dichotomized at the x-axis value. The shaded region represents the 95% CI for each estimate.

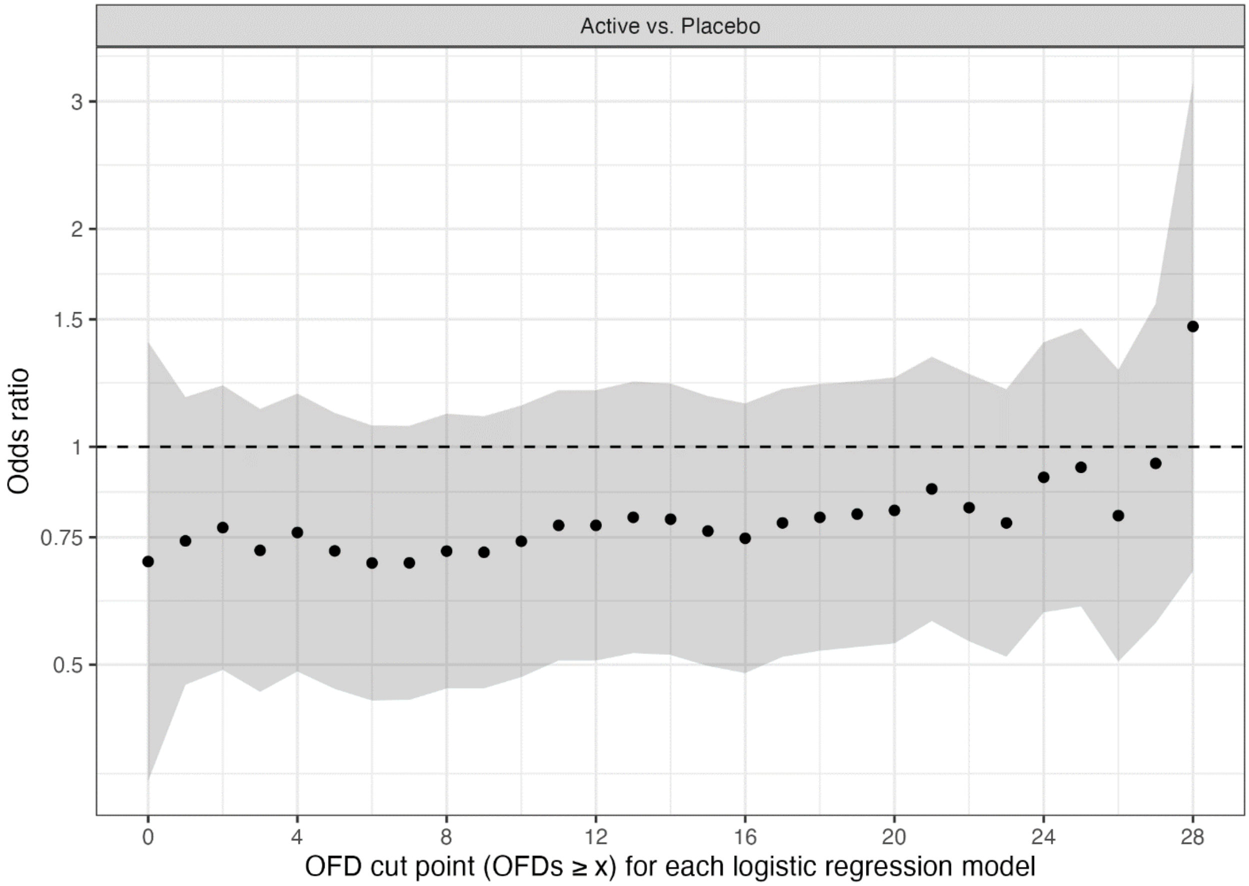

© 2024 Collins SP et al. *JAMA Network Open*.

eFigure 3. Distribution of Oxygen-free days in Fostamatinib trial

The histograms display the proportion of patients at day 28 in each of the 30 categories of the oxygen-free days ordinal outcome, stratified by Fostamatinib study drug vs placebo. The table below the histograms displays summary statistics for the oxygen-free days outcome. Adjusted Odds Ratio comparing distribution of oxygen-free days in Fostamatinib group vs placebo: aOR: 0.82; 95% CI: 0.58 to 1.17 (aOR <1.0 is in the direction of inferiority for Fostamatinib compared to placebo).

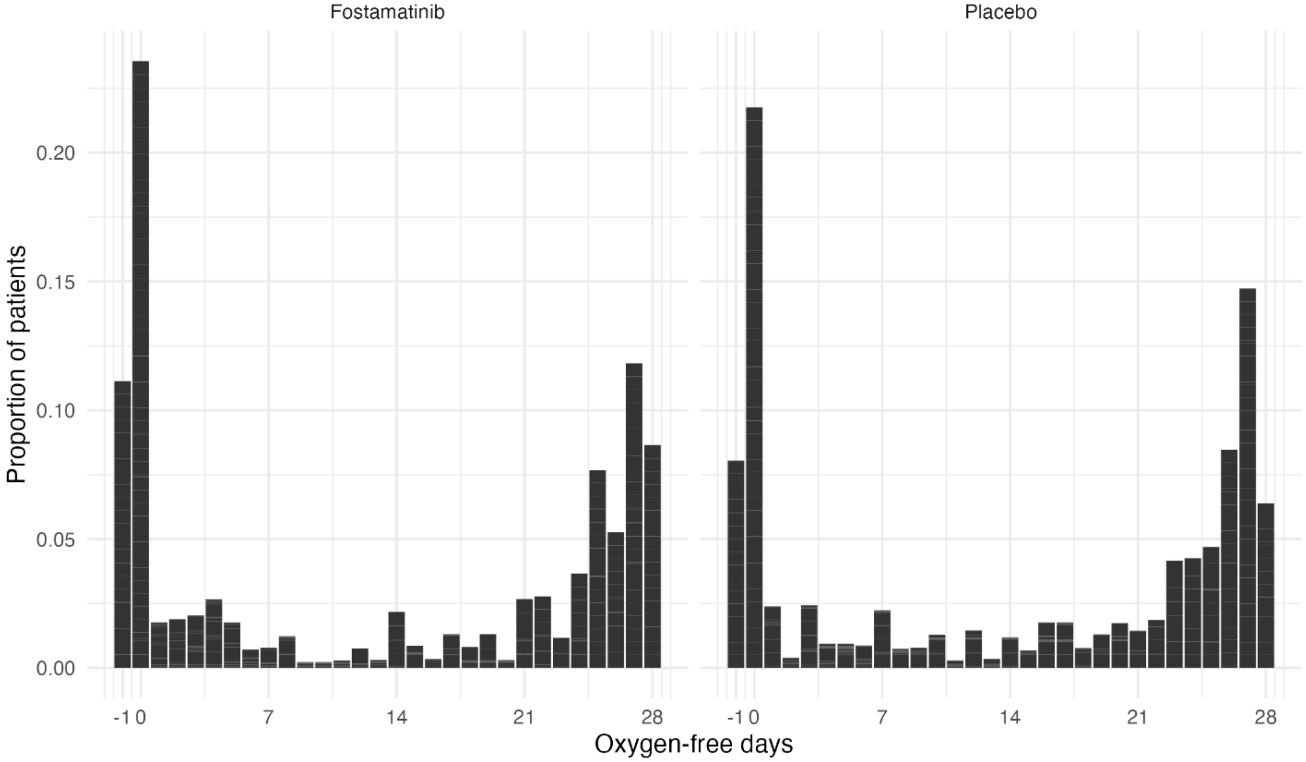

	Fostamatinib (n=199)	Placebo (n=201)	Adjusted absolute difference (95% CI)
Mean oxygen-free days	13.4	14.2	1.26 (-3.52 - 1.00)
Median (IQR) oxygen-free days	17 (0 – 26)	19 (0 – 26)	-2 (-9 – 2)
-1 oxygen-free days (dead), no. (%)	22 (11.1)	16 (8.0)	Difference in percentage: 3.16 (-2.65 - 9.06)
0 OFDs (oxygen use for 28 days), no. (%)	45 (22.6)	43 (21.4)	Difference in percentage: 1.22 (-6.88 - 9.33)
Patients with partially observed oxygen-free days, no (%)	22 (11.1)	20 (10.0)	N/A

© 2024 Collins SP et al. *JAMA Network Open*.

eFigure 4. Treatment effect on primary, secondary, and exploratory outcomes

The figure displays the 95% CI for each outcome, color coded according to primary, key secondary, and other secondary outcomes. The intervals are grouped into panels such that the odds ratio interpretation is consistent with each panel, i.e., either higher OR is beneficial or the opposite.

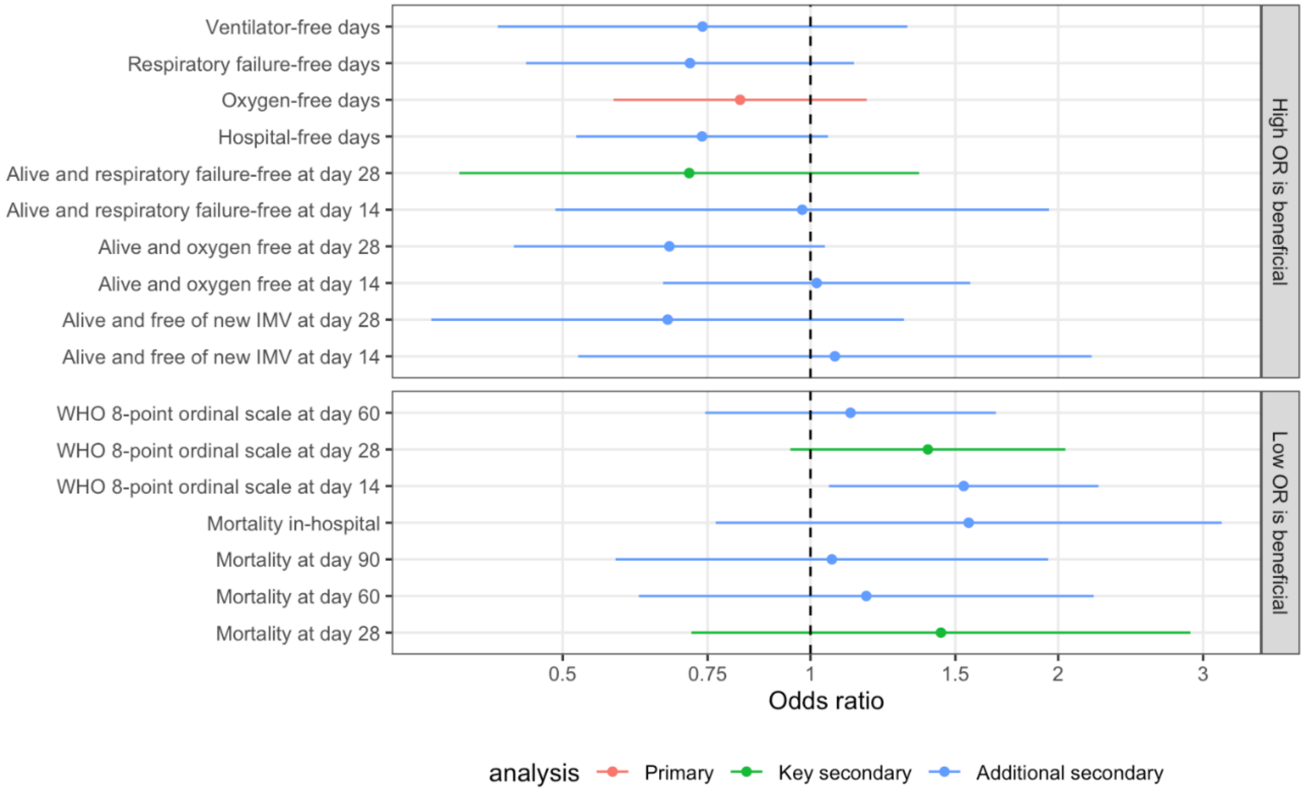

© 2024 Collins SP et al. *JAMA Network Open*.

eAppendix 2. Study Drug Dose Modification Considerations and Stopping Rules

Dose Modifications: The following dose modifications (from the master protocol) were utilized in patients randomized to the fostamatinib arm (active or placebo). Those patients who had doses held only completed 14 days (up to 28 doses) of study treatment; dosing did not extend beyond day 14. Blood pressure was monitored daily in the hospital while on study drug up until the time of discharge.

Hypertension:

Blood pressure >140/90 should be treated with antihypertensives per usual care.

During the inpatient hospitalization: If systolic BP remains > 160 mmHg or diastolic BP > 100 mmHg or higher despite antihypertensive therapy, interrupt study drug. When restarting study drug once BP is below 160 mmHg systolic and 100 mmHg diastolic, drug dose should be reduced to 100 mg twice daily or matching placebo for the remainder of the study.

- **At the time of Hospital discharge:** Patients **still on study drug** at the time of hospital discharge will have the following study procedures performed:

Schematic overview of blood pressure considerations and procedures performed for patients still on study drug at the time of hospital discharge

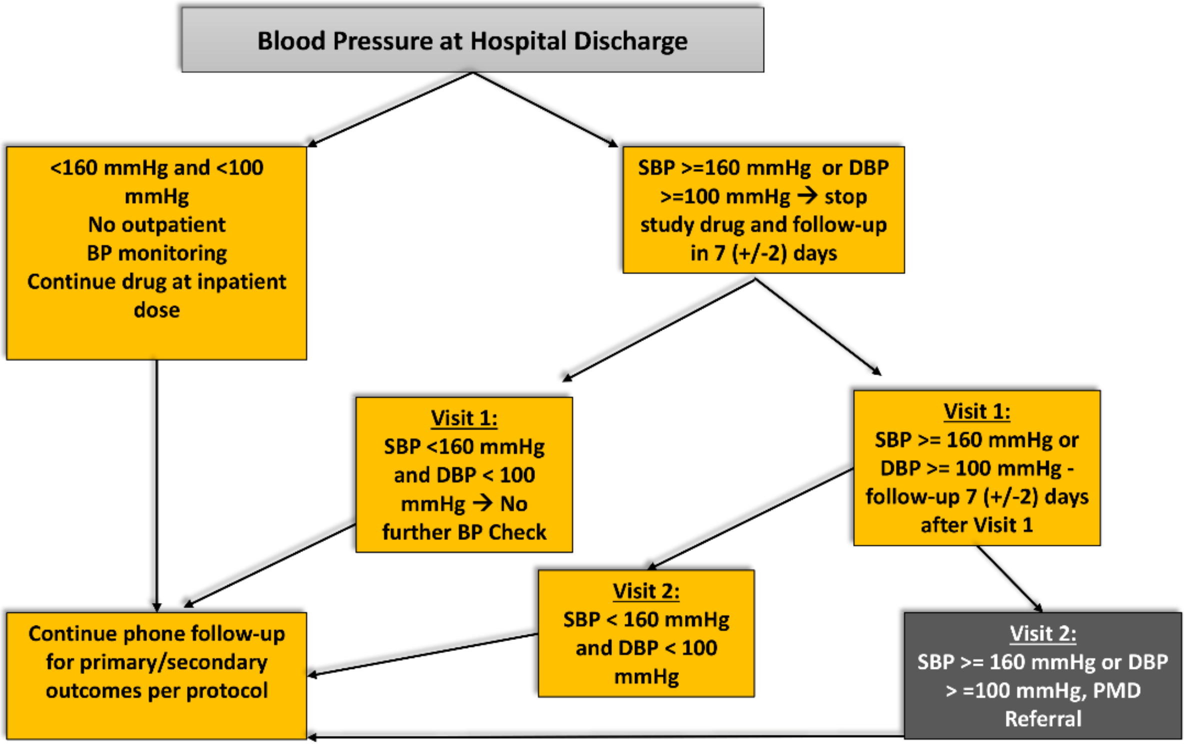

BP = Blood Pressure | SBP = Systolic Blood Pressure | DBP = Diastolic Blood Pressure

Those with SBP < 160 mmHg and DBP < 100 mmHg will continue on study drug at the inpatient dose and have no further BP measurements for study purposes

Those with SBP ≥ 160 mmHg or DBP ≥ 100 mmHg at the time of hospital discharge will have study medication stopped and undergo the following repeat testing:

1. 7 (+/-2) days after post-hospital discharge a repeat blood pressure will be obtained.

a. If the SBP is < 160 mmHg and DBP is < 100 mmHg no further BP monitoring will be performed for study purposes

© 2024 Collins SP et al. *JAMA Network Open*.

b. If the SBP is ≥ 160 mmHg or the DBP is ≥ 100 mmHg a follow-up will be performed in 7 (+/-2) days after the prior visit

   i. At the repeat visit if the SBP is < 160 mmHg and DBP is < 100 mmHg no further BP monitoring will be performed for study purposes

   ii. At the repeat visit if the SBP is ≥ 160 mmHg or DBP is ≥ 100 mmHg study guidance will recommend BP recheck by a healthcare provider

Hepatotoxicity:

Inpatient monitoring: Liver function tests (LFT's) will be checked daily during hospitalization while on study drug.

1. Study drug should be stopped if there is an increase in either:

   a. AST or ALT greater than or equal to 5-times the upper limit of normal at the local lab or for those with elevated AST and ALT at the time of enrollment an increase to 5-times the level at enrollment

   b. AST or ALT to greater than or equal to 3-times the upper limit of normal at the local lab AND total bilirubin to greater than or equal to 2-times the upper limit of normal at the local lab.

Elevated unconjugated (indirect) bilirubin in absence of other LFT abnormalities – continue study drug with frequent monitoring since isolated increase in unconjugated (indirect) bilirubin may be due to UGT1A1 inhibition.

Outpatient monitoring: Patients discharged while on study drug with AST/ALT or bilirubin elevated > 2x the upper reference limits or up trending from most recent value (but less than the inpatient stopping criteria) AND patients discharged not on study drug with either AST or ALT at least 3-times the upper limit of normal or total bilirubin at least 2-times the upper limit of normal at the time of discharge will have the following study procedures performed:

1. 7 (+/-2) days post-hospital discharge a repeat measurement of AST, ALT and total bilirubin will be obtained.

   a. Patients NOT on study drug at the time of repeat LFT measurement:

*If these repeat measurements are less than 3-times the upper limit of normal for AST or ALT and less than 2-times the upper limit of normal for total bilirubin, then no further study procedures will be performed related to LFT monitoring.

*If these repeat measurements are greater than 3-times the upper limit of normal for AST or ALT or greater than 2-times the upper limit of normal for total bilirubin, then a repeat measurement of AST, ALT and total bilirubin should be obtained 7 (+/-2) days later. If the AST or ALT remain above 3-times the upper limit of normal or total bilirubin is above 2-times the upper limit of normal after this repeat testing 7 days later, LFTs should be monitored per standard of care by a provider or if no provider followed-up by the study team.

   b. Patients ON study drug at the time of repeat LFT measurement:

*If these repeat LFT measurements are below the upper reference limit of normal continue study drug to completion and no recheck is needed.

*If these measurements remain above the upper reference limit of normal for either AST, ALT or total bilirubin but do not meet inpatient stopping criteria (defined above), continue study drug and a repeat measurement of AST, ALT and total bilirubin should be obtained 7 (+/-2) days later. If this repeat testing remains above the upper limit of normal for AST, ALT or bilirubin it should be monitored per standard care by a provider or if no provider followed-up by the study team.

*If study drug is stopped (per inpatient stopping rules above) repeat measurement of AST, ALT and total bilirubin should be obtained 7 (+/-2) days later. If this repeat testing remains above the upper limit of normal for AST, ALT or bilirubin it should be monitored per standard care by a provider or if no provider followed-up by the study team.

© 2024 Collins SP et al. *JAMA Network Open*.

Once study drug is stopped for abnormal LFT values, the study drug will not be restarted.

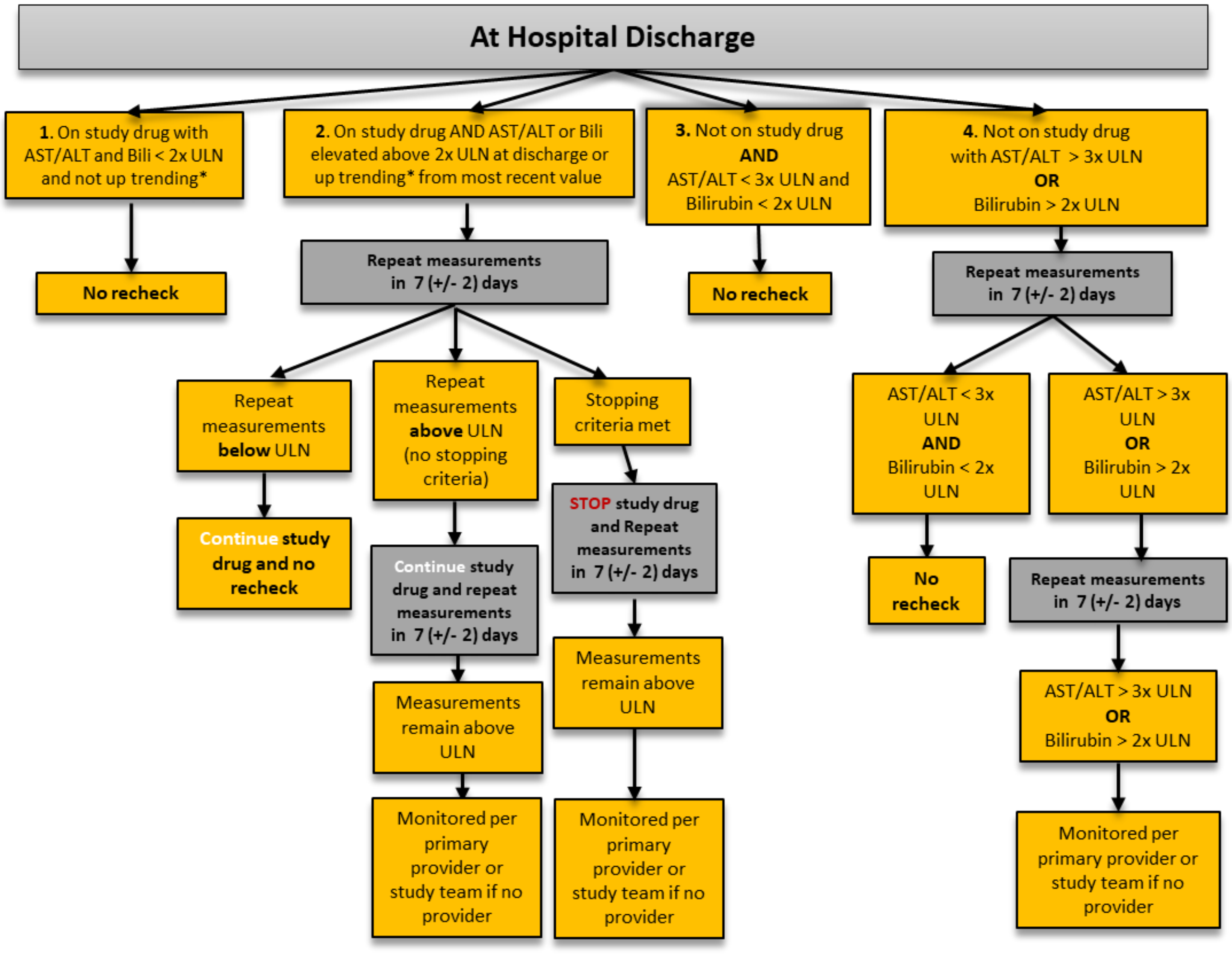

*up trending is defined as a >25% increase in LFTs from the day prior

<u>Diarrhea:</u> If symptoms become severe (grade 3 or above) while the patient is hospitalized, temporarily stop study drug until symptoms resolve to mild (grade 1). When restarting, study drug dose should be reduced to 100 mg twice daily or matching placebo for the remainder of the study. Study drug should be stopped if diarrhea becomes severe in the outpatient setting.

<u>Neutropenia:</u>
If the absolute neutrophil count (ANC) decreases to less than 1.0x10^9/L, the study drug should be discontinued.

- If the ANC returns to above 1.0x10^9/L, the study drug may be restarted. When restarting the study drug, the dose should be reduced to 100 mg twice daily or matching placebo for the remainder of the study.
- In patients who restart study drug at 100 mg BID after neutropenia resolves within the 14-day window, and labs are not rechecked during hospitalization, the team must either: 1) repeat CBC with differential within 7 days of discharge OR 2) not restart the medication if they are unable to repeat CBC with differential in this 7-day time frame.
- A CBC with differential will be checked daily while the patient is in the hospital and on study drug. If the patient is discharged with an ANC < 1.0x10^9/L then a repeat CBC with differential will be performed within 7 days of hospital discharge and repeated weekly for 4 weeks or until the ANC is >1.0x10^9/L.

© 2024 Collins SP et al. *JAMA Network Open*.

- If a patient is found to have an ANC <1.0x10^9/L as part of routine care after hospital discharge while still in the follow-up period, then a CBC with differential should be checked weekly for 4 weeks or until the ANC is >1.0x10^9/L.
- Study drug is not restarted when the ANC remains <1.0x10^9/L Those who are discharged with a normal ANC require no further measurement after discharge.

© 2024 Collins SP et al. *JAMA Network Open*.
