# Supplementary material for: Fostamatinib for Hospitalized Adults With COVID-19 and Hypoxemia: A Randomized Clinical Trial
Source: JAMA Netw Open. 2024 Dec 3;7(12):e2448215. doi: 10.1001/jamanetworkopen.2024.48215 (PMC11615712; doi:10.1001/jamanetworkopen.2024.48215)
Supplement: Supplement 3. — ACTIV-4 Host Tissue Investigators [file jamanetwopen-e2448215-s003.pdf]

Supplemental Online Content: ACTIV-4 Host Tissue Investigators
*First name, last name, and suffix (if applicable) are required and will appear in PubMed.

| *Group Name(s): ACTIV-4 Host Tissue Investigators | | | | | | | |
|---|---|---|---|---|---|---|---|
| ***First Name and Middle Initial(s)** | ***Last Name** | ***Suffix (eg, Jr, III)** | Academic Degrees | Institution | Location (city, state/province, country) | Role or Contribution, eg, chair, principal investigator | Group (if more than 1 Group listed in the byline) and/or Subgroup (eg, Steering Committee) |
| Richard C. | Becker | | MD | University of Cincinnati | Cincinnati, OH, USA | DSMB Chair | |
| Gregory | del Zoppo | | MD | University of Washington | Seattle, WA, USA | DSMB Member | |
| Peter | Henke | | MD | University of Michigan | Ann Arbor, MI, USA | DSMB Member | |
| Richard | Holubkov | | PhD | University of Utah | Salt Lake City, UT, USA | DSMB Member | |
| Maryl | Johnson | | MD | University of Wisconsin | Madison, WI, USA | DSMB Member | |
| Kim | Kerr | | MD | University of San Diego | La Jolla, CA, USA | DSMB Member | |
| Hannah I. | Lipman | | MD | Hackensack University Medical Center | Hackensack, NJ, USA | DSMB Member | |
| Fedor | Lurie | | MD, PhD | University of Michigan | Ann Arbor, MI, USA | DSMB Member | |
| Bertram | Pitt | | MD | University of Michigan Health System | Ann Arbor, MI, USA | DSMB Member | |
| Sara K. | Vesely | | PhD | University of Oklahoma | Oklahoma City, OK, USA | DSMB Member | |
| Jerome L. | Fleg | | MD | National Heart, Lung, and Blood Insti | Bethesda, MD, USA | DSMB Executive Secretary | |
| Dave | Aamodt | | | Vanderbilt University Medical Center | Nashville, TN, USA | Coordinating Center | |
| Debra | Clark | | | Vanderbilt University Medical Center | Nashville, TN, USA | Coordinating Center | |
| Jessica B. | Collins | | | Vanderbilt University Medical Center | Nashville, TN, USA | Coordinating Center | |
| Sheri | Dixon | | RN, BSN | Vanderbilt University Medical Center | Nashville, TN, USA | Coordinating Center | |
| Maya | Cook | | | Vanderbilt University Medical Center | Nashville, TN, USA | Coordinating Center | |
| Carly | Gatewood | | | Vanderbilt University Medical Center | Nashville, TN, USA | Coordinating Center | |
| John | Graves | | | Vanderbilt University Medical Center | Nashville, TN, USA | Coordinating Center | |

| ***First Name and Middle Initial(s)** | ***Last Name** | ***Suffix (eg, Jr, III)** | Academic Degrees | Institution | Location (city, state/province, country) | Role or Contribution, eg, chair, principal investigator | Group (if more than 1 Group listed in the byline) and/or Subgroup (eg, Steering Committee) |
|---|---|---|---|---|---|---|---|
| Debbie | Hunter | | | Vanderbilt University Medical Center | Nashville, TN, USA | Coordinating Center | |
| Courtney | Jordan | | | Vanderbilt University Medical Center | Nashville, TN, USA | Coordinating Center | |
| Sahar | Ko | | | Vanderbilt University Medical Center | Nashville, TN, USA | Coordinating Center | |
| Ugo (Ben) | Lekqauwa | | | Vanderbilt University Medical Center | Nashville, TN, USA | Coordinating Center | |
| Itzel | Lopez | | | Vanderbilt University Medical Center | Nashville, TN, USA | Coordinating Center | |
| David | McKeel | | | Vanderbilt University Medical Center | Nashville, TN, USA | Coordinating Center | |
| Dirk | Orozco | | | Vanderbilt University Medical Center | Nashville, TN, USA | Coordinating Center | |
| Nelson | Prato | | | Vanderbilt University Medical Center | Nashville, TN, USA | Coordinating Center | |
| Ally | Qi | | MPH | Vanderbilt University Medical Center | Nashville, TN, USA | Coordinating Center | |
| Kalley | Stagner | | | Vanderbilt University Medical Center | Nashville, TN, USA | Coordinating Center | |
| Krista | Vermillion | | | Vanderbilt University Medical Center | Nashville, TN, USA | Coordinating Center | |
| Stephanie | Winchell | | | Vanderbilt University Medical Center | Nashville, TN, USA | Coordinating Center | |
| Taylor | Young | | | Vanderbilt University Medical Center | Nashville, TN, USA | Coordinating Center | |
| Esteban | Masuda | | | Rigel Pharmaceuticals, Inc. | San Fransciso, CA | Industry partner for drug supply | |
| Asif | Siddiqui | | | Rigel Pharmaceuticals, Inc. | San Fransciso, CA | Industry partner for drug supply | |

*First name, last name, and suffix (if applicable) are required and will appear in PubMed.

| ***First Name and Middle Initial(s)** | ***Last Name** | ***Suffix (eg, Jr, III)** | Academic Degrees | Institution | Location (city, state/province, country) | Role or Contribution, eg, chair, principal investigator | Group (if more than 1 Group listed in the byline) and/or Subgroup (eg, Steering Committee) |
|---|---|---|---|---|---|---|---|
| Caroline | Brooks | | | Research Organization (KC) LTD (ROKC) | London, UK | Ex-U.S. CRO partner | |
| Carl | Fletcher | | | Research Organization (KC) LTD (ROKC) | London, UK | Ex-U.S. CRO partner | |
| Annie | Duffy | | | Research Organization (KC) LTD (ROKC) | London, UK | Ex-U.S. CRO partner | |
| Paul | Walsh | | | Research Organization (KC) LTD (ROKC) | London, UK | Ex-U.S. CRO partner | |
| Jack | Moody | | | Research Organization (KC) LTD (ROKC) | London, UK | Ex-U.S. CRO partner | |
| Toni | Sobande | | | Research Organization (KC) LTD (ROKC) | London, UK | Ex-U.S. CRO partner | |
| Amina | Ali | | | Research Organization (KC) LTD (ROKC) | London, UK | Ex-U.S. CRO partner | |
| Yomi | Henry | | | Research Organization (KC) LTD (ROKC) | London, UK | Ex-U.S. CRO partner | |
| Frances | Ogueri | | | Research Organization (KC) LTD (ROKC) | London, UK | Ex-U.S. CRO partner | |
| Alba | Sierto | | | Research Organization (KC) LTD (ROKC) | London, UK | Ex-U.S. CRO partner | |
| Maria | Sanchez Grande | | | Research Organization (KC) LTD (ROKC) | London, UK | Ex-U.S. CRO partner | |
| Chiara | Spinello | | | Research Organization (KC) LTD (ROKC) | London, UK | Ex-U.S. CRO partner | |
| Olufunke | Anthony | | | Research Organization (KC) LTD (ROKC) | London, UK | Ex-U.S. CRO partner | |
| Michal | Sieracki | | | Research Organization (KC) LTD (ROKC) | London, UK | Ex-U.S. CRO partner | |
| Michaella | Tomlin | | | Research Organization (KC) LTD (ROKC) | London, UK | Ex-U.S. CRO partner | |
| Anton | Pozniak | | M.D. | NEAT ID | Brussels, Belgium | Ex-U.S. Sponsor | |

*First name, last name, and suffix (if applicable) are required and will appear in PubMed.

| ***First Name and Middle Initial(s)** | ***Last Name** | ***Suffix (eg, Jr, III)** | Academic Degrees | Institution | Location (city, state/province, country) | Role or Contribution, eg, chair, principal investigator | Group (if more than 1 Group listed in the byline) and/or Subgroup (eg, Steering Committee) |
|---|---|---|---|---|---|---|---|
| Tim | Leaver | | | NEAT ID | Brussels, Belgium | Ex-U.S. Sponsor | |
| Polly | Parks | | | NEAT ID | Brussels, Belgium | Ex-U.S. Sponsor | |
| Jakea | Johnson | | MPH | Vanderbilt University Medical Center | Nashville, TN, USA | Enrolling site study personnel | |
| Ryan | Walsh | | MD | Vanderbilt University Medical Center | Nashville, TN, USA | Enrolling site study personnel | |
| Brian | Bales | | MD | Vanderbilt University Medical Center | Nashville, TN, USA | Enrolling site study personnel | |
| Karen | Miller | | RN | Vanderbilt University Medical Center | Nashville, TN, USA | Enrolling site study personnel | |
| Donna | Torr | | PharmD | Vanderbilt University Medical Center | Nashville, TN, USA | Enrolling site study personnel | |
| Clark | Files | | MD | Wake Forest University | Winston-Salem, NC, USA | Enrolling site study personnel | |
| Kevin | Gibbs | | MD | Wake Forest University | Winston-Salem, NC, USA | Enrolling site study personnel | |
| Darija | Ward | | MBA | Wake Forest University | Winston-Salem, NC, USA | Enrolling site study personnel | |
| Leigha | Landreth | | RN | Wake Forest University | Winston-Salem, NC, USA | Enrolling site study personnel | |
| Lisa | Parks | | RN | Wake Forest University | Winston-Salem, NC, USA | Enrolling site study personnel | |
| Lori | Flores | | NP | Wake Forest University | Winston-Salem, NC, USA | Enrolling site study personnel | |
| J. Pedro | Teixeira | | | University of New Mexico Health Sciences Center | Albuquerque, NM, USA | Enrolling site study personnel | |
| Sandra | Cardenas | | | University of New Mexico Health Sciences Center | Albuquerque, NM, USA | Enrolling site study personnel | |
| Juan A. | Ceniceros | | | University of New Mexico Health Sciences Center | Albuquerque, NM, USA | Enrolling site study personnel | |

*First name, last name, and suffix (if applicable) are required and will appear in PubMed.

| ***First Name and Middle Initial(s)** | ***Last Name** | ***Suffix (eg, Jr, III)** | Academic Degrees | Institution | Location (city, state/province, country) | Role or Contribution, eg, chair, principal investigator | Group (if more than 1 Group listed in the byline) and/or Subgroup (eg, Steering Committee) |
|---|---|---|---|---|---|---|---|
| Amy G. | Cunningham | | | University of New Mexico Health Sciences Center | Albuquerque, NM, USA | Enrolling site study personnel | |
| Susan | Kunkel | | | University of New Mexico Health Sciences Center | Albuquerque, NM, USA | Enrolling site study personnel | |
| Debbie M. | Lovato | | | University of New Mexico Health Sciences Center | Albuquerque, NM, USA | Enrolling site study personnel | |
| Brooklin | Zimmerman | | MSN | University of Nebraska Medical Center | Omaha, NE, USA | Enrolling site study personnel | |
| Thanh | Nguyen | | PhD, MSN, FNP-C | University of Nebraska Medical Center | Omaha, NE, USA | Enrolling site study personnel | |
| Wesley | Zeger | | DO | University of Nebraska Medical Center | Omaha NE 68105 | Enrolling site study personnel | |
| Noah | Wiedel | | MD | University of Nebraska Medical Center | Omaha NE 68105 | Enrolling site study personnel | |
| Stephanie | Stilinovic | | BSN, RN | Washington University | St. Louis, MO, USA | Enrolling site study personnel | |
| Caroline | Foster | | | Washington University | St. Louis, MO, USA | Enrolling site study personnel | |
| Jeanne | Flannigan | | | Washington University | St. Louis, MO, USA | Enrolling site study personnel | |
| Carolyn | Brokowski | | | Yale University | New Haven, CT, USA | Enrolling site study personnel | |
| Jing | Lu | | | Yale University | New Haven, CT, USA | Enrolling site study personnel | |
| Muriel | Solberg | | | Yale University | New Haven, CT, USA | Enrolling site study personnel | |
| Dana | Lee | | | Yale University | New Haven, CT, USA | Enrolling site study personnel | |
| Brian | Tiffany | | MD, PhD | Dignity Health Research Institute | Chandler, AZ, USA | Enrolling site study personnel | |

*First name, last name, and suffix (if applicable) are required and will appear in PubMed.

| ***First Name and Middle Initial(s)** | ***Last Name** | ***Suffix (eg, Jr, III)** | Academic Degrees | Institution | Location (city, state/province, country) | Role or Contribution, eg, chair, principal investigator | Group (if more than 1 Group listed in the byline) and/or Subgroup (eg, Steering Committee) |
|---|---|---|---|---|---|---|---|
| Charlotte | Tanner | | RN, BSN | Dignity Health Research Institute | Chandler, AZ, USA | Enrolling site study personnel | |
| Annette | Taylor | | RN, BSN, CCRC | Dignity Health Research Institute | Chandler, AZ, USA | Enrolling site study personnel | |
| Jennine | Zumbahl | | RN, MSHA, CCRC, CRCP | Dignity Health Research Institute | Chandler, AZ, USA | Enrolling site study personnel | |
| Aamer | Syed | | MD | Virginia Commonwealth University | Richmond, VA, USA | Enrolling site study personnel | |
| Jessica | Mason | | MPH | Virginia Commonwealth University | Richmond, VA, USA | Enrolling site study personnel | |
| Patrick E. H. | Jackson | | MD | University of Virginia | Charlottesville, VA, USA | Enrolling site study personnel | |
| Rachael W. | Coleman | | MPH | University of Virginia | Charlottesville, VA, USA | Enrolling site study personnel | |
| Heather M. | Haughey | | PhD | University of Virginia | Charlottesville, VA, USA | Enrolling site study personnel | |
| Kartik | Cherabuddi | | MD | University of Florida, Gainesville | Gainesville, FL, USA | Enrolling site study personnel | |
| Rebecca | Wakeman | | MSW CCRC | University of Florida, Gainesville | Gainesville, FL, USA | Enrolling site study personnel | |
| Kathryn | Fairbank-Haynes | | CRC | Newton-Wellesley Hospital | Newton, MA, USA | Enrolling site study personnel | |
| Angela J. | Rogers | | MD, MPH | Stanford University | Stanford, CA, USA | Enrolling site study personnel | |
| Jennifer G. | Wilson | | MD, MS | Stanford University | Stanford, CA, USA | Enrolling site study personnel | |
| Rosemary | Vojnik | | BS | Stanford University | Stanford, CA, USA | Enrolling site study personnel | |

*First name, last name, and suffix (if applicable) are required and will appear in PubMed.

| ***First Name and Middle Initial(s)** | ***Last Name** | ***Suffix (eg, Jr, III)** | Academic Degrees | Institution | Location (city, state/province, country) | Role or Contribution, eg, chair, principal investigator | Group (if more than 1 Group listed in the byline) and/or Subgroup (eg, Steering Committee) |
|---|---|---|---|---|---|---|---|
| Cynthia | Perez | | BS | Stanford University | Stanford, CA, USA | Enrolling site study personnel | |
| David | Wyles | | MD | Denver Health Medical Center | Denver, CO, USA | Enrolling site study personnel | |
| Terra D. | Hiller | | RN, MSN | Denver Health Medical Center | Denver, CO, USA | Enrolling site study personnel | |
| Judy L. | Oakes | | PhD | Denver Health Medical Center | Denver, CO, USA | Enrolling site study personnel | |
| Ana Z. | Garcia | | BS | Denver Health Medical Center | Denver, CO, USA | Enrolling site study personnel | |
| Flora | Martinez | | MPH, RN | University of Colorado | Aurora, CO, USA | Enrolling site study personnel | |
| Jennifer | Fickes-Siler | | | University of Colorado | Aurora, CO, USA | Enrolling site study personnel | |
| David J. | Douin | | MD | University of Colorado | Aurora, CO, USA | Enrolling site study personnel | |
| Amiran | Baduashvili | | MD | University of Colorado | Aurora, CO, USA | Enrolling site study personnel | |
| Henry | Kramer | | | University of Colorado | Aurora, CO, USA | Enrolling site study personnel | |
| Lakshmi | Chauhan | | MD | University of Colorado | Aurora, CO, USA | Enrolling site study personnel | |
| Amanda | Martinez | | | University of Colorado | Aurora, CO, USA | Enrolling site study personnel | |
| Jennifer | Peers | | | University of Colorado | Aurora, CO, USA | Enrolling site study personnel | |
| Kristine | Schauer | | | University of Colorado | Aurora, CO, USA | Enrolling site study personnel | |
| Lani | Finck | | MPH | University of Colorado | Aurora, CO, USA | Enrolling site study personnel | |

*First name, last name, and suffix (if applicable) are required and will appear in PubMed.

| ***First Name and Middle Initial(s)** | ***Last Name** | ***Suffix (eg, Jr, III)** | Academic Degrees | Institution | Location (city, state/province, country) | Role or Contribution, eg, chair, principal investigator | Group (if more than 1 Group listed in the byline) and/or Subgroup (eg, Steering Committee) |
|---|---|---|---|---|---|---|---|
| Jill | Bastman | | BSN, RN | University of Colorado | Aurora, CO, USA | Enrolling site study personnel | |
| Ashley | Licursi | | PAC | University of Colorado | Aurora, CO, USA | Enrolling site study personnel | |
| Sharon | Hayes | | RN | Beth Israel Deaconess Medical Center | Boston, MA, USA | Enrolling site study personnel | |
| Nicholas | Kurtzman | | MD | Beth Israel Deaconess Medical Center | Boston, MA, USA | Enrolling site study personnel | |
| Elinita | Rosseto | | | Beth Israel Deaconess Medical Center | Boston, MA, USA | Enrolling site study personnel | |
| Douglas | Scaffidi | | | Beth Israel Deaconess Medical Center | Boston, MA, USA | Enrolling site study personnel | |
| Nathan | Shapiro | | MD, MPH | Beth Israel Deaconess Medical Center | Boston, MA, USA | Enrolling site study personnel | |
| Jonathan | Pak | | MD | Oregon Health and Science University | Portland, OR, USA | Enrolling site study personnel | |
| Gopal | Allada | | MD | Oregon Health and Science University | Portland, OR, USA | Enrolling site study personnel | |
| Genesis | Briceno | | MD | Oregon Health and Science University | Portland, OR, USA | Enrolling site study personnel | |
| Jose | Peña | | MD | Oregon Health and Science University | Portland, OR, USA | Enrolling site study personnel | |
| Minn | Oh | | PhD | Oregon Health and Science University | Portland, OR, USA | Enrolling site study personnel | |
| Michelle | Gong | | MD | Moses Hospital - Montefiore Medical Center | Bronx, NY, USA | Enrolling site study personnel | |
| Amira | Mohamed | | | Moses Hospital - Montefiore Medical Center | Bronx, NY, USA | Enrolling site study personnel | |
| Luke | Andrea | | | Moses Hospital - Montefiore Medical Center | Bronx, NY, USA | Enrolling site study personnel | |

*First name, last name, and suffix (if applicable) are required and will appear in PubMed.

| ***First Name and Middle Initial(s)** | ***Last Name** | ***Suffix (eg, Jr, III)** | Academic Degrees | Institution | Location (city, state/province, country) | Role or Contribution, eg, chair, principal investigator | Group (if more than 1 Group listed in the byline) and/or Subgroup (eg, Steering Committee) |
|---|---|---|---|---|---|---|---|
| Rahul | Nair | | | Moses Hospital - Montefiore Medical Center | Bronx, NY, USA | Enrolling site study personnel | |
| William | Nkemdirim | | | Moses Hospital - Montefiore Medical Center | Bronx, NY, USA | Enrolling site study personnel | |
| Sabah | Boujid | | | Moses Hospital - Montefiore Medical Center | Bronx, NY, USA | Enrolling site study personnel | |
| Martha | Torres | | | Moses Hospital - Montefiore Medical Center | Bronx, NY, USA | Enrolling site study personnel | |
| Ofelia | Garcia | | | Moses Hospital - Montefiore Medical Center | Bronx, NY, USA | Enrolling site study personnel | |
| Harith | Ali | | MBchB | Johns Hopkins University | Baltimore, MD, USA | Enrolling site study personnel | |
| Sasha | Beselman | | PharmD, MBA | Johns Hopkins University | Baltimore, MD, USA | Enrolling site study personnel | |
| Yolanda | Eby | | MS | Johns Hopkins University | Baltimore, MD, USA | Enrolling site study personnel | |
| Vitaliy | Klimov | | PharmD | Johns Hopkins University | Baltimore, MD, USA | Enrolling site study personnel | |
| R. Duncan | Hite | | MD | University of Cincinnati | Cincinnati, OH, USA | Enrolling site study personnel | |
| Hammad | Tanzeem | | | University of Cincinnati | Cincinnati, OH, USA | Enrolling site study personnel | |
| Chris | Droege | | | University of Cincinnati | Cincinnati, OH, USA | Enrolling site study personnel | |
| Jessica | Winter | | | University of Cincinnati | Cincinnati, OH, USA | Enrolling site study personnel | |
| Simon | Mucha | | MD | Cleveland Clinic Foundation/Cleveland Clinic Fairview Hospital | Cleveland, OH, USA | Enrolling site study personnel | |

*First name, last name, and suffix (if applicable) are required and will appear in PubMed.

| ***First Name and Middle Initial(s)** | ***Last Name** | ***Suffix (eg, Jr, III)** | Academic Degrees | Institution | Location (city, state/province, country) | Role or Contribution, eg, chair, principal investigator | Group (if more than 1 Group listed in the byline) and/or Subgroup (eg, Steering Committee) |
|---|---|---|---|---|---|---|---|
| Nirosshan | Thiruchelvam | | MD | Cleveland Clinic Foundation/Cleveland Clinic Fairview Hospital | Cleveland, OH, USA | Enrolling site study personnel | |
| Matthew | Siuba | | MD | Cleveland Clinic Foundation/Cleveland Clinic Fairview Hospital | Cleveland, OH, USA | Enrolling site study personnel | |
| Omar | Mehkri | | MD | Cleveland Clinic Foundation/Cleveland Clinic Fairview Hospital | Cleveland, OH, USA | Enrolling site study personnel | |
| Susan | Jackman | | | Cedars-Sinai Medical Center | Los Angeles, CA, USA | Enrolling site study personnel | |
| Antonina | Caudill | | | Cedars-Sinai Medical Center | Los Angeles, CA, USA | Enrolling site study personnel | |
| Emad | Bayoumi | | | Cedars-Sinai Medical Center | Los Angeles, CA, USA | Enrolling site study personnel | |
| Ethan | Pascual | | | Cedars-Sinai Medical Center | Los Angeles, CA, USA | Enrolling site study personnel | |
| Po-En | Chen | | | Cedars-Sinai Medical Center | Los Angeles, CA, USA | Enrolling site study personnel | |
| Sam S. | Torbati | | MD | Cedars-Sinai Medical Center | Los Angeles, CA, USA | Enrolling site study personnel | |
| Tanyalak | Parimon | | MD | Cedars-Sinai Medical Center | Los Angeles, CA, USA | Enrolling site study personnel | |
| Bradley | Rosen | | MD | Cedars-Sinai Medical Center | Los Angeles, CA, USA | Enrolling site study personnel | |
| Yuri | Matusov | | MD | Cedars-Sinai Medical Center | Los Angeles, CA, USA | Enrolling site study personnel | |
| Michael J. | Lanspa | | | Intermountain Healthcare | Salt Lake City, UT, USA | Enrolling site study personnel | |
| Ithan D. | Peltan | | | Intermountain Healthcare | Salt Lake City, UT, USA | Enrolling site study personnel | |

Supplemental Online Content: ACTIV-4 Host Tissue Investigators
*First name, last name, and suffix (if applicable) are required and will appear in PubMed.

| ***First Name and Middle Initial(s)** | ***Last Name** | ***Suffix (eg, Jr, III)** | Academic Degrees | Institution | Location (city, state/province, country) | Role or Contribution, eg, chair, principal investigator | Group (if more than 1 Group listed in the byline) and/or Subgroup (eg, Steering Committee) |
|---|---|---|---|---|---|---|---|
| Samuel M. | Brown | | | Intermountain Healthcare | Salt Lake City, UT, USA | Enrolling site study personnel | |
| Jason R. | Carr | | | Intermountain Healthcare | Salt Lake City, UT, USA | Enrolling site study personnel | |
| Daniel B. | Knox | | | Intermountain Healthcare | Salt Lake City, UT, USA | Enrolling site study personnel | |
| Lindsay M. | Leither | | | Intermountain Healthcare | Salt Lake City, UT, USA | Enrolling site study personnel | |
| Brenda | Lopez | | | Jack D. Weiler Hospital - Montefiore Medical Center | Bronx, NY, USA | Enrolling site study personnel | |
| Benjamin | Galen | | MD | Jack D. Weiler Hospital - Montefiore Medical Center | Bronx, NY, USA | Enrolling site study personnel | |
| Nina | Gentile | | MD | Temple University | Philadelphia, PA, USA | Enrolling site study personnel | |
| Derek | Isenberg | | MD | Temple University | Philadelphia, PA, USA | Enrolling site study personnel | |
| Hannah | Reimer | | BSN | Temple University | Philadelphia, PA, USA | Enrolling site study personnel | |
| Paul | Cincola | | BA | Temple University | Philadelphia, PA, USA | Enrolling site study personnel | |
| Estelle S. | Harris | | MD | University of Utah Health Sciences Center | Salt Lake City, UT, USA | Enrolling site study personnel | |
| Sean J. | Callahan | | MD | University of Utah Health Sciences Center | Salt Lake City, UT, USA | Enrolling site study personnel | |
| Misty B. | Yamane | | BS | University of Utah Health Sciences Center | Salt Lake City, UT, USA | Enrolling site study personnel | |
| Macy AG | Barrios | | BS | University of Utah Health Sciences Center | Salt Lake City, UT, USA | Enrolling site study personnel | |
| Neeraj | Desai | | MD | Alexian Brothers Medical Center | Elk Grove Village, IL, USA | Enrolling site study personnel | |

Supplemental Online Content: ACTIV-4 Host Tissue Investigators
*First name, last name, and suffix (if applicable) are required and will appear in PubMed.

| ***First Name and Middle Initial(s)** | ***Last Name** | ***Suffix (eg, Jr, III)** | Academic Degrees | Institution | Location (city, state/province, country) | Role or Contribution, eg, chair, principal investigator | Group (if more than 1 Group listed in the byline) and/or Subgroup (eg, Steering Committee) |
|---|---|---|---|---|---|---|---|
| Amit | Bharara | | MD | Alexian Brothers Medical Center | Elk Grove Village, IL, USA | Enrolling site study personnel | |
| Michael | Keller | | MD | Alexian Brothers Medical Center | Elk Grove Village, IL, USA | Enrolling site study personnel | |
| Prat | Majumder | | MD | Alexian Brothers Medical Center | Elk Grove Village, IL, USA | Enrolling site study personnel | |
| Carrie | Dohe | | | Alexian Brothers Medical Center | Elk Grove Village, IL, USA | Enrolling site study personnel | |
| Kathryn | Hibbert | | MD | Massachusetts General Hospital | Boston, MA, USA | Enrolling site study personnel | |
| Justin | Margolin | | BS | Massachusetts General Hospital | Boston, MA, USA | Enrolling site study personnel | |
| Blair | Parry | | CCRC, BA | Massachusetts General Hospital | Boston, MA, USA | Enrolling site study personnel | |
| Mark A. | Tidswell | | | Baystate Health | Springfield, MA, USA | Enrolling site study personnel | |
| Jay S. | Steingrub | | | Baystate Health | Springfield, MA, USA | Enrolling site study personnel | |
| Lesley | De Souza | | | Baystate Health | Springfield, MA, USA | Enrolling site study personnel | |
| Cynthia | Kardos | | | Baystate Health | Springfield, MA, USA | Enrolling site study personnel | |
| Denise | Gallant | | | Baystate Health | Springfield, MA, USA | Enrolling site study personnel | |
| Rae Lynn | DeFoe | | | Baystate Health | Springfield, MA, USA | Enrolling site study personnel | |
| Sara | Romain | | | Baystate Health | Springfield, MA, USA | Enrolling site study personnel | |
| Scott | Ouellette | | | Baystate Health | Springfield, MA, USA | Enrolling site study personnel | |

*First name, last name, and suffix (if applicable) are required and will appear in PubMed.

| ***First Name and Middle Initial(s)** | ***Last Name** | ***Suffix (eg, Jr, III)** | Academic Degrees | Institution | Location (city, state/province, country) | Role or Contribution, eg, chair, principal investigator | Group (if more than 1 Group listed in the byline) and/or Subgroup (eg, Steering Committee) |
|---|---|---|---|---|---|---|---|
| Serena | Estrada | | | Baystate Health | Springfield, MA, USA | Enrolling site study personnel | |
| Ryan | Kindle | | | Baystate Health | Springfield, MA, USA | Enrolling site study personnel | |
| Bogdan | Tiru | | | Baystate Health | Springfield, MA, USA | Enrolling site study personnel | |
| Howard | Smithline | | | Baystate Health | Springfield, MA, USA | Enrolling site study personnel | |
| Carolyn | Garcia | | | Baystate Health | Springfield, MA, USA | Enrolling site study personnel | |
| Charles | Terry | | MD, MSCR | Medical University of South Carolina | Charleston, SC, USA | Enrolling site study personnel | |
| Melissa | Blender | | | Medical University of South Carolina | Charleston, SC, USA | Enrolling site study personnel | |
| Abbey | Grady | | | Medical University of South Carolina | Charleston, SC, USA | Enrolling site study personnel | |
| Andrew | Powell | | BS | University of North Carolina | Chapel Hill, NC, USA | Enrolling site study personnel | |
| Peter C. | Hou | | | Brigham and Women's Hospital | Boston, MA, USA | Enrolling site study personnel | |
| Mohammad A. | Hasdianda | | | Brigham and Women's Hospital | Boston, MA, USA | Enrolling site study personnel | |
| Seven E. | Miyawaki | | | Brigham and Women's Hospital | Boston, MA, USA | Enrolling site study personnel | |
| Mikita | Umale | | | Brigham and Women's Hospital | Boston, MA, USA | Enrolling site study personnel | |
| Brandon Lee | | | | Brigham and Women's Hospital | Boston, MA, USA | Enrolling site study personnel | |
| Valeria D. | Cantos | | | Grady Memorial Hospital/Ponce de Leon CRS | Atlanta, GA, USA | Enrolling site study personnel | |

*First name, last name, and suffix (if applicable) are required and will appear in PubMed.

| ***First Name and Middle Initial(s)** | ***Last Name** | ***Suffix (eg, Jr, III)** | Academic Degrees | Institution | Location (city, state/province, country) | Role or Contribution, eg, chair, principal investigator | Group (if more than 1 Group listed in the byline) and/or Subgroup (eg, Steering Committee) |
|---|---|---|---|---|---|---|---|
| Colleen F. | Kelley | | | Grady Memorial Hospital/Ponce de Leon CRS | Atlanta, GA, USA | Enrolling site study personnel | |
| Paulina A. | Rebolledo | | | Grady Memorial Hospital/Ponce de Leon CRS | Atlanta, GA, USA | Enrolling site study personnel | |
| Sheetal | Kandiah | | | Grady Memorial Hospital/Ponce de Leon CRS | Atlanta, GA, USA | Enrolling site study personnel | |
| Renee | Cook | | | Grady Memorial Hospital/Ponce de Leon CRS | Atlanta, GA, USA | Enrolling site study personnel | |
| Betsy | Hall | | | Grady Memorial Hospital/Ponce de Leon CRS | Atlanta, GA, USA | Enrolling site study personnel | |
| Christin | Root | | | Grady Memorial Hospital/Ponce de Leon CRS | Atlanta, GA, USA | Enrolling site study personnel | |
| Juliet | Brown | | | Grady Memorial Hospital/Ponce de Leon CRS | Atlanta, GA, USA | Enrolling site study personnel | |
| Christopher | Foster | | | Grady Memorial Hospital/Ponce de Leon CRS | Atlanta, GA, USA | Enrolling site study personnel | |
| Philip | Powers | | | Grady Memorial Hospital/Ponce de Leon CRS | Atlanta, GA, USA | Enrolling site study personnel | |
| Pamela | Lankford-Turner | | | Grady Memorial Hospital/Ponce de Leon CRS | Atlanta, GA, USA | Enrolling site study personnel | |
| Bela | Patel | | MD | University of Texas, Houston | Houston, TX, USA | Enrolling site study personnel | |
| Rodeo | Abrencillo | | MD | University of Texas, Houston | Houston, TX, USA | Enrolling site study personnel | |
| Robier | Anguillon-Prada | | MD | University of Texas, Houston | Houston, TX, USA | Enrolling site study personnel | |
| Ryan | Huebinger | | MD | University of Texas, Houston | Houston, TX, USA | Enrolling site study personnel | |
| Gabriel | Patarroyo Aponte | | MD | University of Texas, Houston | Houston, TX, USA | Enrolling site study personnel | |

Supplemental Online Content: ACTIV-4 Host Tissue Investigators
*First name, last name, and suffix (if applicable) are required and will appear in PubMed.

| ***First Name and Middle Initial(s)** | ***Last Name** | ***Suffix (eg, Jr, III)** | Academic Degrees | Institution | Location (city, state/province, country) | Role or Contribution, eg, chair, principal investigator | Group (if more than 1 Group listed in the byline) and/or Subgroup (eg, Steering Committee) |
|---|---|---|---|---|---|---|---|
| Syed | Ghazi Rizvi | | MD | University of Texas, Houston | Houston, TX, USA | Enrolling site study personnel | |
| Elizabeth | Vidales | | | University of Texas, Houston | Houston, TX, USA | Enrolling site study personnel | |
| Idorenyin | Udoh-Bradford | | | University of Texas, Houston | Houston, TX, USA | Enrolling site study personnel | |
| Mar | Masiá | | | Hospital General Universitario de Elche | Elche, Spain | Enrolling site study personnel | |
| Sergio | Padilla | | | Hospital General Universitario de Elche | Elche, Spain | Enrolling site study personnel | |
| Guillermo | Telenti | | | Hospital General Universitario de Elche | Elche, Spain | Enrolling site study personnel | |
| Paula | Mascarell | | | Hospital General Universitario de Elche | Elche, Spain | Enrolling site study personnel | |
| Javier | García-Abellán | | | Hospital General Universitario de Elche | Elche, Spain | Enrolling site study personnel | |
| Ángela | Botella | | | Hospital General Universitario de Elche | Elche, Spain | Enrolling site study personnel | |
| Catalina | Robledano | | | Hospital General Universitario de Elche | Elche, Spain | Enrolling site study personnel | |
| Vincente | Estrada | | | Hospital Clinico San Carlos | Lagos, Spain | Enrolling site study personnel | |
| Reynaldo | Homen | | | Hospital Clinico San Carlos | Lagos, Spain | Enrolling site study personnel | |
| Javier | Rodriguez-Añover | | | Hospital Clinico San Carlos | Lagos, Spain | Enrolling site study personnel | |
| Joaquin | Burgos | | MD | Hospital Universitario Vall dHebron (VHIR) | Barcelona, Spain | Enrolling site study personnel | |
| Tom | Boyles | | MD | Clinical HIV Research Unit - Helen Joseph Hospital (WITS CHRU) | Johannesburg, South Africa | Enrolling site study personnel | |

*First name, last name, and suffix (if applicable) are required and will appear in PubMed.

| *First Name and Middle Initial(s) | *Last Name | *Suffix (eg, Jr, III) | Academic Degrees | Institution | Location (city, state/province, country) | Role or Contribution, eg, chair, principal investigator | Group (if more than 1 Group listed in the byline) and/or Subgroup (eg, Steering Committee) |
|---|---|---|---|---|---|---|---|
| Zanele | Mkhabela | | | Clinical HIV Research Unit - Helen Joseph Hospital (WITS CHRU) | Johannesburg, South Africa | Enrolling site study personnel | |
| Galadriel | Pellejero | | | Hospital Clinico Universitario Lozano Blesa | Zaragoza, Spain | Enrolling site study personnel | |
| Elena | Morte-Romea | | | Hospital Clinico Universitario Lozano Blesa | Zaragoza, Spain | Enrolling site study personnel | |
| Santiago | Letona-Carbajo | | | Hospital Clinico Universitario Lozano Blesa | Zaragoza, Spain | Enrolling site study personnel | |
| María | José Esquillor | | | Hospital Clinico Universitario Lozano Blesa | Zaragoza, Spain | Enrolling site study personnel | |
| Carla | Toyas | | | Hospital Clinico Universitario Lozano Blesa | Zaragoza, Spain | Enrolling site study personnel | |
| Silvia | Loscos | | | Hospital Clinico Universitario Lozano Blesa | Zaragoza, Spain | Enrolling site study personnel | |
| Nazreen | Hussen | | MD | Worthwhile Clinical Trials (WWCT Lakeview Hospital) | Benoni, South Africa | Enrolling site study personnel | |
| Carlos | Guijarro | | MD | Hospital Universitario Fundacion Alcorcon | Madrid, Spain | Enrolling site study personnel | |
| Maria | Velasco-Arribas | | MD | Hospital Universitario Fundacion Alcorcon | Madrid, Spain | Enrolling site study personnel | |
| Esaú | Custódio João Filho | | | Hospital Federal dos Servidores do Estado | Madrid, Spain | Enrolling site study personnel | |
| Elaine C. | de Oliveira Souza | | | Hospital Federal dos Servidores do Estado | Madrid, Spain | Enrolling site study personnel | |
| Christoph | Boesecke | | MD | University Hospital Bonn | Bonn, Germany | Enrolling site study personnel | |
| Jurgen | Rockstroh | | MD | University Hospital Bonn | Bonn, Germany | Enrolling site study personnel | |
| Marco | Ripa | | MD | San Raffaele Turro Hospital | Milan, Italy | Enrolling site study personnel | |

*First name, last name, and suffix (if applicable) are required and will appear in PubMed.

| ***First Name and Middle Initial(s)** | ***Last Name** | ***Suffix (eg, Jr, III)** | Academic Degrees | Institution | Location (city, state/province, country) | Role or Contribution, eg, chair, principal investigator | Group (if more than 1 Group listed in the byline) and/or Subgroup (eg, Steering Committee) |
|---|---|---|---|---|---|---|---|
| Silvia | Nozza | | MD | San Raffaele Turro Hospital | Milan, Italy | Enrolling site study personnel | |
