# Supplementary material for: Fostamatinib for Hospitalized Adults With COVID-19 and Hypoxemia: A Randomized Clinical Trial
Source: JAMA Netw Open. 2024 Dec 3;7(12):e2448215. doi: 10.1001/jamanetworkopen.2024.48215 (PMC11615712; doi:10.1001/jamanetworkopen.2024.48215)
Supplement: Supplement 4. — Data Sharing Statement [file jamanetwopen-e2448215-s004.pdf]

Data Sharing Statement

Collins. Fostamatinib for Hospitalized Adults With COVID-19 and Hypoxemia. *JAMA Netw Open*. Published December 03, 2024. doi:10.1001/jamanetworkopen.2024.48215

Data
Additional Information: ClinicalTrials.gov number: NCT04924660
Data available: Yes
Data types: Deidentified participant data
How to access data: https://www.ncbi.nlm.nih.gov/gap/study/status/56326
When available: With publication

Supporting Documents
Document types: Statistical/analytic code
How to access documents: https://www.ncbi.nlm.nih.gov/gap/study/status/56326
When available: With publication

Additional Information
Who can access the data: anyone requesting data
Types of analyses: any purpose
Mechanisms of data availability: by the enclosed URL
